# Overlapping but distinct topology for zebrafish V2R-like olfactory receptors reminiscent of odorant receptor spatial expression zones

**DOI:** 10.1186/s12864-018-4740-8

**Published:** 2018-05-23

**Authors:** Gaurav Ahuja, Vera Reichel, Daniel Kowatschew, Adnan S. Syed, Aswani Kumar Kotagiri, Yuichiro Oka, Franco Weth, Sigrun I. Korsching

**Affiliations:** 1 Institute of Genetics, University at Cologne, Zülpicher Str. 47A, 50674 Cologne, Germany; 2 Present address: Department of Anatomy and Neuroscience, Graduate School of Medicine, Osaka University, 2-2 Yamadaoka, Suita, Osaka, 565-0871 Japan; 3 Present address: Department of Child Development, United Graduate School of Child Development, Osaka University, Kanazawa University, Hamamatsu University School of Medicine, Chiba University and University of Fukui, 2-2 Yamadaoka, Suita, Osaka, 565-0871 Japan; 4 Karlsruher Institut fuer Technologie (KIT) - Campus Sued, Zoologisches Institut, Abteilung fuer Zell- und Neurobiologie, Fritz-Haber-Weg 4, 76131 Karlsruhe, Germany; 5 Present address: Center for Molecular Medicine Cologne (ZMMK), Robert-Koch-Str. 21, 50931 Cologne, Germany; 6 Present address: Max Planck Institute for Biology of Ageing, Joseph-Stelzmann-Str. 9b, 50931 Cologne, Germany

**Keywords:** In situ hybridization, Spatial distribution, Olfactory receptors, V2Rs, Microvillous neurons, Zebrafish

## Abstract

**Background:**

The sense of smell is unrivaled in terms of molecular complexity of its input channels. Even zebrafish, a model vertebrate system in many research fields including olfaction, possesses several hundred different olfactory receptor genes, organized in four different gene families. For one of these families, the initially discovered odorant receptors proper, segregation of expression into distinct spatial subdomains within a common sensory surface has been observed both in teleost fish and in mammals. However, for the remaining three families, little to nothing was known about their spatial coding logic. Here we wished to investigate, whether the principle of spatial segregation observed for odorant receptors extends to another olfactory receptor family, the V2R-related *OlfC* genes. Furthermore we thought to examine, how expression of *OlfC* genes is integrated into expression zones of odorant receptor genes, which in fish share a single sensory surface with *OlfC* genes.

**Results:**

To select representative genes, we performed a comprehensive phylogenetic study of the zebrafish *OlfC* family, which identified a novel *OlfC* gene, reduced the number of pseudogenes to 1, and brought the total family size to 60 intact OlfC receptors. We analyzed the spatial pattern of *OlfC*-expressing cells for seven representative receptors in three dimensions (height within the epithelial layer, horizontal distance from the center of the olfactory organ, and height within the olfactory organ). We report non-random distributions of labeled neurons for all *OlfC* genes analysed. Distributions for sparsely expressed *OlfC* genes are significantly different from each other in nearly all cases, broad overlap notwithstanding. For two of the three coordinates analyzed, OlfC expression zones are intercalated with those of odorant receptor zones, whereas in the third dimension some segregation is observed.

**Conclusion:**

Our results show that V2R-related *OlfC* genes follow the same spatial logic of expression as odorant receptors and their expression zones intermingle with those of odorant receptor genes. Thus, distinctly different expression zones for individual receptor genes constitute a general feature shared by teleost and tetrapod V2R/OlfC and odorant receptor families alike.

**Electronic supplementary material:**

The online version of this article (10.1186/s12864-018-4740-8) contains supplementary material, which is available to authorized users.

## Background

The sense of smell is unrivaled in terms of molecular complexity of its input channels. Several hundred to over two thousand different receptor genes convey olfactory signals in mammals [1–3]. Nearly all of these olfactory receptors belong to one of four different gene families, the initially discovered odorant receptors proper (ORs, [4]), two types of vomeronasal receptors (V1Rs and V2R, respectively), and the trace amine-associated receptors (TAARs), all of which have counterparts in the teleost olfactory system (ORs, ORAs, OlfCs, and TAARs, respectively [5]. Generally, out of this large repertoire only a single olfactory receptor gene is expressed in any particular olfactory sensory neuron [6, 7]. Olfaction is different from most other senses in that neurons with the same sensivity, i.e. expressing the same sensory receptor, are scattered within the sensory surface [8–10]. Nevertheless, qualitative as well as quantitative analysis of expression patterns of rodent and zebrafish odorant receptors has shown that this scattering is not completely random, but that different ORs segregate into distinct spatial subdomains within a common sensory surface [8, 10, 11].

Some borders between subdomains appear to be rather sharp, e.g. between zone I and II in the mammalian olfactory epithelium [8, 9, 12], but in many cases expression zones of different genes overlap widely [10, 11]. It has been suggested that sharply delineated subdivisions serve to segregate receptor groups with different biological functions or with different target regions in the olfactory bulb [13]. However it is difficult to make the same argument for receptor genes with extensively overlapping expression zones. Interestingly chromosomal location maps to some extent to expression zone [14] suggesting potential ways how a spatial segregation of expression might be generated by the olfactory system.

So far it is not known, whether other olfactory receptor families besides ORs show similar segregation into expression zones, although an initial report in rodents points to this possibility for V2Rs [15]. Moreover, in the fish olfactory system all four olfactory receptor gene families are expressed in a single sensory surface, but it is not known, how the other receptor gene families integrate into the spatial expression patterns found for odorant receptors [10]. The cell types expressing ORs and V2R-related OlfC receptors (ciliated and microvillous neurons, respectively) are intermingled in the zebrafish olfactory epithelium [16], although initial qualitative assessment suggested microvillous neurons to lie more apical (closer to the lumen) than ciliated neurons [17, 18]. We have recently developed a thorough analysis method to quantify and compare three-dimensional spatial distribution patterns observed for different receptor neuron populations and receptor genes [19–21]. Here we use this method to analyse expression patterns for seven *OlfC* genes, chosen as representative based on a rigorous phylogenetic analysis of the zebrafish *OlfC* gene family. We have performed in situ hybridization for all seven genes, and have quantified the positions of cells expressing these genes in three dimensions. We observe non-random, distinctly different expression zones for different zebrafish V2R-related OlfC receptors that intercalate into those described for odorant receptors. This spatial logic thus constitutes a general feature shared by teleost and tetrapod V2R/OlfC and OR receptor families.

## Results

### Zebrafish OlfC family consists of 60 intact genes and 1 pseudogene

**Fig. 1:**
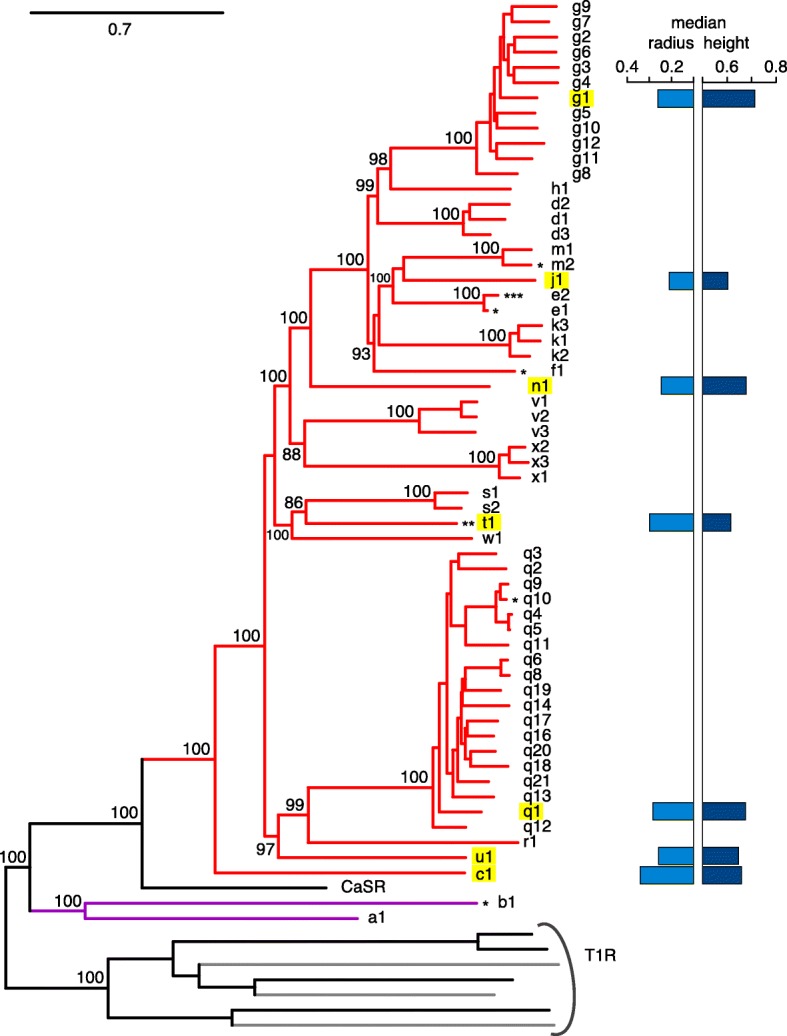
Selection of seven representative genes from a phylogenetic tree of the zebrafish OlfC family. A phylogenetic tree of 60 full length *OlfC* gene sequences was constructed using a maximum likelihood (ML) algorithm (see Methods for details). Bootstrap support in percent is indicated at relevant nodes. As in previous analyses, the OlfC family appears polyphyletic, with the calcium sensor gene CaSR intercalating between OlfCa1, OlfCb1, and the remainder of the OlfC family. Mouse and zebrafish T1R taste receptors were used as outgroup. Single asterisk, gene was predicted in [23] as pseudogene (*OlfCb1*, *e1, q10*) or fragment (*OlfCf1, m2*), but is intact and full length in the current prediction; double asterisk, gene is lost in GRCz10; triple asterisk, novel gene. *OlfC* genes highlighted with yellow were selected for expression analysis. To the right of the tree the core cell distribution parameters for the genes analysed are shown as bar graphs. Light blue, median radial position; dark blue, median height position, all values normalized to the respective maximal values. No correlation is apparent between position in the phylogenetic tree and median radius or height

In previous studies the size of the zebrafish *OlfC* gene family was given as 46 [22] or 54 intact genes [23], additionally several incomplete and pseudogenes were reported. We performed extensive Blast searches in the latest zebrafish genome assembly (GRCz10), using representative zebrafish OlfC amino acid sequences as templates. *OlfC* genes were identified by their position in the phylogenetic tree, using the closely related calcium sensor and *t1r* taste receptor genes as outgroup (Fig. 1).

**Fig. 2:**
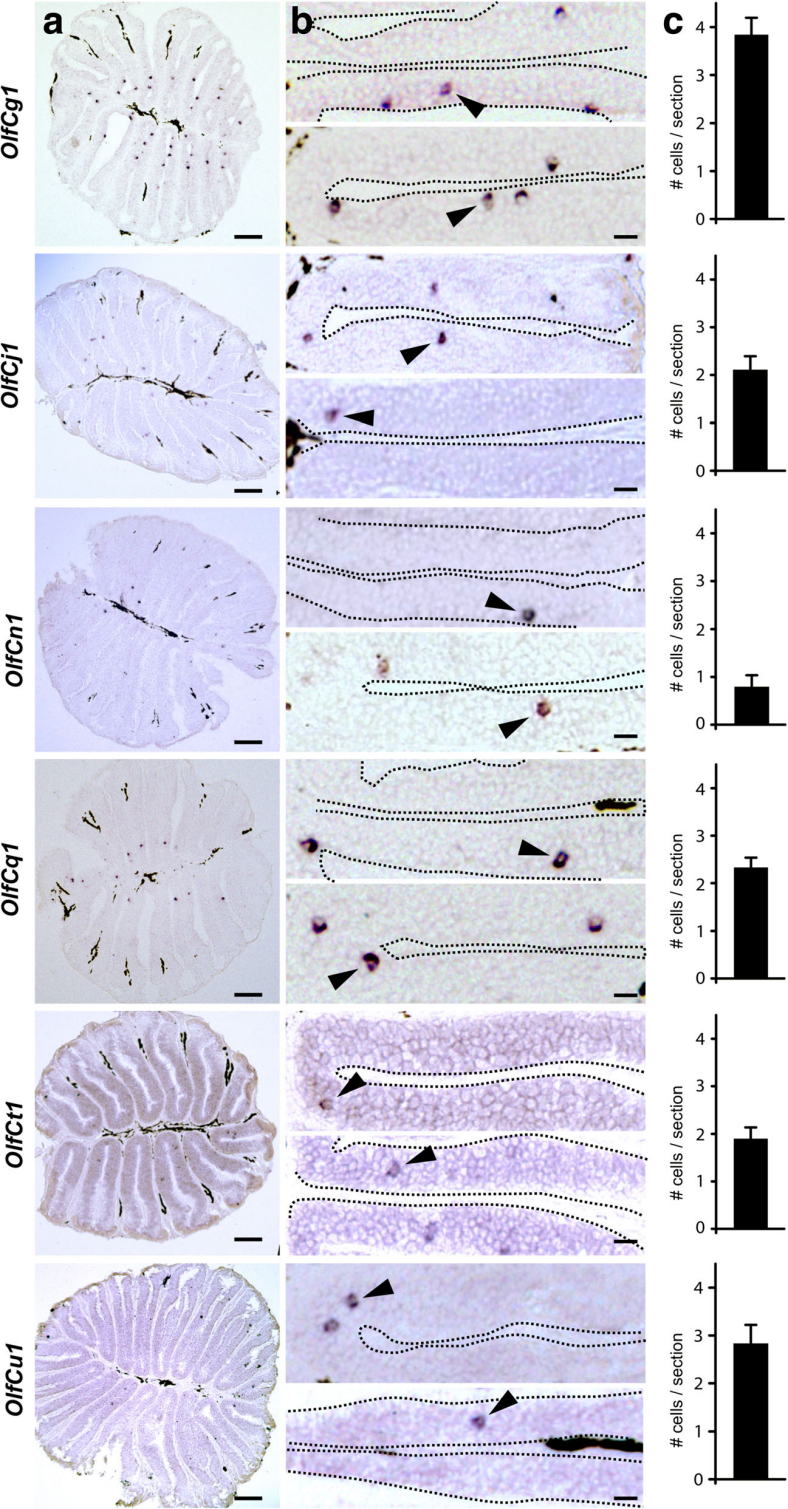
v2r-related *OlfC* genes generally are expressed in small subsets of scattered olfactory sensory neurons. Horizontal sections of adult zebrafish olfactory epithelium were hybridized with probes for *OlfCg1*, *OlfCn1*, *OlfCq1*, *OlfCj1*, *OlfCu1*, and *OlfCt1*. Column **a** shows representative complete sections labeled with the respective probes. The scale bars correspond to 40 μm. Column **b**, higher magnifications from different sections. The hybridization signal was observed in sparse cells within the sensory region of the olfactory epithelium, as expected; arrowheads point to some labeled neurons. The scale bars correspond to 20 μm. **c** Bar graphs representing number of labeled cells per section for each *OlfC* gene (mean +/− SEM, *n* = 78–265 sections/gene)

We report 60 intact zebrafish *OlfC* genes and one pseudo gene (Fig. 1, Additional file 1). One gene was newly identified, and named for its close homology to *OlfCe1* as *OlfCe2*. Four sequences formerly reported as pseudogenes or fragments were identified as intact and full length and were renamed accordingly. The sequences of the novel gene and the corrected predictions are given in (Additional file 2). The higher number of pseudogenes and fragments in previous studies may reflect inadequacies of the earlier versions of the genome assembly used in those studies. Unexpectedly, one gene, *OlfCt1,* is absent from the current assembly, although it was present in earlier versions. Since we cloned the gene from zebrafish DNA and could demonstrate specific in situ hybridization signals (Fig. 2), we assume an erroneous curation of the current assembly as most likely cause. In the phylogenetic analysis the *OlfC* family is paraphyletic, with *OlfCa1* and *OlfCb1* ancestral to the calcium sensor, which itself is ancestral to the main group of *OlfC* genes (Fig. 1). All subfamilies suggested by [23] were confirmed with very high branch support (Fig. 1). Based on the phylogenetic analysis we selected seven representative genes for analysis of expression patterns, including the hypothesized co-receptor OlfCc1 [18], two genes that are members of large gene expansions (g1 and q1), and four relatively isolated genes (j1, n1, u1, t1).

### Main group zebrafish OlfC genes are expressed in sparse populations of sensory neurons

To analyse the spatial distribution of *OlfC*-expressing cells, we have performed in situ hybridization with cRNA probes on complete series of horizontal cryostat sections from adult zebrafish olfactory epithelia. Cross-reactivity of probes to other genes is not expected, since nucleotide identity of probes to the closest neighbor gene was below 80%, cf. [24, 25] in all but one case (*OlfCg1* probe 80.7% identical to *OlfCg7*). For each gene, 3–5 complete epithelia were taken from different adult zebrafishes of similar age (8–10 months).

*OlfCc1* is expressed in a large population of olfactory sensory neurons (Additional file 3), consistent with earlier observations [18]. *OlfCc1* is an ortholog of murine v*mn2r1*, a hypothesized co-receptor, and indeed recently has been shown to co-express with other OlfC receptors in microvillous sensory neurons [18]. In contrast, all six genes from the main OlfC clade labeled sparse populations of olfactory sensory neurons within the sensory surface of the olfactory epithelium (Fig. 2). The frequency of labeled cells ranged between 60 to 180 per olfactory organ, similar to frequencies observed for expression of ORs [10] (Additional file 3). The average number of labeled cells per section ranges between 0.8 and 3.8 for different *OlfC* genes (Fig. 2c), again within the range of frequencies reported for other olfactory receptor genes in zebrafish [19, 21]. At first glance the spatial expression pattern of all tested *OlfC* genes from the main clade looked rather similar. We therefore performed quantitative analysis of spatial expression patterns in three dimensions (height within lamella, radial distance, height within organ) to examine if and how distributions for different genes differ.

### Three different expression zones distinguishable in analysis of laminar height

We quantified the laminar height (height of the neuronal soma within the epithelial layer) as relative height (h_rel_ = h _soma center_/_thickness of sensory layer_; 0, basal;1, apical, see Additional file 3 for a graphical visualization of the parameter) for all tested *OlfC* genes as well as *omp* and *trpc2*, which serve as cell type markers for ciliated and microvillous receptor neurons, respectively. The laminar height is characteristically different between all four populations of olfactory sensory neurons, ciliated, microvillous, crypt and kappe neurons [20]. However, it is not known, whether the distribution for individual receptors is identical to that of their corresponding cell type, as the distribution for the cell type might be composed of several different and more narrow distributions for individual receptors. To evaluate similarity and dissimilarity of distributions, we evaluated median values, half width, and maximal vertical distance in pairwise comparisons. The median value is less sensitive to outliers and skewed data than the arithmetic mean and constitutes thus a more robust measure of the center-of-gravity of the respective distribution within the sensory surface. The half width is a measure for the broadness of the respective distribution, and was estimated as difference between 1st and 3rd quartile. The maximal vertical distance between two distributions is a measure for the degree of difference between two distributions, and is measured as the maximal difference in their respective cumulative distribution functions.

**Fig. 3:**
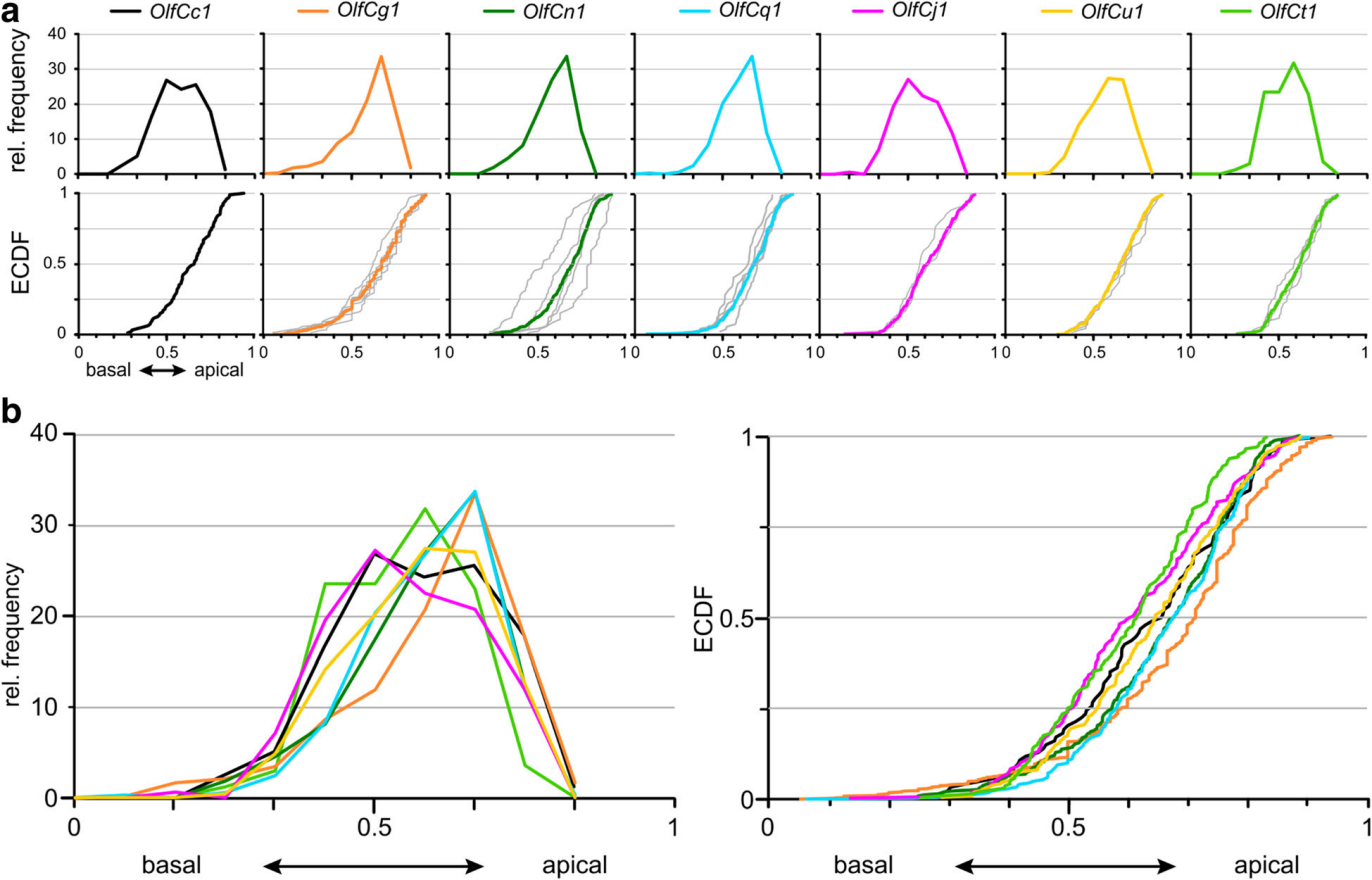
Quantitative assessment of laminar height distributions of v2r-related OlfC-expressing neurons. Laminar height of OlfC-expressing cells was quantified for seven OlfC genes, including OlfCc1. Complete series of sections from three to five olfactory epithelia were evaluated for each *OlfC* gene. Height within the lamina was normalized to maximal laminar thickness. **a** The resulting distributions of relative laminar height (from 0, most basal to 1, most apical, i.e. bordering to the lumen) are shown binned (histogram, top row) and unbinned (empirical cumulative distribution function, ECDF, bottom row). The color code for the OlfC genes is the same as for later figures to facilitate comparisons between different positional parameters. Light grey curves in the ECDF plot for each of the tested *OlfC* genes represent the distribution for individual olfactory organs. Due to the smaller number of cells, scatter is increased. **b** Overlay of the seven distributions shown individually in panel **a**), both as histogram (left panel) and ECDF (right panel)

The height distributions for *OlfCg1*, *OlfCn1* and *OlfCq1* show a more narrow peak in the histogram representation, corresponding to a steeper slope in the empirical cumulative distribution function, ECDF, compared to the broader peaks seen for *OlfCu1*, *OlfCj1* and *OlfCt1* (Fig. 3, Additional file 4). Kolmogorov-Smirnov test suggests the distributions to fall into three groups, which are significantly different from each other (Fig. 3, Additional file 4). The first group is formed by *OlfCg1*, which is different from all five other receptors. The second group contains *OlfCn1* and *OlfCq1*, which are also different from *OlfCj1* and *OlfCt1* that form the third group. *OlfCu1* is similar to both the second and the third group.

The overall spread of the distributions is moderate (Fig. 3). Median values occupy a narrow range between 0.603 and 0.714, i.e. the difference in median height between the most apical (OlfCg1) and the most basal (OlfCj1) receptor amounts to only 11% of the total height of the epithelial layer, and the maximal vertical difference between two distributions ranges between 5 and 31% (Additional file 4).

All six distributions are significantly different from the more basal distribution of *omp*-expressing cells (Fig. 7 Additional file 4). Interestingly, the most basal and most apical distributions are also different from the distribution for *trpc2*, the microvillous marker, whereas the intermediate distributions (*OlfCn1*, *q1*, *u1*) are undistinguishable from *trpc2* (Additional file 4). This may seem surprising, but is in fact expected, if the *trpc2* distribution results from summing over a heterogenous group of distributions for individual *OlfC* genes. In such a case, only distributions for individual receptors from the middle range would be expected to be undistinguishable from the *trpc2* distribution.

### Analysis of the radial coordinate yields two further subdivisions in expression zones

**Fig. 4:**
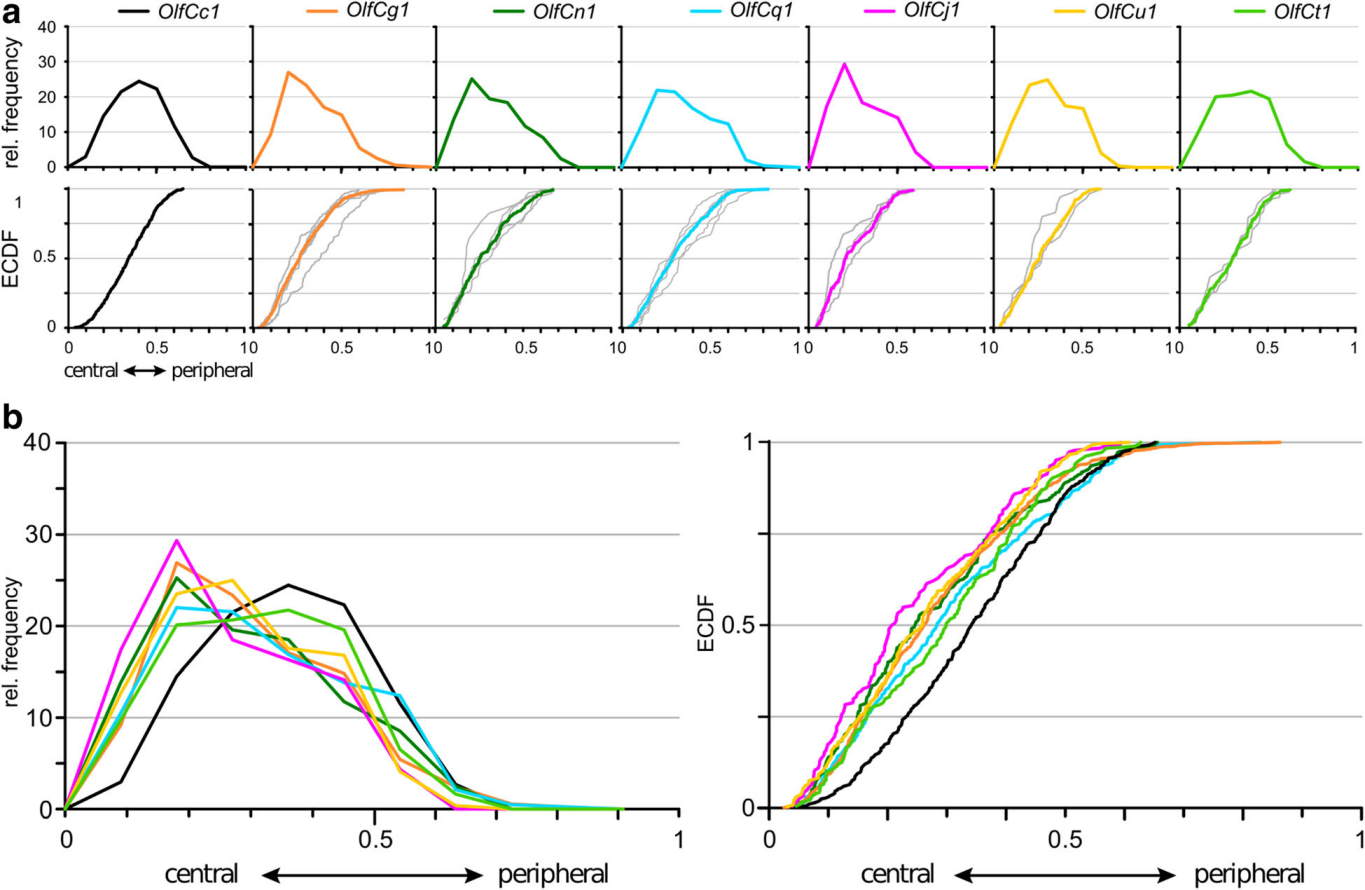
Quantitative assessment of the radial distribution of v2r-related OlfC-expressing neurons. The distribution of radial positions of OlfC-expressing cells was quantified for seven *OlfC* genes, including *OlfCc1*, using the same set of sections, for which laminar height was determined, except the very first sections, where the sensory surface does not yet extend toward the median raphe. Radial position within the section was normalized to maximal radius, i.e. length of the lamella containing the respective labeled cell. In other words, radial distance was measured from the apex of the lamellar ‘curve’, i.e. closest to the median raphe, to the cell soma center, and normalized to the distance between this apex position (most central) and the border of the epithelial section (most peripheral). **a** The resulting distributions of relative radius (from 0, innermost to 1, outermost) are shown binned (histogram, top row) and unbinned (ECDF, bottom row). The color code for the OlfC genes is the same as in Fig. 3 to facilitate comparisons between different positional parameters. Light grey curves in the ECDF plot for each of the tested OlfC genes represent the distribution for individual olfactory organs. Due to the smaller number of cells, scatter is increased. **b** Overlay of the seven distributions shown individually in panel **a**), both as histogram (left panel) and ECDF (right panel)

The horizontal distance of the labeled cell from the center of the lamella has been shown to be characteristically different for several *or* genes [10]. We quantified this coordinate as relative radius (r _rel_ = r _soma center/length of the lamella_; 0, innermost; 1, outermost, cf. [10], see also Additional file 3 for a graphical representation of the coordinate. For each gene, cells were found over a wide range, with a half width (hw = r _rel 3rd quartile_ - r _rel 1st quartile_) in the range of 0.225 to 0.262 (Fig. 4, Additional file 4). On first glance distributions for the seven *OlfC* genes analysed looked rather similar, with a maximal difference in median radius (which indicates the center of the distribution) between innermost and outermost distribution of 13.2% of the total lamellar length (Fig. 4, Additional file 4). The range for maximal vertical distance between radial distributions of sparsely expressed *OlfC* genes was between 6.2 and 20.2% of all cells (Additional file 4). Indeed the Kolmogorov–Smirnov test showed few significant differences for the radial distributions (Additional file 4). However, those differences found resulted in two further subdivisions of the three expression zones identified in analysis of laminar height: radial patterns for *OlfCj1* and *OlfCt1* are significantly different, and the radial pattern for *OlfCu1* could be distinguished from *OlfCq1* (Additional file 4). *omp* and *trpc2* distributions are rather similar for the radial coordinate (Additional file 4), thus no distinction between ciliated and microvillous neurons exists for this coordinate.

### Two further subdivisions of OlfC spatial distributions become apparent in the analysis of height within the olfactory organ

**Fig. 5:**
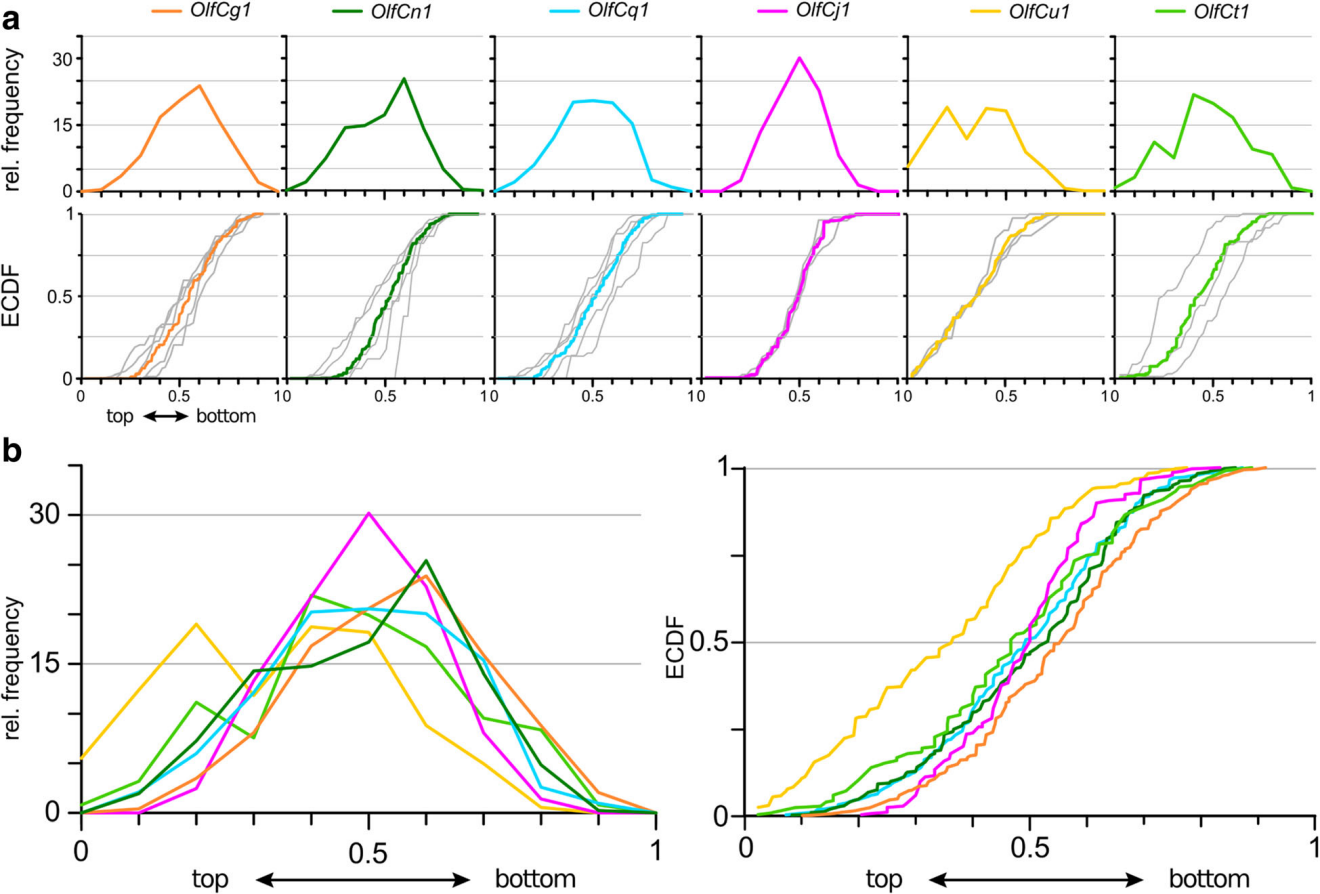
Quantitative assessment of distribution of v2r-related OlfC expressing neurons along the vertical z-axis (height within the organ). Height within the olfactory organ was quantified as section number in a series of horizontal sections, and normalized to the total number of sections containing sensory epithelium, using the same set of cells, for which laminar height was determined. Relative height within the organ ranges from 0 (top section, near to the opening of the bowl-shaped olfactory organ) to 1 (bottommost section). **a** The resulting distributions are shown binned (histogram, top row) and unbinned (ECDF, bottom row). The color code for the OlfC genes is the same as in Figs. 3 and 4 to facilitate comparisons between different positional parameters. Light grey curves in the ECDF plot for each of the tested OlfC genes represent the distribution for individual olfactory organs. Due to the smaller number of cells, scatter is increased. **b** Overlay of the distributions shown individually in panel **a**), both as histogram (left panel) and ECDF (right panel)

As third dimension we analysed the position of *OlfC*-expressing cells with respect to height within the organ (z coordinate, see Additional file 3 for a graphical representation). This parameter was quantified as (horizontal) section number and normalized to total section number of the whole olfactory organ. Z distributions for all but one OlfC receptor are rather similar, and are centered within the middle region of the z axis, with median values between 0.44 and 0.56, and maximal vertical distance between distributions in the range of 5.6 to 22.3% of all cells (Additional file 4). The exception is *OlfCu1*, which is found closer to the top, i.e. closer to the opening of the cup-shaped olfactory epithelium (Fig. 5, Additional file 4). Indeed the distribution for *OlfCu1* is significantly different from that of *OlfCj1*, *n1*, *q1*, and thus the fuzzy border between the two major domains distinguishable in analysis of laminar height can be resolved into an additional expression domain for *OlfCu1*.

**Fig. 6:**
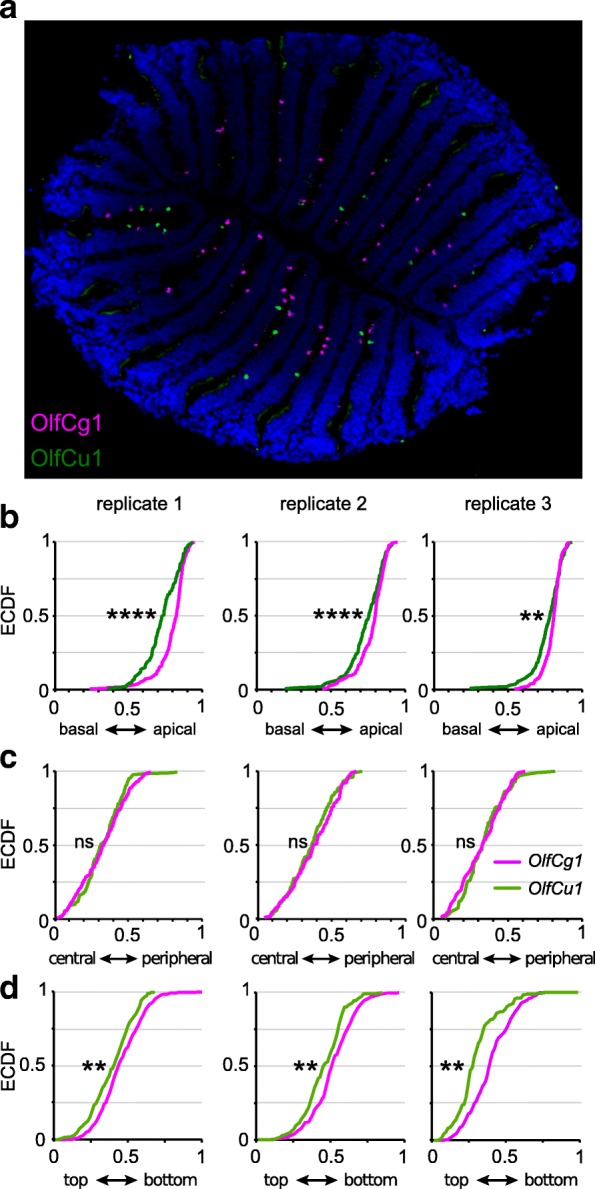
Simultaneous labeling of two OlfC genes confirms distinctly different distributions. **a** Representative micrograph of two-color in situ hybridization, depicting sparse expression of *OlfCg1* (in magenta) and OlfCu1 (in green), within a single horizontal section of the olfactory epithelium. **b-d** Quantitative assessment of the distributions of the labeled cells for laminar height (**b**), radial distance (**c**) and along the vertical z-axis (**d**). The resulting distributions of relative laminar height (from 0, most basal to 1, most apical, i.e. bordering to the lumen), relative radius (from 0, innermost to 1, outermost) and relative height within the organ (from 0, top section to 1, bottommost section) are shown unbinned as empirical cumulative distribution function (ECDF). Color of ECDF graphs corresponds to the color employed in panel **a**. KS-test with a *p*-value cutoff of < 0.01 was used to evaluate the significance of differences between the distributions, if any. Significance is indicated by asterisks, **, *p* ≤ 0.01; ****, *p* ≤ 0.0001

When combining the results for all three coordinates analysed, in total five significantly different expression zones can be distinguished for six *OlfC* genes analysed. Preferred positions in the radial coordinate do not covary with preferred laminar height (Figs. 1 and 6), i.e. radius and laminar height appear to be specified independently. Furthermore, no obvious correlation between preferred radius or laminar height and position of the corresponding gene in the phylogenetic tree is visible (Fig. 1). We note that we use the term ‘preferred position’ strictly to indicate the location of the corresponding distribution, not to imply potential causative mechanisms.

### Double-labeling experiment confirms distinctly different, if broadly overlapping distributions

Due to the broad overlap in the observed distributions we wished to investigate, whether the differences found to be significant in single gene analyses would also hold up, when two genes were analysed by double-labeling in the same olfactory organ. For this analysis we chose *OlfCg1* and *OlfCu1*, which differ in laminar height (g1 is more apical), height within organ (g1 is closer to the bottom), but not in the radial coordinate (Figs. 3, 4 and 5). We performed fluorescent in situ hybridization in complete series of sections for three different epithelia, and determined laminar height, radial parameter, and height within the organ as described in the preceding paragraphs. We report that for each olfactory organ the differences between *OlfCg1* and *OlfCu1* distributions determined in single labeling experiments are faithfully reproduced in the double-labeling experiment. For laminar height the *OlfCg1* distribution was always more apical than that of *OlfCu1*; for radius both distributions were always extremely similar; and for height within the organ (z axis) *OlfCg1* was closer to the bottom than *OlfCu1* in all three cases (Fig. 6).

Thus the differences in expression domains found in the single labeling experiments for *OlfCg1* and *OlfCu1* are validated by the double-labeling experiment. Despite it being practically impossible to test all possible combinations of seven genes in such double-labeling experiments the close concordance of both measuring methods in the chosen example suggests the single labeling experiments in general to deliver robust results.

### The distribution of OlfCc1-expressing cells is not identical to that of trpc2-expressing cells

**Fig. 7:**
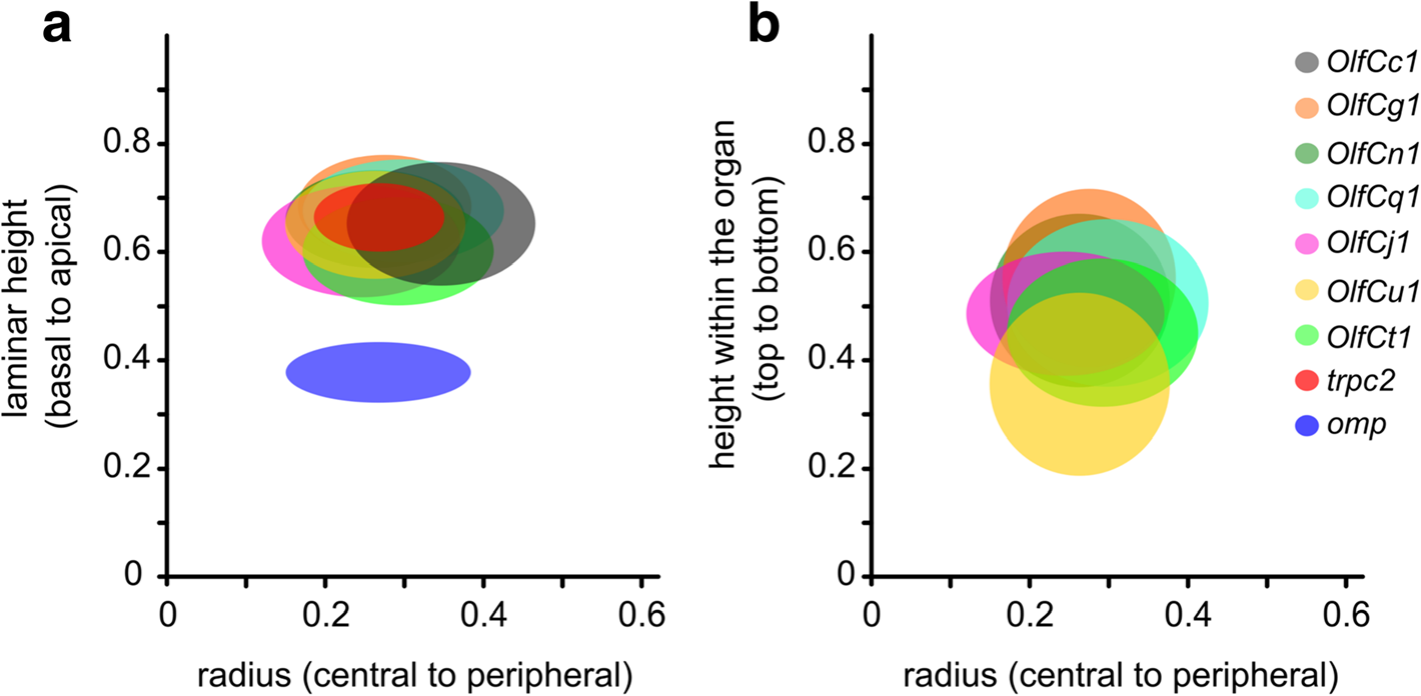
Comparison of spatial distribution parameters between *OlfC* genes. Schematic representation of spatial distributions for different *OlfC* genes and two marker genes, *omp* and *trpc2*, by ellipses ranging from the 1st to the 3rd quartile value for x and y parameter. Color code for *OlfC* genes as before. **a** Radius (x axis) is depicted vs. laminar height (y axis). Note the distributions for all *OlfC* genes and *trpc2*, the marker for microvillous neurons, center on rather apical positions within the lamina (large values for laminar height), clearly segregated from the much more basal positions for omp-positive, ciliated neurons. No correlation is apparent between radius and laminar height values. **b** Radius (x axis) is depicted vs. height within the organ (y axis). No correlation is apparent between radius and height within organ values

*OlfCc1*, the ancestral gene of the main *OlfC* group, is expressed broadly in microvillous neurons, suggesting a co-receptor function for this gene [18]. We have quantified the distribution of *OlfCc1*-expressing cells for two coordinates, radius and laminar height. For laminar height the distribution is very similar to that of the microvillous neuron marker *trpc2* (Additional file 4), and lies central within the distributions observed for the six sparsely expressed *OlfC* genes analysed here (Fig. 7). However, for the radial coordinate *OlfCc1*-expressing cells show the largest preferred radius of all genes analysed, and their distribution is significantly different from that of *trpc2* (*p* < 0.001). It has been reported that a subset of *OlfCc1*-positive cells do not co-localize with *trpc2* expression [18], and our data are consistent with this observation. Differences in onset of expression of these two genes during maturation of microvillous neurons could conceivably play a role, alternatively *OlfCc1* might be expressed in potential microvillous neurons negative for *trpc2* expression.

### Distributions of OlfC-expressing cells are similarly broad as those of OR-expressing cells, but more narrowly clustered

To compare the position and shape of *OlfC* expression zones with those of OR-expressing cells we have re-analysed the raw data from [10]. The width of *OlfC* and OR expression zones, estimated as half-width, was found to be similar for individual *OlfC* and OR genes, both for radial distance and height within the organ (Additional file 4, and data not shown). The median value for height within the organ of *OlfC* and OR expression zones was rather similar for all but one gene (Fig. 7, Additional file 4, and data not shown). However, median values for the radial coordinate of ORs were more divergent, and covered more than double the range than what we observed here for the *OlfC* genes, even though more *OlfC* genes were examined (6 *OlfCs* vs 4 ORs). Notably, the *OlfC* distributions are located between inner and intermediate OR radial distributions, whereas no OlfC receptors with distributions close to the outer OR radial distributions were found. However, we cannot exclude the potential presence of such distributions for other, untested OlfC receptors.

Our results show that (partial) spatial segregation of olfactory receptor gene expression extends to zebrafish OlfC receptors and thus constitutes a general feature shared by teleost OlfC and OR receptor families.

### Segregation of teleost expression zones similar to that observed in tetrapods

Recently some *OlfC*-related *v2r* genes of an amphibian were found to be expressed in the main olfactory epithelium, i.e. sharing a common sensory surface with, among others, ORs [25]. This corresponds to the situation of fish OlfC receptors, which are intermingled with ORs in the single sensory surface. We were therefore interested to evaluate the degree of similarity in the spatial representation strategies of these two species. To enable comparison with results reported here, we have re-analysed the raw data of [25]. Indeed the extent of segregation of ciliated and *OlfC*/V2R-expressing neurons with respect to laminar height is very similar in the clawed frog and zebrafish although interestingly the order is inverse: zebrafish *OlfC*-expressing cells are located apical to ciliated cells, whereas V2R-expressing cells in the clawed frog lie basal to ciliated neurons ([25], Additional file 5). Furthermore, different amphibian *v2r* genes exhibit broadly overlapping, but significantly different expression domains: The two sparsely expressed *v2r* genes in the amphibian olfactory organ show a significantly more basal distribution than the broadly expressed V2R-C (*p* < 0.003), the ortholog of the potential co-receptor *OlfCc1* (Additional file 5). This amounts to the same type of laminar sub-segregation within the amphibian V2R domain, as shown here for the zebrafish *OlfC* family.

Furthermore, two initial analyses showed pronounced segregation according to laminar height in the rodent olfactory system, both for *v2r* gene expression in the vomeronasal organ (seven rat *v2r* genes, [15]) and for *or* gene expression in the main olfactory epithelium (four rat *or* genes, [26]).

A thorough investigation of OR expression patterns in mice [11] has suggested that different ORs have slightly, but distinctly different preferred positions in the unrolled olfactory sensory surface, measured as zonal index along the dorsomedial/ventrolateral axis of the olfactory epithelium. This coordinate corresponds to a radial coordinate in the coronal cross section, the usually chosen representation [11].

Taken together, although some quantitative differences exist between olfactory receptor gene families and species, the basic feature of gradually changing preferred positions for different genes from the same family appears to be conserved across a large swath of vertebrate evolution and several different olfactory receptor families. In all cases large overlap occurs between neighboring distributions.

## Discussion

Spatial segregation of olfactory receptor gene expression within a family appears to be a conserved feature in the tetrapod lineage [8, 9, 11, 15, 25, 27]. In teleost fish a single study has shown a corresponding organisation for ORs [10]. Fish possess only one olfactory organ, in which all their olfactory receptor families are expressed. We were interested to find out, how the expression of another major olfactory receptor family, the V2R-related *OlfC*s is integrated into the spatial pattern of OR-expressing neurons. We have examined six different sparsely expressed zebrafish *OlfC* genes, chosen as representative by their position in the phylogenetic tree, as well as the broadly expressed [17] gene *OlfCc1,* which lies ancestral to the main group in the phylogenetic tree. Earlier expression studies had not analysed spatial positions of *OlfC-*expressing cells beyond noting a sparse expression pattern [17]. We have performed a thorough quantitative analysis in three dimensions, radial distance (central to peripheral), laminar height (basal to apical) and along the vertical z-axis of the olfactory organ (top to bottom), to establish the spatial pattern of *OlfC* gene expression.

We show here that several distinctly different, if broadly overlapping expression zones can be distinguished for zebrafish OlfC receptors. Preferred position appears to be independently specified for each coordinate analysed, similar to results obtained for the amphibian main olfactory epithelium [25]. Furthermore we have re-analysed the raw data from two earlier publications covering frog V2R and zebrafish OR expression. These re-analyses show highly similar segregation into several distinct, and always broadly overlapping expression zones.

The spread of distributions for zebrafish *OlfC* genes appears to be somewhat more narrow than those observed for both *OlfC*-related *v2r* genes [15] and *or* genes [10, 11, 26]. Although we cannot exclude this to be a consequence of our selection of *OlfC* genes analysed, we consider it unlikely due to our sampling a large swath of the phylogenetic tree of OlfC receptors. For the *or* genes one could argue that their larger evolutionary age [5] would have allowed a correspondingly larger diversification in position-specifying mechanisms. However the apparently larger spread of tetrapod V2R expression zones would need to be explained differently. Dedicated experiments to quantify the tetrapod *v2r* distributions will be required to unambiguously solve this issue.

Both in fish (our results, [10]), and in mammals [11] large overlaps are observed between all neighboring distributions, suggesting the (partial) segregation of olfactory receptor gene expression not to be relevant for segregation of function. Alternatively the differences in center of gravity between distributions might constitute a ‘trace record’ of ontogenetic processes. For some zebrafish odorant receptor genes different onsets of expression have been reported, from 24 h to 3 dpf, see e.g. [28]. However, considering the complex morphological remodelling of the sensory surface in the following weeks (formation of median raphe and of lamella) and the huge growth in absolute size of the olfactory organ from larval stage to adulthood, it appears very unlikely that any potential initial asymmetries would have propagated to the adult stage.

One could speculate that age differences in the individual epithelia examined would represent ‘snapshots’ of different migration or maturation stages of sensory neurons or their precursors resulting in differently positioned expression domains. However, this is highly unlikely for several reasons: Firstly, we examined a narrow age range, between 8 and 10 months of age. Secondly, we also find the distribution differences in double-labeling experiments, i.e. within single olfactory organs. Lastly, any potential mechanism has to take into account the constant renewal of olfactory sensory neurons during adult life. The average lifetime of zebrafish OSN has been estimated as around one month [29], so at the age examined, several rounds of neurogenesis have already occurred blurring any potential relation to organismal age.

It is noteworthy that the zebrafish olfactory organ possesses two proliferative zones, one at the apex of the lamella, the other at the outer border of the sensory region [30] . A recent publication [29] examining three zebrafish odorant receptor genes has suggested that different olfactory progenitor cells might migrate differentially from these proliferative zones, which could result in a pattern of broad expression zones with different *centers-of-gravity*. While beyond the scope of the current investigation, it will be interesting to see whether *OlfC* genes follow a similar pattern. As an aside, at least for the mammalian system tangential migration appears to be less relevant, cf. [31]. Alternatively, the molecular mechanisms underlying olfactory receptor gene choice might rely on gradients of signalling molecules, which are read out differently in different precursor cells.

## Conclusions

The basic feature of gradually changing preferred positions for different genes from the same family appears to be conserved across a large swath of vertebrate evolution and several different olfactory receptor families, some quantitative differences between olfactory receptor gene families and species notwithstanding. A hallmark of this organisational principle is the large overlap even of distinctly different distributions, suggesting that these expression domains may constitute a byproduct of ontogenetic processes rather than being relevant for segregation of function.

## Methods

### Sequence data mining and phylogenetic analysis

TblastN searches were performed on the GRCz10 assembly of the zebrafish genome, using representative zebrafish OlfC and mouse V2R sequences as queries. Candidate sequences were aligned by MAFFT algorithm (E-INS-i setting, [32] and sequence positions with over 90% gaps were removed using Gapstreeze (http://hcv.lanl.gov/content/sequence/GAPSTREEZE/gap.html). A phylogenetic tree was constructed using a modified Maximum Likelihood method (PhyML-aLRT) with SPR setting for tree optimization and chi square-based aLRT for branch support [33]. Trees were drawn using Treedyn [34]. Zebrafish and mouse T1Rs served as outgroup. Candidate sequences had to fulfill three criteria to be accepted as bona fide unique *OlfC*s: the gene had to be located inside the corresponding phylogenetic tree with branch support over 80%; the sequence had to map to a unique, non-overlapping genomic position; the amino acid sequence had to show at least 2% difference to other OlfC receptors, and the variable amino acids had to be distributed along the sequence. Sequences that are > 98% identical in amino acid sequence are considered allelic variants [14].

Sequences were named according to the *OlfC* nomenclature introduced by [23] since this study previously had named most *OlfC* genes; one novel sequence (*OlfCe2*) was named according to phylogenetic relationship. *OlfCb1p*, *OlfCe1p*, and *OlfCq10p* were assumed to be pseudo genes [23], but have intact full length ORFs in GRCz10 as predicted by Genewise [35], and have been renamed as *OlfCb1*, *OlfCe1*, and *OlfCq10*, respectively. Two fragment predictions (*OlfCf1* and *OlfCm2*) were extended, *OlfCm2* to full length using Genewise [35].

### Animal handling and probe generation

Zebrafish used in this study are of Ab/Tü genetic background and were raised in the local fish facility. Adult wild type zebrafish (8–10 months old) were anesthetized with MS-222 (ethyl 3-aminobenzoate, Sigma) and decapitated. Olfactory epithelia were dissected out, embedded in TissueTek O.C.T. compound (Tissue-Tek; Sakura Finetek USA), and frozen at − 20 °C. Ten micrometer-thick horizontal cryosections were thaw mounted onto Superfrost Plus slide glasses (Fisher Scientific, Pittsburgh, PA).

Digoxigenin (DIG)-labeled probes for seven V2R-related *OlfC* genes were generated as described [36]. Templates for probes were amplified either from genomic DNA or in some cases from cloned cDNA fragments, with T3 promoter site (TAT TAACCCTCACTAAAGGGAA) attached to the 5′ end of the primers. In situ probes were generated for the following genes: *OlfCn1, OlfCq1, OlfCg1, OlfCu1, OlfCj1, OlfCt1*, *OlfCc1, omp* and *trpc2*, using the following primer pairs: *OlfCn1* Fwd: 5´ GACTTGGATTGGAGCTTTGC 3′; Rev.: 5´ TTGCAGATGGCTCACAGTTC 3′; *OlfCq1* Fwd: 5´ GAGATCCAGGGACTTCGTGA 3′; Rev.: 5´ CCAGGGCATAAACTGCCTTA; *OlfCg1* Fwd: 5´ AGTCAAGCACTTTGGCTGGT 3′; Rev.: 5’CCTCCCAGCACATGAAAACT 3′; *OlfCu1 Fwd: 5´ GCTCCTGGTTGAAGTTGCTC 3′; OlfCu1* Rev.*: 5´ ACA GGC TCTCCATTGGTG TC 3′; OlfCj1 Fwd: 5′ TGAGGGTTG GATCACGTACA 3′; Rev.: 5´ ATGCGTCATACAAGCCAATG 3′; OlfCt1 Fwd: 5´ GCAGCA ATTCTCTCCACTCC 3′; Rev.: 5´* TCTTGTTTTGCCACTGAGCTG *3′; OlfCc1 Fwd: 5´* GGGCCTTTTGAGAACGACACATG 3′; *Rev.: 5´* CAGATTTGCCCATTAGCGAAGAGAG *3′.* The primer sets used for *trpc2* and *omp* are described in [37].

### In situ hybridization

Ten micrometer-thick horizontal cryosections were thaw-mounted onto Superfrost Plus slide glasses (Thermo). Pretreatment of sections, probe hybridization, and stringent washing were performed as described [10], except omitting Proteinase K digestion. After stringent washing at 65 °C, sections were blocked in 1% blocking reagent (Roche) in PBS for 1 h. The slides were then incubated at 37 °C for 2 h with sheep anti-DIG Fab fragments conjugated with alkaline phosphatase (Roche), dilution 1:500 in blocking solution. After washing 3 times in PBS, hybridized probes were visualized by enzymatic reaction with NBT-BCIP (Roche). After evaluating the success of the staining, slides were washed 2 times in PBS for 5 mins each, mounted with VectaMount (Vector Laboratories, Burlingame, CA, *USA*) and photographed with a wide field microscope (Keyence BZ-9000). In case of the two-color double in situ hybridization, probes for OlfCg1 and OlfCu1 were labeled with dig and flu antigen respectively and similar in situ conditions were applied as described above except the detection steps. OlfCg1 probe was detected with anti-dig conjugated with alkaline phosphate, followed by HNPP detection method (Sigma Aldrich) whereas the OlfCu1 probe was detected by peroxidase labeled anti-flu antibody, followed by treatment with biotin-tyramide. Biotin was detected with Alexa-488 conjugated streptavidin.

### Measurement and analysis of spatial coordinates

The distribution of receptor neurons labeled with a DIG-labeled probe was assessed in complete series of sections of olfactory epithelium. Three spatial coordinates were evaluated: radial distance (center of the lamella to cell position), height within the lamella (basal border of the lamella to cell position; laminar height), and height within the organ (Number of horizontal section from top to bottom; z axis). A graphical description of the measurements is given in Additional file 3. No differences in frequency were observed between left and right side of the center line (the median raphe). Spatial coordinates were measured in arbitrary units and normalized as described [20]. For example, apical-to-basal position within an lamella (laminar height) was measured as the shortest distance between center of the cell and basal border of the epithelial layer, and normalized to the thickness of the epithelial layer at the position of the cell [20]. Thus the range of values is between 0 (most basal) and 1 (most apical). Unbinned distributions were represented as the corresponding empirical cumulative distribution function (ECDF) [38, 39]. In this presentation, data points are sorted by their parameter value (x axis), with their ordinal number (normalized) as y axis. Each data point results in a curve point, thus no information about the distribution is lost in the representation as ECDF, in contrast to the usual histogram representation. To estimate, whether two spatial distributions were significantly different, we have performed Kolmogorov-Smirnov tests on the unbinned distributions using R [40] with the following command: laply(inputfile.csv, function(x) llply(inputfile.csv, function(y) ks.test(x, y)$p.value)). The Kolmogorov-Smirnov test makes no assumptions about the nature of the distributions investigated, which is essential since the skewness of many distributions showed that these are not Gaussian. Due to the sensitive nature of the test on large distributions (*n* > 100) we selected *p* < 0.01 as cutoff criterion for significant difference, cf. [25].

## Additional files

Additional file 1: List of all zebrafish OlfC genes. Gene names, synonyms, accession numbers, genomic localisation in the GRC z10 genome, amino acid and nucleotide sequence length are listed. (XLS 19 kb)

Additional file 2: The treefile for the tree shown in Fig. 1 is given in Newick format. The predicted protein sequences for all OlfC genes identified are listed, and differences to the most complete previously published OlfC repertoire are indicated (yellow overlay, blue text color) and described. All sequences used as outgroup for the phylogenetic tree are listed as well. The nucleotide sequences for all OlfC genes (coding region only) identified are given. (DOCX 118 kb)

Additional file 3: Quantitative in situ hybridisation of OlfC-expressing cells. (A-C) Comparison of OlfCc1 expression with that of olfactory neuron marker genes OMP and TRPC2. Labeled cells were exclusively detected in the sensory region of the adult olfactory epithelium. OlfCc1 distribution is similarly apical as TRPC2 and more apical than OMP, the marker for ciliated neurons. (D) Expression frequency for six different OlfC genes. The bar graphs represent the number of cells observed for a particular *OlfC* gene in the complete olfactory organ comprising 40–60 sections (mean +/− SEM, *n* = 3–5 olfactory organs). (PDF 12927 kb)

Additional file 4: Distribution properties and significance of differences. A) The first sheet of the spreadsheet contains the parameter values for the first, second and third quartile of the distributions, i.e. for the radial, laminar height and z-axis coordinates. Half width of distributions was determined as 3rd quartile-1st quartile difference. Distributions of cells expressing V2R-related *OlfC* and marker genes in adult zebrafish were determined in this study. Values for zebrafish OR and Xenopus V2R were determined from the raw data of the respective publications. B) Maximal vertical distance between two distributions. Pairwise comparison of spatial distributions for different olfactory receptor genes and marker genes of the adult zebrafish (OlfC), and larval *Xenopus laevis* (V2Rs) to determine the maximal vertical distance between the respective cumulative distribution functions. The range for this maximal vertical distance is pointed out by indicating the minimal and maximal values found. C) The Kolmogorov Smirnov test (see Materials and Methods) was used to determine significance of distribution differences. As a cutoff for significance, we chose *p* < 0.01 due to the sensitive nature of this test for large distributions (> 100 data points). (XLS 51 kb)

Additional file 5: Interspecies comparison of spatial expression patterns. Interspecies comparison of spatial distributions of several OR and V2R genes for adult zebrafish, larval *Xenopus laevis*, adult mouse and adult rat. The respective species is represented graphically, olfactory organ and the olfactory receptor gene family examined are noted below. The respective genes are indicated by color code, which is unique within each panel; gene names are as given in the respective publications. Spatial distributions are represented as ECDF (y axis). For the radial distribution, the x-axis represents the normalized radial distance with a scale ranging from 0 (central) to 1 (peripheral); for the height distribution the x-axis represents the normalized laminar height with a scale ranging from 0 (basal) to 1 (apical). For Xenopus V2R [25, 41] and zebrafish OR [10] the raw data of the respective publications were used to generate the graphs. Original gene names used in [10] were fZOR6 (or112–1), fZOR9 (or107–1), fZOR8 (or103–1), and fZOR5 (or102–1). Note the structural similarities between different species, olfactory organs, and olfactory receptor families. (PDF 557 kb)

## Abbreviations

*OlfC*: ORs belonging to class C GPCRs
ORA: Olfactory receptor class A-related
ORs: Olfactory receptors
TAAR: Trace amine-associated receptor
V2R: Vomeronasal receptor type 2)
