# Supplementary material for: Overlapping but distinct topology for zebrafish V2R-like olfactory receptors reminiscent of odorant receptor spatial expression zones
Source: BMC Genomics. 2018 May 23;19:383. doi: 10.1186/s12864-018-4740-8 (PMC5966872; doi:10.1186/s12864-018-4740-8)
Supplement: Supplementary file 2 — The treefile for the tree shown in Fig. 1 is given in Newick format. The predicted protein sequences for all OlfC genes identified are listed, and differences to the most complete previously published OlfC repertoire are indicated (yellow overlay, blue text color) and described. All sequences used as outgroup for the phylogenetic tree are listed as well. The nucleotide sequences for all OlfC genes (coding region only) identified are given. (DOCX 118 kb) [file 12864_2018_4740_MOESM2_ESM.docx]

## Supplementary Text

Ahuja et al., Overlapping but distinct topology for zebrafish V2R-like olfactory receptors reminiscent of odorant receptor spatial expression zones

## Table of Contents

Treefile in Newick format for tree shown in Fig.1 ……………………………………….… 2

Predicted protein sequences of the zebrafish V2R-related OlfC family ………………… 3

Sequences used as outgroup for the phylogenetic tree ………………………………… 24

Nucleotide sequences of the zebrafish V2R-related OlfC family ………………………. 27

References …………………………..………………………………………………………. 75

## Treefile in Newick format for tree shown in Fig.1

The phylogenetic tree was built using all confirmed candidate genes (except a short pseudogene fragment) and zebrafish and mouse T1Rs as outgroup. A modified Maximum Likelihood method (PhyML-aLRT) was used, with SPR setting for tree optimization and chi square-based aLRT for branch support [1]. The treefile is given in Newick format, and includes branch support as fraction of 1.

(Dr_T1Rb2:0.19587086,Dr_T1Rb1:0.25113994,((Mm_T1R2_GI:1.31263305,(Dr_T1Ra1:0.94132448,Mm_T1R1_GI:0.87431581)0.999850:0.20349820)0.932806:0.09553676,((Dr_T1Rc1:1.15853598,Mm_T1R3_GI:1.17725037)0.999850:0.45265724,((OlfCa1:0.99477778,OlfCb1.sik:1.43080942)0.995159:0.20174443,(Dr_CaSR:0.67221430,(OlfCc1:0.91212450,((OlfCu1:0.68481329,(OlfCr1:0.76644400,(OlfCq12:0.11938819,(OlfCq1:0.15357666,((OlfCq13:0.14904395,(OlfCq21:0.11241197,(((OlfCq18:0.13581678,OlfCq20:0.08931615)0.999850:0.02059973,(OlfCq16:0.09629501,OlfCq17:0.11062921)0.963067:0.00764626)0.999850:0.01411949,(OlfCq14:0.16996162,(OlfCq19:0.12873503,(OlfCq8:0.03052561,OlfCq6:0.02430649)0.999850:0.12927360)0.987240:0.01088886)0.942133:0.00426634)0.999850:0.01313233)0.999850:0.01784780)0.999850:0.02114506,((OlfCq11:0.15489338,((OlfCq5:0.00384642,OlfCq4:0.01110499)0.999850:0.04518046,(OlfCq10p.s:0.02179681,OlfCq9:0.02777389)0.999850:0.01549082)0.999850:0.11142639)0.999850:0.05390355,(OlfCq2:0.17284848,OlfCq3:0.13572726)0.999850:0.02843067)0.998812:0.01410617)0.786177:0.02815587)0.876818:0.02297939)0.999850:0.45618108)0.990464:0.10951987)0.969576:0.04928073,((OlfCw1:0.65597255,(OlfCt1:0.55004221,(OlfCs2:0.09592561,OlfCs1:0.11851860)0.999850:0.47097424)0.857384:0.05082577)0.999850:0.06321961,(((OlfCx1:0.07408549,(OlfCx3:0.06863551,OlfCx2:0.05701121)0.979481:0.03670629)0.999850:0.71083539,(OlfCv3:0.20434298,(OlfCv2:0.05426670,OlfCv1:0.05639767)0.999850:0.15423393)0.999850:0.41816437)0.878053:0.05431199,(OlfCn1:0.65272470,((OlfCf1.sik:0.51372182,((OlfCk2:0.07144703,(OlfCk1:0.07877027,OlfCk3:0.08444525)0.996580:0.03107927)0.999850:0.47883307,((OlfCe1.sik:0.01311779,OlfCe2.sik:0.05187243)0.999850:0.33147256,(OlfCj1:0.48038670,(OlfCm2.sik:0.10223156,OlfCm1:0.10334766)0.999850:0.36369743)0.443348:0.03809246)0.999850:0.05154205)0.842054:0.01782868)0.928844:0.02216500,((OlfCd3:0.09751116,(OlfCd1:0.12869249,OlfCd2:0.14426663)0.911178:0.02307256)0.999850:0.31394099,(OlfCh1:0.43746312,(OlfCg8:0.14783970,((OlfCg11:0.12861967,OlfCg12:0.17311207)0.998387:0.02269229,((OlfCg10:0.14667625,OlfCg5:0.13897205)0.484231:0.00929247,(OlfCg1:0.13408050,(((OlfCg4:0.15886176,OlfCg3:0.16202067)0.997156:0.02094192,(OlfCg6:0.15164446,OlfCg2:0.15442296)0.999850:0.02249831)0.999850:0.01653369,(OlfCg7:0.13456806,OlfCg9:0.16192008)0.994329:0.02963995)0.997384:0.01269995)0.999850:0.02156547)0.993968:0.01458663)0.998057:0.04993112)0.999850:0.31482187)0.976677:0.04776555)0.992621:0.03466171)0.999850:0.21007716)0.995597:0.07551510)0.999850:0.05532505)0.658208:0.03658275)0.999850:0.18146259)0.999850:0.26733075)0.999850:0.40938789)0.999850:0.46303970)0.999850:0.23740866)0.999850:1.11461822);

## Predicted protein sequences of the zebrafish V2R-related OlfC family

The list contains the current amino acid sequences of the OlfC family in zebrafish, in total 60 intact genes, and one pseudogene (OlfCq15, a short fragment). The repertoire was established by extended tblastn searches in the zebrafish whole genome sequence, release Z10, as provided by NCBI, followed by Genewise2 gene prediction and in some cases manual curation. Differences to the previously most complete description (56 intact genes, 6 pseudogenes) by [2] are indicated by yellow overlay of the sequence header, the respective sequence region is additionally indicated in bold blue text. In short, one sequence was newly found (OlfCe2), three sequences formerly listed as pseudo genes are now present as intact, full length sequences (OlfCb1, OlfCe1, and OlfCq10), and one sequence formerly described as truncated version is now present as full length (OlfCm2). N-terminal extensions of the ORF in the range of 4 to 46 amino acids were observed for 15 OlfC genes (a1, d2, f1, g1, g7-11, k1, q2, q11-12, v3, and x1). Unexpectedly, OlfCt1 is lost from the current genome database, although it is present in the genome (from which we cloned it, see Materials and Methods). Two short pseudogenes described by [3] and [2] could not be localized in the version of the genome (OlfCy1p and OlfCq7p, respectively).

>OlfCa1

**MMCKCTPVSRSGELRE**MDLMSFILLWAGLMKVAEASIAQFSQLGASAPGNIIIGGLFPIHEAVVPVNYTGNNSISAPEHPDCIRFYTKGLNQALAMINAVEMANKSPMLSSLNITLGYRIYDTCSDVTTALRAVHDIMRPFSDCESPEDSSQPVQPIMAVIGTTSSEISIAVARDLNLQMIPQISYASTATILSDKSRFPAFMRTVPSDEYQTCAMAKLLKSNKWSWVGIIITDGDYGRSALEGFIQHTETEGICIAFKAILPDSLADQQKLNTDIENTLNIIENNPKVRVVISFAKSSQMQLLFKGLQSRNISNNMVWVASDNWSTAKHILNDGSITDIGKVLGFTFKSGNFTSFHQYLKNLQFESEDEMNNSFLKEFLKLNAGNASNTVLELMKSTNLDKIFSIEMAVTAVANAVAKLCAERQCQDSTALQPWELLRQLRSITFENGGKMYKFDANGDINLGYDLFLWEGDQSDEHADDIIAEYDPTKGGSLYIHNDLSEIEKVVSRCSNSCQPGQYKKTAEGQHTCCYECLTCVENHYSNITDADECSPCDSESMWSLANSTECHPKVFEYFDWNSGFAIVLLILAALGVLLLFFMSALFFWQRHSPVVKAAGGPLCHLILVSLLGSFISVVFFVGEPSDLTCRARQVIFGFSFTLCVSCILVKSLKILLAFEMNFELKELLCMLYKPYMIVSVGMGVQIIICTVWLTLYKPFKDKEVQTDSILLECNEGFYVMFWLMLGYIALLALFCFTFAYIGRKLPQKYNEAKFITFSMVICLMAWIIFIPIHVTTSGKYVPAVEMVVILISNYGILSCHFLPKSYIILFKKEHNTKDAFMKNVYEYARKSAENIKGLTGTEPQFKQENSVYTISNLSFVPEEKHE

>OlfCb1

MKAAGQLCVLGLMMLSWVSWLRCDPVDSMCGAYLNGDVNIAILSSIHSKVRNLHQRTRPQ

PFICSDFDLMTFVQSLGAIHTVEEINNSNFLPGIKLGYKICDPCASPTKALHCLEHLLAI

NGSLPALLDYSDFCPPSKAIMGERYSELSIAIAKLLSLYLVPQVSTSSSSPVLSDKLRYP

SFMRVIPSDVYQAQALVKLMSHFSWNWVGVVYGDDDYGRGAYQSFMEESQGKICADFEKV

VPHYLDQVDVDKYIQEAANAIRSSSANVTLLILKPQLVEKLFKEMIKTNTSRVWIASDAW

SMFRPLTQMKDINKVGPIFGFSFSLGNIPGFEDYLRNLRPTPGGKNDFIEEYQQLRLNCS

LWPSNCTTDDVLYAVELREAYRERVAIYAIAHGLRELLKCNDTTCSSETNFPPWQLVASM

RRVNFTLDGNSYFFNENGDFTDGYDVIMWKENNEERMIEPVGKFLIKKGDVEIFSEYHWI

NETLLSSSCSQFCQPGTVKKQSNITCCYKCVPCDVGYYTNASDQS**E**CLKCPDGQSSLKGA

**TQ**C**E**IVKEQYLFWTDGYPITLLAATAIGLLLVLVSSILFCVHRNTMVIKKADGTMSGFML

LGLTASFISVIMFIGRPNEHLCRAQQAVYSLGFTLCVSCILVKAFRTFLAFLVFNPQKQH

ELKKLYKPLIILVLLTGGQAIILLFWLILKSPYPDPLWPGSGLIKYVICNEGSIAGFGAM

HGYIALLAFTCFFLAFKGRKVPQDFNETGVIIFSMLIHLFVWLCFIPIYIDRNRTEQRHI

VQASAILASNYGIMFCHFLPKCYVVLWELSENSRAIILGRLTRRIRDEMTSADIAVVTVS

GIICEEVSAEISPVSKDPALKSVELFHAERGGAEQTVTHRVQTRRRHITK

>OlfCc1

MDLTGLSYEGRFLLVLCMISYLFTPTDAEGSCKLKAKFNLRGYKEVEKTTVVIGGMFPVH

RSLVSTDSNTTDPPESVDCQGFNFRAFRWAQTMLFALKEINSRTDLLPKTELGYVIYDSC

FTISKAVEGTLTFLTGQDEAVPNYRCGNGPPLSALVGAGGSDLSIATARILGLYYFPQVS

YESSCSVLESRFQYPTFLRTIPSDENQSVAMAKLVLRFGWTWVGTIAAEDDYGKYGIKRF

KEVVEEAGVCISFSETLPKISNPEAIQRIVQTVHDSTAKIIVVFSSDVDLSPLVEALLQS

NVTNRTWIASEAWVTSAAISRQPNVLSLLGGTIGFAVKRAEIPGLKKHLLSISPFNDSLT

EEFWGIVFNCTTNYTLILKGMRRCTGEEMLGTVDNTYSDVSQLRITYNVYKAVYAVAHAL

HNLEQCKTGSGPFENDTCADITNFEPWQLMYYLIHLRFTVPHTGEELFFTNGEVEGFYEL

LNWQSDSNGGITYTHIGYYNSTAAPEDKLVINNNSIIWNNNVLKAPRSVCSERCQPGTRM

GIRQGEPVCCFDCIPCADGEISNTTDARGCIQCDGDYWSNANHDECVPKTIEFLDFSEPL

GITLIAIAAFGALATIVVAIIFLMHLNTPLVNVNDPLLTFSLLLGLVITFLCSIVFLGKP

QMWSCMTSQMALAVGFALILSSLMGKSALLMLRARAVKAVKAAAKAAKAAAAASEQSPDT

AVFAPAIPQKNDVDPIHPRHQRAIMILCTLIQVVGCTAWLILMPPHPVKNTGVQNIKIIL

ECDPGNIIFICSIFGYDILLALVTFAFAFVARKLEDHFNEGKSVTFGMLVFFIVWSSFVP

AYLSTRGKFMVAVQIFAILASSFGLLACVFIPKCYVLLVKPERNKEEMMIPRPKSRDIAA

AAASSASLATTSSSGNPNGTTVSTLSLEE

>OlfCd1

MTKKSELLLLMVLVTHGICVPNLAQVCRLLGQPALPLLSEERNINIGAIFSLHKSALLKS

HNFTSKPKQTTCGGLNLREFKFVQTLIFAIEEINNSSLLPGASLGYKIYDSCGSVAQAIF

SGMALMNGYEETLSDTSCSTPPAVHAIVGESNSSPTIGLASVVGPFSLPLVSHFATCSCL

SNRKRFRTFFRTIPSDYYQSRALAQLVKHFGWTWVGTVRSRSDYGNNGIATFEEAAKQEG

ICIEYSEAVFKTDPEVQFLKTIEVIKKATTKVVVAFMAFGDFVLLLKVMAQQNITGIQWI

GSESWITSRNLAETKEYHFLSGAVGFAIANSKPVGLREFLMNVHPDKELNNELLKEFWET

VFLCSFRNSSSGGCTGSEKLSELQNEYTDFSELRIENKVYTAVYAVAHSLHNVLKGFKFF

TNSSKKQLPTPQKVLEYLRDVNFTVNTNENIFFDASGDPVARYDLVNWQPTKDGSLQFKL

VGIYDSSLPSEQRLQINQESMLWAGNSGQLPVSVCSESCPPGTRKAVQKGRPICCYDCIP

CAEGEISNYTDSSDCFPCGLEYWSNESKDRCVLKVIEFLSYTEIMGVGLCIFSFIGVLLT

ATVSFLFYFYKETPIVRANNSELSFLLLFSLTLCFLCSLTFIGRPTDWSCMLRHTAFGIT

FVLCISCVLGKTIVVLMAFRATLPGSNVMKWFGPLQQRLSVVSLTLIQVIICVLWLTMSP

PFPFLNLSYYREKIILECNLGSAFGFWAVLGYTGLLSTLCFVLAFFARKLPDNFNEAKFI

TFSMLIFCAVWLTFIPAYVSAPGKYTVAVQLFAILASSFGLLFCIFAPKCYIILLKPEKN

TKKQMMGKS

>OlfCd2

**MQHYFSFASEDLLFMFTLG**MANTLSLLLLLLLLHGHFLPATVQICDLVGQSALPVLSAER

DINIGAIFSIHRNALLRLYPFTSKPEPTTCVRLNLREFKFAQTFIFAIEEINNNTQLLPG

VTLGYKIYDACNAISPTLVSGMALINGYENTLSDTSCSQPPAVQAIVGESSSSPTTALAA

LVGPFNIPVISHFATCACLSNRKRYPSFFRTIPSDYYQSRALAQLVKHFGWTWVGTVRSR

SDYGNNGITAFEDAAKQEGICIEYSEAILRTDPQEQFRKTVEVIKKGTSKVVVAFISFGD

FAPLVKVIAENNITGLQWVGSESWITSRNLAETKEYSFLSGAVGFAIVNAKLVGLKEFLV

NVHPDQEPKNKLLKEFWETAFQCSFSNSDSALCTGSEKLGELKNEYTDVSELRIEHKAYA

AVYAVAHTLHNVFKDVKAPNNSKRELPTPQKVLQYMRNLSFTIKTGENIFFDASGDPVAR

YDLVNWQTAEDGSLQFKHVGIYDSSLPSKQRLQVNQEIMLWAGKSGLLPLSVCTESCLPG

TRKAVQKGRPVCCYDCIPCAEGEISNSTDSSDCFSCDLEYWSNEKKDRCILKVVEFLSYA

EIMGIILCIVSFIGLLLTVMVTCLFYLHKETPIVRANNSELSFLLLFSLSLCFLCSLTFI

GRPTQWSCMLRHTAFGITFILCISCVLGKTIVVLMAFRATLPGSNVMKWFGPLQQRLSVV

SLTLVQVIVCLLWLTLSPPYPYMNLSYYREKIILECNLGSAFGFWAVLGYTGLLSTLCFV

LAFLARKLPDNFNEAKFITFSMLIFCAVWLTFIPAYVSSPGKYTVAVEIFAILVSSFGLL

FCIFAPKCYIILLKPEKNTKKQMMGKL

>OlfCd3

MAKKIVSLPVLLLIVYGISVSALAQVCRLLSQPALPLLSAERDINIGAIFSIHISALLKM

HSFTFKPESTACISLSLREFKFAQTLIFAIEEINNNTQLLPGVSLGYKIYDSCGSIAQAI

FSGMALMNGYEETLSDTSCSRPPAVNAIVGESNSSPTIALASIAGPFSLPVISHFATCAC

LSNRKRYASFFRTIPSDYYQSRALAQLVKHFGWTWVGTVRSRSDYGNNGIATFEEAAKKE

GICIEYSEAILRTDPQDQFLKTVEVIKKGTARVVLAFVALRDFYPLLKEIAQQNITGLQW

VGSESWITSRTVAETKEYSFLSGAVGFSIANVKLVGLLDFLVNVHPDHEPKNKLLKEFWE

TTFQCSFNNRGSVGCTGSEKLANLQNEYTDASELRIANKVYTAVYAVAHTLHNIFQDFKS

ANRSKLKQPTPQMVLNYMKDVRFTVKTGEEIFFDTSGDPVARYDLVNWQPSGDGSLQFKN

VGIYDSSLPSEKCLQVNQEHVLWADNSRQLPVSVCSESCPLGTRKAVQKGRPVCCFDCIP

CADGEISNNTDSSDCSPCDEEYWSNERKEKCVLKVIEFLSYTEIMGMVLCIFSFIGLLLT

AMVSFLFYLHKETPIVRANNSELSFLLLFSLSLCFLCSLTFIGRPTEWSCMLRHTAFGIT

FVLCISCVLGKTIVVLMAFRATLPGSNVMKWFGPTQQRLSVVFLTLIQVIICVVWLTVSP

PFPYKNLSYYREKIILECNLGSALGFWAVLGYTGLLSVLCFILAFLARKLPDNFNEAKFI

TFSMLIFCAVWLTFIPAYVSSPGKFTVAVEIFAILSSSFGLLFCIFAPKCYIILLKPEKN

TKKQMMGKSSSKTI

>OlfCe1

MVFLHTWLFFTLVRAANPPCKLLGPPNIPQFSKDGDVTIGGIFSFHNSWEEIMPTFTSKP

EQPKCKSLSLREFQNAQTMIYAIEEINKRADILPGLSLGYRIFDSCGSIEMALRASLSLV

NGENASHLSCQRPKTVQAIIAETSSTPTIAISATVGPLHLPVISHFATCACLSDRKKHPS

FFRTVPSDYYQSRALAKLVKYFGWTWVGALCSDNDYGNNGMNTFIKAATEFGVCVEFSEA

FFRTHPREEILRIVDVVKKSSSKVIVAFVSYSDMEVLLLELAKQNITGLQWIGSESWISD

MNIATGKWQYILRGSMGFAIPKAEIQGLREFLIKIHPSSNIYLYKELWESVFQCRLSTEQ

SSESKSMCTGNESLNNVQNQYTDVTELQIANNVYKAVFAIAHALNSSVGCSKWDTNQREC

NTWQ**V**VLQALREVSFFTETGEKVFFDKNGDPAARYDLLNWQQGEEGATKFVKVGFYDASL

QPEFQLSFNNITIMWAK**NQHQ**VPVSVCSESCPMGTRKAVKKGKPLCCYDCIQCAEGEISN

KTDSVMCLKCPPEFWSNKWRDTCVPKLVEFLSFEDVMGIVLIIFSLLGVSFTLGIAIIFF

VHKDTPIVKANNSELSFLLLFSLTLCFLCSLTFIGRPTEWSCMLRHTAFGITFVLCISCV

LGKTIVVLMAFKATLPGSNVMKWFGPLQQKLSVITFTLLQVVICVLWLTLSPPFPYMNMN

YYQERIILECNLGSAFGFWAVLGYIGLLAILCFILAFLARKLPDKFNEAKFITFSMLIFC

AVWITFIPAYVSSPGKFTVAVEIFAILASSFALLFCIFAPKCYIILLRPEENTKKHMMSK

S

>OlfCe2

MFFLHLWLFFTLVRAANTLCKLLGPPNIPQLSKDGDVTIGGIFSIHTSWEEILPTFTSKP

EQPKCKSLSLGEFQNAQTMIYAIEEINKRADIISGQSLGYRIYDSCGSTDMALKASLSLV

NGENASQLYCQRPLTVQAIIAETYSTPTIAISATVGLLHLPVVSHYATCACLSDKKKHPS

FFRTIPSDYYQSRALAKLVQYFGWTWVGALCSDNDYGHNGMNTFIKAATEFGVCVEFSES

FFRTHPREEILRIVNIVKKSSSKVIVAFVSYADMEVLLLELAKQNITGLQWIGSESWISD

MNIATGKWQYILRGSMGFAIPKSEIQGLKEFLTKVNPSSNIYLYKELWESVFQCRLSPEQ

SSESKSMCKGNESLNHVQNQYTDVTELQIPNNVYKAVLAIAHALNSSVGCLKWDKNQREC

LEKINNTWQVVLQALREVSFFTETGEKVFFDKNGDPAARYDLLNWQQGEEGATKFVKVGF

YDASLQPEFQLSFNNITIMWAKVPVSVCSESCPMGTRKAVKKGKPICCYDCIQCAEGEIS

NKTDSVMCLKCPPEFWSNKWRDTCVPKLVEFLSFEDVMGIVLIIFSLLGVSFTLGIAIIF

FVHKDTPIVKANNSELSFLLLFSLTLCFLCSLTFIGRPTEWSCMLRHTAFGITFVLCISC

VLGKTIVVLMAFKATLPGSNVMKWFGPLQQKLSVITFTLLQVVICVLWLTLSPPFPYMNM

NYYQERIILECNLGSAFGFWAVLGYIGLLAILCFILAFLARKLPDNFNEAKFITFSMLIF

CAVWITFIPAYVSSPGKFTVAVEIFAILASSFGSLFCIFAPKCYIILLKPEENTKKHMMS

KS

>OlfCf1

**MIVIITTKKDQGRFSNILGAA**MTLVNNQEQSEPGANCSKPQTVQAIIGHSGSTPTMSFAK

IVGNFHIPVISHFATCACLSNRKEYPSFFRTIPNDFYQSRALAQLVRHFGWTWVGALSNK

NDYGIDGITTFIKSATEEGVCIEYIEAFESTSQYTEILRIVNIIRSSTSKVIMAFMSHRE

IKLLVDELYKQNVTGLQWIGSDAWITDDSLADIYGHTLLTGSIGFAVRNAQIPGLGSFLQ

KVNPSQFPNSIFVKDFWEHVFDCSFTTSKLTKRCNGSENLSDVQNSFTDLTDLRFSNNIY

KAVYAVAHAINNLLLCEENKSSSPNACLKLAEIKPWQLLNSLQSVNFTTPGGESVFFDSN

GDSPARYELINLQSVDRGKIDVETIGYYDASLPKDQRFSMNNVKVVWREGGDQVPVSVCS

TSCPSGTRKAMQKGRPVCCYDCILCPPGEISNKTDSVDCLKCPVTQWSNTRGDACIPKEI

EFLSYDEIMGILLGLFSLIGAFFTIVVTLIFYIYRSTPIVRANNSELSFLLLFSLTLCFL

CSLTFIGRPTEWSCMLRHTAFGITFVLCISCVLGKTLVVLMAFKATLPGSNVMKWFGPLQ

QRLSVVSFTFIQLLICALWLILSPPFPYMNMKHYQEKVILECNVGSATGFWAVLGYIGLL

AVLCFVLAFLGRKLPDNFNEAKFITFSMLIFCAVWITFIPAYISSPGKFIVAVEIFAILV

SSFGLLFCIFLPKCYVILLRPEINTKKHVMAKINK

>OlfCg1

**MRILMQIVYQAIYICQSKFSSIFV**MLLFLYIFLHFTLIRTKAGNGLCQMMGDPKYPLFSK

DGDITIGALFAILSKETLPSFQFTQKPQLLSCSSVNLRDFRMTQIMIFAIEEINRSKSLL

PNVSIGYRIYDTCGSRMSAMSATMALMNGPEFTADKICNGESPIHAIIGETESSATIILS

KTTGPFKIPVISHSASCECLSNRKIYPSFFRTIASDYHQGRALAFIVKHFGWSWVGTVNS

DNDYGNNVMAIFLNTAQKEGICVEYSVKFYRTEPEKLKKVVETIKKSTAKVIVAFVSLVE

MGLLIDQLSIQNITGFQVIGGEGWITTKSLINSKSFHVLGGSLGFAFRKIIIEGFADYVI

KTFWERDFPCLTNEGNYSQYALTCSSYQDLFTLKHYNEDIPEQRYASNVYKAVYAVAHSL

HNLLKCKENEGCEKSLTMQPQQVVEALKNVNFTLKFGDRVWFDSTGGAVAHYEVVNWQQD

SDGSFQFKKVGYFDASLPPDQSFMLNTKNIIWSGGQLERPRSVCSESCPPGTRKASQKGR

PVCCYDCIPCAEGEISNETDSVNCKQCQGEYWSNVEKNKCVLKAVEFLSFTDFMGIVLVF

FSLFGVGLTVLVAILFYSKKDTPIVKANNSELSFLLLLSLTLCFLCSLTFIGRPTEWSCM

LRHTAFGITFVLCISCVLGKTIVVLMAFKATLPGSNVMKWFGPVQQRLSVLAFTLIQVLI

CVLWLTMSSPFPYKNMKYYKEKIILECSLGSVIGFWAVLGYIGLLAALCFILAFLARTLP

DNFNEAKFITFSMLIFCAVWVTFIPAYVSSPGKYTVAVEIFAILASTFGLLFCIFVPKCH

IIIFKPEQNTKQHMMGKNYIHTEK

>OlfCg2

MLFFLHTLPLLYQLNTNTPCQTMGDPKDPLFSKDGDVTIGGVFAIHRKETLPSFEFAQKP

LPLSCSSVNLRDFRLAQIMIFAIEEINKSKSLLPNIYIGYRIYDTCGSRLSTMSATMAVL

SGQEFRPRDRCNDQPPLHAILGESESSATVILSRTTGPFKVPVISPSATCECLSNRKDYP

SFFRTIASDYHQSRALAYIVKHFGWTWVGAVSSDNDYGNNGMAIFQKIAQEEGICVEYSV

KFYRTESDKLKKVVNEIKKGTAKVIVAFVSFVEMGLLIDQLSIQNITGFQLIGVESWITT

KNYFTPNSFSSLRGSLGFAVRKINIEGFIDYVIKAFWDTAFPCTKTEGNSSQYSISCTRS

EDLLELKNYTEDVLEQRYASNVYKAVYAVAHSLHSLLGCENQGLCDKNKPIRPQQMVEAL

QKVNFTIQMGDQVWFDSTGGTVAHYEVVNWQQDFDGSFQFKTVGYYDASLPPQQRLMLNT

KNIIWAGGQQKKPNSVCSESCPPGTRKAAQKGRPVCCYDCIPCAEGEISNETDSINCKQC

LEEYWSNAEKNKCVLKAVEFLSFTEIMGIILVFFSLFGVVLTTLVTVLFYRKKDTPIVKA

NNSELSFLLLFSLSLCFLCSLTFIGRPTEWSCMLRHTVFGITFVLCISCVLGKTIVVLMA

FKATLPGSNVMKWFGPVQQRLSVLAFTLIQIIICVVWITLSPPFPYKNMEYYKEKIIIEC

NLGSTLGFWAVLGYISLLSVLCFVLAFLARKLPDNFNEAKFITFSMLIFCAVWITFIPAY

VSSPGKLTVAVEIFAILSSSFGLLFCIFAPKCYIILLKPEQNTKQHMMGKTLKSY

>OlfCg3

MLLFLYTLLLFHHLHTKAGNTLCRIMGDPQYPLLSTDGDITIGALFPVHSTETLPSFEFT

KQPQLLSCSSVYLRDFRLAQILIFAIEEINRSVQLLPNISIGYRIYDTCGSRQSTMSAIM

GLMNGQEFGAGERCNGRSPIHAIIGESESSATVILSRTTGPFKIPVISHSASCECLSNKK

DYPSFFRTMASDYHQSRALAYLVKDLGWSWVGAVSSDNEYGNYGMSIFLKIAQEVGICVE

YYAKFYRTEAENLHKAVNMIKKGTAKVIVAFVSLPEMGILVDQLSIENITGLQMIGVKSW

ITVKSFITPSSFHVLKGSLGIAVKKISVEGFAEYAINEFWKKGFPCLKSEVNSSRYALSC

SRYEDLFVLKSISEDVPEQRYASYVYKAVYAVAHSLHSLFNCKEHEGCEKDLTIQPQQVV

EALKKVKFTIKTGDHVWFDSTGAAVAQYDVVNLQLDSYGSTLFKSVGYYDASLPPEQQFV

LNTTNIIWAGGEQKVRSVCSESCPPGTRKATQKGRPVCCYDCIQCADGEISNETDSNNCK

QCPEEYWPNTEKSKCVLKIVEFLSFTELMGIVLVIFSLFGVTLTVLVTFLFYSKKDTPIV

KANNSELSFLLLFSLTLCFLCSLTFIGRPTEWSCMLRHTTFGITFVLCISCILGKTIVVL

MAFRATIPGNNMIKWFGPVQQRLSVLAFTLIQILICVVWLTISPPFPSKNIKYYKEKIIL

ECSLGSTIGFWIVLGYIGLLAVLCFILAFLARTLPDNFNEAKFITFSILIFCAVWITFIP

AYVSSPGKLTVAVEIFAILASSFGLLFCIFAPKCYIILFKPEQNTKQHIMGKTISKN

>OlfCg4

MLLFIYILLLFHQLHTKAGNTQCRIMGDSTYPLISKNGDITIGALFPIHSTETLTSLKFT

RKPQPISCSSVNLRDFRLAEIMIFAIEEINKSESLLPNVTIGYQVYDTCGSRLSTMSAIM

GLMNSQGFDAEDGCKGQTPIQAIIGDSESSATVILTRTTGPFKIPVISHSASCECLSNRN

DYPSFFRTTASDYHQSRALVHIVKHLGWTWLGAVSSDNDYGNYGMSIFQKIAQEEGICME

YSVKFYRTEQEKLQKVVETMKKGTAKVIVAFVSFLEMGLLIDQLSVQNITGLQMIGVKSW

ITSENYITPKSFHVLGGSLGFAMRKMYIEGFSDFALKTFWEKHFQCSQTKPNASNYASSC

SRYNDLLMLKNYKEDVTEHRYSSNVYEAVYAVAYSLHSLLKCKERVKCEKGMTIQPQQVV

EALKKVNFSVKFGDRVWFDSTGSTVAQYEVVNWQRISDESFQFKTVGYYDASLPPNQRFL

LNTENIIWAGGMLEKPRSVCSESCPPGTRKASQKGRPACCYDCIPCAEGEISTDTDSNNC

KQCPGEYWSNDKKNKCVLKAVEFLSFTEVMGVLLLFVSLFGVGLTTLVAILFYNKKDTPI

VRANNSELSFLLLFSLILCFLCSITFIGRPTEWSCMLRHTAFGITFVLCISCVLGKTIVV

LMAFKATHPGSNVMKWFGPVQQRLSVLVFTFIQVIICVLWLTISPPFPYRNMKYYKEKII

LECSLGSIVGFWAVLGYIGLLAALCFILAFLARKLPDNFNEAKFITFSMLIFCAVWITFI

PAYFSSPGKLTVAVEIFAILASSIGLLFCIFAPKCYIILCKPEQNTKQHIMGKNN

>OlfCg5

MLILLSIFLLFNEPFTKAEDTICQKMGDPKYALFSKVGDVTIGGIFSIRSVEILPSLEFT

QKPQLLSCSSVNLRDFRMAQIMIFAIEEINRSESLLPNVSIGYKIYDSCGSRLSSISATM

SLMNDQEFPKGNICNGQYPIHAIIGETESSATVILSRTTGPFKIPVISHSATCECLSNRR

DYPSFFRTIASDYHQSRALAYIVKYFGWSWVGAVNSDNDYGNNGMAIFLKTAQEEGICVE

YSVKFYRTEPEKLQKVVETMKRSTTKIIIAFLSRVEMVNLLEELSIQNISGFQVIGVEAW

TTAKSLITPNSFHILGGSLGFAVRKIDTEGFLDYVIKSFWDSAFPCLIVNSSQLKVNCSS

YKDLLVLKNDNEDVPEQRYASNVYKAVYAVAHSLHSLLNCKEKKGCQKNLTIQPYQMVEA

LKKVNFTIKFGDRVWFDSTGATIAQYEVVNWQQDSDGLIQFKPVGYYDASLPPDKRFVVK

TENIIWAGGKLKKPKSVCSESCPPGTRKAAQKGRPVCCYDCIPCAEGEISNETDSVNCKQ

CPLEYWSNTEKNKCVFKSVEFLSFTEVMGVVLVFFSLFGVGLTMLVAILFYNKKDTPIVK

ANNSELSFLLLFSLTLCFLCSLTFIGRPTEWSCMLRHTTFGITFVLCISCVLGKTIVVLM

AFKATLPGSNIMKWFGPVQQRLSVLAFTLIQVIICVLWLIISPPFPYKNMKYYKEKIILE

CSLGATICFSAVLFYIGLLAILCFILAFLARKLPDNFNEAKFITFSMLIFCAVWITFIPA

YVSSPGKFTVAVEIFAILASSFGLLFCIFVPKCYIILFKNEQNTKQHIMGKIK

>OlfCg6

MFLFYTILLFHLLHTKVENSLCRIMGDSNYPLFSKNGDVSIGGIFAIHRKETLPSFEFMQ

KPQPLSCSSVNLRDFRLAQTMIFAVEEINRSKSLLPNVSIGYKIYDTCGSRLSTMTAIMG

LMNGQDFSTEDRCNGQSRLHAIIGESESSATIVLSRTTGPFRIPVISHSSSCECLSNKKD

YPSFFRTISSDYHMSRALVYIVKHLDWSWVGAVNSDNDYGNNGMAIFLKAAHEEGICVEY

SVKFYRTEPEKLKKVVDTINKGTAKVIVAFVSFVEMGLLIDQLSIQNITGIQMIGVEPWI

TANTYITSNSFRAMGGSLGFATKYIYIEGFAEYVMTPFWNTAFPCSESDRNHSHYELICS

RYEDLLALKNDNKDVNEHRYSSNVYKAVYAVAHSLHGLLNCKEQEGCEKGLTIQPQQVVK

ALKNINFTIKSGDSVWFDNTGSVVALYEVVNWQKDSDGSFQFKSVGFYDAMMPPYKNLRL

NTKNIVWAGGQLEKPRSVCSESCAPGTRKAAQKGRPVCCYDCIPCAEGEISNETDSINCK

QCPGEYWSNTERNRCVIKAVEFLSFSEVMGIVLVIFSLFGAGLTVLVAVLFYSKKDTPIV

KANNSELSFLLLFSLTLCFLCSLTFIGQPTKWSCMLRHTAFGITFVLCISCVLGKTIVVL

MAFKATLPGSNVMKWFGPVQQRLSVFAFTLIQVLICVLWLTISPPFPYKNMKYYKEKIIL

ECNLGNTIGFWAVLGYIGLLAALCFLLAFLARKLPDNFNEAKFITFSMLIFCAVWITFIP

AYVSSPGKFTVAVEIFAILSSSFGLLVSIFAPKCFIILLKPEQNTKQHVMGKTTCSL

>OlfCg7

**MSANSLFMVSLTFR**MILYFYTILFLRCFQAKTENAFCQIIGEAKYPLLSKDGDVTIGGIF

AIHSKETLPSFEFQQKPQPLLCSSVNLRDFRLAQIMIFAIEEINKSENLLPNVSIGYRIF

DTCGSRLSSMSATMGLMNELKFAAGETCNGQSPIHAIIGETESSATVILSRTTGPFKIPV

ISHSASCECLSNRNDYPSFFRTISSDYHQGRALAYIVKYLGWSWVGAVNSDNDYGNYGMA

IFLETAQKEGICVEYSVKFYRTETEKLKRVVDTIKKSTAKVIVAFVSFIEMGLLIEQLSI

QNITGFQIIGVEGWITSKNYITTNSFHSMGGSLGLALRKIHLEGFLDYVTKSFWSTAFPC

SQTEGILPTVGCSKYKDLLPLKNYTEDVPEHRYSSHVYKAIYAVAHSLHSLLKCKEGEDC

EKGHATQPQQVVEALKKVNFTVKFGDRVWFDRTGATVAHYEVVNWQQDTDGSFQFKQVGY

YDASLPPDQRFVLNIESIIWPGGNLEKPRSVCSESCSPGTRKAAQKGRPVCCYDCVPCAD

GEISNETDSVNCKQCPREYWSNGEKNKCVLKAIEFLSFTEIMGIVLVCFSLFGVGLTAVV

AILFWSKMDTPIVKANNSELSFLLLFSLTLCFLCSLTFIGRPTEWSCMLRHTAFGITFVL

CISCVLGKTIVVLMVFKATLPGSNVMKWFGPTQQRLSVLAFTFIQVLICVLWLTISPPFP

NKNMTYYKEKIIIECSLGSTISFWAVLGYIGLLAVLCFILAFLARKLPDNFNEAKFITFS

MLIFCAVWITFIPAYVSSPGKFTVAVEIFAILASSFGLLLCIFAPKCYIIICKPEQNTKQ

HVMGKTSLKVQ

>OlfCg8

**MSIKVVKSSIHSLILGSITHQNLVSG**MLLFLYSVLSLYHLNTKAENTLCQMIGNPKYPLL

SKDGDITIGALFVIHSEVSLPSFAFTQKPNLLSCSSLNIKDFRLAQTMIFAIEEINKNTN

LLPNISVGYQIYDTCGSRFHSMSATMALMNGPKNSEGYTCNEQSSVHAIVGETESSNTII

LSRTTGPFKIPVISPTATCECLSNRKEFPSFFRTIASDYHQSRALAYIVKHFGWTWVGAV

NSDNDYGNNGMATFQKTAKEVQICVEYSVKFIRTETEKIRNVVNVIKKGTAKVIVAFLTG

FEMKSLLEQLSSQNITGLQMIGVEAWITSKTLITSKSFHVLGGSLGFAVRKIQIEGFADY

VMKAFWDTAFQSFSNADLNYSQYQDLLLVKNYNEDVLEQRFVSYVYKAVYALAHSLHSLL

RCTEQGGCGKALTIQPHQLVEALRKVNFTVKMGDQVWFDSTGGVIAQYDVVNWQQNSDGS

VQFQSVGYYDASLSPDQRFMLNTEKIVWAGGQLEKPRSVCSESCPPGTRKASQKGRPVCC

HDCIPCADGEISNETDSNNCKQCPGEYWSNANKNKCVTKAVEFLSFTEVMGIILVFFSLF

GAGLTVLVAILFYSKKDTPIVKANNSELSFLLLFSLTLCFLCSIPFIGRPTQWSCMLRHT

AFGITFVLCISCILGKTIVVLMAFKATLPGSNIMKWFGPVQQRLSVLAFTLIQGLICVIW

LTKSPPFPYKNMKYFQEQIILECSVGSTIGFWAVLGYIGLLAVLCFILAFLARTLPDNFN

EAKFITFSMLIFCAVWITFIPAYVSSPGKLTVAVEIFAILASSFGLLFCIFAPKCYIILL

QPEQNTKQQMMAKTPSKI

>OlfCg9

**MQLSSFFK**MLLYLCTLLLFFKLKVSVGNASCKIMGEPKYPLLFKDGDVTIGALFPVHSIE

TAPSFEFTQKPQLLSCSSVNLRDFRLAQILIFAIEEINRSESLLPKLSLGYKIYDTCSSK

LSSMSATMALMNSFEFAGRDKCNGQTSVHAIIGETESSATVILTRTTGPFKIPVISHAAS

CECLSSRKEYPSFFRTISSDYHQGRALAYIVKHLGWSWVGAVNSDNDYGNYGMAIFLNTA

HKEGICVEYSEKFYRTEPEKLKKVVDTIKKSTAKVIVAFISFLEMRLLVEQLSAENITGL

QIVGVEGWTTSKSLITPKTLNVLRGSLGFAMRKIYIEGFAEYVLKPFWDTAFPCIPNQRN

DSWVILNCSRYQDLLVLKNYIEDVPEHRFSINVYNAVYAVAHALHSLFKCKEQEGCEKDL

LTQPQQVVDALKKVNFTVKMGDRVWFDSTGATLAQYEVVNWQQNSDGSVNFKKVGYYDAS

LPPDQRFVLNIKEILWAGGNLKKPRSVCSESCPPGTRKAAQKGRPICCYDCIPCADGEIS

NDTDSNNCKQCQGEYWSNADKNKCVQKSVEFLSFTEVMGIVLVFFSLFGAGLTVLVAILF

YSKKDTPIVKANNSELSFLLLFSLTLCFLCSLTFIGQPTQWSCMLRHTAFGITFVLCISC

VLGKTLLVLMAFKATLPGSNIMKWFGPVQQRLSVLAFTFIQVLICVLWLSISPPFPHKNM

KYYEEKIILECSLGSTIGFWAILGYIGLLAALCFILAFLARTLPDNFNEAKFITFSMLIF

CAVWITFIPAYVSSPGKFTVAVEIFAILSSSISLLLCIFAPKCYIILLKPEQNTKQYMLG

KTTSKFY

>OlfCg10

**MTCLMSYPFVCGYNQSSVLIIFSYLKTLYQFKLCLKFSVDLNEGLG**MVSFIWTLLLVVPL

QAKAENSLCRMMGQNINPLISKEGEVTIGALFPIHSIEILPSFEFTVKPQLLSCSSVNLR

DFRMAQTMTFAIDEINKNQSLLPNVSIGYRIYDTCGSRLSSMSATMALMNGKEFSAEDKC

NGQSAIHAIIGETESSATVILSRTTGPFKIPVISHSATCECLSNRKNHPSFFRTIASDYH

QSRALAYIVKHFGWSWVGAVNSDNDYGNNGMAIFLNTAQEEGICVEYSEKFYRTDPEKLR

KVVDTIKNSTAKVIVAFLTSLEMENLLQELTKANITGLQIIGVEAWITANSLLTPNSFRV

LGGSLGFAVPKVNIQGFSNYVIKDFWETAFPCSETEINVSQYSSLSCNSYDDLLLLKNYN

EDVPEQRYASNVYKAVYAVAHALHSLLKCKVNEGCKKDLKIQPQQVVDTLKEINFTINMG

DRVWFDSTGATIAQYEVINWQQGSDGSIQFKTVGYFDASLPHDQRFVLNTESIIWTGGQL

EKPKSVCSENCPAGTRKAVQKGRPVCCYDCIPCADGEISNDTDSVNCKQCPGEYWSNAEK

NRCVLKTVEFLSFTEVMGIVLVFFSLFGVGLTVLVAILFYSKKETPIVKANNSELSFLLL

FSLTLCFLCSLTFIGRPTEWSCMLRHTTFGITFVLCISCVLGKTVVVLMAFRATHPGKDI

MKWFGPVQQRLSVIALTLIQVLICVLWLTISPPYPYKNMKYFKEKTILECNLGSTIGFSA

VLGYIGLLAVLCFFLAFLARTLPDKFNEAKFITFSMLIFCAVWITFIPSYVSSPGKLTVA

VEIFAILASSFGLLFCIFAPKCYVILLKPEQNTKQHIMGKISSKSY

>OlfCg11

**MMSESLIHTHYGYKSCHIVLSQVHLAPLNRIVS**MSVFLYTVLIFIFYTKAENPLCQMMGN

PKFPLLSKDGDVNIGAIFSVHSTEILTSFTYTQKPQLLSCSSVSLRDFRMVQTMIYAIEE

INRSLGLLPNITVGYQIYDACGSRLSAMSATMALMNGPEFTWRDRCTGQSPIHAIIGETE

SSATVILSRTTGPFKIPVISPSATCECLSNRKEYPSFFRTIASDYHQSRALAYIVKHFGW

SWVGAVNTDNDYGNNGMTTFLNTAQEEGICVEYSVKFYRTEPEKLQKVVETIKKGTAKVI

VAFLTSSEMYNLLEQLSIQNITGLQMIGVEGWITAKSLITPNSFHVLGGSLGFAVRKTAI

EGFADYVIKSFWETAFPCTMTIGNSSQYSLSCGIYQDLLLLKNYNEDVPEQRYSTNVYKA

VYAVAHSLHSLLKCKEDGCKKGLAIQPQQVVGALKKINFTLKLGDIVSFDSTGATVAQYE

VVNWQKDANESIKFKPIGYYDASLPPHQRFVLHTENIIWAGGQLDRPRSVCSESCPPGSR

KAAQKGRPVCCYDCIPCAEGEISNQTDSNNCKQCPGEYWSNAEKNKCVLKDVEFLSFTEI

MGIVLVIFSLFGAVLTALMAILFYRKKDTPIIKANNSELSFLLLFSLILCFLCSLTFIGR

PTEWSCMLRHTAFGITFVLCISCVLGKTIVVLMAFKAALPGNNIMKWFGPVQQRLSVFAL

TLIQVLICVLWLTMSPPFPHKNLKYYQEKIVLECNLGSNIGFWAVLGYIGLLAVLCFILA

FLARKLPDNFNEAKFITFSMLIFCAVWITFIPAYVSPPGKFTVAVEIFAILASSFGLLFC

IFVPKCYIILCKPEKNTKQHLMGKVLM

>OlfCg12

MSVFFCTLLIFFQLYAKAEKPICIMMGDPKYPLLSKEGDISIGAVFPVHSIETLPLFKFT

QKPQLLSCSSVSIRDFRMAQIMAFAIEEINRNESLLPNVSLGYQIYDTCGSGLSLMGANM

ALMNVQEFASRGSCNGQSPVHAIIGETESSNTVILSRTTGPFKIPVISPSASCECLSNRK

EYPYFFRTIASDYHQSRALAYIIKYFGWSWVGAVNSDNDYGNHGMAIFLSTAEKEGVCVE

YSVKFQRTEPEKLKKVVDTIKKGTSKVIVAFLTEFEMKNLLEYLIIQNVTGLQVIGVEAW

ITANSMIKPNSFHVLGGSLGFAVKKLNIEGFEDYVTKAFWETAFPCSQTSQKENSQYKLI

CNIYRDLLVLKNDNKDVPEQRYASNVYKAVYAVAHSLHNILKCRENGACETDMKIQPQQV

VEALRKVNFTIKMGDCVWFDSTGAVVAQYEVVNWQPDYNGSIQFKPVGYYDASLPPNQRF

VINTENIIWAGGQLKKPRSVCSENCPPGTRKAVQKGRPVCCYDCIPCAEGEISNETDSNN

CKQCPGEYWSNAEKNKCVLKAVEFLSFKELMGIVLVFFSLLGAGLTTLVAILFYSKKDTP

IVKANNSELSFLLLFSLMLCFLCSLTFIGRPTEWSCMLRHTAFGITFVLCISCVLGKTIV

VLMAFKATLPGSDVMKWFGPIQQRLSVLVITIVQVLICVLWLAVSPPLPYKNTKYFKEKI

ILECSLGSTIGFSAALGYIGLLAILCFILAFLARTLPDKFNEAKFITFSMIIFCAVWITF

IPSYLSSPGKLTVAVEIFAILASSFGLLFCIFLPKCYIIMCKPEQNTKLHIIGKLNK

>OlfCh1

MFGFGFLLLCSIKAKEVESNCNMIGKPENPLLSQDGDIIIGGAFSIHNKINLIIPSFTEK

PHHLMCTSLNLRELHFAQTMIFAIEEINSKRSLLPNISIGYQIFDSCGSTLASMRSSMAL

INGQELTAEHTCSGKPAVKAIIGESESSTTIVLSRAAGPFNIPVISHFATCACLSSRKQF

PSFFRTIPSDYYQSRALAQLVKHFGWTWIGAVRSDNDYGNNGMATFVEVAEKEGVCIEYS

EAISRTNSKDKIAKVVEVIKKGTAKVLMAFLAQGEMDVLLEELIRQNVVGLQWVGSESWI

TSSYLATERTLNILGGAIGFTIIKSKIPGLKEFLLKVGPSQNLSNALLGEFWEMVFGCCL

SPTVCPNSEHATFCDGSENLTNVSNAFTDVSELRISNNVYKAVYAIAYALHNTITCKNSN

GGNENITCGDVDLLVSSQVLHSLQNVNFTMDSGETVYFDKNGDPMAKYELVNWQKNGAGE

TKFITVGQYDASLSSEQQFVINSFDIIWAGDSPTKPISVCTESCQPGFRQAVIKGRPVCC

FECLQCPAGEISNTTDSAECIKCPLEYWSNKNHSICVLKKVEFLSFEENMGILLTAFSLT

GVTLTIAVAIVFYKFIDTPLVKASNTELSFLLLFSLSLCFLCSLTFIGRPTEGSCMLRHS

SFGVTFALCMSCVLTRTIAVVMAFKTTVPGSGLPHCSLPLQRISVFCCTVFQVMICILWL

ALARPMPYKNSMYSLDKVILECDLGSAIGFWAVLGYIGLLSVLCFFLAFLARKLPDNFNE

AKFITFSMVIFCAVWITFIPAYISSPGKLTVAVEIFAILASTFGLLFCIFTPKCYIIIFK

PEQNTRKHIMGKT

>OlfCj1

MLLLATILTTIACTLSAAEPECEAYMTDELLYFSKEGNVSIGGVFSFHQNPVGVNPTLRT

NPGNIRCNGLDPGELQYAITMIFAIEEINNRTDLLPGFILGYRIYDSCPSIPLSVGASLT

LMNGQMETKKSCASPSAVQAVIGETTSTSTIDIARTIGPFKIPVLSHSATCACLSNRQQY

PSFFRTIPSDYYQSRALVKLVTYFGWKWVGAVRSMGDYGNNGMATFLEAAEKEGICVEYS

VSIYRTNSREKILEVTDIIKKSTSKVIVAFADGNDLDMLIKELYYQNVTGYQWVGSEGWI

TYRFLATAINYAVVGGAIGFAVPNAYIPGLKEFITGSQPSLRPGNTGLVELWESVFDCTL

NSQTHNASKICNGQESLANINTRFTDVSDASLLNNVYNAVYAVAHAVEELLTCEKGKGPF

HKKTCAEKGKIQPWQVLYYLTQVNFTTKNGENVHFDKHGDPVARYTLVNWQMSYEGIITF

ESIGLYDASKPEGQEIQMRDDIEAIWAGNQKKVPLSVCSETCLPGTRQAFVKGKPICCFD

CIDCADGEFSNTTNAVTCIPCPLEYKSNGNRTQCVLKNIEFLTFNEVMGNILVTFSMCGG

CLTITVGLIFFYHRHTPIVRANNSELSFLLLFSLTLCFLCSLTFIGQPTEWSCMLRHTAF

GITFVLCISCVLGKTLVVLMAFRATLPGSNVMKWFGPPQQRLSVFVFTFIQLLICMLWLT

MSPPFPNKTTNNYKDKIILECDSGSAVGFWAVLSYIGFLAILCFILAFLARKLPDNFNEA

KFITFSMLIFCAVWITFIPAYISSPGKFTVAVEIFAILASSYGMLFCIFIPKCYIILLKP

DLNSKKKIMGKVSSRVF

>OlfCk1

**MRISLGWVVCLVKE**MHVNLLLILTLLCIRRLFPAVCGVHLGTCILQGDPQPPSLFSKGDF

VIGGTFTIHYYLRTEKRTYTVRPQPLMCSGSMDFRELRFARVLQFAIQEINNSSNLLPGI

TLGYHIYDSCASVPMVIKVAVQLANGLQLAFNDTDSCAQSSEVLALVGESGSTAAITTSR

LFGPFGIPQVSHYATCACLSDKRQHPTFFRTIPSDHHQAAALARMVKRFGWTWIGAVRSD

SDYGNNGMASFLKAAEVEGICVEYSEAYYRTQTRNKLQRVADVIRRSTARVIVAFMAAGD

MRFLLEELSQQPPPPMQWIGSEAWVTDPQMLRFNLSIGAVGFAIPRSVIPGFRKFLLDLS

SEQALKIPVLKEFWESSFGCSLKQHTGYFSGMPACDGTEDLGTLKNPYTDTSQLRISNMV

YKATYAIAHALHGIVCNGKLCDKNIKVEPRKVSDQLKQVNFSKNNYSVSFDANGDPVAVY

ELVNWQLQGDGSIDFVTVGKYDASQPKGQEFSLNRAIIWYDGTEKVPVSVCSESCPPGTR

KAVKKGRPVCCYDCINCADGEINNETDSLDCHKCQPDYWPNAEKIKCLPKPVEFLSWDEI

LGNTLAAFSIAGSLVALSMALVFYKNRVSPIVRANNSELSFLLLFSLTLCFLCSLTFIGQ

PTEWSCMLRHTAFGITFVLCISCVLGKTIVVLMAFKATLPGSNVMKWFGPPQQRLSVFGF

TLVQIIICVLWLTISPPFPYKNMQHYKDKIILECSLGSAVGFWAVLGYIGLLAFLCFVLA

FLARKLPDNFNEAKFITFSMLIFCAVWITFVPAYVSSPGKFTVAVEIFAILASSFSLILL

IFAPKCFVIVFRPEENTKRHLLGKVPPKAL

>OlfCk2

MNAHLLFTVKMLCIARLCIAVCGVDLGTCILQGDAQPPALSEDGDFIVGGAFTIHYYVRT

EKYTYTRRPQPLECSGSMDFRELRFARALQFAIQEINNSSDLLPGIFLGYHIYDSCGSVP

MAIKVALQLANGLDPKYNDTDSCAKSAAVLALVGDSASTPAISISRIFGPFEIPQVSHYA

TCACLSDKRQHPTFFRTIPSDHHQAAALARMVKRFGWTWIGAVRSDSDYGNNGMASFLKA

AEKEGICVEYSEAYYRTYPLSKLKRVADVIRRSTARVIVAFVAAGDMRFLLEELSKEPLP

PMQWIGSEAWVSDPQMLRFNLSIGTMGVAVPRSLIPGFRKFLLDLSPYKVLKFPLLTEFW

ESSFSCSLQQQTDPSTGMPACDGTEDLGRLKNPYTDTSQLRVSNMVYKATYAIAHALHGI

VCNETQCSKNIKVKPRQVFDQLKQVNFSKNNYSVSFDANGDPVAVYELVNWQLQGDGSID

FVTVGKYDASQPEGQEFSLNKAIIWYDGSEKVPVSVCSENCPPGTRKAVKKGRPVCCYDC

IPCGEGEISNKTDSPDCDKCPPDYWPSTEKDKCLPKPVEFLSWDEILGIILAVLSVSGSL

LALSMALVFYKNRASPIVRANNSELSFLLLFSLTLCFLCSLTFIGQPTEWSCMLRHTAFG

ITFVLCISCVLGKTIVVLMAFKATLPGSNVMKWFGPPQQRLSVFGFTLVQIIICVLWLTI

SPPFPYKNMQHYKDKIILECSLGSAVGFWVVLGYIGLLASLCFVLAFLARKLPDNFNEAK

FITFSMLIFSAVWITFIPAYVSSPGKFTVAVEIFAILASSFSLILCIFAPKFFIILFRPE

ENTKKHLMGKVQSKSY

>OlfCk3

MYNNVLLIVTLQCIITRLYVVYGNRLGSCILQGDPQPPVLFDEGDFIIGGAFSIHYYLRT

EKHTYTMRPQSLECSGSMDFRELRFARVLQFAIQEINNSSDLLPGITLGYRIYDSCGSVP

MAVKLSFQLANGLDLIFNDNDSCSKSAAVAALVGESGSTPSISISRLYGPFGIPQVSHYA

TCACLSDKRQHPTFFRTIPSDHHQAAALARMVKRFGWTWIGAVRSDSDYGNNGMASFLKA

AEEEGICVEYSEAYYRTQPRSKLMRVADVIRRSTARVIVAFMASGDMRLLLEELSQQPLP

PMQWIGSEAWFTDPEMLRFNMCIGGVGFAIPRSVIPGFRTFLLDLSPQRAMKFPLLTEFW

ESSFSCSLKQQTGPSTGMPACDGTEDLGRLKNPYTDTSQLRISNMVYKATYAIAHALHGI

ICNEKKCNKNIKIEPREVLDKLKQVKFSKNNYSVSFDAYGDPVAVYELVNWQLQKDGSID

FVTVGKYDSSQPKGKEFSLSRAIIWYDGTEEVPVSVCSESCPPGTRKAVQKGRPVCCYDC

INCADGEISNDTDCLDCHECLSDYWPNNEKNKCLRKPVEFLSWDEILGIILAAFSVAGSL

VALSMALVFYKNRASPIVRANNSELSFLLLFSLTLCFLCSLTFIGQPTEWSCMLRHTAFG

ITFVLCISCVLGKTIVVLMAFKATLPGSNVMKWFGPSQQRLSVFGFTLVQIIICVLWLTI

SPPFPYKNMQHYKDKIILECSLGSAVGFWAVLGYIGLLAFLCFVLAFLARKLPDNFNEAK

FITFSMLIFCAVWITFVPAYVSSPGKFTVAVEIFAILASSFGLILCIFAPKFFIIIFRPE

RNTKKHIMGKVPSKAL

>OlfCm1

MEPLFALIHVVMNIISFAKANETACTLQGQPVYPQLWKDGDIIIGGVFSFHSSWEIRQLT

YSVTPPPLKCISLNYRDFQYVQSMLFAVEEINNSSTLIPGVSLGYRIYDTCGSMAMAVRV

TMALANAHENTTSDGPCTKQAYVQTILGDTTSSACMAMAKTIGPFNLPMISHYATCECLS

DKVKYPSFLRTITSDYYQSRALAELVRHFGWTWVGAIRTDDDYGNNGMATFTKVAEQMGI

CLEYSVPFFRTYAEDKVIRIIEQIKSSTSRVIVAFLVHWDLEVLLHKFVEYNITGYQWVG

TEGWISDSVIATMDTHHILQGAVGLAIPKTEVTGLKEYILNIKQLKSSGSTIFSELWESL

YQCKYPNKDDSVSMNACTGNEELSQMQNSFTDMSLMPIFSNVYKGVYAVAHTLHELLGCK

EKCASKKQLDPITFLKHLRKVRFKTKDGEDVYFDENGEAVARYEVINWHPSNGKHDQFVT

VGLYDASLPVKDRLAVNVASIVWSNNATKVPVSVCSESCSPGTRKAVKKGKFICCYDCIS

CADGEISNTTDSVTCLRCHQELWSNLQKDVCVEKETEFLSFEEIMGILLTTISIVGAFVT

MIIAVIFFRYKNTPIVKANNSELSFLLLFSLMCCFLCSLTFIGQPTEWSCMLRHTAFGIT

FVLCISCVLGKTIVVLMAFKATLPGSNVMKWFGPPQQRLSVLGFTLIQVLICVLWLTISP

PFPFKNFNYFKEKIILECHVGSSIGFWAVLGYIGCLALLCFILAFLARKLPDKFNEAKFI

TFSMLIFCAVWIGFIPAYVSSPGKFTVAVEIFAILASSFGLLFCIFLPKCYVILFKPDKN

SKKHIMGKLTSN

>OlfCm2

MEPLVALLHMVIAIMTFSKANETTCTLQGEPVLPELWKDGDIIVGGVFSFHSSWEVRQLT

YTFVPPPLKCISLNFRDFQYAQSMLFAIEEINNSSTLLPGVSLGYKIYDTCGSVAVGVRA

AMALANGHEKISVEGPCTKHAEVQAILGDTTSSACMAITKSIGPFKLPLISHYATCECLS

DKVKYPSFLRTIASDHYQSRALAELVRHFGWTWVGALRTDDDYGNSGMATFTKVAEQMGI

CLEYSLPFFRTYTEDKVMRIIEQIKSSTSRVIVGFLAHWDLEVLLHKFVEYNITGYQWVG

TEAWISDSVIASMDTHHILQGAVGLAIPKTKVTGLQEFILNITPLKSSGGAIFSEFWEAL

FQCKYSNKDTSVSINACTGKEELSQVENLFTDMSLMPIFSNVYKGVYAVAHALHELLGCK

DKCALKKQPDPVTFLKHIRKVHFKTKDGEEVYFDENGDPVAKYDIINWQQSTKQHYEFVT

VGFYDASFMGMDRLAVNMSSIFWAINSTKVPVSVCSESCPHGTRKAVKKGKPICCYDCIS

CTEGEISNTT**DSVTCLRCHQDFWSNLQKDQCIRKQTEFLSYEEIMGLLLTTISVAGAFLT**

**MLIAVIFFKYKNTPIVKANNSELSFLLLFSLMLCFLCSLTFIGRPTEWSCMLRHTAFGIT**

**FVLCISCVLGKTIVVLMAFKATLPGSNVMKWFGPPQQRLSVLAFTLIQVLICVLWLTISP**

**PFPFKNVKYFKEKIILECNVGSVLGFWAVLGYVGLLAILCFFLAFLARKLPDNFNEAKFI**

**TFSMLIFCAVWVAFIPAYVSSPGKFTVAVEIFAILASTYGLLFCIFIPKCYVILLKPDKN**

**SKKHLIGKGPPRVL**

>OlfCn1

MKNFNILLFCSLLYVQTSSMCQLLRTFEMPNIFKVGDIMIGGIFPIFNKQENIIGSFERN

MQRIKCTGFDLRAFRWIQSMMFAVDEINKNEELLPHISLGYKIMDSCASPTNVLRAVFSL

VSEQKEQEFTSQCHLPLTALVAESGSSQSLAVAGMLGPFRVPMVSYFSTCACLSDRKKYP

SFFRTIPSDFYQAKALASLVKQFGWTWIGALQSDNDYGRNGISAFTKEVEKMGVCIAFVG

TILRTYPQSKITEVVEMIKESTVKVILAFVPEGDLYPLMKEVVNRNITGIQWIASEAWVT

AARPSTPEMFKSFGGTVGFVVRKMAMLKLRPYLENISPYSPTQSAFVSDFWETVVGCKPC

LNCEPSANSTLNGQMCTGQEKLTFTDKFFDVTQVRVTYNVYQAVYAIAHAIHKVLYCQGD

NNLSKMCLNVSQITPKQVSDQLERVNFIDEYGENVFFDENGDPPASYELINWQLNQGEVQ

HVTVGYFSKSPDGTYKLTIKEDNVHWSTENLIPKAVCSDTCPKGTRKAQIKGRPVCCFDC

IPCADGSISNTTGAADCTLCPKEYWSNERRDKCLVKTIEFLSYTETMGIILTALSLFGAS

LTVATMIVFIHYRETPIVKANNSELSSLLLSSLFFCFLCPLTFIGEPTHWSCMLRHTAFG

LTFALCISCVLGKTIVVVTAFRATLPGNKLSGKFGPVQQRAIVFLCTAIQIVICALWLLI

KPPFPDKALRYDHKKIILECNTGSDAGFYAALGYVGLLSTICLILAFLGRKLPDNFNEAK

FISFSMLIFCAVWVTFIPAYISSPGKYTVAVEIFAILSSAFGLLLCIFAPKCFIILIKPE

RNTRKHVMGKSKTSL

>OlfCq1

**MDTWITLYLCFYLFFKSISASSISKSGSCQLQGHFKLNG**MYQDGDLMIGGLFAFHLITVF

PELNFKREPEQTHCERFYMASFQQAQTMVYAINEINNNPNLLPNITLGYHLYDNCVKLGV

AFRSATALVSGTEESFNVLNCTGPPPIIGIVGDPGSTHCIAISSVLGLFRIPMVSYYATC

SCLSDRSKYPSFFRTIPSDAFQVRAMVLILKHFGWTWVGLLYSDDDYGINAAQSFQKEVQ

LFGGCVSFSEILPLDNNHMDIQRIVQVIQASTAIVVVVFSTEAYLLSLMDEVVLQNVTGR

QWIASEAWATSSVFHTKRLLPFLGGTLGIAIRRGEIQGLRDFLLSLHPDSNLRNNMVKIF

WENMFECSFDTVGRKGETMCTGQEDIRTTNTPYSDVSELRASYNVYKAVYALAHALHDLI

QCEEGKGPFRCDGITNLKPWQLVHYLQKVNFTTGFGDHVSFDENGDALAIYDVMNWHPSS

DGSIVVRTVGVVDEGASTGKVLTLEEDEIYWNTAKNKPARSVCSESCPTGTRRTRRKGLP

VCCFDCLPCADGAISTIPDSIECVVCPDEFWSSPKKNQCVPKDVEFLTYGDPLGISLTTA

SLLGSCICSAVVVIFAHHRHTPVVRANNSELSFLLLVSLKLCFLCVLLFIGQPQLWTCRL

RHAVFGISFVLCISSILVKTMVVIAVFKSSRPEGKSAMKWFGSHQQRCTVLVLTALQVVI

CAVWLTNASPKPYKNNQYTSSKIVYECTIGSVVGFAMLLGYIGILAAVSFLLAFLARNLP

DHFNEAKFITFSMLIFCAVWIAFVPAYVSSPGKYAVAVEIFAILASSFGLLAAIFAPKCY

IIILHPERNTKKAIMGRAT

>OlfCq2

**MLCE**MWITLNICLYMYFNHISGVFTSESCQLQGCFKLNGMYQDGEFIIGGLFEVQNLKVF

PELSFTNEPELPQCEEFYMASFQQALTMVFAINEINSNSKLLPNITLGYQIYDNCLRLGV

AFRAAMSLVSGTEESSNLSCTGPPPVIGIVGDPGSTQSITISSVLGLYRVPMVSYYATCS

CLSDRKKYPSFFRTIPSDAFQVRAMVQILRHFGWTWVGLLYSNDDYGIYAAQSFQQEMQI

LGGCVAFSEMLPYDNNGRDIQRIAGVIQKSTAKVVVAFSTDLASLMDELLLQNVTGKQWI

ASEAWTTTPVLQTPQYLPLLGGTLGIAIRRGQIQGLYEFLKNLRPDKNPKNSTIRIFWET

MFGCKFEVGGKKVDEQQERGRNKCSGQEDLNNSETAYTDVSELRASYNVYKAVYALAHAL

HDLVKCEEGKGPFTGNSCADISNLKPWQLVHYIQNVKFTTGFGDHVSFDENGDALAIYDV

MNWHPRSDGSIIVHVVGVYEEAILGKVLTLDEDALFWNFETKKPPDSVCSESCPPGTRRA

RKKGLPVCCFDCLPCGDGEISNTTDSPECFICPEDFWSSPENDRCVPKEMEFLSYDDPLG

ISLTTASLLGTCFCVLVLVIFAYHHNTPVVRANNSELSFLLLVSLKFCFLCVLLFIGRPR

LWTCQLRHAVFGISFVLCISSILVKTMVVIAVFKTSRPEGKTSIKWFGSAQQRGTVIALT

SAQVAICTVWLSSASPTPHKNNQYIHSKIVYECAIGSVAGFSLLLGYIGLLAAVSFLLAF

MARKLPDNFNEAKFITFSMLIFCAVWIAFVPAYASSPGKYAVAVEIFAILASSFGLLAAI

FAPKCYIIILHPERNTKKAIMGRSTQNK

>OlfCq3

MCITLNICLHLSFIFISVNSDSCQIQGHFKLNRMYKDGDYIIGGLFEVQHLKVFPELSFR

MEPEQPKCEEFYMSSFQQAQTMVFAIDEINKNPNLLPNITLGYHLYDNCLKLVVAFRAAT

TLISGTEETFSNFNCTGPPPVIAIVGDPGSTHSIAVSSVLGLFHVPLISYYATCSCLSNR

KKYPSFFRTIPSDAFQVRAMVQILKYFGWTWVGVLYSDDDYGIYAAQSFQQEMQRFKGCV

AFSEIVPYDNHRDIQRIVAVIKASTARVVVAFSTDLLPLMEELLQQNVTGRQWIASEAWS

TSPVLHLPRFVPLVRGTLGIAIHRGKIKGLHEFLLHIQPDNDPTNNMVRIFWENMFGCSF

EKGNGYGEKMCTAQEDLNITVNEYNDVSELRASYNVYKAVYALAHALHDLMQCEEGRGPF

SGNSCADITKLKPWQMVPYLQKVNFTTGFGDHVSFDENGDALAIYDVMNWHPSSDGLIVV

RTVGVVDEGASAGRVLTLDEDAIYWNFETKKPPRSVCSESCPPGTRRAMRKGLPVCCFDC

LPCADGEISNMSDATECTSCPNEFWSSPKKDQCVPKEVEFLSYEDPLGISLTTASLLGTC

FCALVLAVFAHHHNTPVVRANNSELSFLLLLSLKLCFLCVLLFIGRPQLWTCQLRHAMFG

ISFVLCVSSILVKTMVVIAVFKSSRPEGKSAVKWFGAVQQRGTVLVLTAVQIVICVVWLS

TASPTPHKNILYVRSKIVFECSIGSMAGFAILLGYIGILAAVSFLLAFLARNLPDNFNEA

KFITFSMLIFSAVWIAFIPAYVSSPGKYSVAVEIFAILASSFGLLVAIFVPKCYIIILHP

ERNTKKAIMGRTAEK

>OlfCq4

MWAVLLFCLVLSCYYICVTLMASTGICQQQGHFTINGMHQDGDFVIGGLFDVQTYLKVYP

EISFRTQPKLPNCELFYMESFQQALTMVFAISEINHNPNLLPNITLGYQIYDTCLRLRVA

FQAATALISGTEETISDFNCKGPPPVIGLIGDPGSTHSIAISSVLGLFRMPMISYYATCS

CLSDKKKYPSFFRTIPSDTFQVRAIVQTLRHFGWTWVGLIYSNNDYGIYAAQSFHQEMQL

FGHCVAFSEILPQDNNPRVIDHIMGVIQASTARVVVVFSASSLLIPLMNKVVLHNLTSRQ

WIASEAWVTAAVFRTPYYQPFLKGTLGIAIRRGEIQGLHSFLLRLHPNSDQRNNIVRIFW

ETMFGCSFETGDKETFGQQMKKVCTGLEDLSTANTPYTDVSGLRATYNVYKAVYALAHAL

HDLMQCKEKRGPFSGNSCADITDLKPWQLVHYLKKVNFTTSFGDSVSFDNNGDALAIYDV

LNWQPSSEESIKLHNIGVVNEVATEMVLTLNNDEIYWNFEAQKPPQSVCSESCLPGTRRA

MRKGLPVCCFDCLICGDGEISNTTDAIKCTVCPDEFWSNLNKDQCVPKEIDFLSYEDPLG

ISLTTTSLLGTCFCALVMIIFTFHRNTPIVRANNSELSFLLLLSLKLCFLCVLLFIGQPQ

LWTCQLRHAVFGISFVLCISSILVKTMVVIAVFKSSRPEGSGAMKWFGTAQQRCTVLVLT

ALQIVICAVWLSTSSPTPYKNNQSIRSKIVYECAIGSLAGFSLLLGYIGLLAAISFLLAF

LARNLPDNFNEAKFITFSMLIFCAVWVAFVPAYMSSSGKYAVAMEIFAILASSFGVLVAI

FAPKCYIIILHPERNTKKAIMGRENKNK

>OlfCq5

MWAVLLFCLVLSCCYICVTLIASTGTCQQQGHFTINGMHQDGDFVIGGLFDVQTYLKVYP

EISFRTQPKLPNCELFYMTSFQQALTMVFAISEINHNPNLLPNITLGYQIYDTCLRLRVA

FQAATALISGTEETISDFNCKGPPPVIGLIGDPGSTHSIAISSVLGLFRMPMISYYATCS

CLSDKKKYPSFFRTIPSDTFQVRAIVQTLRHFGWTWVGLIYSNNDYGIYAAQSFHQEMQL

FGHCVAFSEILPQDNNPRVIDHIMGVIQASTARVVVVFSASSLLIPLMNKVVLQNLTSRQ

WIASEAWVTAAVFRTPYFQPFLKGTLGIAIRRGEIQGLHSFLLRLHPNSDQRNNIVRIFW

ETMFGCSFETGDKETFGQQMKKVCTGLEDLSTTNTPYTDVSGLRATYNVYKAVYALAHAL

HDLMQCKEKRGPFSGNSCADITDLKPWQLVHYLKKVNFTTSFGDSVSFDNNGDALAIYDV

LNWQPSSEGSIKLHNIGVVNEVATGMVLTLNNDEIYWNFEAQKPPQSVCSESCLPGTSRA

MRKGFPVCCFDCLICGDGEISNTTDAIKCTVCPDEFWSNLNKDRCVPKEIDFLSYEDPLG

ISLTTTSLLGTCFCALVMIIFTFHRNTPIVRANNSELSFLLLLSLKLCFLCVLLFIGQPQ

LWTCQLRHAVFGISFVLCISSILVKTMVVIAVFKSSRPEGSGAMKWFGTAQQRCTVLVLT

ALQIVICAVWLSTSSPTPYKNNQSIRSKIVYECAIGSVAGFSLLLGYIGLLAAISFLLAF

LARNLPDNFNEAKFITFSMLIFCAVWVAFVPAYMSSSGKYAVAMEIFAILASSFGVLVAI

FAPKCYIIILHPERNTKKAIMGRENKNK

>OlfCq6

**MWITLLINIHLILKCISAA**VVLRAGACQLQGRFRLNGMYQDGDVILGGLFEAHFFTLFPE

LTFRTEPAPPYCEIFNMESFQYAQTMAFAINEINRNPSLLPNISLGYHLYDNCVMLGMAL

RAAMSLVSGIEESFLNLNCTGPPPIIGVVGDPSSTPSIAISSVLGLFRVPIVSHYATCSC

LSDRKKYPSFFRTIPSDAFQVRAMIQLISHFGWTWIGLLYSDDDYGTYAAQSFHQEMQLF

GFCIAFSEPLRYDSNPRDIQRLMEVIQASTSTVVVVFSPSTLVIPLMNEVVLQNMTGRQW

IASESWATSPVFYTPRFLPFLGGTLGIAIRRGEIEGLREFLLQLRPKNDPRNNMLKIFWE

NMFGCSFETGPHVKNVCTGQEDLSTTNTPYTDVSELRAANNVYKAVYALAHALHDLMKCE

EGKGPFSKNSCAEISNLKPWQLVHYLQKVNFSTRFGDHVSFNKNGDALAIYDVMNWQPGS

DRSIRIHTVGVVSEELEKGLMLTLDEDAIYWNFETKKPPQSVCSESCPRGSRRATRKGHP

VCCFDCLPCRDGEISNTTDATKCTVCPDDFWSNLYKDQCIPKEVEFLSYNDPLGISLTTA

SLLGTCFCAFVMVIFVHHHNTPIVRANNSELSFLLLFSLKLCFLCVLLFIGRPQLWTCQL

RHAVFGISFVLCISSILVKTMVVIAVFKSSRPEGKMAMKWFGAAQQRCTILALTAIQVVI

CVVWLSTASPTPHKNNLYIRSIIVYECTIGSVTGFSMLLGYIGLLAAVSFLIAFLARNLP

DNFNEAKFITFSMLIFCAVWITFVPAYVSSPGKYSVAVEIFAILASSFGLLVSIFAPKCY

IILLHPERNNKKAIMGRETH

>OlfCq8

**MWITLLINIYLILKCISAAVVLRAGACQLQGRFRLNGMYQDGDVILGGMFEAHFFTLYPE**

**LTFRTEPAPPYCEI**FSMESFQNVQTMAFAINEINMNPNLLPNIILGYHIHDNCVTFGMAL

RAAMSLVSGIEESFINLNCTGPPPIIGIVGHPSSTPSIAISSVLGLFRVPIVSHYATCSC

LSDRKKYPSFFRTIPSDAFQVRAMIQLISHFGWTWVGLLYSNDDYGTYAAQSFHQEMQLF

GICIAFSEPLRYDSNPRDIQRIMAVIQASTSRVVVVFSPSTLVIPLINEVVLQNMTGRQW

IASESWATSPVFYTPHFLPFLGGTLGIAIRRGEIEGLREFLLQLRPQNDPRNNMPKIFWE

NMFGCSFETGAQAKNVCTGQEDLSTTNTSYTDVSELRAANNVYKAVYALAHALHDLMKCE

EGKGPFSGNSCADITNLKPWQLVHYLQKVNFSTRFGDHVSFNKNGDALAIYDVMNWQPGS

DRSIRIHTVGVVREEPEKGLMLTLDEDAIYWNFETKKPPQSVCSESCPRGSRQATRKGHP

VCCFDCLPCRDGEISNTTDPTKCTVCPDDFWSNLYKDQCIPKEVEFLSYYDPLGISLTTA

SLLGTCFCAFVMVIFVHHHNTPIVRANNSELSFLLLFSLKLCFLCVLLFIGRPQLWTCQL

RHAVFGISFVLCISSILVKTMVVIAVFKSSRPEGKKAMKWFGAAQQRCTILVLTAIQVVI

CAVWLSTASPTPHKNNLYIRSIIVYECTIGSVTGFSMLLGYIGLLAAVSFLIAFLARNLP

DNFNEAKFITFSMLIFCAVWITFVPAYVSSPGKYSVAVEIFAILASSFGLLVAIFAPKCY

IILLHPERNNKKTIMGRETH

>OlfCq9

**MWAILLFCLFLSCN**FICVTLMVSSGTCQKQGHFTLNGMHQDGDFVIGGLFEIQSYVKVYP

EISFRTQPKLPNCDLFYMTSFQQALTMVFAISEINNNPNLLPNITLGYQIYDNCLRLGVA

FRAATALISGTEETISDLNCKGPPPVIGVIGDPGSTHSIAISSVLGLFRLPMISYYATCS

CLSDKKKYPSFFRTIPSDTFQVRAMVQTMRHFGWTWVGLIYSNNDYGIYAAQSFHKEMQL

FGHCVAFSEILPQDNNPRVIDHIIGVIQASTARVVVVFSASSLLIPLMNKVVLQNLTGRQ

WIASEAWVTSTVFRTPYYQPFLKGTLGIAIRRGEIQGLHSFLLRLHPNSDQRNNIVRIFW

ETMFGCSFETEDKETFGQQIKKVCTGQEDLSITNTPYTDVSGLRAPYNVYKAVYALAHAL

HDLMQCKEKRGPLSEKNCADITDLKPWQLLSYLKKVNFTTGFGDPVSFDNNGDALAIYDV

LNWQPSSEGSIKLHSIGVVNDEVATGMVLRLNKDEIYWNFEAQKAPRSVCSESCPPGTRK

AMRKGFPVCCFDCLTCGDGEISNTTDAIKCTVCPDEFWSNFNKDQCVPKEIEFLSYEDPL

GISLTTASLLGTCFCALVMIIFAFHHNTPIVRANNSELSFLLLLSLKLCFLCVLLFIGQP

QLWTCQLRHAVFGISFVLCISSILVKTMVVIAVFNSSRPEGKGAMQWFGAAQQRCTVLVL

TVLQVVICAVWLSTSSPTPHKNNQYVRSKIVYECAIGSVAGFSLLLGYIGLLAAISFLLA

FLARNLPDNFNEAKFITFSMLIFCAVWIAFVPAYVSSPGKYAVAVEIFAILASSFGVLVA

IFAPKCYIIILHPERNTKKAIMGRETKKY

>OlfCq10

**MWAILLFCLFLSCNYICVTL**MVSSGTCQQQGHFTLNGMHQDGDFVIGGLFEIQRYLKVYP

EISFRTQPNLPNCELFYMTSFQQALTMVFAISEINSNPNLLPNITLGYQIYDNCLRLGVA

FRAATALISGTEETISDLNCKGPPPVIGLIGDPGSTHSIAISSVLGLFRLPMISYYATCS

CLSDKKKYPSFFRTIPSDTFQVRAMVQTLRHFGWTWVGLIYSNNDYGIYAAQSFHQEMQL

FGHCVAFSEILPQDNNPRVIDHIMGVIQASTARVVVVFSASSVLIPLMNEVVLQNLTGRQ

WIASEAWVTSAVFRTPYFQPFLKGTLGIAIRRGEIQGLHSFLLRLHPKSDQRNNIVRIFW

ETMFGCSFETGDEVTFVQQMKKVCTGQEDLSITNTPYTDVSGLRAPYNVYKAVYALAHAL

HDLMQCKEKRGPLSENSCADITDLKPWQLVPYLKKVNFTTGFGDPVSFDNNGDALAIYDV

LNWQPSSEESIKLHTIGVVNDEVATGM APRSVCSESCPPGTRKAMRKGFPVCCFDCLTCG

DGEISNTTDAIKCTVCPDEFWSNFNKDQCVPKEIEFLSYEDPLGISLTTASLLGTCFCAL

VMIIFTFHRNTPIVRANNSELSFLLLLSLKLCFLCVLLFIGQPQLWTCQLRHAVFGISFV

LCISSILVKTMVVIAVFNSSRPEGTGAMKWFGAAQQRCTVLVLTALQVVICAVWLSTSPP

TPHKNSQYVRSKIVYECAIGSVAGFSLLLGYIGLLAAISFLLAFLARNLPDNFNEAKFIT

FSMLIFCAVWIAFVPAYVSSPGKYAVAVEIFAIVASSFGVLVAIFAPKCYIIILHPERNT

KKAIMGRETKNN

>OlfCq11

**MTNTISLFLEWYRLSRRKKKALYICDLNDEGHKQIRG**MWVILHIYFFISCNYMSVALMAS

SGTCQLQGHFTLNGMYQNGDFLIGGLFEIQYLKAFPGLSFRTEPKLPHCENFYMTSFQQA

ITMVFAINEINNNPNLLPNITLGYQMYDNCLRLGVAFRAATALISGTEETLSDLNCKGPP

PVIGVIGDPGSTHSIAISSVLGLFRMPMISYYATCSCLSNRKKYPSFFRTIPSDAFQVRA

MIQILRHFEWTWVGLLYSDDDYGINAALSFHHDVQQFGGCVAFSEILPNDNNQMAVQHIV

RVIQNSTAKVVVVFSTSSYLLPVIDEMLLKNITGRQWIASEAWSTSPVLLNPRLRYVLGG

TLGIAIRRGEIEGLDNFLLRLRPDNFSQNSMMRIFWENMFECNFDTIGGLRTKLCSGQED

LRSKFTPYTDVSELRASYNVYKAVYALAHALHDLMHCEEGRGPFSENRCVDISNLKPWQL

VHYLQRVKFTTGFGDHVSFDKNGDALAIYDVLNWQLSSEESVTVRRIGVVDEGVTTGKVF

TLDENAIYWNFETNKPPRSVCSESCPPGTRQATRKGLPVCCFDCLPCGDGEISNTTDTTE

CIACPNDFWSNPEKDQCIPKEVEFLSYEDPLGISLTTAAMLGTFICALVMTIFAHYRNTP

VVRSNNSELSFLLLLSLKLSFLCVLLFIGQPQLWTCQLRHAVFGISFVLCVSSILVKTMV

VIAVFKSSRPEGKNAMKWFGAAQQRGTVLILTALQVLICAVWLSTASPTPHKNSRYIRSI

IVYECAIGSVAGFSLLLGYIGLLAAVSFLLAFLARNLPDSFNEAKFITFSMLIFCAVWIA

FVPAYVSSPGKYAVAVEIFAILASSFGLLLAIFAPKCYIIILHPERNTKKAIMGKGTKNK

>OlfCq12

**MLTWHRLFIKATPSKVFIINNIHRGELKRNVE**MWIIAKICLYLSFSCISVASIFRSGPCQ

LQGQFRLNGMFQEGDLILGGLFEVHFLTVFPEQSFRTEPEPPYCEQFDMASFQQAQTMVF

AIDEINRNPNLLPNITLGYYLYDNCVKLSVAFRAAMALVSGTEESFSSLNCTGPPPVIAI

VGDPGSTHSIAISSILGLFRVPMVSYFATCSCLSDRKTYPSFFRTIPSDAFQVRAMVQIL

KRFGWTWVGLLYSDDDYGIYAAQSFQKEMQLFGACVGFSEMLPRDNNHEDIQSIVEVIQT

STARVVVVFSTEAYLLPLMDEVALKNVTGRQWIASEAWATSPVFHTQHLLPFLGGTLGIA

IRRGEIQGLKEFLYYLHPDSNTRNNIVRIFWENIFGCSFEIGGRERKVCTGQEDLKSTNT

AYTDVSGLRASYNAYKAVYALAHALHDLMQCEDGRGPFSGNKCADQINLQPWQVVHYLQK

VNFTTGFGDHVSFDENGDALAIYDVMNWQPSSDGAISVSTVGVVNEGASMKMVLTLKENS

IFWNFKNRKPPQSVCSESCPPGTRQVRRKGLPVCCFDCLPCADGEISNTTDAIECKICPD

ELWPNPNKDQCVPKEEDFLSFEDPLGISLTTASLLGTCFCALVMVIFSHHRNTPVVRANN

SELSFLLLLSLKLCFLCVLLFIGRPKLWTCQLRHAAFGISFVLCVSSILVKTMVVIAVFK

SSRPESKSAMKWFGVAQQRGTVMALTTLQIVICTVWLSMASPKPYKNSLYISSKVVYECD

IGSVVGFSLLLGYIGLLAAVSFLFAFLARNLPDNFNEAKFITFSMLIFCAVWITFVPAYV

SSPGKYAVAVEIFAILASSFGLLVAIFAPKCYMILFHPERNTKKSIMGRATQIK

>OlfCq13

MWSPLIICLYLSYNFKSAASILMGSCQLQGHFGLNGMYQAGDVILGGLFEVHLLAVFPEL

SFRSAPEPPYCEQFDMASFQQAQTMVFAIDEINKNPKLLPNITLGYHLYDNCVMLGMAFR

AAISLVSGTEELFSNLNCTGSPPVIGIVGDSNSTPSIAISSVLGLFRVPIVSYFATCSCL

SNRKQYPSFFRTIPSDAFQVRAMVNILKHFGWTWVGLIYSDDDYGNYAAQSFLQDIQIYG

GCVAFTEILPLNNNRKYIEHVVGVIQASTARVVVVFSTSTYVLPLMDEVVLQNVTNRQWI

ASEAWATSPMFHTQRLLPFLGGTLGIAIRRGEIQGLHEFLLHLRPKNDQHNNMVRIFWEK

MFGCRFNPGGKGDKQCSGQEDLSSIDTAYTDVKELRASYNVYKTVYALAHALHDLTECDE

ERGPFSENRCADITNLQPWQLVHYLQKVNFTTGFGDHVSFDKNGDVLAIYDVMNWHPSSD

GSISVRTVGVVNEGAASGKVLTLDEDAIYWNFKIKKAPQSVCSESCPPGTRQATKKGLPV

CCFDCLPCGDGEISNVTNAIECTVCPDEFWSNKDKDQCVPKEVEFLSYEDPLGISLTTAC

LLGTCFCALVMIIFCQHRNTPIVRANNSELSFLLLLSLKLCFLCVLLFIGRPQLWTCQLR

HAVFGISFVLCVSSILVKTMVVIAVFKSSRPEGKGSMKWFGTTQQRCTVLILTALQVVIC

IVWLSNSSPAPHKNSQHISSKIVYECAIGSLAGFSLLLGYIGLLAAVSFLLAFLARNLPD

NFNEAKFITFSMLIFCAVWIAFVPAYVTSPGKYAVAVEIFAILASSFGLLGAIFAPKCYI

ILLHPEKNTKKAIMGRQITK

>OlfCq14

MWIILNIFLCLSKWISADSVLRLDTCQLQGHFMLNGVYKHGDVILGGLFDVHLLTVFPQL

SFRVQPKPPYCEKQVMENFKSSQTMAFTIDEINNNPNLLPNITLGYHLYDNCVRLAMAFR

AAMSLASGTEESFSNLNCTGPPPVIGIVGDPSSTPSIAISSILGLFHVPIVSHYATCSCL

SDKKKYPSFFRTIPSDAFQVQAMVQVLRHFGWTWVGLLYSDDDYGTYAAQSFQKEVQLFG

GCIAFSTILPQNNPREIQNIMGVIRASTARVIVAISTSSYLLPLMDEVVLQNLTGRQWIA

SEAWATTPAFRTPRFLTILRGTIGIAIRRGEIQGLLEYLLRLRPSSDPRNNLVRIFWENM

FGCSFASQTVGEQVKKLCTGQEDLSITNTAYTDISGLRGPYNVYKAVYALAHALHGLMQC

EEGRGPFSGNSCADITNLKPWQLIHYLQKVNFITGFGDRVSFDKNGDALPIYDVMNWQPS

SDGSIRVQTVGVVNKGVTSGMVLNLVEDAIYWNFETKKPPQSMCSESCPLGTRKARRKGL

PVCCYDCLPCGDGEISNRTDATECLVCPYEFWSNKEKDYCVPKQVEFLSYEDPLGISLTT

ASLLGICFCALVMVIFSHHHNTPIVRANNSELSFLLLVSLKLCFLCVLLFIGQPQLWTCQ

LRYAVFGISFVLSVCSILVKTMVVIAVFKSSRPEGKDAMKWFGLLQQRCTILVLTTIQVV

ICTVWISNASPTPHKNHQYIRSKIVFECAIGSVAGFSLLLGYIGLLAAISFLLAFLARNL

PDNFNEAKFITFSMLIFCAVWIAFVPAYVSSPGKYAVAVEIFAILASSFGLLMAIFTPKC

YIILLHPERNTKKAIMGR

>OlfCq15p

GAHIAIRRGEILGLQEFLLYSHASINPKNNMLKIF*ENMFRCSFETRGKDTIGEQEKSIC

TGHENLSTTKTPYTDVLGLRASYNVYKTVYALAHALHDLMEFEEGRGPS

>OlfCq16

MWTTLYIFLLFRCFSSESALRSGSCQLQGRFKLEGMYQDGDFILGGLFHVHFFTVFPELS

FQTEPEPPYCEKFNIEIFQQAQTMAFAVDEINRNPNLLPNITLGYHLYDNCVRLGIAFRA

AISLASGTEESFSNLNCTGPPPVIGIVGDPSSTPSIAISNILGLFRVPIVSHYATCSCLS

DRKKYPSFFRTIPSDAFQVRAMIQILRYFGWTWVGLIYSDDDYGIYAAQSFQQEMLLFGY

CVAFSAILPHDNNHRDIQRITAIIQASTARVVVVFSTSSFLIPLMEEVVVQNMTGRQWIA

SEAWTTSPVYHTPRFLPILGGTLGIAIRRGEIEGLHDFLLRLIPSNDKKNSIIRIFWENI

FGCSFEKWGTETFGEQVKNICTGQEDLSTTDTPYTDVSGLRAAYNVYKAVYALAHGLHDL

MQCEEGRGPFNGNSCAETTNLKPWQLVHYLQNVNFTTGFGDQVSFDKNGDALPIYDVLNW

HPSTDGSIKLHTVGLVNKGAAMEMVLTLDDDAIYWNFETRKPPQSVCSESCPPGTRQARR

KGLPVCCFDCLPCGEGEISNATGAIECTVCPDEFWSNPEKDQCVPKEVEFLSYEDPLGIS

LTTASLLGTCFCALVMIIFALHRNTPIVRANNSELSFLLLLSLKMCFLCVLLFIGRPQLW

TCQLRHAVFGISFVLCISSILVKTMVVIAVFKASRPEGKGAMKWFGATQQRCTVLVLTAL

QVVICVVWLLTASPTPHKNNQYIRSKIVYECAIGSVAGFSLLLGYIGLLAAISFLLAFLA

RNLPDNFNEAKFITFSMLIFCAVWIAFVPAYVSSPGKYAVAVEIFAILASSFGLLVAIFA

PKCYIILLHPERNTRNAIMGREMQNK

>OlfCq17

**MCITLYLSLCLCFKHIYADSI**HRLRSCQLQGRFKLNGMYQDGDFILGGLFHVHFFTVFPE

LSFRMEPQQPYCEKFNMEGLQHAQTMAFAINEINKNPTLLPNITLGYHLYDNCVMLRMAF

RAAMSLASGTEDSYSNLNCTGPPPVIGIVGDASSTPSIAISSVLGLFRVPIVSHYATCSC

LSDRKKYPSFFRTIPSDAFQVRAMVQILKHFKWTWVGLLYSGDDYGVYAAQMFHKEMQLS

GHCVAFSEILPNDHNPKDIQRIIRVIQGSTARVVVVFALSSFLIPLMDEVVLQNMTGCQW

IASEAWATSLEYHTPRFLPFLGGTLGIAIRRGEIEGLHEFLLRIRPSNDTSQNIVRIFWE

NLFGCRFETGGLKTGREQEKMECTGQEDLSTTNTPYTDVSGLRASYNVYKAVYALAHGLH

DLMQCEEGRGPFIGNSCADITSLKPWQLVHYLQNVNFTTGFGDHVTFDKNGDALAIYDVL

NWQPSSDGSITVHKIGVVYEGATTGMMLTLDADAIYWNFETKKPPLSVCSESCPPGTRQA

TRKGLPLCCFDCLPCGDGEFSNTRDAVECMMCPDEFWSSPDKDQCVPKEVEFLSYEDPLG

ISLTTASLLGTCFCALVMIIFALHHNTPIVRANNSELSFLLLLSLKLCFLCVLLFIGRPQ

LWTCQLRHAVFGISFVLCVSSILVKTMVVIAVFKSSRPEGKGAMKWFGASQQRCTVLVLT

ALQVVICAVWLSTASPTPHKNNHYFRSIIVYECAIGSVAGFSLLLGYIGLLAAISFLLAF

LARNLPDNFNEAKFITFSMLIFCAVWIAFVPAYVSSPGKYAVAVEIFAILASSFALLLAI

FAPKCYIILLHPERNTKNAIMGRETQNK

>OlfCq18

MWFTLYIYICLSLNCISATLILKPSMCLLQRSFKLNGMYQDGDFILGGLFEVHFFTVFPD

LTFTTNPKSPYCEIFNMEGFQHAQTMAFAIEEINKNPKLLPNITLGYHLYDNCVMLGMAF

RAAISLTSGTEESFLNLNCSGPSPVIGIVGDPSSTPSIAISSVLGLFRVPIVSHYATCSC

LSNRKKYPSFFRTIPSDAFQVRAIVQVLKHFKWTWVGLLYSDDDYGVYAAQSFQQEMRQF

GLCAAFSEFLPHDNNPRTIQHIMGVIQGSTAKAVVVFAPSSFLIPLMNEVVLQNMTGRQW

IASEAWATSLESHIPSFQPFLRGTIGIAIRRGEIQRLHDFLLRIRPSNDPKNYMLRIFWE

NMFGCSFGKGDTEGEQVIKVCTGQEDLSTTNTPYTDVSGLRAAYNVYKAVYALAHALHDL

MECEEGKGPFSKNSCADKTNLKPWQVVHYLQNVNFTTGFGDYVSFDKNGDALAIYDVLNW

QPSSDESIRIYTVGVVKEGTETGMVLTLDEDAIFWNFETRKPPRSVCSESCPPGTRRATR

KGLPVCCFDCLPCGDGEISNTTNAVECFLCPDEFWSNQYNNHCVPKEVEFLSYEDPLGIS

LTTASLLGTCFCALVIFVFALHRNTPIVRANNSELSFLLLLSLKLCFLCVLLFIGRPQLW

TCQLRHVVFGISFVLCMSSILVKTMVVIAVFKSSRPEGKGAIKWFGAVQQRSTVVVLTVL

QVLICAVWLSTASPTPYKNNQYIRSKIVYECAIGSVVGFSMLLSYIGFLAAASFLLAFLA

RNLPDNFNEAKFITFSMLIFSAVWIAFVPAYVSSPGKYTVAVEIFAILASTFGLLIAIFA

PKCYIILLHPERNTKNVIMGRETQNK

>OlfCq19

MWVILNISIYLIFNFIMSASILGSNTCQLQGHFRLNEMYHDGDVILGGLFEVDFLTVFPD

LDFKTEPEPPYCVQFDMESFQQAQTMAFAIDEINKNPNLLPNITLGYHLFDNCVMLGMAF

RAAISMASGTEEFFNNINCTGPPPVIGIVGDSSSTVSIAISSILGLFRVPIVSHYATCSC

LSDRKKYPSFFRTIPSDAFQVRAMVQILKYFRWTWVGLIYSDDDYGVYAAQSFQQEMQLF

GGCVAFSEVLPHDNNLEDIKHITKMIQASTARVIVVFSTPSFLIPLIDELVLQNMTDRQW

IASEAWATSFVHHSPRLLPFLKGTLGIAIRRGEIQGLYEFLLRLQPTNDPKNNMIRIFWE

IMFGCNFETGGKKNSGDQVINICTGQEDLRTTKTPYTEVSELRASYNVYKAVYALAHALN

DLMQCEDGRGPFSVNSCADITNLKPWQLVHYLQNVNFTTDFGDHVSFDKNGDALAIYDVL

NWQPSSDGSIRAQTVGVVNEEVATGMVLTLDEDALYWNFESKKPPSSVCSESCAPGTRLA

TIKGLPVCCFDCLPCGDGEISNITGAVECTMCPDEFWSNLDKDQCIPKEVEFLSYEDPLG

ISLTTASLLGTCFCVLVMFVFAVHRNTPIVRANNSELSFLLLLSLKMCFLCALLFIGQPQ

LWTCQLRHVVFGISFVLCISSILVKTMVVIAVFKSSRPESKGAMKWFGAVQQRCTVLVLT

AIQVVICAVWLSTASPAPHKNNQYIRSKIVYECAIGSVAGFSMLLAYIGLLAAVSFLLAF

LARNLPDNFNEAKFITFSMLIFCAVWIAFVPAYVSSPGKYAVAVEIFAILASSFGLLLAI

FAPKCYIILLHPERNTKKAIMGKEAQNT

>OlfCq20

MWITLCIYLYLSLNYICAHSILRTSDSCRLQRRFHLNGMYKDGDVILGGLFQVHFFTVFP

EQSFRTEPEPPFCEKFDMESFQQARTMAFAIEEINKNPNLLPNITLGFHLYDNCVRLGMA

FRAAISLASGTEESFQNLNCTGPPPVIGIVGDPSSTPSIAISSLLRLFRVPIVSYYATCS

CLSDRKKYPSFFRTIPSDAFQVRAMVQILRHFGWTWVGLIYSDDDYGVYAAQSFHLEMQM

FGHCVAFSEILPHNNNQRDIQRIMGVIQASTARVVVVFSTSSFLLPLIDEVASQNMTGRQ

WIASEAWATSPVYHSPRLLPFLGGTLGIAVRHGEIPGLHDFLLHLGPGNESRNNMLRIFW

ENMFGCSFEPGVKDTNSEIKLCTGQEDLSTTNTPYTDVSGLRAAYNVYKAVYALAHGLHD

LMQCEKGRGPFRGESCADITNLKPWQLVHYLQKVNFTTGFGDHVSFDENGDALAIYDVLN

WQPSSDGSIQIFTVGVVKERTETGMVLTIDEDAIYWNFETKKSPQSVCSESCSPGTRRAT

RKGLPVCCFDCLPCRDGEISNTTDAIECMLCPDEFWSSPDKDQCVPKEVEFLSYEDPLGI

SLTTASLLGSCFCVLVMIIFGLHRNTPIVRANNSELSFMLLLSLKMCFLCVLLFIGRPQL

WTCQLRHAVFGISFVLCISSILVKTMVVIAVFKSSRPEGKGAMKWFGAAQQRCTVLVLTV

LQVVICSVWLSTASPTPYKNNQYIRSKIVYECAIGSVAGFSLLLGYIGLLAAVSFLLAFL

ARNLPDNFNEAKFITFSMLIFCAVWIAFVPAYVSSPGKYAVAVEIFAILASSFGLLVAIF

APKCYIILLHPERNTKNAIMGRETQKR

>OlfCq21

MWISLNIFLYLSLNCIFAASVVNPGTCQLQGHFKLNGMYQDGDFILGGLFEVHFFTLFPK

LSFKSEPEPPYCEKFDMESFQQAQTMVFAIDEINKNPNLLPNITLGYHLYDNCVMLGMAF

RAAISLASGTEEYFSNLNCTGPPPVIGVVGDPGSTPSIAISSVLGLFRVPIVSHYATCSC

LSDRKKYPSFFRTIPSDAFQVRAMVRILSHFGWTWVGLIYSDDDYGIYASVSFQQEMQQF

GSCISFSEILPHDNNPKDIQRIMEVIKASTASVVVVFSTSSYLMPLIDEVVLQNLTGRQW

IASEAWATSPVFHTPRYLPFLGGTLGIAIRRGEILGLQEFLLHIHASNNPKDNMLKIFWE

NMFRCSFETRSKDTIGEQEKSICTGHEDLSTTKTPYTDVSGLRASYNVYKAVYALAHALH

DLMECEEGRGPFDGNSCGNITNLKPWQLVHYLQKVNFKTGFGDHVSFDENGDALAIYDVL

NWHPSSDGSIRLHTVGVVNEGAGTGKVLSLDESALYWNFQTKNPPRSVCSASCPPGTRQA

MRKGLPICCFDCLPCGDGEISNATDSTECVTCPDEFWSNFDKNQCVPKEVEFLSYEDPLG

ISLTTASLLGTCFCALVMIIFALHLNTPIVRANNSELSFLLLLSLKLCFLCVLLFIGQPQ

LWTCQLRHVVFGISFVLCISSILVKTMVVIAVFKSSRPEGKGTMKWFGAAQQRCTVLVLT

ALQVVICAVWLSNASPTPIKNNQYIRSKIVYECAIGSVAGFSLLLGYIGLLAAISFLLAF

LARNLPDNFNEAKFITFSMLIFCAVWIAFVPAYVSSPGKYAVAVEIFAILASSFGLLVAI

FAPKCYIILLHPERNTKKAIIRRETKKR

>OlfCr1

MSIHVLSWLTLSVPVLLLWPVCGSGTEVTPLETCVYLITPQYSDELGTYQDGDVIIGGLI

NLHNLAATPDLSFTRKPGLAQCLEFQERTYRWFQAIVFTVEEINQNPSLLPGVKLGYHIM

DSCSRYPHSLTAAMSMISGGNKTCGPTKPAKLLIGDSSSTQSILLSTTLVPLKIPMISYL

AGCPCLSDRQKYPNFFRTIPSDFYQARTMVQIAKRFGWTWIGAVIADSDNGHGTLQALEE

EIKGTGICLAFYHTLYRERLQKDVAFAARTVQASSARVILVLAWYTDVEAFLLELMRRNV

TDRLFLASEIWSTSYNIMANPQLYTISKGTLGVALRSAPIPGFSAHLQQLHPSHYPEDKF

LRTLWENTFGCSPTISSSKIIQNSLPPCSGRESLEGHIEFTDTSHLTVSYNIYLAVYAAA

HALHSLLECNSQNHTNSKIKPKCSSPDNITPAQLLQHVKDVHYTTQLGEEFYFLEGGIPP

VYDLVNWQIAPDGSLQYAFIGHVDGNQLSINDSAITWPGDSGKVPISVCTSECPPGTRKA

IKKGLPVCCFDCLPCTEGEISNSTGSIKCYRCPKEFWSNSYKNECVARETEFLSVKETMG

ITLMSVAVSGAVMTTTVAVIFLYHRNTPIVKANNSELSFLLLLSLKLCFLCALVFVGQPS

LWSCRIQQAAFGISFVLCISCILVKTFVVLIAFHSTRPESSALIKWFGLGKQRGIVLVFT

CVQVVICAIWLCVSPPLPYQNFGMHRSKVILECTIGSVVGFTCVLGYIGFLATVCFLLAF

FARKLPDNFNEAKFITFSMLIFCAVWVAFVPAYVSSPGKYSVVVEVFAILASSFGLLFCI

FVPKCYIILLKPENNTKKFLMGKE

>OlfCs1

**MMGFIIGLWLVGFIVG**MVRVCNANPTCSLKKSFVSESLYKDGDVIIGGLFPVHVQSPVPD

PAFTQQQHGSNCQGIDLRSYRWLRAMIFTVDEINRDPVLLPNVTLGYLVADTCLAESTTL

SAALALVTGQETTVFSTECNGAPMVPVIIGDARSSASVVVADTLGVFDIPMVSYFASCAC

LSDKHRFHTFLRTVPSDAFQAKAMARLLHLLDWNWVGVVAGDDEYGKSGSHLLLKELEGS

GVCVDYVEVIPKSHSQSRIKQIMERIQSSTASVVVTFAIGPDLEVIFKEMVVQNVTNRQW

IATEAWSTTVQYSDPESIPLLAGTIGFALRRAEIKGLGAFLTQLNPVKQSNEPFVKDVWE

EIFRCSLAHDRQPSFKRPKCTGSESVEKHGRIYTDVSQLRVTYNVYKAVYAIAYALHNMI

ACLPGRGPFENGQCPDATQVKPRQLLHYLNAVNFTTPVGELVYFENNGEPSASYDIMNWY

VNKSGEVNFVQVGQYDAAKGPGQELNINIEKVIWGGGWGNQVPVSVCSVSCLPGTRKVVQ

KRKPICCFDCIPCAAGEISNMSDSTECMRCPEKFWSNTEKTKCIPKVVEFLSLQDTMGIV

LTVLSVTGATLTTTVLATFFHHRDTPLVRAYNSELSFLLLMSLTLCFLCALVFIGRPAAW

NCMLRHTLFGVSFVTCIGCILSKTVVVLVAFRATRPGSHLMQYFGPIQQRAAIFICTLVQ

VVICLLWLLLDPPQPTESAGELGARLILQCTVGSVVGFVFVLGYIGMLAVVCFLLAFFAR

KLPDNFNEAKFITFSMFIFCAVWAAFVPAYISSPGKYTVAVEIFAILASSYGLLICIFTP

KCYIILLKPEKNTKKTMMAR

>OlfCs2

MQLWIVGLIGEIIRLCSTEQSCGLQGRVISESLYKEGDVIIGGLFPVYNEAPVPNHAFTQ

IQNRSRCQGVDLRSYRWLKTMLFTVEEINRDPFLLPNIILGYLVADTCLAESTTLSAALA

IMTGQEETVSDTECIVAPRVPVIVGDARSSASIGVADTLGVFDIPMVSYFASCACLGDNH

RFHTFLRTVPSDAFQAKAMARLLHLLDWTWVGVVAGDDEYGKSGVQLLLKELEHVGVCVD

YLEFIPKSHSQRRIRRTVETIQSSTAHVVVTFAIAPDIEVLFKEVVVQNVTNRQWIATEA

WSTSVQFLDPASIPLLAGTLGFALHRADIEGLGAYLSQLNPAKQSNEPFVKDVWEEIFGC

SLAQDWQPSFKRPKCTGSENVEIHGGIYTDVSQLRVTYNVYKAVYAIAYAIHNMIACQPG

RGPFENGQCPDVNQIKPRQLLHYLNAVNFTTPVGELVYFEDNGEPSASYDIMNWHVDESG

AVNFVQVGQFDAANGPGQELNINIKKVVWGGGWSDQVPVSVCSVSCLPGTRKSVQKGKAI

CCFDCLPCAAGEISNLTDSTKCIRCPEKFWSNVERTKCVPKIVEFLSLQDTMGIVLTVLS

VTGATLTTTVLATFFHHRDTPLVRANNSELSFMLLVSLTLCFLCALVFIGRPATWNCMLR

HTLFGVSFVICIACILSKTVVVLVAFQATLPGSNLMQYFGPIQQRAGILVCTMVQVVICL

LWLLLAPPLPTERAGGEFGARVILQCTVGSVVGFALVLGYIGLLAVVCFLLAFFARKLPD

SFNEAKFITFSMLIFCTVWIVFVPAYVSSPGKYTVAVEIFAILASSYGLLLCIFTPKCYI

ILLKPEKNTKKNMMAK

>OlfCt1

MAFLRLYLFLTVLLFSFFISSQLPQICRLLRRTDDLPVLVSEGDIMIGALFPLHDTILES

PPSFTTEPHPTQCSGFNFRTFRWMQTLIFAIEEINRDKQLLGNLTLGYKIYDSCSTHFHA

LRTALTLMNGNEEIAGISECRGGVPVVIGDGGSTLSIVVAQFLGVFHVPQISYFSSCACL

SNKLDFPAFLRTIPSDFFQVDALVQLVQHFGWSWIGTFAGDDAYGRGGAQIFNEKVTKLG

ACIAFYEIIPKNHQQTEMSRIVERILESRSHVILVFALEQDARALFLEVLHHNLTGIQWL

ASEAWITAAILSTPEFHSVLQGSLGYAIRRADIPGLQPFLLRLHPSKYPQDPFVEQFWEE

MFKCSLGIINRSSSIRPPCDGSEVLVNINNIYSDVSQLRISYNVYKAVYAIAHALDLMLH

CEPGKGPFAGGLCPNFSSMHPWQLLDYLKHVHFTNDFGEDTKFDINGDPVAMYDLINWQL

SGKQEMQYVTVGKYDETMKPKLVIEEKNIIWSGNQKQVPLSVCSTSCPPGTRQATRPNFP

ICCFDCIVCAAGEISNHTDALECVKCSPEFWSNTRRDTCIPKVVEFLSYNDTMGITLLSV

ALLGSCFTLAVTLIFALKRHTPLVRANNSEISFLILSSLLLCFLCALAFIGRPTTWSCGL

RHTAFGIAFSLCLSCVLGKTLVVLMAFKASLPGSNVMRWFGPLQQRGIIFMCTAVQVVIC

CTWLCVAPPIPHMLMTHETARIILLCDVGSSFAFSLVLGYIGLLAAVCFLLAFFARKLPD

NFNEAKFITFSMLIFCAVWIAFVPAYISSPGKYTVAVEIFAILASSYGLLLCIFTPKCYI

ILLKPEKNTKRYVMSKSNS

>OlfCu1

MPVIELVSLGLALLLNFSVVLCTSLDKSCWTLGDFNSPVLEQDGDIVIGGLFPMHNIAPE

TDYNFSDLPHYQDCSRFDFRAFRWVQTMVFAIEEINSNSSLLPGVALGYRILDSCDHVHT

SLRSALFLLNGTFGQTQTDAVGTANCLSTAPVPAVIGLASSSPTRAVAQTLGPFGIPLIS

YFATCTCLTDKKEYPSFLRTVPSDRFQVQGLVQLVSHFGWRWVGTVGTDDDYSHYGIQAF

TEQLERLGSCIAFHQTIPKSPSQAQIRSILNSLESSTAQVIIAFATEGELLELLVEVARR

NLTRLQWVASEAWVTAKLLTIPELHPVLIGTVGFSFRGTAIPGLAEFLFRVRPSSRPESA

FTNMFWEELFGCRLGYEDSNDSVLPLCTGSENLLETESSYTDVSRVRISYNVYKAVYAIA

HALHQLLQCGSKEQGSDHSCNTESKFSPLQLLRYLKKVHFTNQFNEKVYFDTNGEPVPLY

DIINWQKNARGTINFQLVGTYDGSAPHGQQLKIEEGLIRWTGGQTKVPVSLCSPPCPPGT

RQATRPGQPVCCFDCLPCAEGEISNISGATECLKCPEYYWPNKERVSCVAGIEEFLSYHD

VMGIILISLSLFGVAVATVVMVIFFIFRSTPIVKANNSEMSFLLLLSLKLCFLCSLVFVG

RPSPLTCRARQAAFGISFVLCISCILVKTIVVLLAFRSTVPGSISLKVFGPPQQRVFIFC

CTTGQVILCACWLALAPPYPYKNTSYQDGRIILECKDILPLGFYLVLGYIGLLSCMCFVL

AFLGRKLPDTFNEAKLITFSMLIFCAVWISFIPAYNSSPGKYTVAVEIFAILASTFGLLF

SIFIPKCYVILLRPDLNTKKGMTGKLSK

>OlfCv1

MIHIFLCIFHLSSAAMTSRCIPRGNFDLPVFMTSGDFTIGGIFPLHYRVELPPTDYMKKP

LTAMCRGFDPMAFHWALTMGLAVEEINNRKDLLPEHTLAYRIFDSCATPVMAQKAVLAAL

NGQDVVQSFMCSGASPLLGLIGESGSSQSIVVSRTLEPFRIPMISYFSTCSCLSDRKQFP

TFFRVVPSDDYQVKAIAQLLKRFDWTWIGVVTEDHDYGRFALQGLKREIENTKICLAYHE

MIPKDYTQERVLKILKVMKESTAKVVVVFSGEGEFYPFLTEFVAQNITGIQWIASEAWVT

ASMLAETYSFLDGTIGFAIRQGHVPGLQDYIRTVTPERYPSIPQVQELWEALYGCSPSTS

TLSSHLPSCTGKEILRKEYSAYMNTSSPRVTYNVYKAVYAFAHSLHNLIECRNGHGPFEN

LSCANLNNVFPWQLQHYLQEISFSISGEEVNFDIKGDAIPSYDLINWQRSASGDMQFIKV

GLYDGAQHSGKELVIEKQAIAWSNQQTKVPVSVCSNGCTPGYRKAVRHGQSLCCFDCVPC

DIGKISNQTDSVDCLSCPEDYWSNVNRTECIQKVIEFLSHDAMGMTLTVIAVAGAFLTIT

VLGIFLYNKGTPIVRVNNSELSFFILVSLTFCFLCALIFIGEPTSWSCMLRHTAFSITFS

LCISCILGKTLVVLAAFTATRPGNNIMKWLGPTQQRIIIFCCTLVQVLICTVWLVVSPPF

PYRNTKYQQSKIILDCSVGSDLAFWCVLGYIGLLACVCFFLAFLARKLPGNFNEAKYITF

SMIIFCTVWLAFVPAYVSSPGKFTTAVEIFAILASSFGLLFCLFAPKCYIILVKPEKNTK

QHLIGKVTK

>OlfCv2

MIYIFMSIFHLSSASMALICSPHDFDLPVFMTSGDFTIGGIFPLHYRVKLPPTDYMKKPL

TAQCRGFDPRAFRWALSMKLAVEEINNRKDLLNNFTLAYKIFDSCSTPVTAQKAVLAVMN

GQEVVQSSMCSGAGPLIGLIGESGSSQSIVLSRTVQAFQIPMISYFSTCSCLSDRKQFPT

FFRVVPSDDYQVKAIAQLLKRFDWTWIGVVTEDHDYGRFALQGLKREIENTNICLAYHEM

IPKDYTQERVLKILKVMKESTAKVVVVFSVEGEFYPVLREFVAQNITGIQWIASEAWVTA

SMLAETYSFLDGTIGFAIRQGHVPGLQDYIRTVTPEKYPSIPQVQELWEALYGCSPSTST

LSSHLPSCTGKETLRKEYSAYMNTSSPRVTYNVYKAVYAFAHSLHNLIECRNGHGPFENF

SCANLNNVFPWQLKHYLEDVSFSISGQNVNFDNKGDSVPYYDLINWQRSASGDMQFVKVG

LYDGAQHSGKELVIEEQAITWSNQQTKVPVSVCSYRCAPGFRTAACRGQPLCCFDCVPCE

SGKISNQTDSVDCLPCPEDYWSNANGTSCVPKVIEFLSHDAMGMTLTVIAVIGAFLTITV

LVIFLYNKGTPIVRVNNSELSFFILLSLTLCFLCALIFIGEPTSWSCMLRHTAFSITFSL

CISCILGKTLVVLAAFTATRPGNNIMKWLGPTQQRIIIFCCTLVQVLICTVWLVVSPPFP

YRNTKYQQSKIILDCSVGSDLAFWCVLGYIGLLACVCFFLAFLARKLPGNFNEAKYITFS

MIIFCAVWLAFVPAYVSQPGKFTTAVEIFAILASSFGLLLCLFAPKCYIILMKPEKNTKQ

HLMGKVIT

>OlfCv3

**MHSLET**MTILLLWLLVYHLSVLNPAQASKCLLQNEFEPGLMANGDFVIGGIFPLHYNQEM

PDLNFTYKPGPVKCNGFDTRAFRWAITMKLAVEEINKRVDLLPNYTLGYKIFDSCAYPLT

GQRSAVAVLNGPNELESPLCADAAPLLAVIGESGSAQSIVVSRILQPFGIPMISYFSSCA

CLSDRREFPTFFRVIPSDAYQVKAIAKLLRHFNWTWIGVIRGDHEYGRFALQGLLKELEG

TGICVAYQKMIPLLYDRQKALEIIHVMSHSTARVVVVFSAEGELTPFLRDYMEQNVTGIQ

WIASEAWVTSSVFAGSEFDPFLGGTIGFGIRQGQIPRLKEYLTTVNPERYPTNPLVYELW

GALYGCSPSWSNLSSHLPSCTGKETVRLQYSAYLNTSSPRISYNVYKAAYAIAYSLHNLI

YCTPGKGPFSNSTCANAPHIYPWQLQQYLQEVSFTISGEKVNFDMKGDSIPSYDLINWQR

GSAGNIEFINVGMFDGALESGQELVIKEEAIMWPGHQTEVLVSVCSNSCAPGFRKAVRRG

QPLCCFDCVPCDSGKISNETDSLDCIACSEDYWSNADGTVCIPKVVEFLSHDAMGLTLTV

IAVAGACLTLAVFSVFLYYKNTPVVRINNSELSFFILLSLTLCFLCALIFIGEPTSWSCM

LRHTAFSITFSLCISCILGKTLVVLAAFTATRPGNNLMKWLGPTQQRIIIFCCTLIQVLI

CTVWLVASPPFPYRNTKYQQSKIILDCSVGSDLAFWCVLGYIGLLACVCFFLAFLARKLP

GNFNEAKYITFSMIIFCAVWLAFVPAYVSSPGKFTTAVEIFAILASSFGLLLCLFTPKVY

IILVKPEKNTKQHLMGKDK

>OlfCw1

MKTPVLRAIHLVFFLFYPIQSNSEANCKLWKELDLTVVHKEGDVILAGMFPIHSKGIDQE

LNFRNQPDQRKCWGFNMRVFRWSQAMIFFIEEINRNPTLLPNITLGYRLYDTCGLIALSL

RTALSVVSQPMKRSSSEMCSSPSIPIIIGDSGSTLSMAISRLLNLFHIPLVSYFASCACL

SNKHQFPYFFRTIPSDVNQANALARLVKHFGWTWVGTVGADDAYGRTGIDLFTAAVTQLG

VCVSYRIIIPKLPTQQQLQDIVRTIRDSSAHVLVAFAIEEDIKPVVDEIVLQNVTGKQWV

ASEAWVTSTLISTKENFPSLSGTIGFAIRRAEIPGLKHFLDSIQPLADPYNVFAREFWET

QFQCTLNTSLPTSSTMDPVNYSHSCTGMERMQDTQSIFNDVSQLRVTYNMYKAVYTVAHA

LHNLLLCQREHRSALTQQCPDIHNLQPWQVIEVLRKVNYTNMFGDLIYFDENGDPVGSYD

IVNWQKGGDDVPVQYITVGRFDSSLPKGQQLVLNQNKIVWHGGTNKVPVSVCSASCPPGY

RKVRLEGQPVCCYDCMLCAEGSISNTTDQAECLLCPEDFWSNKHRNYCVPKEIEFLSYLE

AFGMVLAAIAILGAVAAISVGVVFFRHRDTPLVRANNSELSFLLLISLTLCFICALTFLG

QPSHWACPLRRISFGLTFALCLSCLLSKTLVVLIAFKSTLPGNNTARWFRPPQQRLGVFI

CSLLQGGVCIAWLTTASPYPVKNTWLYRDRIILECHLGSVVYFCCVLGYIGCLAAFCFIL

AFLARKLPDNFNEAKFITFSMLIFCAVWITFIPAYVSSPGKFTVAVEIFAILASSYGVLL

CIFAPKCYIIIFMPEKNSKKYLMTQK

>OlfCx1

**MCTTGCSHQHCSVST**MAQIYATLLTLCLLLYIRVQCQSNNKYKCIYQGDDDTYSFYQGGD

LVLGGIFPLHSSTIPQFFSFTTKPKLIQYKFFTPRALRWMQTMIFAVREINQRQDLLPNL

SLGYHIRDSGDDIPVSVKRSLLLVNGQPEKGSGQSCEDTRKQPSPVIVGEASSGVSMAVL

RTLGTFQIPLVSYFASCSCLSNKREFPAFMRTMPSDLFQIKALVKLVYYFKWTWLGVIGV

DTDYARFAIQLFLKESEKYNICPAYVHIYPVALTQNTVEELVKILKSSSARVILNFSVDS

YLYGVLKECRRQNVTNLQWIASEAWATSKVLWDDFGDLLKGTLGFAIRRADIPHLGSYLR

SVSSSVAQTSPFFTEFWEETFHCRLNGSLNTHVHEEASYNWPACNGNETLDDVYTLYSDV

SQLRVSYNVYKAVYLIAHALHDMSTCDPGKGPFKNGTCGSLYQILPWQLLYYMKRTNFTT

LGEEVRFDKNGDPIASYDLMNWQRESDGSLQLVRVGIYDASFKDDKDLVIDESVIMWHRG

DKAPESLCSKSCLPGSRKARQKGKPVCCFDCISCAEGEISNQTDSIDCLTCSKDTWPNQS

QDQCIPKTLEFLSFQEPLGIILWVFSALGACAALAVLCVFVMYRTTPVIRGNNIELSFLL

LLFLCACFLIGLTFLGKPTDWLCQIRYPAFGISFTLCISCILAKTVVVLMAFRATIPGNN

VMKWFGPVKQRSSVILCTCVQALICIIWLTTKPPLASYNSKFLSATIIVECSVGSEVGFW

CVLGYIGFLSSLCFFLAFLARKLPDNFNEAKFITFSMLIFFAVWITFIPVYVSSSGKYMV

AVHVFAILASAFGLLMCIFAPKCYVVLLKPERNDKKSMMKK

>OlfCx2

MALLCATLLMVWFLMYLSVQSQYNSKVRCVFQGDDDTNTFYQGGDVVLGGLFPLHFSPIS

SLSSYKTKPTPTTYKFFTPRALRWMQTMIFAVREINQRQDLLPNLSLGYHIRDSGDDIPV

SVKRSLLLVNGQPDKDSGQSCEDTRKQPSPVVVGEASSGISMAVLRSLGCFKIPLVSYFA

SCSCLSNKKEFPAFMRTMPSDLFQIKALAKLVHYFQWTWVGVIGVDTDYARFAIQLFLKE

SQQLNICLAYVQLYPVALNQDSAEELVRMLKSSSATVIISFSVDSYLYGILKECRRQNVT

HLQWIASEAWATSKSLWEEFGDLLKGTLGFAIHRADIPHLGSYLKEIRPQTSHFLSEFWE

ETFRCRLNGSLNTHAHGEQAQNWPACNGSESLDDVYTTYSDVSQLRVSYNVYKAVYLIAH

ALHDMSTCVPGKGPFQNGTCGSLFQIQPWQLLYYMKQTNFTTLGEEIRFDENGDPIASYD

LMNWQRGSDSSLQLVRVGFYDASLEDDKDLVIDESLIMWHRAEKAPESLCSKSCLPGSRK

ARQKGKPVCCFDCISCAEGEISNHTDSIECLTCSEDTWPNQSQDQCIPKTLEFLSFQEPL

GIILWVFSAFGACAALAVLCVFVMYRKTPVVRGNNIELSFLLLLFLCACFLIGLTFLGKP

TDWLCQIRYPAFGISFTLCISCILAKTVVVLMAFRATIPGNNVMKWFGPVKQRSSVILCT

CVQALICIIWLTTKPPLASYNSKFMSETIIVECSVGSEIGFWCVLGYVGLLACLCFLMAF

LARKLPDNFNEAKFITFSMLIFFAVWITFIPVYLSSSGKYMVAVHVFAILASAFGLLLCI

FFPKCYIVLLKPEKNDKKHIMKK

>OlfCx3

MAGVYKVLISFWPLLCIGVQSQYKSKENCVFQGDEDTYSFYQRGDVVLGGIFPLHSSPVS

SLFSFRTKPKPTSYKFFTPRALRWMQTMIFAVREINQRQDLLPNLSLGYHIRDSGDDIPV

SVKRSLLLVNGQPEKGSGQSCEDIQMQPSPVIVGDAASGVSMTVLRTLGSFKIPLVSYFA

SCSCLSNKREFPAFMRTMPSDLFQIKALVKLINYFQWTWVGLIGVNSDYAHFAIQLFLKE

SEQFNICPAYVHFYPVVLMEDALEELVNILKASSATVIINFSGESELHSILKECRRQNVT

HLQWIASEAWATSKSLWEEFGDLLKGTLGFAIHRADIPHLGSYLKEIRPQTSHFLSEFWE

ETFRCRLNGSLNTHAHGEQAQNWPACNGSENLDNVYTTYSDVSQLRVSYNVYKAVYLIAH

ALHDMSTCVPGKGPFQNGTCGSLYQIQPWQLLYYMKQANFTILGEEVRFDENGDPIASYD

LMNWQRGSDSSLQLVRVGFYDASLEDDKDLVVEESVIMWHRAEKAPKSMCSQSCLPGTRK

ARQKGKPVCCFDCISCAEGEISNQTDSIDCLTCSKETWPNQAQDRCIPKTLEFLSFQEPL

GIILWVFSAFGACAALAVLCVFVMYRKTPVVRGNNIELSFLLLLFLCACFLIGLTFLGKP

TDWLCQIRYPAFGISFTLCISCILAKTVVVLMAFRATIPGNNVMKWFGPVKQRSSVILCT

CVQALICIIWLTTKPPLASYNSKFMSETIIVECSVGSEIGFWCVLGYVGLLACLCFLMAF

LARKLPDNFNEAKFITFSMLIFFAVWVTFIPVYVSSSGKYMVAVHVFAILASAFGLLMCI

FAPKCYVVLLKPERNDKKHMMKK

### Sequences used as outgroup for the phylogenetic tree

>Dr_CaSR

MRFHLKFYLHYLVLLGSSCVISTYGPNQRAQKTGDILLGGLFPMHFGVASKDQDLAARPE

STECVRYNFRGFRWLQSMIFAIEEINNSSTLLPNITLGYRIFDTCNTVSKALEASLSFVA

QNKIDSLNLDEFCNCTGNIPSTIAVVGASGSAVSTAVADLLGLFYIPQISYASSSRLLSN

KNQYKSFMRTIPTDEYQAIAMAAIIEHFQWNWVIAIASDDEYGRPGIEKFENEMFHRDIC

IDLNVLISQYVDEAEIRRLADRIQNSSAKVIVVFASGPDIEPLVKEMVRRNITDRVWLAS

EAWASSSLVAKPEYLDVMGGTIGFALRAGHIPGFKDFLQQVHPKKSSHNEFVREFWEETF

NCYLEDSPRNADSENGSTSFRPLCTGEEDIASVETPYLDYTHLRISYNVYVAVYAIAQAL

QDILTCTPGKGLFSNGSCADIRKVEAWQVLKQLRHLNFIDSMGERVRFDNGSELSANYTI

INWHRSPEDGSVVFKEVGYYSIHNKNVAKLSIDKSKILWNGRLTEVPFSNCSVECEPGTR

KGIIDGEPTCCFECTECSDGEYSDHKDASFCVKCPNNSWSNGNHTSCFLKQIEFLSWTEP

FGIALALFAVLGVLLTAFVLGVFVQFRDTPIVKASNRELSFLLLFSLICCFSSSLIFIGE

PQDWTCRVRQPAFGISFVLCISCILVKTNRVLLVFEAKIPTSLHRKWWGLNLQFLLVFLF

TFVQVMICVVWLYNAPPGSYKNYDIDEIIFITCNEGSMMALGFLIGYTCLLAAICFFFAF

KSRKLPENFTEAKFITFSMLIFFIVWISFIPAYFSTYGKFVSAVEVIAILASSFSLLACI

FFNKVYIILLKPSRNTIEEVRCSTAAHAFKAAAKATLRHSSGFRKRSSSVGGSSASSPSS

SISMKTNGNEMESPSTRRHGSKPRVSFGSGTVSLSLSFEEARNSMK

>Dr_T1Ra1

MGTMLSNFVFLCLLRFSDSTGLHLQGDYILSGWFALHNSDSTIPPTPYLNDCKKGLTNKH

GYHLVQAFRYAVDEINNGTQDKQLLPGVTLGYRTYDICSLPASNLATLDLLAQQLHPSAV

DSRAVAIIGPDSSSYAFTPAAALGVFLVPQISYEATNELLSNKLLYPSFFRTIPSDKNQV

NAMIQVLVKFNWTWIALLGSDNSYGIQGMQSLSEQAPLNNICIAYQAQIPAVTDSTKKYM

QDMVKNILKTKVNTIVVFANKRRAAGFFPFVIAQNVTGKVWIGTEDWSVASTVSSIPGIS

MIGTVIGVAVKYTEFDGFDNYERLSVPGLKNPGQFNLSVPCMQNTNLYDIAINGFSLSQY

DITSSFNVYKAVYAVAHALHNVLDCDSGLCQKYDVQPWQVYEQLRQVRFSIRNASFYFDK

NGDPPTGYDIVTWEWTNGLWSFKVVGNYSPSANDLHLDDSQIDWSGQVSPVTNVPESICS

PECPYGFRKLMTGQHKCCFDCMACPAATFLNKTGYTSCQPCPIDDWSEAESEVCLPRAEL

YLSWGAPLTTALIIYLAVTLFVTLGTTLVFLLNLSTPVVKSAGGKTCLLMLVSMIVACCS

TLCHFSRPSRVGCLLKQPLFVISCTVCLACVTVRSFQVVCIFKWSSKLPRSYETWAKNRG

PEMFILITTVVETFISVLRMLLDPPFPSQDYNFYHDSIVLECSKTLSLSAFAELFFVCAL

SLVCFCLSYMGKDLPANYNEAKCITFSLMIYMISWITFFTAYCISRGSFVMALNVGAILL

SVLGILGGYFLPKVYIILIKPQLNTTAHFQNCIQMYTMAKQ

>Dr_T1Rb1

MLPCYSLLLAFINVVSCLESEFSLKGDYLLGGLFPIHEGKPATHLYTFSKSGYQMLQVMR

FAVEEINNSTTLLPDVSLGYEIFDHCSDTKNFPSVLSFISKNGSIKPKVKLNNYEPEVIA

LTGPYGSTTTITIAPLITMDLIPLVNYGASSYSLSNKLMYPSFVRTVPSNKDLIHMIIQI

IQWFGWNWVAFLGDQDDYSEDGLRLFNTFISNSGICLAYQEALSQKTNYSLTFEMIDMLN

VNVIVVFAEQQYASNIIKAAIANDVRDKVWIASETWSMNQQLPREPGIEKIGTVIGITER

FLSLPGFNEFIYKERRSVDDAGHNHGMGEVKSQTCNQDCDCCTLLTAEEIITENPTFTFG

IYSAIYTIAHALHKVLQCDVNVCDKNTTAKPYMVLEQIKKLDFPLNGRQVKYDANGDPTI

SFAIILWHTETDPPHFDKVGMFDTYPEVTFTINNTLLPWHNNVPFSNCSAECKEGFAREH

DRFHTCCFLCKKCPRNSYVDYTRDPYTCFPCAESEWSDEGSIACKNRSVAYLQLTEASSI

TVLFSATCLMTVLIAIFVLFACNYNTPVVRSAGGSMCFLMLACLIMSTLSVFFFFGEPTF

EHCILRNVIFAYFFSVFLSCMAVRSFQIVCIFKMAAKFPSMHSLWVKHNGQWLFVGFFSI

INIVSCMLYMTVSPPKPFRDSVTFKDQLILSCEIGNTVTISMVMFIAWFLGFLCLVFSYM

GRDLPKNYNEAKSITFSLILYYLSWIVYFTTYLMLKSKYIQLVNAMTELSSIYGILFSYF

IPKSYIIIFQPHKNTPAYFQTSIQSYTQTISRT

>Dr_T1Rb2

MLLDSNYIFLLGFINSLFFNFCSLASDFSLEGDFLLGGLFPLHEIDQVTPVFTPETTECF

RYTASPSGFQMLQVMRFAVEEINNSSTLLPNVSLGYELFDHCSNTRNFGSLLSFISKNGS

IKPKVKRINYQSDVIALTGPYGSTRTISIAPLITMDLIPLVNYGASSSVLSNKLQYPSFV

RTVPSNKDMINMIIHMIRWFGWNWVAFLGSKDAYSTDGLNLFNTYINNTGICVAYQERLN

LDANYSQTLKKIDMLNINVIVVFAVPQYAVNIINTAIADNIRDKVWIASETWSMNQQLPR

EPGVEKIGTVIGITDRLLTLPGFNEFILKDMGTANVNASKFQTNTCNQNCDYCPSLTAED

IINENPSFSFAIYAAMYTIAHALHKVLQCDTKGCPKNTPFEPYMLLGEMKKLDFPLNGRQ

VKYDKNYDPPISYAVVLWHTDENSPQFEMVGTYDTYPKTVFTIDNSRFPWRNDSIPFSNC

SVECKPGFARQPEGFHSCCFTCKKCPRNSYVDYSQDPYTCFPCAVSDWSDEGSTACKTRA

VVYLEFTEITSIAVMISVSFLIILLIGIFGLFAYNFNTPVVRSAGGGMCLLMLLCLTISS

ISVFFFFGKPSSVHCLVRNAIFAFFFTVCLSCLTVRSFQIICVFKMAAQFPRLHSLWVKH

NGQWLFIAFSSFIHLISCVIWTTVSPDIPIADSWTFKDQTLLMCEMVNTITFTVVLFISW

FLGFLCLVFSYMGRDLPKNYNEAKSITFSLILYYLSWIVYFTAYLSIKSKYIMLLNAMAQ

ISSIYGILFSYFIPKSYIMIFQPQKNTAAYFQTSIQNYTQTISRS

>Dr_T1Rc1

MKNKWTFLVLCGILGSGLGDNPSWFNNITTNFFKSPGDILIGGLFPINQLTSELSQRVKP

DDLQCDSISTYGLSLSLVMKFTVDEINSKKHILPGITLGFESYDTCMQPAVIMKPVLQLL

TQESTDELDIYCNYTNYKPRVMAIIGPDSSDVVPDAGKLIGFFLMPMISYGATSDEFSNK

QTYPSFMRTVASDQWQVVAMIQLLKQFGWNWVSVIGSDEEYGQMGQQQFSSMANDESICV

AYQGLIPVYSDPGPTIQDMLNRIVDAKVGVVVVFSIPIPAKAFFKEVIKRNITAVWVAST

AWSLNDGVSTLPGITSIGTVLAFADITRPLDLFTPYIRELFTKIEGMAIPQQLDADISPL

DNPCPRCSYVSQANVSMVEVDLVQRSAFSVYAAVYCAAYALHDLLGCNATSCTRNPKRDN

VYPWQLLRKLQKLSLDLEGVNIQFDDEGNPNFGYDFMQWIFNDTTVTFDVIGYFYQNLTI

ESNAIKWHTKNGEVPMSTCSSDCGIGQVRRVKGFHSCCFDCIDCLEGTFLNNTDDIQCKS

CPNGQWSTLRSTSCVYPIYTYLDWTNYESIGVILGGIVVLASHVWVGALFFKHRGTPLVK

TAGGPLCGLTLLSLAGGCMSLVLFLGQPGDTVCRLQEPLNAFFPTVALSVILSSSLQIVC

VTEFLEQSSEHLENLRGRGSWFVILGCCGLQAGLCGWYGLEGPSLTQYVASLDVTYVKTF

LRCPVEPMLNFGLMLGFNVILALMSFMSTFMALKPPGQYNLARDITISTLSYCVMWVMFI

PIYTSLDDKNKSLAQVGVSLLSNMGLVAAYFFPKCHLLVKQPELNTDDHFRTFLEGVPPT

PPEES

>Mm_T1R1

MLFWAAHLLLSLQLAVAYCWAFSCQRTESSPGFSLPGDFLLAGLFSLHADCLQVRHRPLV

TSCDRSDSFNGHGYHLFQAMRFTVEEINNSTALLPNITLGYELYDVCSESSNVYATLRVL

AQQGTGHLEMQRDLRNHSSKVVALIGPDNTDHAVTTAALLSPFLMPLVSYEASSVILSGK

RKFPSFLRTIPSDKYQVEVIVRLLQSFGWVWISLVGSYGDYGQLGVQALEELATPRGICV

AFKDVVPLSAQAGDPRMQRMMLRLARARTTVVVVFSNRHLAGVFFRSVVLANLTGKVWIA

SEDWAISTYITNVPGIQGIGTVLGVAIQQRQVPGLKEFEESYVQAVMGAPRTCPEGSWCG

TNQLCRECHAFTTWNMPELGAFSMSAAYNVYEAVYAVAHGLHQLLGCTSGTCARGPVYPW

QLLQQIYKVNFLLHKKTVAFDDKGDPLGYYDIIAWDWNGPEWTFEVIGSASLSPVHLDIN

KTKIQWHGKNNQVPVSVCTRDCLEGHHRLVMGSHHCCFECMPCEAGTFLNTSELHTCQPC

GTEEWAPEGSSACFSRTVEFLGWHEPISLVLLAANTLLLLLLIGTAGLFAWRLHTPVVRS

AGGRLCFLMLGSLVAGSCSLYSFFGKPTVPACLLRQPLFSLGFAIFLSCLTIRSFQLVII

FKFSTKVPTFYHTWAQNHGAGIFVIVSSTVHLFLCLTWLAMWTPRPTREYQRFPHLVILE

CTEVNSVGFLVAFAHNILLSISTFVCSYLGKELPENYNEAKCVTFSLLLHFVSWIAFFTM

SSIYQGSYLPAVNVLAGLATLSGGFSGYFLPKCYVILCRPELNNTEHFQASIQDYTRRCG

TT

>Mm_T1R2

MGPQARTLHLLFLLLHALPKPVMLVGNSDFHLAGDYLLGGLFTLHANVKSVSHLSYLQVP

KCNEYNMKVLGYNLMQAMRFAVEEINNCSSLLPGVLLGYEMVDVCYLSNNIQPGLYFLSQ

IDDFLPILKDYSQYRPQVVAVIGPDNSESAITVSNILSYFLVPQVTYSAITDKLRDKRRF

PAMLRTVPSATHHIEAMVQLMVHFQWNWIVVLVSDDDYGRENSHLLSQRLTNTGDICIAF

QEVLPVPEPNQAVRPEEQDQLDNILDKLRRTSARVVVIFSPELSLHNFFREVLRWNFTGF

VWIASESWAIDPVLHNLTELRHTGTFLGVTIQRVSIPGFSQFRVRHDKPEYPMPNETSLR

TTCNQDCDACMNITESFNNVLMLSGERVVYSVYSAVYAVAHTLHRLLHCNQVRCTKQIVY

PWQLLREIWHVNFTLLGNQLFFDEQGDMPMLLDIIQWQWGLSQNPFQSIASYSPTETRLT

YISNVSWYTPNNTVPISMCSKSCQPGQMKKPIGLHPCCFECVDCPPGTYLNRSVDEFNCL

SCPGSMWSYKNNIACFKRRLAFLEWHEVPTIVVTILAALGFISTLAILLIFWRHFQTPMV

RSAGGPMCFLMLVPLLLAFGMVPVYVGPPTVFSCFCRQAFFTVCFSVCLSCITVRSFQIV

CVFKMARRLPSAYGFWMRYHGPYVFVAFITAVKVALVAGNMLATTINPIGRTDPDDPNII

ILSCHPNYRNGLLFNTSMDLLLSVLGFSFAYVGKELPTNYNEAKFITLSMTFSFTSSISL

CTFMSVHDGVLVTIMDLLVTVLNFLAIGLGYFGPKCYMILFYPERNTSAYFNSMIQGYTM

RKS

>Mm_T1R3

MPALAIMGLSLAAFLELGMGASLCLSQQFKAQGDYILGGLFPLGSTEEATLNQRTQPNSI

PCNRFSPLGLFLAMAMKMAVEEINNGSALLPGLRLGYDLFDTCSEPVVTMKSSLMFLAKV

GSQSIAAYCNYTQYQPRVLAVIGPHSSELALITGKFFSFFLMPQVSYSASMDRLSDRETF

PSFFRTVPSDRVQLQAVVTLLQNFSWNWVAALGSDDDYGREGLSIFSSLANARGICIAHE

GLVPQHDTSGQQLGKVLDVLRQVNQSKVQVVVLFASARAVYSLFSYSIHHGLSPKVWVAS

ESWLTSDLVMTLPNIARVGTVLGFLQRGALLPEFSHYVETHLALAADPAFCASLNAELDL

EEHVMGQRCPRCDDIMLQNLSSGLLQNLSAGQLHHQIFATYAAVYSVAQALHNTLQCNVS

HCHVSEHVLPWQLLENMYNMSFHARDLTLQFDAEGNVDMEYDLKMWVWQSPTPVLHTVGT

FNGTLQLQQSKMYWPGNQVPVSQCSRQCKDGQVRRVKGFHSCCYDCVDCKAGSYRKHPDD

FTCTPCNQDQWSPEKSTACLPRRPKFLAWGEPVVLSLLLLLCLVLGLALAALGLSVHHWD

SPLVQASGGSQFCFGLICLGLFCLSVLLFPGRPSSASCLAQQPMAHLPLTGCLSTLFLQA

AETFVESELPLSWANWLCSYLRGLWAWLVVLLATFVEAALCAWYLIAFPPEVVTDWSVLP

TEVLEHCHVRSWVSLGLVHITNAMLAFLCFLGTFLVQSQPGRYNRARGLTFAMLAYFITW

VSFVPLLANVQVAYQPAVQMGAILVCALGILVTFHLPKCYVLLWLPKLNTQEFFLGRNAK

KAADENSGGGEAAQGHNE

## Nucleotide sequences of the zebrafish V2R-related OlfC family

>OlfCa1

ATGATGTGTAAGTGCACACCTGTCAGTCGCTCAGGTGAACTCAGGGAAAACATGGATTTGATGAGCTTCATTCTCTTATGGGCTGGGCTGATGAAAGTCGCAGAAGCCTCAATTGCACAGTTCAGCCAGTTGGGAGCCTCAGCCCCTGGAAACATCATCATTGGAGGACTTTTCCCCATCCATGAGGCAGTGGTGCCAGTAAACTACACCGGCAACAACAGCATCTCTGCCCCTGAGCATCCGGACTGCATCAGATTCTACACAAAGGGTCTAAATCAAGCTCTAGCGATGATTAATGCTGTAGAAATGGCAAACAAATCCCCCATGTTGAGCAGTTTGAACATTACTCTAGGATACCGAATCTACGACACATGTTCTGATGTCACGACTGCACTGCGGGCTGTCCATGATATTATGAGGCCGTTCTCAGACTGTGAATCACCAGAAGACTCATCTCAACCCGTCCAGCCAATAATGGCAGTAATTGGGACCACTTCATCCGAGATCTCAATCGCAGTTGCTCGAGATCTCAACCTTCAGATGATACCTCAGATTAGTTACGCATCTACAGCCACGATTTTGAGTGATAAAAGTCGTTTCCCTGCTTTCATGAGGACTGTGCCCAGTGATGAGTATCAAACCTGTGCCATGGCCAAACTTCTAAAGTCCAACAAATGGAGCTGGGTTGGCATTATCATTACAGATGGAGATTATGGACGTTCTGCCTTGGAAGGTTTCATACAGCACACCGAAACGGAGGGAATTTGCATCGCCTTTAAAGCAATCCTTCCAGACTCACTAGCAGATCAACAGAAACTAAACACAGACATCGAAAACACCTTGAACATCATTGAAAACAATCCGAAAGTTAGAGTGGTGATCTCGTTTGCTAAATCCTCTCAAATGCAGTTGCTATTTAAGGGGCTGCAGAGTAGAAACATTTCAAATAACATGGTGTGGGTTGCCAGTGATAACTGGTCGACGGCTAAACATATTCTGAATGATGGTAGCATCACTGATATTGGGAAAGTGCTGGGCTTTACCTTCAAGAGTGGAAATTTTACATCTTTTCATCAGTACCTAAAGAATCTACAGTTTGAAAGTGAAGATGAGATGAACAATTCATTCCTGAAGGAATTTTTAAAACTCAACGCAGGCAATGCTTCCAATACCGTGCTGGAGCTGATGAAAAGCACCAATTTGGACAAGATTTTCAGCATTGAGATGGCCGTCACTGCTGTTGCTAATGCTGTGGCTAAACTATGTGCAGAAAGACAATGTCAGGACTCTACAGCTCTCCAGCCTTGGGAGCTCCTTAGGCAGTTGCGGAGCATCACTTTTGAGAATGGAGGAGAAATGTACAAATTTGATGCGAATGGTGATATTAATTTGGGTTATGATCTCTTCCTGTGGGAAGGAGATCAATCTGACGAACATGCTGATGACATAATAGCAGAATATGATCCAACCAAAGGTGGATTCCACTACATACACAATGATCTGAGTGAAATTAAGAAAGTGGTATCTAGGTGTTCAAACAGCTGTCAGCCAGGCCAGTACAAGAAAACAGCAGAGGGTCAGCACACATGCTGTTATGAGTGCCTCACCTGTGTGGAAAACCATTATTCCAACATAACAGCTGATGAATGTTCCCCATGTGACAGTGAGAGCATGTGGTCATTGGCCAACAGCACTGAATGTCATCCCAAGGTTTTTGAATACTTTGATTGGAACAGTGGCTTCGCTATTGTCCTGCTGATACTGGCTGCCCTCGGCGTCCTTCTCCTCTTCTTCATGTCCGCACTATTCTTCTGGCAAAGACACTCTCCGGTGGTCAAGGCTGCAGGCGGGCCGCTTTGTCATCTGATCCTTGTCTCCCTGCTGGGCAGTTTTATCAGTGTCGTTTTCTTTGTAGGCGAACCGAGCGATTTGACATGCAGGGCAAGGCAGGTTATCTTCGGCTTCAGCTTCACGCTGTGCGTCTCATGCATCCTGGTCAAGTCCTTAAAAATCCTGCTGGCGTTCGAGATGAACTTTGAGCTGAAGGAGCTTCTCTGTATGCTCTATAAGCCATATATGATTGTCAGCGTCGGCATGGGGGTACAGATCATCATTTGCACTGTTTGGCTGACCTTGTACAAGCCGTTTAAAGACAAAGAGGTGCAGACCGAATCCATTCTACTTGAATGTAACGAGGGATTCTATGTGATGTTTTGGTTAATGCTGGGATATATAGCTTTGTTGGCTTTGTTCTGCTTCACGTTTGCATATATAGGCAGAAAACTACCTCAGAAGTACAATGAAGCCAAGTTCATCACTTTCAGCATGGTCATCTGCCTCATGGCGTGGATCATCTTCATCCCGATTCATGTCACCACCAGTGGCAAATACGTGCCGGCTGTGGAAATGGTTGTCATTCTTATTTCAAACTATGGAATCCTGAGCTGTCACTTTTTGCCCAAATCTTACATTATTCTTTTCAAAAAGGAGCACAATACTAAAGACGCATTCATGAAGAATGTTTATGAATATGCAAGAAAGAGCGCAGAGAATATCAAGGGCTTGACCGGGACTGAGCCGCAATTTAAACAAGAGAATTCGGTCTACACAATATCCAATCTGTCATTCGTGCCTGAAGAGAAACACGAA

>OlfCb1

ATGAAGGCTGCAGGACAGCTCTGTGTGCTGGGGCTGATGATGCTGAGCTGGGTCTCGTGGCTCCGCTGTGACCCAGTAGACTCCATGTGCGGCGCGTATTTAAATGGAGACGTCAACATCGCAATCCTGAGCTCAATTCACTCTAAGGTGAGGAACCTTCACCAGCGAACCAGACCACAGCCATTCATCTGCTCTGATTTTGATCTGATGACATTTGTACAGTCGCTGGGTGCCATCCACACAGTCGAGGAGATCAACAATTCTAATTTTCTCCCGGGGATTAAGCTGGGATATAAAATCTGTGACCCTTGTGCATCTCCCACCAAAGCACTGCACTGCCTGGAGCATCTACTGGCCATCAACGGATCCTTACCGGCCCTTTTGGACTATTCAGACTTCTGTCCACCATCCAAAGCAATAATGGGAGAAAGATACTCTGAGTTATCCATCGCTATCGCCAAGCTCCTCAGCCTCTACCTCGTTCCTCAGGTCAGCACGTCCTCCTCTTCTCCGGTTCTGAGTGATAAACTGCGCTACCCGTCCTTCATGCGTGTGATTCCCAGTGATGTGTACCAGGCTCAGGCGCTGGTCAAGCTCATGTCTCATTTCTCCTGGAACTGGGTCGGTGTCGTGTACGGAGATGATGACTATGGTAGAGGAGCCTATCAGAGCTTCATGGAGGAGTCACAGGGCAAAATATGTGCAGACTTTGAGAAAGTGGTGCCGCACTACTTAGACCAGGTGGATGTAGATAAGTACATCCAAGAAGCTGCAAATGCTATAAGATCATCCTCTGCTAATGTGACGCTGCTCATCCTGAAGCCGCAGCTGGTGGAGAAGCTCTTTAAGGAGATGATCAAGACCAACACTAGCAGAGTCTGGATCGCGAGCGACGCCTGGTCCATGTTTCGCCCACTAACCCAGATGAAGGACATCAACAAAGTGGGCCCAATCTTTGGCTTTTCTTTTTCTTTGGGAAACATTCCAGGGTTTGAAGACTACTTGAGGAACCTGAGGCCAACACCTGGAGGGAAGAACGACTTCATCGAGGAGTACCAGCAGCTGAGGCTGAACTGCTCGCTGTGGCCGTCCAACTGCACCACAGATGATGTGCTGTACGCAGTGGAGCTGAGGGAGGCCTATCGAGAGAGAGTGGCCATATACGCAATAGCACATGGGCTCAGGGAACTATTGAAGTGCAACGACACCACCTGCTCGTCTGAGACCAACTTCCCTCCGTGGCAGCTCGTGGCAAGCATGCGCAGAGTCAATTTCACACTCGACGGCAACTCTTATTTCTTTAATGAAAATGGTGACTTCACGGATGGTTATGATGTCATAATGTGGAAGGAAAATAATGAAGAGAGAATGATTGAGCCTGTGGGGAAATTCCTCATTAAAAAAGGAGATGTGGAAATCTTTAGTGAATACCACTGGATCAATGAAACGCTCCTCAGCTCCAGTTGCTCTCAGTTCTGCCAACCGGGAACAGTGAAGAAGCAGTCAAACATCACCTGCTGCTACAAGTGCGTCCCCTGCGATGTGGGATACTACACCAACGCATCAGATCAGTCTGAATGCCTGAAGTGTCCTGACGGGCAGTCATCTCTTAAAGGAGCAACCCAATGTGAGATCGTCAAGGAGCAGTATCTGTTCTGGACGGATGGTTACCCCATAACTCTGTTAGCGGCCACTGCTATAGGCCTGCTTCTGGTGTTGGTTTCGTCCATCCTCTTCTGTGTGCACAGGAACACGATGGTCATCAAAAAGGCGGACGGCACAATGTCAGGGTTCATGCTTCTGGGGCTGACGGCCAGCTTCATCAGCGTGATCATGTTCATCGGCAGACCAAATGAGCATCTGTGCAGAGCACAGCAAGCCGTGTACAGCCTCGGCTTCACCCTGTGTGTTTCCTGCATCCTGGTTAAAGCCTTTCGCACCTTCCTGGCCTTCCTAGTCTTCAATCCACAAAAGCAGCATGAGCTTAAGAAACTCTACAAGCCTCTGATCATCCTGGTCCTGCTGACTGGGGGTCAAGCCATCATCCTACTGTTCTGGCTGATTTTAAAGTCTCCTTACCCAGATCCTCTGTGGCCAGGAAGTGGCTTGATCAAATATGTCATCTGTAATGAAGGCTCTATTGCAGGTTTTGGGGCAATGCATGGCTACATCGCTCTGCTGGCCTTCACCTGTTTCTTTCTTGCGTTTAAAGGCAGAAAAGTCCCGCAGGATTTCAACGAAACCGGAGTCATCATCTTCAGCATGCTGATCCATCTGTTCGTCTGGCTTTGCTTCATTCCCATTTACATTGACAGAAACAGGACAGAGCAGCGGCACATCGTACAGGCCTCAGCCATCCTGGCCTCCAACTACGGCATCATGTTCTGTCATTTTCTCCCCAAGTGTTATGTGGTGCTGTGGGAGCTGTCCGAGAACTCTAGGGCGATTATCCTAGGCAGGCTGACCAGACGCATTAGAGACGAGATGACCAGTGCAGATATTGCTGTAGTCACAGTCTCGGGCATCATCTGTGAAGAAGTATCAGCAGAGATCAGCCCCGTTTCCAAAGATCCTGCCTTAAAATCTGTGGAGTTATTTCATGCAGAGAGAGGCGGTGCAGAGCAGACCGTGACCCACCGTGTGCAGACCAGACGAAGACACATTACTAAA

>OlfCc1

ATGGATTTAACTGGGTTGAGTTACGAAGGACGCTTTTTGTTGGTTTTATGCATGATTTCTTACCTTTTCACACCCACCGATGCTGAAGGGAGCTGTAAACTCAAAGCCAAATTCAACCTGAGGGGTTACAAAGAAGTGGAGAAAACCACGGTGGTGATTGGCGGTATGTTTCCCGTTCATCGGAGCCTGGTTTCAACAGACAGCAACACCACAGACCCTCCAGAGTCTGTGGATTGCCAGGGCTTTAACTTCCGCGCATTCCGCTGGGCTCAGACCATGTTGTTTGCTCTGAAAGAGATTAACAGCAGAACAGATCTGTTACCCAAAACTGAGCTGGGTTATGTCATCTATGACTCCTGCTTTACCATTTCCAAAGCTGTGGAGGGAACACTCACCTTCCTGACAGGCCAGGACGAGGCTGTGCCCAACTACCGCTGTGGGAATGGACCTCCTCTATCTGCTTTGGTGGGGGCTGGAGGATCAGATCTGTCTATCGCCACAGCCAGGATTCTTGGCCTCTACTATTTCCCACAAGTCAGTTACGAGTCCTCATGCTCCGTTCTGGAAAGCAGGTTCCAGTACCCCACATTCCTCCGTACCATCCCAAGTGATGAGAACCAGTCTGTTGCGATGGCAAAACTGGTCCTTCGCTTTGGCTGGACTTGGGTGGGTACCATCGCTGCCGAGGATGACTATGGGAAATACGGCATTAAGAGGTTTAAGGAGGTGGTGGAAGAAGCAGGAGTGTGCATATCCTTCTCTGAGACCCTTCCGAAAATTAGCAACCCAGAGGCCATCCAGCGCATAGTGCAGACGGTGCACGACTCCACGGCTAAGATCATTGTAGTGTTCTCCTCCGATGTGGATCTCAGTCCTCTAGTGGAGGCACTACTGCAAAGCAACGTCACCAACCGTACATGGATCGCCAGCGAAGCTTGGGTCACTTCAGCTGCCATTTCGCGTCAGCCCAACGTTCTGTCTCTTCTGGGCGGCACTATAGGGTTTGCCGTTAAACGTGCCGAAATACCCGGCCTGAAAAAGCACTTACTGAGCATTAGTCCATTTAACGACTCTCTGACAGAAGAATTTTGGGGGATAGTCTTTAACTGTACTACAAATTATACGCTGATATTAAAAGGCATGAGGAGATGCACTGGAGAAGAGATGTTAGGGACAGTGGATAATACCTACTCCGATGTGTCGCAGTTAAGGATTACATACAACGTCTATAAGGCTGTATATGCTGTAGCACATGCTTTACATAACCTAGAGCAATGCAAAACAGGAAGCGGGCCTTTTGAGAACGACACATGTGCTGATATTACTAATTTCGAGCCTTGGCAGCTCATGTACTACCTGATACACCTCAGATTCACCGTGCCTCACACCGGAGAGGAGTTGTTCTTTACTAATGGTGAGGTGGAAGGCTTTTATGAACTCTTAAATTGGCAGAGTGATTCAAACGGAGGGATTACATATACACATATCGGTTACTATAATAGCACAGCGGCGCCTGAGGACAAGTTGGTCATTAATAACAACTCCATCATATGGAATAATAATGTTCTGAAGGCACCACGCTCTGTGTGCAGTGAACGCTGTCAGCCAGGCACTAGAATGGGCATCCGGCAAGGAGAACCAGTCTGTTGCTTTGACTGCATTCCCTGTGCAGATGGCGAGATTTCCAACACAACAGATGCACGAGGCTGTATCCAATGTGATGGGGACTACTGGTCCAATGCCAATCATGACGAGTGTGTGCCCAAGACTATTGAATTCCTTGACTTTTCAGAACCTCTTGGAATTACGCTTATTGCCATTGCTGCTTTTGGGGCTCTTGCGACCATTGTAGTTGCCATCATCTTCTTAATGCATCTTAATACACCTTTGGTGAATGTCAATGACCCTCTGCTTACCTTTTCTCTACTGTTGGGTTTGGTGATCACCTTCTTGTGCTCCATTGTGTTCCTTGGTAAGCCTCAGATGTGGTCCTGCATGACCAGTCAGATGGCTTTGGCTGTTGGTTTTGCTCTCATCCTCTCTTCGCTAATGGGCAAATCTGCTTTGCTTATGCTGAGGGCTAGAGCTGTAAAGGCAGTCAAAGCTGCCGCCAAAGCTGCCAAAGCAGCCGCGGCGGCGTCTGAGCAGAGTCCCGATACCGCTGTCTTCGCTCCAGCTATTCCTCAAAAGAATGACGTTGACCCCATACATCCAAGACACCAGAGAGCGATAATGATCTTGTGCACTCTGATTCAAGTCGTAGGCTGCACTGCGTGGTTGATTCTGATGCCTCCACACCCCGTGAAGAACACAGGCGTCCAGAACATCAAGATCATCCTGGAGTGTGACCCTGGAAACATCATCTTCATTTGCTCCATCTTCGGTTACGACATTCTGTTGGCTCTGGTGACGTTCGCATTCGCTTTTGTGGCTCGTAAGTTGGAAGACCACTTCAACGAGGGTAAGAGTGTGACCTTCGGCATGCTGGTGTTTTTTATTGTTTGGAGCTCCTTTGTTCCTGCTTACCTGAGCACGCGGGGGAAGTTTATGGTGGCCGTGCAGATTTTCGCCATTTTGGCCTCCAGCTTCGGCTTGCTTGCTTGCGTCTTCATACCCAAGTGCTACGTGCTACTTGTCAAACCAGAGAGGAACAAAGAGGAGATGATGATTCCCCGACCCAAATCACGTGACATAGCTGCTGCTGCTGCAAGCTCTGCATCGCTCGCAACCACCAGCAGCTCTGGTAACCCTAATGGAACCACGGTGTCCACTTTGTCCCTAGAAGAG

>OlfCd1

ATGACAAAGAAGTCTGAGTTATTGCTGTTAATGGTGCTGGTGACACATGGCATCTGTGTACCCAATTTAGCACAAGTCTGCAGACTTCTTGGTCAGCCTGCCCTTCCTCTACTCTCTGAAGAAAGAAACATTAACATTGGGGCAATTTTCTCATTGCACAAAAGTGCTCTTTTGAAGAGCCATAATTTCACTTCCAAACCAAAGCAAACAACATGTGGCGGCTTGAACCTCCGTGAATTTAAATTTGTTCAAACACTAATTTTTGCCATTGAGGAGATTAATAACAGCTCTCTGTTGCCTGGTGCGTCACTGGGCTATAAAATATATGATTCCTGTGGCTCAGTAGCTCAAGCTATTTTCTCAGGCATGGCTTTGATGAACGGTTATGAAGAGACTTTGAGTGATACATCTTGTTCTACACCACCAGCTGTTCATGCTATTGTAGGAGAATCAAACTCATCTCCCACAATTGGCCTGGCATCAGTAGTTGGTCCATTTAGCTTACCTCTTGTCAGTCATTTTGCTACATGTTCATGCCTGAGTAACAGAAAAAGATTTCGAACCTTTTTTAGAACAATACCCAGCGATTATTACCAAAGCCGAGCACTGGCTCAACTTGTCAAACACTTTGGCTGGACCTGGGTTGGGACAGTCAGAAGCCGCAGTGACTATGGTAATAATGGCATTGCAACATTTGAGGAAGCAGCAAAACAAGAGGGGATTTGTATTGAATACTCAGAAGCTGTATTCAAAACAGATCCAGAAGTACAGTTTCTGAAGACAATAGAAGTAATAAAGAAGGCAACCACAAAGGTGGTGGTGGCGTTTATGGCATTTGGAGATTTTGTCCTCCTTTTGAAAGTAATGGCACAACAAAACATTACAGGGATACAGTGGATCGGTAGTGAGTCCTGGATAACATCGCGAAATCTTGCAGAAACAAAGGAATACCATTTTCTCTCTGGAGCTGTGGGTTTTGCTATTGCAAATTCCAAACCTGTTGGCCTACGAGAGTTCTTAATGAATGTGCACCCTGATAAAGAGCTAAACAATGAACTTTTAAAAGAATTTTGGGAAACTGTTTTTCTTTGCTCTTTCAGGAACAGCAGCAGTGGTGGATGTACTGGCTCAGAAAAACTGTCAGAGCTTCAGAATGAATACACTGATTTCTCAGAATTACGAATCGAAAATAAAGTATACACTGCTGTGTATGCTGTTGCACATTCTTTGCATAATGTTCTAAAAGGCTTCAAATTCTTCACCAACAGCAGCAAAAAGCAATTGCCCACACCACAGAAGGTGTTGGAATATTTGAGAGATGTGAACTTCACTGTCAATACGAATGAGAATATCTTCTTTGATGCAAGTGGTGATCCAGTGGCAAGATACGACTTGGTGAACTGGCAGCCTACTAAGGATGGAAGTCTGCAGTTTAAACTTGTGGGCATCTATGACAGTTCACTGCCTTCAGAGCAACGTCTTCAAATCAATCAGGAATCCATGTTATGGGCAGGAAACAGTGGACAATTGCCTGTGTCCGTGTGCAGTGAGAGCTGTCCCCCTGGAACAAGAAAAGCTGTGCAAAAAGGAAGACCCATCTGCTGTTATGACTGTATCCCATGCGCAGAGGGAGAAATCAGTAATTATACAGATTCTAGTGACTGTTTTCCTTGTGGTTTGGAGTACTGGTCAAATGAAAGCAAAGACAGATGTGTACTTAAAGTCATTGAATTCCTTTCCTATACAGAAATCATGGGTGTGGGGCTTTGTATTTTCTCCTTCATTGGGGTATTATTAACTGCAACGGTGTCTTTTCTGTTTTATTTTTATAAAGAAACACCTATTGTCAGGGCCAACAACTCAGAGTTGAGCTTCCTGCTGCTCTTCTCATTAACTCTGTGTTTTCTCTGTTCACTTACATTCATTGGTCGTCCCACTGACTGGTCCTGTATGTTGCGTCACACAGCATTTGGGATCACTTTTGTCCTCTGTATTTCCTGTGTTCTGGGGAAAACAATAGTGGTTTTAATGGCATTCAGGGCTACACTTCCAGGAAGTAATGTCATGAAATGGTTTGGGCCTCTTCAACAGAGACTTAGTGTAGTTTCTTTAACATTAATACAGGTGATCATCTGTGTTCTTTGGTTAACAATGTCTCCTCCTTTCCCATTTCTAAATTTGAGTTATTACAGAGAAAAGATCATCCTAGAATGTAACTTAGGTTCAGCTTTTGGTTTCTGGGCTGTTCTGGGTTATACTGGCCTGTTATCAACCTTGTGTTTTGTTTTAGCTTTTTTTGCTCGAAAACTCCCAGATAACTTCAATGAAGCCAAGTTCATCACTTTCAGCATGCTAATTTTCTGTGCTGTCTGGCTCACATTTATCCCAGCTTATGTCAGTGCTCCTGGAAAATATACTGTTGCTGTGCAATTATTTGCTATTTTAGCTTCTAGTTTTGGTTTACTATTTTGCATATTTGCCCCAAAATGTTACATTATTTTGCTAAAACCTGAAAAAAACACAAAGAAACAAATGATGGGAAAGTCC

>OlfCd2

ATGCAACACTATTTCAGTTTTGCCTCAGAGGATCTGCTGTTCATGTTTACACTTGGCATGGCAAATACTCTGTCACTGCTGCTACTTCTACTGTTATTACATGGTCACTTTCTTCCAGCAACAGTGCAAATATGTGATCTGGTAGGTCAATCTGCTTTGCCTGTACTGTCTGCAGAAAGAGATATCAACATTGGAGCAATTTTCTCAATTCATAGGAATGCTTTGCTAAGGCTGTATCCTTTTACTTCCAAACCAGAGCCAACAACATGTGTCAGGTTAAACTTGCGTGAATTTAAATTTGCCCAGACATTTATTTTTGCTATTGAGGAAATAAATAACAACACACAGTTGTTACCTGGAGTTACTTTGGGTTATAAAATCTATGATGCCTGTAATGCAATTTCTCCGACTCTTGTCTCAGGCATGGCTTTAATAAATGGCTATGAAAATACTTTGAGTGATACATCCTGCTCTCAGCCACCTGCTGTTCAGGCTATAGTTGGAGAATCATCATCCAGTCCCACAACGGCCTTGGCTGCTTTAGTTGGTCCATTTAACATACCCGTTATCAGTCATTTTGCAACATGTGCATGTCTGAGTAACAGGAAAAGATATCCCTCCTTCTTCAGAACTATACCGAGTGACTATTACCAAAGCAGAGCACTGGCTCAGCTTGTCAAGCACTTTGGCTGGACCTGGGTTGGGACAGTCAGGAGTCGCAGTGACTATGGTAATAATGGCATCACAGCATTCGAGGATGCAGCAAAACAAGAAGGGATTTGCATTGAGTACTCAGAGGCCATATTAAGAACTGATCCACAAGAACAGTTTCGAAAGACAGTGGAAGTGATCAAAAAGGGCACATCCAAGGTGGTGGTAGCCTTTATTTCATTCGGAGATTTTGCTCCCCTTGTAAAAGTAATTGCAGAAAACAACATCACAGGACTGCAGTGGGTTGGCAGTGAATCTTGGATAACATCTCGAAATCTTGCAGAAACAAAGGAATACAGTTTCCTTTCTGGAGCTGTGGGCTTTGCCATTGTAAATGCCAAACTTGTAGGTTTAAAAGAGTTCCTGGTAAATGTGCACCCAGATCAAGAACCAAAAAACAAGCTTTTAAAAGAATTTTGGGAAACAGCTTTTCAGTGTTCTTTCAGCAACAGTGACAGTGCTCTATGTACTGGTTCTGAGAAACTTGGAGAGTTGAAGAATGAATATACTGATGTATCAGAGCTACGGATAGAACACAAAGCGTATGCTGCAGTGTATGCAGTTGCACACACACTGCATAATGTTTTTAAAGATGTAAAAGCCCCCAACAACAGCAAAAGAGAGCTACCCACACCACAAAAGGCAATGAATGAATGCATAAATACATGAATAAATAATTACATGAATACTGTATTGCAGTATATGAGAAATCTGAGTTTCACAATAAAAACAGGTGAGAACATCTTCTTTGATGCAAGTGGAGATCCAGTGGCAAGATATGACCTTGTGAATTGGCAGACTGCTGAGGATGGAAGTCTGCAGTTTAAGCATGTGGGCATCTATGACAGCTCACTGCCTTCAAAGCAACGTCTACAAGTCAATCAGGAAATCATGCTGTGGGCAGGGAAAAGTGGATTGTTACCATTGTCCGTGTGCACTGAGAGCTGTCTCCCAGGAACTAGAAAGGCTGTACAAAAAGGACGCCCTGTCTGCTGTTATGACTGTATTCCATGTGCAGAGGGAGAAATCAGTAACAGCACAGATTCTAGTGACTGCTTTTCATGTGATTTGGAGTACTGGTCAAATGAAAAGAAAGACAGATGTATATTAAAAGTGGTTGAATTCCTTTCATATGCAGAAATCATGGGGATAATTCTTTGTATTGTCTCTTTCATTGGGTTGCTATTAACAGTAATGGTAACTTGTCTGTTCTATCTTCATAAAGAAACACCTATTGTCAGAGCCAACAACTCAGAGCTGAGCTTTTTGCTGCTCTTCTCACTCTCACTGTGTTTTCTCTGTTCACTCACATTCATTGGTCGTCCCACTCAGTGGTCCTGTATGTTGCGTCACACAGCATTTGGGATCACGTTTATCCTCTGTATTTCCTGTGTTCTGGGGAAAACAATAGTGGTGTTGATGGCATTCCGAGCTACACTTCCAGGCAGTAATGTCATGAAATGGTTTGGGCCTCTTCAACAAAGACTCAGTGTTGTTTCCTTAACATTAGTACAGGTTATTGTCTGTTTGCTTTGGTTAACATTATCTCCTCCTTACCCATATATGAATTTGAGCTATTACAGAGAAAAGATCATCCTTGAATGTAACTTAGGTTCAGCATTCGGTTTCTGGGCTGTTCTGGGTTATACTGGTTTGCTATCAACCTTGTGTTTTGTTTTAGCTTTTCTTGCTCGAAAACTCCCGGATAACTTTAACGAAGCGAAGTTCATTACATTTAGTATGCTCATATTCTGTGCTGTCTGGCTTACATTTATTCCAGCATATGTAAGTTCTCCTGGAAAGTATACAGTAGCTGTGGAGATCTTTGCCATTTTAGTTTCAAGTTTTGGTTTACTATTTTGCATATTTGCTCCAAAGTGTTACATCATTTTGCTGAAACCAGAAAAAAACACAAAGAAACAAATGATGGGTAAACTT

>OlfCd3

ATGGCAAAGAAGATTGTGTCACTGCCAGTACTGCTGCTGATAGTGTATGGCATAAGTGTTTCAGCTTTGGCACAAGTCTGCAGACTGCTGAGTCAGCCTGCACTCCCTCTACTTTCTGCAGAAAGAGACATCAACATTGGGGCAATATTCTCAATCCACATTAGTGCTCTGCTAAAGATGCATTCTTTTACTTTCAAGCCAGAGTCAACTGCATGCATCAGCTTAAGTTTGCGTGAGTTTAAATTTGCTCAAACACTGATTTTTGCCATCGAAGAGATTAATAACAACACACAGCTATTGCCTGGTGTGTCTTTGGGCTATAAGATATATGATTCATGTGGCTCAATAGCTCAAGCTATATTCTCAGGCATGGCTTTGATGAATGGTTATGAAGAAACTTTGAGCGATACATCCTGCTCTAGACCACCAGCTGTTAATGCCATTGTAGGAGAGTCAAACTCCTCTCCCACCATAGCCTTGGCTTCTATAGCTGGTCCATTCAGTTTACCTGTTATCAGTCACTTTGCCACATGTGCCTGCTTGAGTAACAGAAAAAGGTATGCATCCTTCTTTAGAACGATCCCCAGTGACTATTACCAAAGCAGAGCACTGGCTCAGCTTGTTAAGCACTTTGGCTGGACCTGGGTTGGTACAGTCAGGAGTCGCAGTGATTATGGCAATAATGGCATTGCAACATTCGAGGAAGCAGCAAAAAAAGAGGGGATTTGTATTGAATACTCAGAGGCCATATTAAGAACTGATCCACAAGATCAATTTCTGAAGACAGTAGAAGTGATAAAGAAGGGCACTGCGAGGGTTGTGTTAGCTTTTGTTGCATTAAGAGATTTTTACCCACTTTTAAAAGAAATTGCGCAACAAAACATTACAGGGCTGCAGTGGGTTGGCAGTGAATCTTGGATAACATCCCGAACTGTAGCAGAAACAAAGGAATATAGTTTCCTTTCTGGAGCTGTGGGCTTTTCTATAGCAAATGTCAAACTTGTGGGCCTACTAGATTTTCTAGTGAATGTGCACCCTGATCATGAACCGAAAAATAAACTTTTAAAAGAATTCTGGGAAACAACATTCCAGTGCTCTTTTAATAACAGAGGAAGCGTTGGGTGTACTGGCTCAGAGAAACTAGCAAACTTACAAAATGAATATACTGATGCCTCAGAGCTACGGATAGCCAATAAAGTGTACACAGCAGTGTATGCTGTTGCACATACACTACACAATATATTTCAAGACTTCAAATCAGCCAACAGGAGCAAATTAAAACAACCAACACCTCAAATGGTACTGAACTATATGAAAGATGTAAGATTCACTGTCAAAACAGGTGAAGAAATATTTTTTGATACAAGTGGTGATCCAGTGGCAAGATATGATCTTGTGAACTGGCAACCTTCTGGGGATGGAAGTCTGCAGTTTAAAAATGTGGGCATCTATGACAGTTCACTTCCTTCAGAGAAATGTCTTCAGGTTAATCAGGAACATGTGCTATGGGCAGATAACAGTAGACAGTTGCCTGTATCCGTGTGCAGTGAGAGCTGCCCCCTTGGAACTAGGAAGGCTGTGCAAAAAGGAAGACCGGTCTGCTGTTTTGATTGTATTCCATGTGCAGATGGAGAAATAAGTAACAACACTGACTCTAGTGACTGCTCTCCTTGTGATGAGGAGTACTGGTCAAATGAAAGAAAAGAAAAATGTGTGTTAAAAGTGATCGAATTCCTCTCTTATACTGAAATCATGGGGATGGTGCTTTGTATATTCTCCTTTATTGGATTGTTATTAACAGCAATGGTATCTTTTTTGTTCTATCTTCACAAAGAAACACCTATTGTCAGAGCCAACAACTCAGAACTGAGCTTCCTGCTGCTCTTCTCACTCTCACTGTGTTTTCTTTGTTCACTCACTTTCATTGGTCGTCCCACTGAGTGGTCCTGTATGTTGCGTCACACAGCATTTGGGATCACTTTTGTCCTCTGTATTTCCTGTGTTCTGGGGAAAACAATAGTGGTGTTAATGGCCTTCAGGGCTACACTTCCGGGAAGTAATGTCATGAAATGGTTTGGGCCTACACAACAACGACTCAGTGTTGTTTTTTTAACATTAATACAGGTGATTATCTGTGTTGTTTGGTTAACTGTCTCTCCTCCATTCCCATATAAGAATTTGAGCTATTACAGAGAAAAGATCATCCTGGAATGTAATTTGGGTTCAGCTTTGGGATTTTGGGCCGTTCTGGGTTATACTGGCCTGCTATCTGTCTTGTGTTTTATTTTAGCTTTTCTTGCTCGTAAACTCCCTGATAACTTCAATGAAGCCAAGTTCATCACATTTAGTATGCTCATATTTTGTGCTGTTTGGCTCACATTCATTCCAGCTTATGTCAGCTCTCCTGGAAAATTCACAGTAGCAGTGGAGATATTTGCTATCTTATCTTCAAGTTTTGGTTTACTATTTTGCATATTTGCTCCGAAATGTTACATAATTTTACTCAAACCAGAAAAAAACACAAAGAAACAAATGATGGGAAAGTCGTCTTCTAAAACTATT

>OlfCe1

ATGGTTTTCTTACACACCTGGCTTTTTTTTACACTAGTGAGAGCAGCCAACCCTCCATGCAAGTTATTGGGGCCTCCTAACATACCTCAGTTTAGCAAAGACGGTGATGTGACAATCGGGGGCATTTTCTCTTTCCACAACAGCTGGGAGGAAATTATGCCCACATTCACATCAAAACCAGAACAGCCAAAATGCAAGAGTTTGAGTCTCAGAGAATTTCAAAATGCACAGACAATGATATATGCCATTGAGGAAATCAACAAGAGAGCTGATATTCTTCCTGGTCTAAGTTTGGGTTACAGAATCTTTGATTCATGTGGATCCATTGAAATGGCTCTCAGAGCCTCTCTGTCATTAGTTAATGGGGAAAATGCCTCACATTTATCCTGTCAAAGACCAAAAACAGTCCAAGCAATCATAGCAGAAACATCCTCTACTCCTACCATTGCCATCTCTGCCACTGTTGGACCGCTGCATTTACCGGTGATTAGTCATTTTGCAACATGTGCCTGCCTCAGCGACAGAAAGAAACATCCCTCATTTTTCAGGACTGTTCCAAGTGATTATTACCAGAGCAGAGCACTGGCCAAACTAGTCAAGTATTTTGGCTGGACCTGGGTAGGGGCTCTTTGCAGTGATAATGACTATGGAAACAATGGCATGAACACTTTTATCAAAGCAGCTACAGAATTTGGAGTCTGTGTAGAATTTTCTGAAGCATTCTTCAGGACTCATCCCAGGGAGGAAATTTTAAGAATCGTGGATGTTGTAAAGAAATCAAGCTCAAAAGTGATTGTTGCCTTTGTATCATACTCAGACATGGAAGTTCTTTTATTGGAATTAGCTAAACAGAACATTACTGGGCTACAGTGGATTGGTAGTGAATCCTGGATCTCTGATATGAACATTGCTACTGGTAAATGGCAATACATTCTTAGAGGGTCTATGGGCTTTGCCATCCCAAAAGCTGAAATTCAAGGATTAAGGGAATTTCTAATTAAAATCCACCCATCTTCCAACATATATCTGTACAAAGAGTTGTGGGAATCAGTGTTTCAATGTAGACTTTCCACAGAACAAAGTTCTGAAAGCAAGAGCATGTGCACAGGCAATGAAAGTTTAAACAATGTGCAGAACCAGTACACAGATGTTACAGAATTACAAATAGCCAATAATGTTTACAAGGCTGTGTTTGCCATTGCACATGCACTGAACAGCTCAGTTGGCTGTTCAAAATGGGACACAAATCAAAGAGAATGTAATACATGGCAGGTAGTACTTCAAGCCCTAAGGGAAGTGAGTTTTTTCACTGAAACGGGTGAAAAGGTATTCTTTGATAAAAATGGAGATCCGGCAGCAAGGTATGATCTCTTGAACTGGCAGCAGGGGGAAGAAGGGGCAACAAAGTTTGTTAAAGTGGGTTTCTATGATGCCTCCCTACAACCAGAATTTCAACTTTCCTTTAATAACATCACCATCATGTGGGCCAAGAATCAACATCAGGTGCCTGTGTCTGTTTGCAGTGAAAGCTGTCCCATGGGCACTAGGAAGGCTGTGAAAAAAGGAAAACCTCTTTGCTGTTATGACTGTATTCAATGTGCAGAAGGGGAAATAAGTAATAAGACAGATTCAGTGATGTGTCTCAAGTGTCCTCCAGAATTTTGGTCCAACAAATGGAGAGATACATGTGTCCCAAAACTCGTTGAATTCTTATCATTTGAGGATGTGATGGGAATTGTTTTAATAATATTTTCTTTATTGGGTGTGTCTTTTACTTTGGGTATTGCTATCATTTTCTTTGTGCATAAGGACACACCAATAGTAAAAGCCAACAACTCGGAGCTAAGCTTCCTGCTGCTCTTCTCACTGACTCTGTGTTTCCTCTGTTCACTCACTTTCATTGGTCGACCCACTGAGTGGTCCTGTATGTTGCGTCACACAGCGTTTGGGATCACTTTTGTTCTCTGCATCTCATGTGTTCTGGGGAAAACAATAGTGGTGTTAATGGCCTTCAAAGCTACACTTCCAGGAAGTAATGTCATGAAGTGGTTTGGGCCCTTACAGCAAAAACTCAGTGTAATTACTTTCACTCTGTTACAGGTTGTAATTTGTGTGCTTTGGTTAACATTATCTCCTCCTTTCCCATACATGAACATGAATTATTACCAAGAAAGAATAATTCTAGAATGTAATTTAGGTTCAGCATTTGGCTTTTGGGCTGTTCTTGGTTATATTGGCTTACTGGCTATATTGTGTTTCATTCTGGCTTTTCTGGCTCGAAAGTTGCCTGATAAATTCAATGAAGCTAAATTCATCACTTTCAGTATGCTCATATTCTGTGCTGTGTGGATCACGTTTATACCAGCTTATGTCAGTTCTCCTGGAAAATTCACTGTAGCTGTGGAGATATTTGCTATTTTAGCTTCAAGTTTTGCTTTGCTGTTTTGCATATTTGCTCCAAAATGTTACATAATTTTACTCAGACCAGAAGAAAACACAAAGAAACACATGATGAGTAAATCA

>OlfCe2

ATGTTTTTCTTACACTTATGGCTTTTTTTCACACTAGTTAGAGCAGCCAACACTCTTTGCAAATTATTGGGGCCTCCTAACATACCTCAGCTTAGCAAAGATGGTGATGTGACAATTGGAGGCATTTTCTCCATCCACACTAGCTGGGAGGAAATTTTGCCCACATTCACATCAAAACCAGAACAGCCAAAATGCAAGAGTTTGAGTCTGGGAGAATTTCAAAATGCACAGACAATGATATATGCCATTGAGGAAATCAACAAGAGAGCTGATATTATTTCTGGTCAGAGTTTGGGTTACAGAATCTATGATTCATGTGGATCCACAGATATGGCTCTTAAAGCCTCTCTGTCATTAGTTAATGGGGAAAATGCTTCTCAGTTATACTGTCAAAGACCTCTGACAGTCCAAGCTATAATTGCAGAAACATACTCTACTCCTACCATTGCAATCTCAGCCACTGTTGGACTGCTGCATTTACCAGTGGTTAGTCATTATGCAACATGTGCCTGCCTCAGTGACAAAAAGAAACATCCCTCATTTTTTAGGACTATTCCCAGTGATTATTACCAGAGCAGAGCACTGGCCAAACTGGTCCAATATTTTGGCTGGACCTGGGTAGGGGCTCTTTGCAGTGATAATGACTATGGACACAATGGCATGAACACTTTTATCAAAGCAGCTACAGAATTTGGAGTCTGTGTAGAATTTTCTGAATCATTCTTCAGGACTCATCCCAGGGAGGAAATTTTAAGAATCGTGAATATTGTAAAGAAATCAAGTTCAAAAGTTATTGTTGCCTTTGTATCATATGCAGACATGGAAGTTCTTTTATTGGAATTAGCTAAACAGAACATTACTGGGCTACAGTGGATTGGTAGTGAATCCTGGATCTCTGATATGAACATTGCTACTGGTAAATGGCAATACATTCTTAGAGGATCGATGGGTTTTGCCATCCCAAAATCTGAAATACAAGGTCTAAAGGAATTTCTAACTAAAGTCAATCCATCTTCCAACATATATCTGTATAAAGAGTTGTGGGAATCAGTGTTTCAATGTAGACTTTCCCCAGAACAAAGTTCTGAAAGCAAGAGCATGTGCAAAGGCAATGAAAGTTTAAACCATGTGCAGAACCAGTACACAGATGTTACAGAATTACAAATACCCAATAATGTTTACAAGGCTGTGTTAGCCATTGCACATGCGCTGAACAGCTCAGTTGGTTGTTTAAAATGGGACAAAAATCAAAGAGAATGTCTGGAAAAAATCAATAACACATGGCAGGTAGTACTTCAAGCCTTGAGAGAAGTGAGTTTTTTCACTGAAACGGGTGAAAAGGTATTCTTTGATAAAAATGGAGATCCGGCAGCAAGGTATGATCTCTTGAACTGGCAGCAGGGGGAAGAAGGGGCAACAAAGTTTGTTAAAGTGGGTTTCTATGATGCCTCCCTACAACCAGAATTTCAACTTTCCTTTAATAACATCACCATCATGTGGGCCAAGGTGCCTGTGTCTGTGTGCAGTGAAAGCTGTCCCATGGGCACTAGGAAGGCTGTGAAAAAAGGAAAACCTATTTGCTGTTATGACTGTATTCAATGTGCAGAAGGGGAAATAAGTAATAAGACAGATTCAGTGATGTGTCTCAAGTGTCCTCCAGAATTTTGGTCCAACAAATGGAGAGATACATGTGTCCCAAAACTTGTTGAATTCTTATCATTTGAGGATGTGATGGGAATTGTTTTAATAATATTTTCTTTATTGGGTGTGTCTTTTACTTTGGGTATTGCTATCATTTTCTTTGTGCATAAGGACACACCAATAGTAAAAGCCAACAACTCGGAGCTAAGCTTCCTGCTGCTCTTCTCACTGACTCTGTGTTTCCTCTGTTCACTCACTTTCATTGGTCGACCCACTGAGTGGTCCTGTATGTTGCGTCACACAGCGTTTGGGATCACTTTTGTTCTCTGCATCTCATGTGTTCTGGGGAAAACAATAGTGGTGTTAATGGCCTTCAAAGCTACACTTCCAGGAAGTAATGTCATGAAGTGGTTTGGGCCCTTACAGCAAAAACTCAGTGTAATTACTTTCACTCTGTTACAGGTTGTAATTTGTGTGCTTTGGTTAACATTATCTCCTCCTTTCCCATACATGAACATGAATTATTACCAAGAAAGAATAATTCTAGAATGTAATTTAGGTTCAGCATTTGGCTTTTGGGCTGTTCTTGGTTATATTGGCTTACTGGCTATATTGTGTTTCATTCTGGCTTTTCTGGCTCGAAAGTTGCCTGATAACTTTAATGAAGCCAAATTCATCACTTTCAGTATGCTCATATTCTGTGCTGTGTGGATCACGTTTATACCAGCTTATGTCAGTTCTCCTGGAAAATTCACTGTAGCTGTGGAGATATTTGCTATTTTAGCTTCAAGTTTTGGTTCACTGTTTTGCATATTTGCTCCAAAATGTTACATCATTTTACTCAAACCAGAAGAAAACACAAAGAAACACATGATGAGTAAATCA

>OlfCf1

ATGATTGTTATTATTACTACAAAAAAAGATCAAGGAAGATTTAGTAACATACTAGGAGCTGCAATGACATTAGTTAATAATCAAGAGCAGTCAGAACCAGGAGCAAACTGCTCTAAACCTCAAACCGTGCAAGCCATCATTGGACATTCTGGATCAACACCAACTATGAGCTTTGCAAAAATAGTTGGAAATTTTCATATACCTGTGATCAGTCATTTTGCTACATGTGCTTGCTTGAGCAACAGAAAGGAATATCCCTCCTTTTTCAGAACTATTCCCAATGACTTCTATCAAAGCAGAGCACTGGCCCAGCTGGTCAGACATTTTGGCTGGACATGGGTGGGGGCATTAAGCAATAAAAATGATTATGGTATAGATGGGATAACCACTTTTATCAAATCTGCTACTGAGGAAGGGGTTTGCATTGAATACATTGAAGCCTTTGAAAGTACAAGTCAATACACTGAAATACTGAGAATTGTGAACATCATAAGAAGCTCTACATCTAAGGTAATAATGGCCTTCATGTCACACAGAGAGATCAAATTATTAGTAGATGAGCTCTACAAGCAGAATGTCACAGGACTGCAGTGGATTGGCAGTGATGCCTGGATCACAGATGACTCTCTAGCAGATATCTATGGTCACACTTTACTTACTGGCTCTATTGGATTTGCTGTCCGCAATGCTCAAATACCTGGACTGGGCTCTTTTCTACAGAAAGTTAATCCCTCTCAATTTCCTAACAGCATATTTGTTAAAGATTTTTGGGAGCATGTCTTTGACTGTTCATTCACTACTAGTAAATTAACAAAAAGGTGTAATGGATCTGAAAACTTAAGTGATGTGCAAAACTCTTTTACAGATTTGACTGACTTAAGGTTCTCAAACAATATATATAAAGCTGTTTATGCGGTAGCTCATGCAATTAATAATCTACTCTTATGTGAGGAAAATAAAAGCTCCAGTCCTAACGCCTGTCTGAAACTTGCAGAAATAAAGCCTTGGCAGCTTTTAAACTCCTTGCAAAGTGTAAATTTCACAACACCTGGGGGAGAAAGTGTCTTCTTTGATAGCAACGGGGATTCACCAGCAAGATATGAGCTGATCAATCTTCAAAGTGTTGACAGAGGCAAAATAGATGTGGAAACTATTGGCTACTATGATGCCTCTCTGCCTAAAGATCAACGGTTCTCCATGAATAATGTTAAGGTAGTCTGGAGAGAAGGGGGAGATCAGGTGCCTGTATCTGTGTGCAGTACAAGCTGTCCCTCAGGCACCAGGAAAGCAATGCAGAAAGGAAGACCGGTCTGTTGTTATGACTGTATACTGTGCCCACCAGGAGAGATTAGTAACAAAACTGATTCAGTTGATTGCCTTAAATGCCCTGTTACACAGTGGTCAAATACAAGAGGAGATGCCTGTATCCCAAAAGAAATTGAATTTTTGTCATATGATGAAATAATGGGAATACTTTTGGGATTGTTTTCTTTAATTGGGGCATTTTTTACTATAGTAGTCACACTTATTTTCTATATTTACAGAAGCACACCTATAGTAAGAGCTAACAATTCAGAACTGAGTTTCCTACTGCTCTTCTCACTGACTCTGTGTTTCCTCTGTTCACTCACTTTCATTGGTCGTCCCACTGAGTGGTCGTGTATGTTGCGACACACAGCGTTTGGGATCACTTTTGTCCTCTGTATCTCATGTGTTCTGGGGAAAACATTAGTGGTGTTAATGGCCTTCAAAGCTACACTTCCAGGAAGTAATGTCATGAAATGGTTTGGGCCTCTTCAACAAAGACTCAGTGTTGTTAGCTTTACTTTTATACAGCTCCTTATATGTGCACTTTGGTTAATATTATCACCACCATTTCCATATATGAACATGAAGCATTACCAAGAAAAAGTTATTTTAGAATGCAATGTAGGTTCAGCTACTGGTTTCTGGGCTGTACTGGGTTATATTGGTCTCCTGGCTGTCCTTTGCTTTGTTTTGGCTTTTCTCGGAAGGAAACTACCTGACAATTTCAATGAGGCTAAATTCATCACATTCAGTATGCTCATATTTTGTGCTGTATGGATCACATTTATCCCAGCTTATATCAGCTCTCCTGGAAAGTTTATCGTAGCTGTGGAGATATTTGCTATTTTGGTGTCATCCTTTGGATTACTTTTCTGTATTTTCTTGCCAAAGTGCTATGTAATTTTACTGAGACCAGAAATTAACACTAAAAAGCATGTCATGGCGAAGATAAATAAA

>OlfCg1

ATGCGTATATTAATGCAAATAGTGTATCAAGCCATTTACATTTGTCAGTCAAAGTTCAGCTCAATCTTTGTGATGCTTCTTTTCCTTTATATATTCCTACATTTCACTCTAATTCGTACAAAAGCTGGAAATGGTCTTTGCCAAATGATGGGAGACCCCAAGTACCCACTATTTTCCAAGGATGGGGACATAACCATTGGAGCACTTTTTGCAATTCTCAGTAAAGAAACTTTACCTTCCTTTCAGTTTACACAAAAACCTCAGCTTCTGTCATGCTCCAGTGTGAATTTAAGGGATTTCCGGATGACTCAAATTATGATTTTTGCAATTGAAGAGATTAACAGAAGTAAAAGTTTGCTTCCAAATGTTTCTATTGGATACCGGATTTATGACACCTGTGGTTCAAGAATGTCTGCTATGAGTGCAACTATGGCATTGATGAATGGACCAGAGTTTACAGCAGATAAAATTTGTAATGGAGAATCACCTATACATGCTATTATAGGAGAAACAGAGTCTTCTGCCACAATCATTCTGTCAAAAACTACGGGACCTTTTAAAATTCCAGTGATAAGTCACTCAGCCTCATGTGAATGCCTGAGCAACAGAAAAATATATCCATCATTCTTCAGGACTATTGCTAGTGATTACCACCAAGGCAGAGCACTTGCTTTCATAGTCAAGCACTTTGGCTGGTCTTGGGTTGGAACTGTGAACAGTGACAATGATTATGGCAACAATGTTATGGCTATTTTTCTAAATACAGCTCAGAAAGAGGGGATTTGTGTTGAGTATTCTGTGAAATTTTATCGAACAGAGCCAGAAAAACTTAAAAAAGTTGTAGAGACTATTAAAAAAAGCACAGCAAAAGTTATTGTTGCTTTTGTTTCACTTGTTGAGATGGGCTTACTTATTGATCAGCTTAGCATTCAGAATATTACAGGCTTCCAAGTGATCGGAGGGGAGGGATGGATAACTACAAAGAGTCTGATCAACTCAAAGAGTTTTCATGTGCTGGGAGGGTCACTGGGATTTGCATTTAGAAAAATCATTATTGAAGGATTTGCAGATTATGTTATAAAAACATTTTGGGAAAGAGATTTTCCATGTTTAACGAATGAGGGGAATTATTCTCAGTATGCATTAACTTGCAGCAGTTATCAGGATCTATTTACTCTAAAACACTACAATGAAGATATACCTGAACAAAGATATGCAAGCAATGTCTACAAAGCAGTGTATGCTGTGGCTCATTCACTGCATAATCTATTAAAGTGCAAAGAAAACGAAGGTTGTGAGAAAAGCTTGACAATGCAACCACAGCAGGTGGTTGAAGCTCTGAAAAACGTGAATTTTACCTTAAAATTTGGAGATCGTGTCTGGTTTGACAGTACTGGAGGGGCAGTAGCCCACTATGAAGTTGTCAACTGGCAGCAAGACTCCGATGGATCGTTCCAGTTTAAAAAAGTGGGTTACTTTGATGCCTCACTGCCACCTGACCAAAGCTTTATGCTTAACACTAAAAATATAATTTGGTCTGGAGGGCAACTTGAGAGACCGAGGTCTGTGTGCAGTGAGAGCTGTCCTCCAGGTACCAGGAAGGCTTCACAGAAAGGAAGACCTGTCTGCTGTTATGACTGCATTCCATGTGCAGAAGGAGAAATCAGTAATGAAACGGATTCAGTTAACTGCAAGCAGTGTCAAGGGGAATACTGGTCTAATGTCGAGAAAAACAAATGTGTGTTAAAAGCTGTAGAGTTCCTGTCATTCACAGATTTTATGGGTATAGTTCTTGTCTTTTTCTCACTGTTTGGAGTAGGATTAACTGTGCTGGTGGCTATCCTATTTTACAGCAAGAAAGACACTCCAATAGTTAAAGCCAATAACTCAGAGTTGAGCTTCCTGCTGCTCTTATCACTGACTCTGTGTTTCCTCTGTTCACTTACCTTCATTGGTCGGCCAACTGAGTGGTCTTGTATGTTGCGTCATACAGCGTTTGGGATCACCTTTGTCCTCTGTATCTCCTGTGTTCTCGGGAAAACAATAGTGGTGTTAATGGCTTTCAAGGCTACACTTCCAGGAAGTAATGTGATGAAATGGTTTGGTCCTGTACAACAACGACTCAGTGTTCTTGCCTTTACACTTATACAGGTACTCATATGTGTGCTTTGGCTAACAATGTCTTCTCCATTTCCTTACAAAAATATGAAATATTATAAGGAAAAGATCATTCTTGAGTGTAGTCTGGGCTCAGTTATAGGTTTCTGGGCTGTTTTGGGTTATATAGGTCTACTGGCTGCCTTGTGCTTCATTTTGGCTTTTTTGGCTCGTACGCTGCCTGATAACTTCAATGAAGCCAAATTCATCACATTCAGTATGCTCATATTTTGTGCTGTATGGGTCACATTTATCCCAGCTTATGTCAGTTCTCCTGGGAAATACACTGTAGCTGTTGAGATTTTTGCTATTTTAGCATCAACCTTTGGTTTATTATTCTGCATATTTGTCCCTAAATGTCATATTATCATTTTTAAACCTGAACAAAATACAAAACAACATATGATGGGGAAAAATTACATTCATACTGAGAAA

>OlfCg2

ATGCTTTTCTTTCTTCACACACTACCGCTTCTCTATCAACTTAATACAAACACCCCTTGTCAAACAATGGGAGACCCTAAAGATCCTCTGTTTTCGAAGGATGGAGATGTAACTATTGGAGGAGTTTTTGCAATACACAGAAAGGAAACATTGCCTTCTTTTGAGTTTGCACAAAAACCACTACCTCTGTCATGCTCCAGTGTGAATTTGAGAGATTTTCGGCTGGCACAAATAATGATTTTTGCAATTGAGGAGATAAACAAAAGTAAAAGTTTGCTTCCAAATATTTATATTGGATATCGAATTTATGATACCTGTGGTTCAAGGCTATCTACTATGAGTGCAACAATGGCAGTACTGAGTGGTCAGGAGTTTAGGCCAAGAGACAGATGCAATGATCAGCCTCCTTTACATGCTATTCTCGGTGAATCAGAGTCTTCTGCCACAGTAATTTTGTCCAGAACTACAGGACCTTTCAAAGTTCCAGTGATAAGTCCTTCAGCCACATGTGAATGTCTCAGCAATAGGAAAGATTACCCCTCTTTCTTTAGGACTATTGCTAGTGATTACCACCAGAGCAGAGCACTTGCATACATAGTCAAGCACTTTGGCTGGACTTGGGTGGGAGCTGTGAGCAGTGACAATGACTATGGAAACAATGGAATGGCCATATTTCAAAAAATTGCACAGGAGGAAGGGATTTGTGTTGAATATTCTGTGAAATTCTACCGAACAGAGTCTGATAAACTCAAAAAAGTGGTAAATGAAATTAAAAAAGGCACAGCGAAAGTAATTGTTGCATTTGTTTCATTTGTTGAGATGGGCTTACTTATTGATCAATTGAGTATTCAGAATATTACAGGCTTCCAGCTGATTGGTGTGGAATCATGGATAACTACAAAAAATTACTTCACTCCAAATAGTTTTTCCTCACTGAGGGGATCACTGGGATTTGCAGTGAGAAAAATCAATATTGAAGGGTTTATAGATTATGTTATAAAAGCATTCTGGGATACAGCTTTTCCATGCACCAAGACTGAGGGTAATTCTTCTCAATATTCAATAAGTTGCACCAGATCTGAGGACTTACTTGAGCTAAAAAACTACACTGAAGATGTGCTTGAACAAAGATATGCAAGCAATGTCTATAAAGCAGTTTATGCAGTAGCTCATTCATTACACAGTCTATTGGGGTGCGAAAACCAAGGATTGTGTGATAAAAATAAGCCAATACGCCCGCAACAGATGGTTGAAGCCCTACAAAAGGTTAATTTCACCATACAAATGGGAGATCAGGTGTGGTTTGACAGCACTGGTGGTACGGTGGCCCATTATGAAGTTGTAAACTGGCAGCAGGACTTTGATGGTTCATTCCAGTTTAAAACAGTGGGATACTATGATGCTTCACTTCCCCCTCAGCAACGCTTAATGCTAAATACTAAAAATATAATTTGGGCTGGAGGACAGCAGAAGAAGCCAAATTCTGTATGTAGTGAGAGCTGTCCTCCAGGTACTAGGAAGGCTGCACAAAAAGGAAGACCTGTCTGCTGTTATGACTGCATTCCATGCGCAGAGGGAGAAATTAGCAATGAGACAGATTCAATTAACTGCAAGCAGTGTCTGGAGGAATACTGGTCTAATGCTGAGAAAAATAAATGTGTGTTAAAAGCTGTAGAGTTTCTATCATTCACAGAAATTATGGGTATAATACTAGTCTTTTTCTCACTATTTGGAGTAGTACTAACCACACTAGTGACTGTCCTGTTTTACAGGAAGAAAGACACTCCCATAGTAAAAGCCAACAACTCAGAGCTAAGCTTTCTATTGCTCTTTTCCTTGAGTCTGTGTTTCCTCTGTTCACTCACTTTCATTGGTCGACCCACCGAGTGGTCTTGTATGTTGCGTCACACAGTGTTTGGAATCACTTTTGTCCTTTGTATCTCCTGTGTCCTTGGGAAAACAATAGTGGTGTTAATGGCCTTTAAAGCTACGCTTCCAGGAAGTAATGTCATGAAATGGTTTGGGCCTGTCCAACAAAGACTCAGTGTTCTTGCCTTTACACTTATACAAATTATTATCTGTGTGGTTTGGATAACACTATCTCCTCCATTTCCCTACAAAAATATGGAATATTATAAGGAAAAGATTATTATAGAGTGCAATCTGGGTTCTACATTAGGCTTCTGGGCTGTTTTGGGTTATATCAGCCTACTGTCTGTCTTGTGCTTTGTTCTGGCTTTTCTGGCTCGGAAGCTGCCTGATAACTTTAATGAAGCCAAATTCATCACATTCAGTATGCTTATATTTTGTGCTGTATGGATTACTTTTATACCAGCTTATGTCAGTTCTCCTGGAAAATTGACTGTAGCTGTGGAGATATTCGCCATTTTGTCATCTAGCTTTGGTTTACTATTTTGCATTTTTGCACCTAAATGTTACATCATCCTGCTTAAGCCTGAACAAAATACAAAGCAACATATGATGGGAAAAACTTTAAAGTCATAT

>OlfCg3

ATGCTTCTCTTTCTTTACACACTCCTGCTTTTCCATCACCTTCACACTAAGGCTGGAAACACTCTTTGCCGAATAATGGGAGACCCTCAATATCCACTGCTTTCCACAGATGGAGACATAACTATTGGAGCTCTTTTTCCAGTCCACAGCACAGAAACATTGCCTTCATTTGAATTTACAAAACAACCTCAGCTTCTATCATGCTCCAGTGTGTACCTGAGAGATTTTCGTCTGGCACAAATCCTGATCTTTGCCATTGAGGAGATAAACAGAAGTGTACAATTGCTACCAAATATTTCAATTGGTTATAGAATCTATGATACCTGTGGTTCAAGACAGTCTACCATGAGTGCAATTATGGGACTAATGAATGGTCAGGAGTTTGGAGCAGGTGAGAGATGTAATGGACGGTCTCCTATACATGCAATCATTGGTGAATCAGAGTCTTCTGCAACAGTGATACTGTCCAGAACTACGGGACCTTTTAAAATTCCAGTGATAAGTCACTCAGCCTCATGTGAATGTCTCAGTAATAAGAAAGACTACCCATCATTCTTCAGGACTATGGCTAGTGATTACCATCAGAGCAGAGCACTTGCTTATCTAGTTAAGGACTTAGGCTGGTCTTGGGTGGGAGCTGTGAGCAGTGACAATGAATATGGAAACTATGGAATGTCCATATTTCTGAAAATAGCCCAAGAAGTGGGGATTTGTGTAGAATATTATGCCAAATTCTACCGAACAGAGGCAGAAAATCTCCATAAAGCAGTGAATATGATTAAAAAAGGCACAGCAAAAGTAATTGTTGCTTTTGTTTCACTTCCAGAAATGGGCATACTTGTTGATCAGTTAAGCATTGAGAACATTACAGGACTCCAAATGATTGGTGTGAAATCATGGATAACTGTAAAAAGTTTCATCACTCCAAGCAGCTTTCATGTGCTTAAAGGGTCATTGGGGATTGCGGTGAAAAAAATCAGTGTTGAAGGCTTTGCTGAATATGCCATTAACGAATTTTGGAAAAAAGGTTTTCCATGTTTAAAGAGTGAGGTTAATTCATCTCGATATGCATTAAGTTGTAGCAGATATGAGGATCTCTTTGTGCTAAAATCAATCAGTGAGGATGTGCCTGAACAAAGATATGCAAGCTACGTGTACAAAGCAGTTTATGCTGTGGCACATTCATTACACAGTCTATTCAATTGTAAAGAACACGAAGGTTGTGAGAAAGACCTGACAATACAACCACAGCAGGTTGTTGAGGCTTTAAAAAAGGTCAAGTTCACCATAAAAACAGGGGATCATGTGTGGTTTGACAGCACTGGTGCCGCTGTAGCCCAATATGATGTTGTAAATTTACAGCTGGACTCATATGGTTCAACACTTTTTAAATCAGTGGGATACTATGATGCTTCATTGCCCCCTGAACAGCAGTTTGTGCTAAACACTACAAACATCATTTGGGCTGGAGGAGAGCAGAAGGTGAGGTCCGTGTGCAGTGAAAGCTGTCCTCCAGGCACCAGGAAAGCTACACAGAAAGGAAGACCTGTCTGCTGTTATGACTGTATTCAATGTGCAGATGGAGAAATTAGTAATGAGACAGATTCGAATAATTGCAAACAGTGCCCAGAGGAATACTGGCCTAATACTGAGAAAAGTAAATGTGTGTTAAAGATTGTAGAGTTTCTTTCATTCACAGAGCTTATGGGCATCGTGTTAGTTATTTTTTCACTGTTTGGGGTAACATTGACTGTATTGGTGACCTTCCTGTTTTACAGCAAGAAGGACACTCCTATAGTAAAAGCCAACAACTCAGAGCTGAGCTTCCTGCTCCTCTTCTCACTGACTCTGTGTTTCCTCTGTTCACTCACTTTCATTGGCCGTCCCACTGAGTGGTCCTGTATGTTGCGTCACACAACATTTGGGATCACTTTTGTCCTCTGTATCTCTTGTATTCTGGGAAAAACAATAGTGGTGTTAATGGCTTTCAGAGCTACAATTCCAGGTAATAATATGATAAAATGGTTTGGTCCTGTACAACAACGACTCAGTGTTCTTGCTTTTACGCTTATACAGATTCTTATATGTGTGGTTTGGCTGACAATTTCACCTCCATTTCCAAGCAAAAACATTAAATATTATAAAGAAAAGATTATTCTTGAGTGCAGTCTGGGTTCTACTATAGGTTTCTGGATTGTTCTGGGCTATATTGGCTTGCTAGCTGTCTTGTGCTTCATTTTGGCTTTTTTGGCTCGCACATTGCCTGACAACTTTAATGAAGCAAAATTCATCACATTCAGTATACTCATATTCTGTGCAGTATGGATCACATTTATTCCAGCGTATGTCAGTTCTCCTGGAAAATTGACTGTAGCTGTGGAGATATTTGCCATTTTAGCCTCGAGCTTTGGCTTACTTTTTTGCATATTTGCACCTAAATGTTACATTATCCTGTTCAAGCCTGAACAAAATACAAAGCAACATATAATGGGAAAAACAATATCCAAGAAC

>OlfCg4

ATGCTTCTGTTTATTTATATATTGCTGCTTTTCCACCAGCTTCATACAAAAGCAGGAAACACCCAGTGCCGGATAATGGGAGACTCTACTTACCCTCTGATTTCAAAGAATGGAGACATAACCATTGGAGCACTTTTTCCAATACACAGCACAGAGACTTTAACTTCTTTAAAATTTACTCGAAAACCTCAGCCTATATCATGTTCAAGTGTAAATCTAAGAGATTTTCGACTGGCAGAGATTATGATCTTTGCAATTGAGGAGATTAACAAAAGTGAAAGTTTGCTCCCAAATGTAACTATCGGTTATCAAGTCTATGATACATGTGGTTCAAGACTGTCTACCATGAGTGCAATTATGGGATTAATGAATAGTCAGGGATTTGATGCTGAAGATGGATGCAAAGGGCAAACTCCAATACAAGCTATTATAGGAGATTCAGAGTCCTCTGCCACAGTGATTCTCACCAGAACTACAGGACCTTTTAAAATTCCAGTGATAAGTCATTCAGCTTCATGTGAATGCCTAAGTAATAGGAATGATTACCCTTCATTCTTCAGGACTACTGCTAGTGATTACCATCAGAGTAGAGCCTTAGTGCATATAGTCAAGCACTTAGGATGGACTTGGTTGGGTGCTGTAAGCAGTGACAATGACTATGGAAACTATGGAATGTCCATATTTCAGAAAATTGCCCAGGAAGAAGGGATTTGCATGGAATACTCTGTAAAATTTTATCGAACAGAACAAGAAAAACTTCAGAAAGTGGTAGAGACAATGAAAAAAGGCACTGCAAAAGTGATTGTAGCATTTGTTTCATTTCTTGAAATGGGCTTACTTATTGATCAATTAAGTGTTCAGAACATTACAGGCCTTCAAATGATTGGTGTGAAATCATGGATAACTTCAGAGAATTATATCACTCCAAAAAGTTTTCATGTGCTGGGAGGGTCACTGGGGTTTGCTATGAGAAAAATGTATATTGAAGGGTTTTCGGATTTTGCATTAAAAACATTTTGGGAAAAACACTTCCAATGCTCACAAACCAAGCCAAATGCTTCTAATTATGCATCAAGTTGTAGCAGATATAATGATTTACTCATGCTGAAAAATTACAAGGAAGATGTGACTGAACACAGATATTCAAGCAACGTCTATGAAGCAGTTTACGCAGTAGCTTATTCATTGCATAGTCTACTAAAGTGCAAAGAACGAGTAAAGTGTGAGAAAGGCATGACAATACAACCACAGCAGGTAGTTGAGGCTCTGAAGAAAGTAAATTTCTCTGTAAAGTTTGGAGATCGTGTGTGGTTTGACAGTACTGGTTCCACAGTAGCCCAATATGAAGTTGTGAATTGGCAACGCATCTCTGATGAATCATTCCAGTTTAAAACTGTGGGATACTATGATGCCTCATTGCCCCCTAACCAGCGTTTTTTGCTCAATACTGAAAATATAATCTGGGCTGGAGGAATGTTGGAGAAGCCAAGATCTGTGTGCAGCGAGAGCTGTCCTCCAGGCACTAGGAAAGCTTCACAGAAAGGAAGACCTGCCTGCTGTTATGACTGTATTCCATGTGCAGAAGGCGAAATCAGTACTGATACAGATTCAAATAACTGCAAGCAGTGTCCAGGGGAATACTGGTCTAATGATAAAAAAAATAAATGTGTGCTAAAGGCTGTAGAGTTTCTATCATTTACAGAAGTTATGGGTGTATTGTTACTTTTTGTTTCACTGTTTGGAGTAGGATTAACAACACTGGTGGCCATTCTGTTTTACAACAAGAAGGATACTCCCATAGTAAGAGCCAACAACTCGGAGTTGAGCTTCCTGCTGCTCTTCTCATTGATTCTGTGTTTCCTCTGTTCAATTACTTTCATTGGTCGCCCCACTGAGTGGTCCTGTATGTTGCGTCACACAGCATTTGGGATCACATTTGTCCTCTGTATCTCGTGTGTTCTGGGAAAAACAATAGTTGTGTTAATGGCCTTCAAGGCTACACATCCAGGAAGTAATGTCATGAAATGGTTTGGGCCTGTACAACAAAGACTCAGTGTTCTTGTCTTTACATTTATACAGGTAATTATCTGTGTGCTTTGGCTAACTATATCACCTCCATTTCCCTACAGAAATATGAAATATTATAAAGAAAAGATCATTCTTGAGTGTAGTCTGGGTTCCATTGTAGGTTTCTGGGCTGTGCTTGGTTATATAGGCCTACTGGCTGCCTTGTGCTTCATTCTAGCTTTTCTAGCTCGGAAGCTGCCTGATAACTTTAATGAAGCCAAATTCATCACATTCAGTATGCTCATATTCTGTGCTGTATGGATCACATTTATCCCAGCTTATTTCAGTTCTCCTGGTAAATTAACGGTAGCTGTGGAAATATTTGCCATTTTGGCATCAAGCATTGGTTTACTATTTTGCATTTTTGCTCCTAAATGTTATATTATTTTGTGTAAGCCTGAACAAAATACAAAGCAACACATAATGGGAAAAAACAAC

>OlfCg5

ATGCTTATCTTGCTTTCTATATTTTTGCTTTTTAATGAACCTTTTACAAAGGCAGAAGACACTATTTGTCAAAAAATGGGAGATCCCAAGTATGCGTTGTTTTCAAAAGTTGGAGACGTAACTATTGGGGGTATTTTTTCAATCCGCAGTGTAGAAATATTACCTTCATTGGAATTTACACAAAAACCCCAGCTTCTGTCATGCTCCAGTGTTAATCTAAGAGATTTCCGGATGGCTCAAATTATGATCTTTGCCATTGAGGAGATTAACAGAAGTGAAAGTTTGCTTCCTAATGTTTCAATTGGTTACAAAATTTATGATAGCTGTGGTTCAAGACTATCTTCTATTAGTGCAACCATGTCACTGATGAATGATCAGGAGTTTCCAAAAGGAAACATATGCAATGGACAATATCCAATACATGCTATCATAGGAGAAACTGAGTCTTCTGCCACAGTGATTCTGTCCAGAACTACAGGACCTTTCAAAATTCCAGTGATAAGTCACTCAGCCACATGCGAATGTCTCAGCAATAGAAGAGATTACCCATCATTCTTCAGGACCATTGCTAGTGATTATCACCAGAGCAGAGCACTTGCATACATTGTAAAATACTTTGGGTGGTCTTGGGTGGGAGCTGTGAACAGTGATAATGACTATGGAAACAATGGAATGGCCATATTTCTTAAAACAGCACAAGAGGAAGGGATTTGTGTGGAGTACTCTGTTAAATTCTACCGAACAGAGCCGGAAAAGCTTCAAAAAGTCGTTGAGACTATGAAAAGAAGCACCACAAAAATAATCATTGCATTTCTATCTCGTGTTGAGATGGTCAATCTACTTGAGGAACTAAGCATTCAGAACATTTCAGGCTTTCAAGTAATTGGTGTGGAAGCATGGACAACTGCAAAGAGTTTGATCACTCCAAACAGTTTTCATATTCTTGGAGGGTCACTGGGGTTTGCAGTGAGAAAAATTGATACTGAGGGTTTTTTAGATTATGTTATAAAATCATTCTGGGACTCAGCTTTTCCATGCTTAATAGTGAATTCTTCTCAATTAAAAGTTAACTGCAGTAGTTATAAGGACCTTCTTGTGCTGAAAAATGACAATGAAGATGTGCCTGAGCAGAGATATGCTAGCAATGTCTACAAAGCAGTTTATGCTGTAGCTCATTCATTGCACAGTCTGCTCAACTGCAAAGAAAAAAAAGGTTGTCAAAAAAACCTCACAATACAACCGTACCAGATGGTTGAGGCTCTAAAGAAGGTCAATTTTACAATAAAATTTGGGGATCGTGTATGGTTTGACAGTACTGGTGCCACAATTGCCCAATATGAGGTTGTGAACTGGCAGCAGGACTCTGATGGATTAATACAATTTAAACCAGTGGGATACTATGATGCTTCATTGCCCCCTGACAAGCGTTTTGTTGTTAAAACTGAAAACATAATCTGGGCTGGGGGAAAGTTAAAGAAGCCAAAGTCTGTGTGCAGTGAGAGCTGTCCTCCAGGCACCAGGAAGGCTGCACAGAAAGGAAGACCTGTCTGCTGTTATGACTGTATACCATGTGCAGAGGGAGAAATCAGTAACGAGACAGATTCAGTTAACTGCAAGCAGTGTCCATTGGAATACTGGTCTAACACTGAGAAAAATAAATGTGTGTTCAAATCTGTAGAGTTTTTGTCATTCACAGAAGTTATGGGTGTAGTGTTAGTCTTTTTCTCACTGTTTGGAGTAGGATTAACTATGCTGGTGGCCATTCTCTTTTACAACAAGAAAGATACTCCCATAGTAAAAGCCAACAACTCAGAGTTGAGCTTCCTGCTGCTCTTCTCACTGACTCTGTGTTTCCTCTGTTCACTTACTTTCATTGGTCGCCCAACTGAGTGGTCCTGTATGTTGCGACACACAACATTTGGGATCACTTTTGTCCTCTGCATCTCCTGTGTTTTAGGGAAAACAATAGTGGTGTTAATGGCCTTCAAAGCTACACTTCCAGGAAGCAATATAATGAAATGGTTTGGGCCTGTACAACAAAGACTTAGTGTTCTTGCCTTTACACTCATACAGGTTATCATCTGTGTGCTTTGGTTGATAATATCACCTCCATTTCCCTACAAAAATATGAAGTATTATAAGGAAAAGATCATTCTTGAATGCAGTCTGGGTGCTACAATATGTTTTTCTGCAGTTTTATTTTACATTGGCCTTCTGGCTATCTTATGTTTCATTTTGGCTTTTCTAGCACGTAAGCTCCCTGATAACTTCAATGAAGCCAAATTCATCACCTTTAGTATGCTTATATTCTGTGCTGTATGGATCACATTTATTCCGGCTTATGTTAGTTCTCCTGGGAAATTTACTGTTGCTGTGGAGATATTTGCCATATTGGCCTCAAGCTTTGGTCTACTATTTTGTATATTTGTGCCAAAATGTTATATAATTCTGTTCAAAAATGAACAAAATACAAAACAACATATAATGGGGAAGATAAAG

>OlfCg6

ATGTTTCTTTTTTACACAATCCTGCTTTTCCATCTTCTTCATACAAAGGTGGAAAACTCTCTTTGCAGAATAATGGGAGACTCCAATTATCCACTGTTTTCAAAGAATGGAGATGTTAGCATTGGAGGAATTTTTGCTATCCATAGAAAAGAAACATTACCTTCATTTGAGTTCATGCAAAAGCCTCAGCCTCTGTCATGCTCCAGTGTGAATCTGAGAGATTTTCGGCTGGCGCAAACTATGATTTTTGCCGTTGAGGAAATTAATAGAAGTAAAAGTTTGCTCCCAAATGTTTCTATTGGCTACAAGATTTATGATACATGTGGTTCAAGACTGTCCACCATGACTGCAATTATGGGACTGATGAATGGTCAGGACTTTTCAACAGAGGATAGATGCAATGGACAGTCACGATTGCATGCTATCATAGGAGAATCAGAGTCATCTGCCACAATAGTTTTGTCCAGAACAACAGGACCTTTTCGGATTCCAGTGATAAGTCATTCCTCATCATGTGAATGTCTCAGTAATAAAAAAGATTATCCTTCATTCTTCAGGACTATTTCTAGTGATTACCACATGAGTAGAGCACTTGTCTATATAGTTAAGCATTTAGACTGGTCTTGGGTTGGTGCTGTGAACAGTGACAATGATTATGGCAACAATGGGATGGCCATATTTCTGAAAGCAGCCCACGAGGAGGGAATCTGTGTGGAATACTCGGTTAAATTCTACAGAACAGAGCCAGAAAAGCTTAAAAAAGTGGTTGACACTATAAACAAAGGCACTGCTAAAGTAATTGTAGCATTTGTTTCATTTGTGGAGATGGGCTTACTTATTGATCAATTAAGTATTCAGAACATTACAGGCATCCAAATGATAGGTGTGGAACCATGGATAACTGCAAATACATATATCACTTCAAACAGTTTTCGTGCAATGGGAGGTTCGCTGGGGTTTGCAACAAAATATATTTATATTGAAGGATTTGCTGAATATGTTATGACACCATTCTGGAATACAGCTTTTCCATGCTCAGAGAGTGATAGGAATCATTCTCATTATGAATTAATTTGCAGTAGGTATGAGGATCTGCTTGCTCTGAAAAATGACAATAAAGACGTGAATGAACACAGATATTCAAGCAATGTCTACAAAGCAGTTTATGCAGTAGCTCATTCATTGCATGGCCTACTTAACTGCAAAGAACAAGAAGGGTGTGAGAAAGGCCTGACAATACAACCACAGCAGGTGGTCAAGGCATTGAAAAATATTAATTTCACAATTAAGTCAGGTGACAGTGTATGGTTTGACAACACTGGCAGCGTAGTGGCCCTATATGAAGTTGTGAACTGGCAAAAAGACTCAGATGGATCATTTCAGTTCAAATCAGTGGGATTTTATGATGCTATGATGCCTCCATACAAGAATTTAAGGCTTAACACTAAAAACATAGTCTGGGCTGGAGGACAGCTGGAGAAGCCAAGGTCCGTGTGCAGTGAGAGCTGTGCTCCAGGCACCAGAAAGGCTGCACAGAAAGGAAGACCTGTTTGCTGTTATGACTGCATTCCATGTGCAGAAGGAGAAATCAGTAATGAGACAGATTCAATAAACTGCAAGCAGTGTCCAGGGGAATACTGGTCTAACACTGAGAGAAATAGATGTGTTATAAAGGCTGTAGAATTTCTGTCATTCTCAGAAGTTATGGGTATAGTGTTAGTAATTTTCTCACTCTTTGGAGCAGGATTAACTGTGCTCGTTGCCGTTCTGTTTTACAGCAAGAAGGACACTCCCATTGTAAAAGCCAACAACTCAGAGCTGAGCTTCCTGCTACTTTTCTCACTGACTCTGTGCTTCCTCTGTTCTTTAACATTCATTGGTCAGCCAACTAAATGGTCCTGTATGTTGCGTCACACAGCATTTGGCATCACTTTTGTCCTGTGTATCTCATGTGTTTTGGGTAAAACAATAGTAGTGTTAATGGCCTTTAAGGCTACACTCCCAGGAAGTAATGTGATGAAATGGTTTGGTCCTGTACAACAACGACTCAGTGTTTTTGCTTTTACACTTATACAGGTACTTATTTGTGTGCTTTGGCTAACTATATCTCCCCCATTTCCATATAAAAACATGAAGTATTATAAGGAAAAGATCATTCTTGAGTGCAATCTGGGTAATACTATAGGTTTCTGGGCTGTGCTTGGTTACATTGGCCTCCTGGCTGCCCTGTGCTTTCTTCTAGCTTTTCTGGCTCGTAAGTTGCCTGATAATTTCAATGAAGCCAAGTTCATCACTTTCAGTATGCTCATATTTTGTGCTGTATGGATCACATTTATTCCAGCTTATGTCAGTTCTCCTGGAAAATTTACTGTAGCTGTGGAGATTTTTGCCATTTTATCCTCAAGCTTTGGGTTATTAGTTAGCATTTTTGCACCTAAATGTTTTATTATCCTCCTTAAGCCTGAACAAAATACAAAGCAACATGTGATGGGAAAAACAACTTGCAGTCTT

>OlfCg7

ATGTCAGCTAATTCACTTTTCATGGTCAGCTTAACCTTTAGAATGATTCTCTATTTTTACACTATTCTTTTTTTACGCTGCTTTCAAGCAAAGACAGAAAACGCATTTTGCCAAATAATAGGAGAGGCTAAATACCCACTACTTTCAAAGGATGGAGACGTAACTATTGGGGGAATTTTTGCAATCCACAGTAAAGAAACATTACCTTCATTTGAGTTTCAACAAAAACCTCAGCCACTATTATGCTCAAGTGTGAATCTAAGAGATTTTCGGCTGGCACAAATAATGATCTTTGCTATTGAGGAAATTAACAAAAGTGAAAATTTGCTGCCAAATGTTTCTATTGGCTACCGAATATTTGATACCTGTGGTTCAAGACTGTCATCTATGAGTGCAACAATGGGACTAATGAATGAACTGAAATTTGCAGCAGGGGAGACATGCAATGGACAGTCTCCCATACATGCTATCATTGGAGAAACAGAGTCTTCAGCCACAGTGATTCTGTCCAGAACTACAGGACCTTTTAAAATTCCAGTGATAAGTCATTCAGCATCATGTGAATGTCTCAGCAACAGGAATGATTACCCATCATTTTTCAGGACTATTTCTAGTGATTACCACCAGGGAAGAGCACTTGCATACATAGTCAAGTACTTAGGCTGGTCTTGGGTGGGAGCTGTGAACAGTGACAATGACTATGGAAATTATGGAATGGCCATATTTCTCGAAACAGCCCAGAAAGAGGGAATTTGCGTGGAGTATTCTGTAAAATTCTACAGAACAGAGACAGAAAAACTAAAAAGAGTGGTAGACACAATTAAAAAAAGCACTGCAAAAGTGATTGTTGCATTTGTTTCATTTATTGAGATGGGCTTATTAATTGAACAGCTAAGTATTCAGAATATTACAGGATTCCAAATAATTGGAGTAGAGGGATGGATAACTTCAAAGAACTACATAACCACAAACAGCTTTCATTCAATGGGAGGTTCACTGGGATTGGCATTGAGAAAAATCCATTTGGAAGGGTTTTTAGATTATGTAACTAAATCGTTTTGGAGCACAGCATTCCCATGCTCACAGACTGAGGGGATTTTGCCTACTGTAGGTTGCAGCAAATATAAGGATCTACTTCCACTGAAAAACTACACTGAAGATGTGCCTGAACACAGATATTCAAGCCATGTTTATAAAGCAATTTATGCAGTAGCTCATTCACTACATAGTCTGCTCAAATGCAAGGAAGGAGAAGATTGTGAGAAAGGCCACGCAACACAACCACAGCAGGTGGTTGAGGCTCTAAAAAAAGTTAATTTCACTGTAAAGTTTGGAGATCGTGTGTGGTTTGACCGTACTGGTGCCACAGTAGCCCATTATGAGGTTGTGAACTGGCAGCAGGACACTGATGGATCATTCCAGTTTAAACAAGTAGGATACTATGATGCCTCACTGCCTCCTGACCAGCGCTTTGTTCTCAACATTGAAAGCATCATCTGGCCTGGAGGAAATTTGGAGAAACCAAGGTCTGTGTGCAGCGAAAGCTGTTCTCCAGGCACCAGAAAGGCTGCTCAGAAAGGAAGACCTGTCTGCTGTTATGACTGTGTTCCATGTGCAGATGGAGAAATTAGTAATGAGACAGATTCGGTTAACTGCAAGCAGTGTCCAAGGGAATACTGGTCTAATGGTGAAAAAAATAAATGTGTGTTAAAAGCTATAGAGTTTTTGTCATTCACAGAAATTATGGGTATTGTGTTGGTCTGTTTTTCACTGTTTGGAGTAGGATTAACTGCAGTCGTGGCCATTCTGTTTTGGAGCAAAATGGACACTCCCATTGTAAAAGCCAACAACTCAGAGCTAAGCTTCCTGCTGCTCTTCTCACTGACTCTTTGTTTCCTATGTTCACTTACGTTTATTGGTCGGCCCACTGAGTGGTCCTGTATGTTGCGTCATACTGCCTTTGGGATCACTTTTGTCCTTTGCATCTCCTGTGTTTTAGGGAAAACAATAGTGGTGTTAATGGTTTTCAAGGCTACACTTCCAGGAAGTAATGTCATGAAATGGTTTGGGCCTACACAACAACGACTCAGTGTTCTTGCTTTTACATTTATACAGGTACTTATATGTGTGCTTTGGCTTACAATATCACCACCATTTCCTAACAAAAATATGACATATTATAAAGAAAAGATTATTATTGAGTGCAGTCTTGGTTCTACTATAAGTTTCTGGGCTGTTCTTGGTTACATTGGCCTGCTAGCTGTCTTGTGCTTCATTTTGGCTTTTTTGGCTCGCAAACTACCAGATAACTTCAATGAAGCCAAGTTCATCACATTCAGTATGCTTATTTTCTGTGCTGTATGGATCACATTTATTCCAGCTTATGTCAGTTCTCCAGGAAAATTCACAGTAGCTGTGGAAATATTTGCTATCTTAGCCTCTAGTTTTGGCTTACTTCTCTGCATATTTGCACCTAAATGTTACATAATCATATGTAAGCCTGAACAAAATACAAAGCAACATGTCATGGGAAAAACATCTTTAAAGGTTCAA

>OlfCg8

ATGTCTATAAAAGTAGTAAAAAGCAGTATCCATTCACTTATACTTGGCAGTATAACTCATCAGAACCTGGTGTCTGGGATGCTTCTATTTTTATACTCAGTCCTCTCTTTGTATCACCTAAATACAAAGGCGGAAAACACTCTTTGCCAAATGATAGGCAACCCAAAGTATCCACTGCTTTCCAAAGATGGAGATATCACAATCGGTGCACTTTTTGTAATCCATTCTGAAGTATCACTACCTTCATTTGCGTTCACACAAAAGCCTAATCTTCTATCATGCTCCAGTCTAAATATTAAAGATTTCAGGTTAGCTCAAACCATGATCTTTGCCATTGAAGAAATTAACAAAAATACAAATTTACTCCCAAATATTTCTGTTGGCTATCAAATTTATGACACCTGTGGTTCCCGATTCCATTCTATGAGTGCAACTATGGCATTAATGAATGGTCCAAAGAATTCAGAAGGATACACATGCAATGAGCAGTCGTCTGTACATGCTATCGTAGGAGAAACTGAATCCTCCAACACAATAATTCTGTCCAGAACCACAGGTCCTTTCAAAATTCCAGTGATAAGTCCCACAGCCACGTGTGAGTGTCTCAGTAATAGGAAAGAATTTCCATCTTTTTTCAGAACTATTGCAAGTGATTACCACCAGAGCAGAGCACTTGCATACATAGTAAAGCATTTTGGCTGGACGTGGGTGGGAGCTGTGAACAGTGACAATGACTATGGAAATAATGGAATGGCCACATTTCAGAAAACAGCCAAAGAAGTGCAGATTTGTGTGGAGTACTCTGTAAAATTTATTAGAACAGAGACTGAAAAAATTAGAAATGTGGTAAATGTTATAAAAAAGGGCACAGCTAAAGTAATTGTTGCATTTCTCACTGGTTTTGAGATGAAAAGTCTACTCGAACAGCTAAGTAGTCAGAACATTACAGGCCTTCAAATGATTGGTGTGGAGGCATGGATAACTTCAAAGACTTTAATAACGTCAAAAAGTTTTCATGTGCTAGGAGGGTCACTGGGGTTTGCAGTGAGAAAAATCCAAATTGAAGGGTTTGCAGATTATGTTATGAAAGCATTCTGGGACACAGCTTTTCAAAGCTTTTCTAATGCTGATTTAAATTACAGTCAATATCAGGATCTACTTTTAGTAAAAAACTACAATGAAGATGTGCTAGAGCAAAGATTTGTGAGCTACGTCTACAAAGCAGTTTATGCTTTGGCTCATTCACTACACAGTCTACTCAGATGTACAGAACAAGGAGGTTGTGGGAAGGCCCTGACAATACAGCCACATCAGTTGGTCGAGGCTCTAAGAAAGGTGAATTTCACCGTAAAGATGGGCGATCAGGTGTGGTTTGACAGCACAGGTGGTGTAATAGCACAATATGATGTAGTGAACTGGCAGCAGAATTCTGATGGTTCAGTTCAGTTTCAATCAGTGGGATACTATGATGCATCACTGTCCCCTGACCAGCGCTTCATGCTCAACACTGAAAAAATAGTCTGGGCAGGAGGACAACTGGAGAAGCCAAGGTCTGTGTGCAGTGAGAGCTGTCCTCCAGGCACCAGGAAGGCTTCACAGAAAGGAAGACCTGTCTGCTGTCATGACTGTATTCCATGTGCAGATGGAGAAATCAGTAATGAGACAGATTCAAATAACTGCAAGCAGTGCCCTGGGGAATACTGGTCTAATGCTAACAAAAATAAATGTGTTACAAAAGCTGTAGAGTTTTTGTCATTCACAGAAGTTATGGGTATAATTTTAGTCTTTTTCTCACTGTTTGGAGCAGGATTAACTGTGCTGGTGGCTATTCTCTTTTACAGCAAGAAGGACACTCCCATAGTAAAAGCCAACAACTCAGAGCTAAGCTTCCTCCTGCTCTTCTCACTGACTCTGTGTTTCCTCTGTTCAATTCCTTTTATTGGTAGGCCCACTCAGTGGTCCTGTATGTTGCGTCACACTGCATTTGGAATCACTTTTGTCCTCTGTATATCCTGTATTCTAGGGAAAACAATAGTGGTGTTGATGGCCTTCAAAGCTACGCTACCAGGAAGTAATATCATGAAATGGTTTGGTCCTGTACAACAACGACTCAGTGTTCTTGCTTTTACACTTATACAGGGTCTGATTTGTGTGATTTGGCTTACAAAGTCTCCTCCATTTCCATACAAAAATATGAAATATTTTCAAGAACAGATCATTCTTGAATGCAGTGTGGGTTCCACTATTGGGTTTTGGGCTGTTCTAGGTTATATTGGCCTACTAGCTGTGTTGTGCTTCATTCTGGCTTTTCTGGCTCGCACACTACCTGATAACTTCAATGAGGCCAAATTCATCACATTCAGTATGCTTATATTCTGTGCTGTATGGATCACATTTATTCCAGCTTATGTCAGTTCTCCTGGAAAATTGACAGTAGCTGTGGAGATATTTGCCATTTTAGCCTCAAGCTTTGGTTTACTTTTTTGTATATTTGCACCTAAATGTTATATTATTCTGCTTCAGCCTGAACAAAATACAAAGCAACAGATGATGGCAAAAACACCATCTAAAATT

>OlfCg9

ATGCAGTTGAGCTCATTTTTCAAGATGCTTCTCTATCTTTGCACGCTTCTGCTTTTTTTTAAGCTTAAAGTAAGTGTGGGAAATGCTTCTTGCAAAATAATGGGAGAACCGAAGTACCCTCTGCTTTTCAAGGATGGAGATGTGACTATTGGGGCACTTTTTCCAGTCCACAGCATAGAGACAGCACCTTCATTTGAGTTCACACAAAAACCTCAGCTTTTATCTTGCTCCAGTGTGAATCTGAGAGATTTTCGTCTGGCTCAAATCCTGATCTTTGCTATTGAGGAGATAAACAGAAGTGAAAGCTTGCTCCCAAAACTTTCCCTTGGCTACAAGATTTATGATACCTGTAGCTCAAAATTGTCTTCCATGAGTGCAACTATGGCATTGATGAATAGTTTTGAATTTGCAGGAAGAGACAAATGCAACGGGCAGACTTCTGTACATGCTATCATTGGAGAAACAGAGTCTTCTGCCACAGTGATCTTGACCAGAACCACAGGACCTTTCAAAATTCCAGTGATAAGTCATGCAGCCTCCTGTGAATGTCTGAGTAGCAGGAAAGAGTATCCGTCATTCTTCAGGACTATTTCTAGTGATTACCATCAAGGTAGAGCACTTGCATACATTGTCAAGCACTTGGGCTGGTCTTGGGTGGGAGCTGTGAACAGTGACAATGACTATGGGAATTATGGAATGGCCATATTTCTTAATACAGCCCATAAAGAGGGAATTTGTGTGGAGTACTCTGAGAAATTCTACAGGACAGAGCCTGAGAAACTAAAAAAGGTGGTAGACACAATTAAAAAGAGCACTGCAAAAGTTATTGTTGCATTTATTTCATTTCTTGAAATGAGATTACTTGTTGAGCAGTTAAGTGCTGAAAACATTACAGGCCTCCAAATAGTTGGTGTGGAGGGATGGACAACTTCAAAGAGTTTGATTACTCCAAAAACATTAAATGTGCTGAGAGGGTCACTGGGTTTTGCAATGAGAAAAATTTATATTGAAGGCTTTGCAGAATATGTTTTAAAACCCTTCTGGGACACAGCTTTTCCATGCATACCGAATCAGAGGAATGATTCTTGGGTCATATTAAATTGCAGCAGATATCAGGATCTTCTTGTTCTGAAAAATTACATTGAAGATGTCCCTGAACATAGATTTTCAATAAATGTCTACAACGCAGTTTATGCTGTGGCTCATGCACTGCACAGTCTATTTAAGTGCAAAGAACAAGAGGGTTGTGAAAAAGACCTGTTGACACAACCACAGCAGGTAGTTGATGCTCTGAAAAAAGTCAATTTTACTGTAAAGATGGGAGATCGTGTGTGGTTTGACAGCACTGGTGCAACACTAGCCCAATATGAGGTTGTGAACTGGCAGCAGAACTCTGATGGATCAGTCAATTTTAAAAAAGTGGGATACTATGATGCCTCTCTGCCTCCTGACCAGCGCTTTGTGCTCAACATTAAAGAGATACTTTGGGCAGGAGGAAACCTGAAGGCAAATGGCTTAACATTTATTTGTATTTATTTAAAATCTGTGTGCAGTGAGAGCTGTCCTCCAGGCACCAGGAAGGCTGCACAGAAAGGAAGACCTATCTGCTGTTATGACTGTATTCCATGTGCAGATGGAGAAATCAGTAACGACACAGATTCAAATAACTGCAAGCAGTGTCAAGGGGAATACTGGTCTAATGCTGATAAAAATAAATGTGTTCAAAAGTCGGTAGAGTTTCTGTCATTTACAGAGGTTATGGGTATAGTGCTTGTTTTTTTCTCACTATTTGGAGCAGGATTAACTGTGCTGGTGGCCATTCTCTTTTACAGCAAGAAGGACACTCCCATAGTAAAAGCCAACAACTCAGAGTTGAGCTTCCTGCTGCTCTTCTCACTGACCCTGTGTTTCCTCTGTTCACTTACTTTCATTGGTCAGCCCACTCAATGGTCATGTATGTTGCGTCACACAGCTTTTGGGATCACTTTTGTCCTTTGTATTTCCTGTGTTTTAGGGAAAACATTACTGGTGTTAATGGCCTTCAAAGCTACACTTCCAGGAAGTAATATCATGAAATGGTTTGGGCCTGTACAACAACGACTAAGTGTTCTTGCTTTTACATTTATACAAGTTTTAATCTGTGTGTTATGGCTATCAATATCTCCACCATTTCCCCACAAAAATATGAAATATTATGAAGAGAAGATCATTCTTGAGTGCAGTTTAGGTTCTACTATAGGTTTCTGGGCTATTTTGGGATATATTGGCCTACTGGCTGCGTTGTGCTTCATTCTGGCTTTTCTGGCTAGGACACTTCCTGATAATTTCAATGAGGCCAAATTCATCACATTCAGTATGCTTATATTTTGTGCTGTATGGATCACTTTTATTCCAGCTTATGTTAGTTCTCCTGGAAAATTCACTGTAGCTGTGGAGATATTTGCCATTTTATCTTCAAGCATTAGTTTACTATTGTGCATTTTTGCACCCAAATGTTATATAATCCTTCTTAAGCCTGAACAAAATACAAAGCAATACATGTTGGGAAAAACAACATCTAAGTTCTAC

>OlfCg10

ATGACCTGCCTTATGTCATACCCCTTTGTCTGTGGTTACAATCAATCATCCGTATTAATTATATTTAGTTATTTAAAAACACTGTATCAGTTCAAACTGTGCTTGAAATTTTCAGTTGATCTCAATGAGGGATTAGGGATGGTTTCCTTTATCTGGACACTCCTGCTTGTTGTTCCTTTACAAGCAAAAGCAGAAAACTCGCTTTGCCGAATGATGGGACAAAATATTAATCCACTGATATCCAAAGAAGGAGAAGTAACCATTGGAGCACTTTTCCCAATCCATAGCATAGAAATATTGCCTTCATTTGAGTTTACAGTAAAACCTCAGCTGCTATCATGCTCCAGTGTAAATCTAAGAGATTTCAGAATGGCTCAGACAATGACCTTTGCCATTGACGAGATTAATAAAAATCAAAGTTTGCTTCCAAATGTTTCTATTGGTTATCGAATTTATGATACATGTGGTTCAAGACTTTCTTCTATGAGTGCAACTATGGCATTGATGAATGGTAAAGAATTTTCTGCTGAGGATAAATGTAACGGACAGTCTGCCATACATGCCATCATAGGAGAAACTGAGTCTTCTGCTACAGTGATTCTGTCCAGAACTACAGGACCTTTTAAAATACCAGTGATAAGTCACTCAGCCACATGTGAATGTCTTAGCAACAGGAAAAACCATCCCTCTTTCTTTCGAACTATTGCTAGTGATTACCATCAGAGCAGAGCACTTGCATACATAGTCAAGCACTTTGGCTGGTCATGGGTGGGAGCTGTGAACAGTGACAATGACTATGGCAATAATGGAATGGCAATATTTCTAAATACAGCTCAGGAGGAGGGGATTTGTGTTGAGTACTCAGAAAAATTCTATCGAACAGATCCTGAGAAACTTAGAAAAGTAGTAGACACAATTAAAAACAGCACTGCAAAAGTAATTGTTGCATTTCTTACCAGTTTGGAAATGGAAAATTTGCTTCAGGAACTAACTAAAGCTAACATTACAGGACTCCAAATTATTGGTGTGGAAGCGTGGATAACAGCAAACAGTTTGCTCACTCCAAACAGCTTTCGTGTTCTGGGAGGATCATTGGGGTTTGCAGTACCAAAGGTCAATATTCAAGGTTTTTCAAACTATGTCATAAAAGATTTCTGGGAAACAGCTTTTCCATGCTCAGAGACTGAGATAAATGTATCTCAATATTCATCATTAAGTTGCAATTCTTATGATGACCTGCTTTTGCTGAAAAATTACAATGAAGATGTGCCTGAACAAAGATATGCAAGCAATGTCTATAAAGCAGTTTATGCTGTGGCTCATGCATTACACAGTCTACTTAAATGTAAAGTAAATGAAGGTTGTAAGAAAGACCTGAAAATACAACCTCAGCAGGTAATGATCGCAATACAAATTGACAATGTAAGAGTGATTGCAAAAAGTGACTTGCTCTCCTGTACATCATTTTCTCAGGTTGTTGACACTCTAAAAGAAATAAATTTCACCATAAATATGGGAGATCGTGTGTGGTTTGACAGCACTGGTGCCACAATAGCCCAATATGAAGTCATAAACTGGCAGCAAGGCTCTGATGGATCAATCCAATTTAAAACAGTGGGATACTTTGATGCCTCACTGCCACATGACCAGCGCTTTGTTCTTAACACTGAAATAATGCTTTTAAAAATTAAAATTTTTAAATTTGTAAATCATACATCTTTCTATGTAGAACCAAAGTCTGTGTGTAGTGAGAACTGCCCTGCCGGTACCAGGAAGGCTGTACAGAAAGGACGGCCTGTCTGCTGTTATGACTGTATTCCATGTGCAGATGGAGAAATCAGTAATGACACAGATTCAGTTAACTGCAAACAGTGTCCAGGGGAGTACTGGTCTAATGCTGAGAAAAATAGATGTGTGTTAAAGACTGTAGAGTTTCTGTCATTCACAGAAGTTATGGGTATAGTTCTAGTCTTTTTCTCACTCTTTGGAGTAGGATTAACTGTGCTGGTTGCCATTCTATTTTACAGCAAGAAAGAGACTCCCATTGTAAAAGCCAACAACTCAGAGTTGAGCTTCTTGCTGCTCTTCTCATTAACTCTGTGTTTTCTCTGTTCACTTACTTTTATTGGTCGACCGACTGAGTGGTCCTGTATGTTGCGTCATACAACTTTTGGGATCACATTTGTCCTATGCATCTCATGTGTTTTAGGAAAAACAGTAGTGGTGTTAATGGCCTTTAGAGCTACACATCCCGGAAAAGATATTATGAAATGGTTTGGTCCTGTACAACAAAGACTTAGTGTTATTGCACTTACCCTTATACAGGTTCTAATTTGTGTGCTTTGGCTAACAATATCTCCTCCATACCCCTACAAAAATATGAAATATTTTAAGGAAAAGACCATTCTTGAGTGCAATTTGGGTTCTACTATAGGTTTTTCTGCTGTCCTAGGTTACATTGGTTTGTTGGCTGTCCTGTGCTTTTTCTTGGCTTTTCTGGCTCGCACACTACCTGATAAATTCAATGAAGCGAAATTCATCACATTCAGCATGCTCATATTCTGTGCTGTGTGGATCACATTTATCCCATCATATGTCAGCTCTCCTGGAAAATTGACAGTAGCTGTGGAGATATTTGCAATTTTAGCTTCAAGCTTTGGTTTATTATTTTGCATTTTTGCTCCAAAATGTTATGTCATTCTGCTTAAACCAGAACAAAACACAAAACAACATATTATGGGAAAGATTTCATCAAAATCTTAC

>OlfCg11

ATGATGTCAGAATCACTCATCCATACTCATTATGGTTATAAAAGTTGTCATATTGTATTAAGTCAGGTGCATTTGGCTCCATTAAACAGAATTGTTTCAATGTCTGTCTTTCTATACACTGTGCTTATCTTTATATTTTATACAAAGGCAGAGAATCCTCTTTGTCAAATGATGGGAAATCCTAAATTTCCGCTGCTATCTAAGGATGGAGATGTAAATATCGGAGCAATTTTTTCAGTCCACAGCACAGAGATATTAACTTCATTTACATATACACAAAAACCCCAGCTTCTATCATGTTCAAGTGTGAGTTTAAGAGATTTTCGAATGGTTCAGACCATGATTTATGCCATTGAGGAGATTAATAGAAGTTTAGGTTTGTTACCAAATATTACAGTTGGCTACCAAATTTATGATGCATGTGGTTCCAGACTGTCTGCTATGAGTGCAACTATGGCATTGATGAATGGTCCAGAATTTACATGGAGAGACAGATGCACTGGGCAGTCTCCTATACATGCTATAATAGGAGAAACAGAGTCTTCTGCTACAGTGATTCTTTCCAGAACTACTGGACCTTTTAAAATTCCGGTGATAAGTCCCTCAGCCACGTGTGAATGTCTCAGCAATAGGAAAGAATACCCCTCTTTCTTTCGAACTATTGCTAGTGATTACCACCAGAGCAGAGCACTTGCATACATAGTCAAGCATTTTGGGTGGTCTTGGGTGGGAGCTGTGAACACGGATAATGACTATGGAAACAATGGAATGACAACCTTTCTCAATACAGCTCAGGAGGAAGGAATTTGTGTGGAGTACTCTGTAAAATTTTACCGAACAGAACCAGAAAAACTCCAAAAAGTTGTTGAGACAATAAAGAAAGGAACCGCCAAAGTCATTGTTGCATTTCTTACCAGTTCTGAAATGTACAATCTACTTGAACAACTTAGTATTCAGAACATTACAGGTCTCCAAATGATTGGAGTCGAGGGATGGATAACTGCAAAGAGTTTAATTACGCCAAACAGTTTTCATGTGCTTGGAGGGTCGCTGGGATTTGCTGTGAGAAAAACTGCTATTGAAGGATTTGCAGATTATGTCATAAAATCATTCTGGGAAACAGCTTTTCCATGCACAATGACAATTGGGAATTCTTCCCAATATTCTTTAAGTTGTGGTATATATCAGGATCTACTGTTGCTAAAGAACTACAATGAAGATGTACCTGAACAAAGATATTCAACCAATGTCTACAAAGCAGTATATGCTGTAGCTCATTCACTACACAGTCTACTCAAATGCAAAGAAGACGGCTGTAAAAAAGGTCTTGCAATACAACCACAGCAGGTTGTTGGGGCTCTGAAAAAGATTAATTTCACACTTAAACTGGGAGATATTGTGTCATTTGACAGCACTGGTGCCACAGTAGCCCAATATGAAGTTGTGAACTGGCAGAAGGATGCAAATGAATCAATTAAGTTTAAACCAATTGGGTACTATGATGCCTCACTGCCTCCTCACCAGCGATTTGTGCTTCACACTGAAAACATAATCTGGGCTGGAGGACAACTGGATAGGCCCAGGTCTGTGTGCAGCGAGAGCTGTCCTCCAGGCTCTAGGAAGGCTGCTCAGAAAGGGAGGCCTGTCTGCTGTTATGACTGTATCCCATGTGCAGAAGGAGAAATAAGTAATCAGACAGATTCAAATAACTGCAAACAATGTCCAGGGGAATATTGGTCTAATGCTGAGAAAAATAAATGTGTTTTAAAGGATGTAGAATTTCTGTCTTTCACAGAAATTATGGGTATAGTTCTAGTCATCTTCTCACTGTTTGGTGCAGTATTAACTGCGCTAATGGCCATCCTGTTTTACCGTAAGAAAGACACTCCAATAATAAAGGCCAACAACTCAGAGCTGAGCTTCCTGTTGCTCTTCTCATTGATTCTATGTTTTCTGTGTTCTCTTACTTTCATTGGTCGCCCCACTGAGTGGTCCTGTATGTTGCGTCACACTGCATTTGGAATCACTTTTGTCCTGTGTATATCCTGTGTTCTGGGGAAAACAATAGTGGTGTTAATGGCCTTCAAAGCTGCACTTCCAGGAAATAATATCATGAAGTGGTTTGGTCCTGTACAACAACGACTAAGTGTATTTGCTTTGACACTTATACAGGTTCTTATATGTGTGCTGTGGCTAACAATGTCACCCCCATTTCCCCACAAAAATTTAAAATATTATCAAGAAAAGATTGTTCTTGAGTGCAATTTGGGTTCTAATATAGGTTTCTGGGCTGTGCTTGGTTATATTGGCCTACTTGCTGTCTTGTGTTTTATTCTTGCTTTTCTGGCTCGCAAACTGCCTGATAACTTCAATGAAGCCAAATTTATAACATTCAGTATGCTCATATTCTGTGCAGTATGGATCACATTTATTCCAGCTTATGTCAGTCCTCCTGGGAAATTTACTGTAGCTGTGGAGATATTTGCCATATTAGCCTCAAGTTTTGGTTTACTATTCTGCATTTTTGTACCTAAATGTTACATAATCCTGTGTAAACCTGAAAAAAACACAAAGCAACATCTGATGGGAAAGGTTTTGATG

>OlfCg12

ATGTCAGTCTTTTTTTGCACACTTCTAATTTTCTTTCAACTTTATGCAAAGGCAGAAAAGCCTATTTGCATCATGATGGGAGACCCTAAGTATCCGCTGCTATCCAAGGAGGGGGACATTTCTATTGGAGCAGTTTTTCCAGTCCACAGCATAGAGACATTACCCTTGTTTAAGTTTACGCAAAAACCTCAGCTTTTATCATGCTCAAGTGTGAGTATAAGAGACTTTCGAATGGCTCAAATTATGGCATTTGCCATTGAGGAGATTAACAGAAATGAAAGTTTGCTCCCAAATGTTTCACTTGGTTACCAAATTTATGACACCTGTGGTTCAGGATTGTCTTTGATGGGTGCAAACATGGCATTGATGAATGTTCAAGAATTTGCATCAAGAGGTAGCTGCAATGGACAGTCTCCAGTACATGCTATCATAGGTGAAACAGAGTCTTCCAATACAGTGATTTTGTCAAGAACTACAGGACCTTTTAAAATTCCAGTGATAAGTCCCTCTGCATCATGCGAGTGTCTCAGTAATAGAAAAGAATACCCCTATTTTTTTAGAACTATTGCTAGTGATTACCACCAGAGCAGAGCACTTGCTTACATAATCAAATATTTTGGCTGGTCTTGGGTGGGAGCTGTGAACAGTGACAATGACTATGGAAACCATGGAATGGCTATATTTCTAAGTACAGCAGAGAAAGAGGGGGTTTGCGTGGAGTACTCTGTGAAATTCCAGAGAACAGAGCCAGAGAAACTGAAAAAGGTGGTAGATACAATAAAAAAAGGCACTTCAAAAGTGATTGTTGCATTTCTTACTGAATTTGAGATGAAAAATCTACTTGAATATTTAATTATTCAGAATGTAACAGGTCTACAAGTAATTGGTGTGGAGGCATGGATAACAGCAAACAGTATGATCAAACCAAATAGTTTTCATGTGCTGGGAGGGTCACTGGGGTTTGCAGTAAAAAAACTCAATATTGAAGGTTTTGAAGATTATGTCACCAAAGCATTCTGGGAAACAGCTTTTCCATGCTCACAGACTTCACAGAAAGAAAATTCTCAATATAAATTAATTTGCAACATATATCGAGATCTTCTTGTACTGAAAAATGACAATAAAGATGTACCTGAACAAAGATATGCTAGCAATGTCTACAAAGCTGTTTATGCTGTCGCTCATTCACTACACAACATATTAAAGTGTAGAGAAAATGGAGCATGTGAAACAGACATGAAAATACAACCTCAGCAGGTGGTTGAGGCTCTGCGAAAGGTAAATTTCACCATTAAGATGGGGGACTGTGTGTGGTTTGACAGCACTGGTGCAGTTGTAGCTCAATATGAAGTTGTAAACTGGCAGCCAGACTATAATGGATCGATACAATTCAAACCAGTGGGGTACTATGATGCTTCACTGCCCCCTAATCAGCGCTTTGTGATCAACACTGAAAACATAATCTGGGCTGGAGGACAGCTAAAGAAGCCGAGGTCTGTGTGCAGTGAAAACTGTCCTCCAGGCACTAGAAAGGCTGTTCAGAAAGGAAGACCTGTCTGCTGTTATGATTGTATTCCATGTGCAGAAGGAGAAATCAGTAATGAAACAGATTCAAATAACTGCAAGCAGTGTCCAGGGGAATACTGGTCTAATGCTGAGAAAAATAAATGTGTGTTAAAGGCTGTAGAGTTCCTGTCATTCAAAGAACTAATGGGTATAGTTCTAGTCTTTTTCTCACTGTTAGGCGCAGGATTAACCACACTAGTTGCTATTCTGTTTTATAGCAAGAAGGACACTCCAATAGTAAAAGCCAACAACTCAGAGCTGAGCTTCCTGCTACTCTTTTCATTGATGCTATGTTTTCTCTGTTCTCTTACTTTCATTGGTCGCCCCACTGAATGGTCCTGTATGTTGCGCCACACAGCATTTGGGATCACTTTTGTCCTCTGTATCTCCTGTGTTCTGGGGAAAACCATAGTGGTGTTAATGGCCTTTAAGGCTACTCTACCAGGTAGTGATGTTATGAAATGGTTTGGTCCTATACAACAACGACTCAGTGTTCTTGTGATTACAATTGTACAAGTTCTTATCTGTGTGCTTTGGCTAGCAGTATCTCCTCCATTGCCCTACAAAAATACGAAATATTTCAAAGAAAAAATTATTCTTGAGTGCAGTCTGGGTTCTACTATAGGTTTTTCTGCTGCACTAGGTTATATAGGTCTTCTGGCTATCCTGTGTTTCATTTTGGCTTTTCTGGCTCGCACACTGCCTGATAAATTCAATGAAGCAAAATTCATCACATTCAGTATGATCATATTCTGTGCTGTATGGATCACATTTATCCCTTCTTATTTAAGTTCTCCTGGAAAACTGACTGTAGCAGTAGAGATATTTGCCATTTTAGCTTCAAGTTTTGGTTTATTATTTTGCATATTTTTACCAAAATGTTATATAATCATGTGCAAGCCTGAACAGAATACAAAGCTGCATATTATTGGTAAGCTCAACAAA

>OlfCh1

ATGTTTGGTTTTGGATTTTTACTGCTCTGCAGCATTAAAGCCAAGGAGGTAGAGTCAAATTGCAATATGATAGGCAAACCAGAGAACCCTCTACTCTCCCAGGATGGAGATATTATCATTGGAGGGGCTTTTTCAATACACAACAAAATAAATTTAATAATACCATCATTTACAGAAAAACCTCATCATCTAATGTGCACCAGCCTGAATTTGAGAGAACTCCACTTTGCTCAAACCATGATCTTTGCAATTGAAGAAATTAACAGCAAGAGAAGCCTACTTCCAAACATTTCAATTGGATACCAAATATTTGACAGCTGTGGTTCCACATTAGCCTCTATGAGGTCATCAATGGCTTTGATAAATGGCCAGGAGCTGACAGCAGAACATACCTGCTCTGGAAAACCAGCAGTTAAAGCCATTATTGGAGAATCTGAGTCTTCCACAACTATTGTACTGTCTAGAGCAGCAGGGCCATTCAACATTCCCGTGATTAGCCATTTTGCTACTTGTGCCTGCCTGAGCAGTAGAAAGCAGTTTCCATCTTTCTTCAGAACTATTCCAAGTGATTACTACCAGAGCAGAGCACTGGCACAGTTAGTGAAACACTTCGGGTGGACCTGGATTGGAGCAGTAAGAAGTGACAATGACTATGGAAACAATGGCATGGCAACATTTGTAGAGGTAGCTGAGAAAGAAGGAGTGTGTATTGAGTATTCAGAGGCCATATCAAGGACAAATTCCAAAGACAAGATTGCCAAGGTTGTTGAAGTAATAAAAAAAGGCACTGCTAAAGTTCTCATGGCATTTCTGGCACAGGGTGAAATGGATGTGTTGTTGGAAGAGCTTATCAGGCAAAATGTCGTTGGACTACAGTGGGTTGGCAGTGAATCTTGGATTACATCAAGTTACTTGGCAACTGAAAGAACTTTAAACATCCTTGGTGGTGCAATTGGCTTTACAATTATCAAGTCAAAAATTCCCGGCCTGAAAGAATTCCTACTTAAAGTTGGTCCATCCCAGAACCTTTCAAATGCTCTTCTTGGGGAATTTTGGGAGATGGTGTTTGGTTGTTGTCTCTCTCCTACAGTCTGCCCTAATTCTGAGCATGCAACATTTTGTGATGGATCTGAAAATCTAACCAATGTTAGTAATGCGTTCACAGACGTTTCTGAGCTAAGGATTTCAAATAATGTTTATAAGGCAGTCTATGCTATCGCTTATGCACTGCATAACACAATAACTTGCAAAAACTCAAATGGTGGAAATGAAAACATAACATGTGGAGATGTGGATTTATTGGTTTCCAGCCAAGTCCTGCATTCACTTCAAAATGTCAATTTCACAATGGACTCAGGTGAGACAGTGTACTTTGACAAAAATGGGGATCCTATGGCAAAATATGAGCTAGTAAACTGGCAGAAAAATGGAGCAGGGGAAACAAAGTTCATCACTGTAGGACAATATGATGCCTCGCTATCCAGTGAACAGCAGTTTGTCATTAATTCGTTCGATATAATTTGGGCAGGAGACAGTCCCACGAAACCAATCTCAGTGTGCACTGAGAGCTGCCAGCCAGGATTCAGGCAAGCTGTGATAAAAGGAAGACCAGTATGCTGCTTTGAATGTTTGCAGTGCCCAGCTGGAGAAATTAGCAATACTACTGATTCAGCTGAATGCATCAAATGTCCATTAGAATACTGGTCAAATAAAAACCACAGCATCTGTGTTCTCAAAAAGGTGGAATTCCTTTCATTTGAGGAAAACATGGGAATTCTTTTGACTGCATTCTCATTAACTGGGGTTACTTTAACAATTGCAGTTGCGATAGTGTTTTACAAATTTATAGACACACCTCTTGTGAAGGCCAGCAATACAGAACTGAGCTTTTTGTTGCTTTTTTCATTGTCTCTGTGCTTTCTCTGTTCACTTACTTTCATTGGTCGGCCCACTGAGGGGTCCTGTATGTTGCGTCATTCATCATTTGGGGTCACTTTCGCTCTTTGTATGTCTTGTGTTCTGACAAGAACAATAGCCGTGGTAATGGCCTTCAAGACCACAGTGCCTGGTTCAGGGCTTCCTCATTGTTCATTACCTTTACAAAGAATTAGTGTTTTCTGTTGCACTGTTTTTCAGGTGATGATATGTATCCTGTGGCTGGCACTGGCCCGTCCAATGCCATATAAAAATAGCATGTATTCATTAGATAAAGTTATACTTGAATGTGATTTAGGTTCGGCTATAGGTTTCTGGGCTGTGCTGGGTTATATTGGATTGTTATCGGTGTTGTGTTTCTTTTTGGCTTTTCTGGCTAGAAAACTGCCCGATAATTTCAACGAAGCCAAATTTATTACATTCAGTATGGTCATATTCTGTGCTGTTTGGATCACATTTATACCAGCATATATCAGTTCTCCTGGAAAACTTACAGTAGCTGTTGAAATATTTGCCATTTTAGCTTCTACTTTTGGTTTGCTGTTCTGTATATTTACACCAAAATGCTACATTATCATATTCAAGCCAGAACAAAACACAAGGAAACATATCATGGGCAAAACT

>OlfCj1

ATGCTGTTGCTTGCAACCATTTTGACGACTATTGCCTGTACTCTGAGTGCTGCAGAACCAGAATGCGAAGCTTATATGACAGATGAACTCCTTTATTTCTCAAAAGAAGGTAATGTCTCTATTGGGGGTGTTTTTTCATTCCACCAAAATCCAGTTGGAGTAAATCCAACACTCAGAACTAATCCAGGAAACATCAGATGCAATGGACTTGATCCTGGGGAACTGCAATATGCAATCACAATGATTTTTGCCATTGAGGAAATAAACAATAGAACTGACCTACTTCCTGGCTTTATACTTGGCTACCGCATTTATGACTCTTGCCCAAGTATCCCTCTCTCTGTTGGAGCATCATTAACTCTGATGAATGGGCAAATGGAGACTAAAAAAAGCTGTGCAAGTCCATCTGCTGTGCAAGCTGTGATTGGAGAAACTACATCAACATCCACCATAGACATCGCAAGGACTATTGGTCCATTTAAAATTCCAGTGCTTAGCCATTCAGCAACCTGTGCATGTCTCAGTAATAGACAGCAATATCCATCTTTCTTCAGGACCATTCCAAGTGATTACTACCAGAGCAGAGCACTGGTTAAACTGGTCACCTATTTTGGCTGGAAGTGGGTTGGAGCTGTAAGAAGCATGGGAGATTATGGTAACAATGGAATGGCCACTTTTCTAGAAGCAGCTGAGAAAGAGGGTATCTGTGTCGAATACTCTGTGTCCATTTACAGAACAAATTCAAGAGAGAAGATTTTGGAGGTTACAGACATAATTAAAAAATCAACATCTAAAGTCATAGTGGCTTTTGCAGATGGCAACGATTTGGACATGCTGATTAAAGAGCTTTATTACCAAAATGTAACTGGTTATCAGTGGGTTGGAAGTGAGGGTTGGATCACGTACAGATTTCTAGCAACTGCGATTAACTATGCTGTGGTTGGGGGGGCAATAGGTTTTGCTGTGCCAAACGCTTATATTCCTGGGTTAAAGGAGTTTATTACAGGTAGCCAGCCTTCTTTGAGACCAGGCAACACAGGACTGGTTGAACTTTGGGAAAGTGTGTTTGACTGCACTTTAAATTCACAAACACACAATGCCTCCAAGATATGCAATGGGCAGGAGTCCTTAGCAAATATAAATACACGTTTTACAGATGTGTCTGATGCTAGTCTTTTAAACAATGTCTACAATGCAGTCTACGCCGTTGCTCATGCTGTTGAAGAACTGCTGACTTGTGAGAAAGGGAAGGGGCCATTCCACAAAAAAACATGTGCAGAGAAAGGGAAAATACAGCCTTGGCAGGTGTGTACTACTAAAAGTCATTCTCTGTTATTCAAAGGCTATGGGTATTCAGGTGGAAACTGTTTTATGCAGGTGCTGTATTATCTAACTCAGGTGAACTTTACGACCAAAAATGGAGAAAATGTTCACTTTGACAAACATGGTGACCCAGTTGCACGTTACACACTGGTTAATTGGCAGATGAGCTATGAAGGAATAATAACATTTGAATCCATTGGCTTGTATGACGCATCCAAGCCAGAGGGACAAGAGATTCAAATGAGAGATGATATTGAGGCAATCTGGGCAGGAAACCAGAAAAAAGTGCCTCTGTCTGTGTGCAGTGAGACTTGCCTTCCAGGCACTCGACAAGCTTTTGTGAAAGGAAAACCTATTTGCTGCTTCGACTGCATTGACTGTGCAGATGGAGAGTTCAGCAACACCACAAATGCAGTTACATGCATACCATGCCCTCTTGAGTACAAATCAAATGGGAATAGAACACAATGTGTCCTCAAAAACATTGAATTCCTGACATTTAATGAAGTAATGGGTAATATACTTGTAACATTTTCTATGTGTGGTGGATGTCTCACAATCACAGTAGGGCTGATTTTTTTCTACCACAGACACACACCAATTGTCAGAGCCAACAACTCAGAGCTGAGCTTCCTGCTGCTTTTCTCGCTCACTTTGTGTTTTCTGTGTTCTCTTACTTTCATCGGTCAGCCCACTGAATGGTCCTGTATGTTGCGTCACACAGCATTTGGGATCACTTTTGTGCTCTGCATTTCCTGTGTTCTAGGTAAAACATTAGTTGTTTTAATGGCATTCAGGGCTACACTGCCAGGAAGTAATGTCATGAAATGGTTTGGACCTCCTCAACAGAGATTAAGTGTTTTTGTCTTTACTTTTATACAATTGCTAATTTGCATGCTTTGGTTAACAATGTCACCTCCATTTCCCAACAAAACTACAAACAATTATAAAGACAAAATAATTCTTGAGTGTGATTCAGGTTCAGCTGTTGGCTTCTGGGCTGTACTCAGTTATATTGGCTTTCTGGCTATCTTATGTTTTATTCTAGCTTTTCTTGCCCGCAAGCTGCCTGATAACTTTAATGAAGCCAAATTCATCACATTCAGTATGCTCATATTCTGTGCTGTATGGATCACTTTTATTCCAGCTTATATCAGTTCCCCAGGAAAATTCACTGTAGCCGTGGAGATATTTGCTATTTTAGCATCAAGTTATGGAATGCTCTTTTGTATATTCATTCCTAAATGCTACATTATTTTACTAAAACCAGACTTGAACTCTAAGAAAAAAATCATGGGTAAAGTGTCTTCAAGGGTTTTT

>OlfCk1

ATGCGGATCTCACTTGGATGGGTAGTTTGCCTAGTAAAAGAAATGCATGTTAATCTTTTATTGATCTTGACTCTGCTCTGTATTAGAAGGCTTTTCCCTGCTGTATGTGGAGTTCATTTAGGCACCTGCATCCTTCAAGGTGACCCCCAGCCACCTTCACTTTTCAGCAAAGGAGATTTTGTTATTGGAGGGACTTTTACCATTCATTACTATCTGAGGACAGAGAAGCGCACCTACACTGTACGGCCCCAACCACTAATGTGCAGTGGCAGCATGGATTTCAGAGAGCTGCGCTTTGCTCGCGTCTTGCAGTTCGCCATCCAAGAGATCAACAACAGCTCCAATCTCTTGCCGGGAATCACATTAGGATATCATATATATGACTCATGTGCTTCTGTGCCAATGGTAATAAAAGTAGCTGTGCAGCTTGCCAACGGACTACAACTCGCATTTAACGACACTGACTCCTGTGCACAATCCTCTGAAGTTCTCGCACTAGTTGGAGAATCTGGCTCCACCGCGGCAATAACCACTTCAAGACTTTTCGGCCCTTTTGGAATTCCGCAGGTGAGTCATTACGCAACATGCGCGTGTCTCAGTGACAAGCGACAGCACCCAACTTTCTTCAGGACAATCCCTAGTGATCACCATCAAGCCGCCGCACTGGCGCGGATGGTCAAGCGCTTCGGGTGGACGTGGATAGGGGCTGTGCGCAGTGATTCAGACTACGGGAACAATGGCATGGCATCATTCTTGAAAGCCGCGGAGGTGGAGGGAATTTGTGTGGAATATTCTGAGGCCTACTACAGGACTCAAACGCGCAACAAACTTCAAAGAGTCGCTGATGTGATCCGAAGGTCAACGGCTCGTGTCATTGTTGCCTTCATGGCTGCAGGAGACATGAGATTTCTCCTAGAAGAGCTAAGCCAACAGCCTCCTCCTCCGATGCAATGGATCGGCAGCGAGGCGTGGGTTACAGACCCACAGATGCTGCGGTTTAATTTGAGTATTGGTGCTGTTGGTTTTGCAATCCCTCGGTCCGTAATTCCTGGTTTCCGTAAATTTCTTCTTGATTTGTCTTCAGAGCAGGCGCTAAAGATTCCTGTGCTGAAAGAATTTTGGGAAAGCTCATTTGGCTGTAGTCTAAAACAACACACAGGTTATTTTTCTGGCATGCCCGCATGCGATGGCACAGAGGACCTTGGCACGTTAAAGAACCCGTACACAGACACGTCCCAGTTGCGCATCTCTAACATGGTATACAAAGCCACATATGCTATAGCTCATGCACTCCATGGTATTGTCTGCAACGGAAAACTTTGTGACAAAAACATCAAAGTAGAGCCCCGAAAGGTTTCTGATCAACTTAAGCAAGTGAATTTTTCTAAAAATAATTATTCTGTTTCGTTTGATGCTAATGGAGACCCTGTGGCCGTGTATGAGCTTGTGAACTGGCAGCTTCAAGGAGATGGTTCAATTGATTTTGTGACAGTGGGCAAATATGATGCATCCCAGCCTAAAGGCCAAGAATTCAGCCTGAACAGAGCTATCATTTGGTATGATGGCACTGAAAAGGTGCCTGTGTCTGTGTGCAGTGAAAGCTGTCCACCGGGTACTCGGAAGGCTGTAAAAAAAGGAAGACCTGTTTGCTGTTATGACTGCATTAACTGTGCCGATGGGGAGATCAACAATGAAACAGATTCATTAGATTGTCACAAATGCCAACCTGACTACTGGCCCAATGCTGAGAAGATCAAATGCCTTCCCAAGCCAGTGGAGTTTCTGTCCTGGGATGAGATCCTTGGGAATACCCTAGCTGCTTTCTCTATTGCTGGCTCTTTAGTGGCTTTAAGTATGGCTTTAGTGTTTTATAAAAACAGAGTTTCTCCAATAGTGAGAGCCAACAACTCTGAGCTGAGCTTCCTGCTGCTCTTCTCACTGACTCTGTGTTTCCTCTGTTCACTCACTTTCATCGGTCAGCCCACTGAGTGGTCCTGTATGTTGCGTCACACAGCTTTTGGGATCACTTTTGTCCTCTGCATCTCTTGCGTTCTGGGGAAAACAATAGTGGTGTTAATGGCCTTCAAAGCTACACTTCCAGGAAGTAATGTCATGAAATGGTTTGGGCCTCCTCAACAGAGACTCAGCGTTTTTGGTTTCACTCTTGTACAGATTATTATTTGTGTGCTTTGGTTAACAATATCTCCACCGTTTCCTTACAAGAATATGCAGCACTACAAAGACAAGATCATTTTAGAATGCAGTTTAGGGTCAGCTGTTGGTTTCTGGGCTGTGCTGGGTTATATAGGCCTCCTAGCTTTCCTATGTTTTGTTTTAGCTTTTCTGGCCCGGAAGCTGCCTGATAACTTTAATGAGGCTAAATTCATCACCTTCAGTATGCTCATATTCTGTGCTGTATGGATCACATTTGTTCCAGCTTATGTCAGTTCTCCAGGTAAATTTACTGTGGCTGTAGAAATTTTTGCCATTTTAGCTTCGAGCTTTAGTCTGATTCTCCTTATTTTTGCTCCAAAGTGTTTCGTTATTGTATTTAGGCCAGAGGAAAACACCAAAAGACATTTATTGGGTAAAGTACCACCGAAAGCTCTC

>OlfCk2

ATGAATGCTCATCTTTTATTCACAGTGAAGATGCTGTGCATTGCCAGACTGTGTATTGCAGTTTGTGGGGTTGATTTAGGGACCTGTATCCTTCAAGGTGACGCTCAGCCACCTGCACTTTCTGAGGATGGAGACTTTATTGTTGGAGGGGCTTTTACTATTCATTACTATGTGAGGACAGAAAAGTACACCTATACTAGGAGGCCACAACCATTAGAGTGCAGTGGCAGCATGGACTTCAGAGAGCTGCGCTTTGCTCGTGCTTTGCAGTTCGCCATCCAAGAGATCAACAACAGCTCAGATCTTCTACCGGGCATCTTTTTAGGGTACCACATATATGACTCCTGTGGCTCTGTGCCAATGGCAATCAAGGTAGCGTTACAGCTTGCTAACGGACTAGATCCTAAATATAACGACACCGACTCCTGTGCAAAATCTGCCGCAGTTCTCGCACTAGTTGGAGATTCTGCTTCCACCCCGGCTATAAGCATTTCAAGAATATTCGGTCCTTTTGAAATTCCACAGGTGAGTCATTACGCAACATGCGCGTGTCTCAGTGACAAGCGACAGCACCCTACTTTCTTCAGGACCATCCCCAGTGATCACCATCAAGCCGCCGCACTGGCGCGGATGGTCAAGCGCTTCGGGTGGACGTGGATAGGGGCTGTGCGCAGTGATTCAGACTACGGGAACAATGGCATGGCATCATTCCTGAAAGCTGCGGAGAAAGAGGGAATTTGTGTGGAATATTCTGAAGCCTACTACAGGACCTACCCGCTCAGTAAACTGAAAAGGGTTGCGGATGTCATTCGCAGATCAACGGCTCGTGTTATCGTTGCTTTTGTGGCCGCAGGTGATATGAGATTTCTCCTAGAAGAACTGAGCAAAGAGCCACTCCCTCCAATGCAGTGGATTGGGAGTGAAGCTTGGGTTTCAGACCCACAGATGCTGCGGTTTAATTTAAGTATAGGAACGATGGGCGTTGCGGTCCCGCGATCTCTTATCCCGGGTTTTCGTAAATTTCTACTTGACCTGTCCCCATACAAGGTGTTAAAATTTCCCTTGCTGACAGAATTCTGGGAAAGCTCATTTAGTTGTAGTCTACAACAGCAGACAGATCCTTCCACTGGCATGCCCGCATGTGATGGCACAGAGGACCTTGGCAGGTTAAAGAACCCGTACACAGACACGTCCCAGTTGCGCGTCTCTAACATGGTGTACAAAGCAACATACGCTATAGCTCATGCACTCCATGGCATTGTCTGCAACGAAACACAGTGCAGCAAAAACATTAAAGTTAAGCCCCGACAGGTTTTTGATCAACTCAAGCAAGTGAATTTTTCTAAAAATAATTATTCTGTTTCGTTTGATGCTAATGGAGACCCTGTGGCCGTGTATGAACTTGTGAACTGGCAGCTTCAAGGAGATGGTTCAATTGATTTTGTGACAGTGGGCAAATATGATGCGTCCCAGCCTGAAGGCCAAGAATTCAGCCTGAACAAAGCTATCATTTGGTATGATGGCAGTGAAAAGGTGCCTGTGTCTGTGTGCAGTGAAAACTGTCCACCGGGTACTCGGAAGGCTGTAAAAAAAGGAAGACCTGTTTGCTGTTACGACTGCATTCCATGCGGAGAAGGAGAAATCAGCAATAAGACAGATTCTCCAGACTGTGATAAATGTCCACCTGATTACTGGCCCAGTACTGAGAAGGACAAGTGTCTTCCTAAACCAGTGGAGTTTCTGTCTTGGGATGAGATCCTTGGGATTATCCTAGCTGTTCTCTCTGTTTCTGGCTCATTACTGGCTTTAAGCATGGCTTTAGTGTTTTATAAAAACAGGGCTTCTCCAATAGTGAGAGCCAACAACTCTGAACTGAGCTTCCTGCTGCTCTTCTCACTGACTCTGTGTTTCCTCTGTTCACTCACTTTCATCGGTCAGCCCACTGAGTGGTCCTGTATGTTGCGTCACACAGCTTTTGGGATCACTTTTGTCCTCTGCATCTCTTGCGTTCTGGGGAAAACAATAGTGGTGTTAATGGCCTTCAAAGCTACACTTCCAGGAAGTAATGTCATGAAATGGTTTGGGCCTCCTCAACAGAGACTCAGCGTTTTTGGTTTCACTCTTGTACAGATTATTATTTGTGTGCTTTGGTTAACAATATCTCCACCGTTTCCGTACAAAAATATGCAGCACTACAAAGACAAGATCATTCTAGAATGCAGTTTAGGGTCAGCTGTTGGTTTCTGGGTTGTACTAGGTTATATTGGTCTTTTAGCATCACTTTGCTTTGTTTTAGCTTTTCTGGCTCGGAAGTTGCCTGATAACTTCAATGAGGCTAAATTCATCACCTTTAGTATGCTCATATTCAGTGCTGTTTGGATCACCTTTATCCCAGCTTATGTCAGTTCTCCAGGGAAATTTACTGTGGCTGTAGAGATTTTTGCCATTTTAGCTTCAAGCTTTAGTCTGATTCTTTGTATTTTCGCTCCGAAGTTTTTTATTATTTTATTTAGGCCAGAAGAAAACACCAAAAAACATTTAATGGGTAAAGTACAAAGCAAATCTTAC

>OlfCk3

ATGTATAATAATGTTTTGTTAATCGTGACACTACAGTGTATCATCACGCGTTTGTATGTTGTGTATGGGAACCGTTTAGGTAGCTGCATCCTCCAAGGTGACCCTCAGCCTCCTGTTCTCTTCGACGAAGGAGACTTTATTATTGGAGGTGCTTTTAGCATCCATTACTATTTGAGGACAGAAAAGCACACCTATACTATGCGGCCACAATCATTAGAGTGCAGTGGCAGCATGGACTTCAGAGAGCTGCGCTTTGCCCGTGTCTTGCAGTTCGCCATCCAAGAGATCAACAACAGCTCCGATCTCTTGCCGGGCATTACATTAGGGTACCGCATATATGACTCTTGTGGCTCTGTGCCAATGGCAGTCAAGTTGTCATTTCAGCTTGCCAACGGATTAGATCTCATATTTAACGACAATGATTCTTGTTCGAAATCTGCTGCGGTTGCCGCACTAGTCGGAGAATCTGGCTCCACGCCTTCTATAAGCATTTCAAGGCTTTACGGTCCTTTTGGCATTCCCCAGGTGAGTCATTACGCTACATGCGCGTGTCTCAGTGACAAGCGACAGCACCCAACTTTCTTCAGGACCATCCCCAGTGATCACCATCAAGCGGCCGCACTGGCACGGATGGTCAAGCGCTTTGGGTGGACGTGGATTGGGGCTGTGCGCAGTGATTCAGACTACGGGAATAATGGCATGGCATCATTCCTGAAAGCAGCAGAGGAGGAGGGAATTTGTGTGGAATATTCTGAGGCCTACTACAGGACCCAACCGCGCAGCAAATTGATGAGAGTCGCTGATGTCATCCGAAGATCAACGGCTCGTGTCATTGTTGCTTTCATGGCTTCAGGAGATATGAGACTTCTGTTAGAAGAGTTGAGCCAACAGCCTCTTCCTCCGATGCAATGGATCGGCAGCGAGGCGTGGTTTACAGATCCAGAAATGTTGCGCTTTAATATGTGTATTGGTGGTGTGGGTTTCGCAATCCCGCGGTCAGTTATCCCTGGCTTTCGTACGTTTCTACTTGACCTGTCTCCACAGCGCGCAATGAAATTCCCACTGCTGACAGAATTCTGGGAAAGCTCATTTAGTTGTAGTCTAAAACAGCAGACAGGTCCTTCTACTGGCATGCCCGCGTGTGATGGCACAGAGGACCTTGGCAGGTTAAAGAACCCGTATACAGACACGTCCCAGTTGCGCATCTCTAACATGGTGTACAAAGCCACATACGCTATAGCTCATGCACTCCATGGCATTATCTGTAATGAAAAGAAGTGCAACAAAAACATCAAGATTGAGCCACGAGAGGTTCTTGATAAGCTCAAACAAGTGAAGTTTTCTAAAAATAATTATTCTGTTTCGTTTGATGCTTATGGAGACCCTGTGGCCGTGTATGAGCTTGTGAACTGGCAGCTTCAAAAAGATGGTTCAATTGATTTTGTAACAGTGGGTAAATATGATTCTTCCCAGCCCAAAGGCAAAGAATTCAGCCTGAGCAGAGCTATCATTTGGTATGATGGCACTGAAGAGGTGCCTGTGTCTGTGTGCAGTGAAAGCTGTCCACCGGGTACACGGAAAGCTGTCCAAAAAGGAAGACCTGTCTGCTGTTATGACTGCATTAATTGTGCCGATGGAGAAATCAGCAATGATACAGATTGTTTGGATTGTCATGAATGTCTCTCAGACTACTGGCCCAATAATGAGAAGAACAAGTGTCTCCGCAAACCAGTGGAGTTTCTGTCTTGGGATGAGATCCTTGGAATTATTCTGGCTGCTTTTTCTGTTGCTGGCTCTTTAGTGGCTTTAAGTATGGCTTTAGTGTTCTATAAAAACAGGGCTTCTCCAATAGTGAGAGCCAACAACTCTGAGCTGAGCTTCCTGCTGCTCTTCTCACTGACTCTGTGTTTCCTCTGTTCACTCACTTTCATCGGTCAGCCCACTGAGTGGTCCTGTATGTTGCGTCACACAGCTTTTGGGATCACTTTTGTCCTCTGCATCTCTTGCGTTCTGGGGAAAACAATAGTGGTGTTAATGGCCTTCAAAGCTACACTTCCAGGAAGTAATGTCATGAAATGGTTTGGGCCTTCTCAACAGAGACTCAGCGTTTTTGGTTTCACTCTTGTACAGATTATTATTTGTGTGCTTTGGTTAACAATATCTCCACCGTTTCCTTACAAGAATATGCAGCACTACAAAGACAAGATCATTCTAGAATGCAGTTTAGGGTCAGCTGTTGGTTTCTGGGCTGTGCTGGGTTACATAGGCCTCCTAGCTTTCCTATGTTTTGTTTTAGCTTTTCTGGCCCGGAAGCTGCCTGATAACTTTAATGAGGCTAAATTCATCACCTTCAGTATGCTCATATTCTGTGCAGTATGGATCACATTTGTTCCAGCTTATGTCAGTTCTCCAGGGAAATTTACTGTGGCTGTAGAGATTTTTGCCATTTTAGCTTCAAGCTTTGGTTTGATTCTCTGTATTTTTGCTCCTAAGTTTTTCATTATTATATTTCGACCAGAACGGAACACCAAAAAACACATAATGGGTAAAGTACCATCAAAAGCCCTT

>OlfCm1

ATGGAGCCGCTTTTTGCGCTCATACATGTAGTTATGAACATCATTTCTTTTGCCAAGGCCAATGAAACTGCCTGTACTCTGCAAGGACAGCCTGTGTACCCTCAGTTATGGAAAGATGGAGACATCATAATTGGAGGTGTTTTCTCCTTCCATAGCAGCTGGGAGATCAGACAACTTACATATTCTGTTACACCACCACCGCTGAAGTGCATCAGCCTAAATTATAGAGACTTCCAGTATGTGCAGTCCATGCTTTTTGCTGTGGAGGAAATCAATAACAGTTCCACTCTGATACCTGGGGTCTCATTAGGCTACAGGATATATGATACATGTGGTTCAATGGCAATGGCTGTCAGAGTGACTATGGCACTTGCTAATGCACATGAGAACACAACGTCCGATGGACCCTGCACAAAGCAGGCATATGTCCAAACCATTCTAGGAGACACAACCTCATCGGCATGCATGGCGATGGCAAAAACTATTGGACCATTCAACCTTCCCATGATCAGTCACTATGCCACCTGTGAATGTCTCAGTGACAAAGTCAAATACCCCTCATTTCTTCGAACTATTACGAGCGATTACTACCAGAGCAGAGCACTGGCAGAACTGGTTAGACACTTTGGCTGGACCTGGGTGGGAGCCATAAGAACAGATGATGATTATGGTAATAATGGGATGGCCACTTTCACCAAAGTGGCTGAACAGATGGGAATTTGTTTGGAATATTCTGTGCCATTTTTCAGAACATATGCTGAAGATAAAGTAATAAGGATAATAGAACAGATTAAAAGCTCAACTTCACGAGTGATTGTGGCATTTCTTGTGCACTGGGACTTGGAGGTTCTGTTGCATAAGTTTGTTGAATATAATATTACTGGATACCAGTGGGTAGGTACAGAGGGATGGATCTCTGATTCAGTCATTGCCACTATGGATACGCATCACATTTTACAAGGGGCTGTTGGTCTAGCAATACCCAAAACAGAAGTGACAGGTCTAAAGGAATATATTTTAAACATCAAACAACTAAAATCCTCTGGAAGTACCATATTTTCAGAGCTCTGGGAATCACTGTATCAGTGTAAATACCCCAACAAGGATGATTCAGTGTCTATGAATGCATGCACAGGCAATGAAGAACTGTCCCAGATGCAAAACAGTTTTACTGACATGTCACTTATGCCTATATTCAGCAATGTGTACAAAGGAGTTTATGCTGTTGCTCACACTCTTCATGAACTTTTGGGCTGCAAGGAAAAATGTGCCTCAAAGAAACAGCTTGATCCTATAACTTTTCTGAAGCACCTGCGAAAGGTACGTTTCAAAACCAAAGATGGTGAGGATGTATACTTTGATGAGAATGGTGAGGCAGTTGCACGATATGAGGTAATAAATTGGCACCCGAGTAATGGAAAACACGATCAGTTTGTCACTGTTGGGCTTTATGATGCTTCTCTTCCTGTGAAAGATCGTTTGGCAGTGAATGTGGCTTCAATTGTGTGGTCAAATAATGCAACAAAGGTCCCAGTATCTGTGTGCAGTGAGAGCTGCTCACCTGGCACTAGAAAAGCAGTTAAAAAGGGAAAGTTTATATGTTGTTACGACTGCATATCATGTGCAGATGGAGAGATCAGTAACACTACAGATTCAGTAACATGTCTGCGGTGCCATCAAGAATTGTGGTCAAATCTGCAAAAGGATGTATGTGTTGAGAAGGAGACTGAATTTCTGTCTTTTGAAGAGATCATGGGAATCTTGCTTACAACGATTTCCATTGTTGGTGCATTTGTAACAATGATAATTGCAGTCATATTTTTCCGGTATAAAAACACACCAATAGTGAAAGCCAACAATTCAGAGTTGAGCTTTTTGCTTCTGTTTTCACTTATGTGTTGTTTTCTCTGTTCACTCACTTTCATTGGTCAACCTACTGAGTGGTCCTGTATGTTGCGACACACAGCGTTTGGGATCACTTTTGTCCTCTGTATCTCATGTGTTCTGGGGAAAACAATAGTGGTGTTAATGGCTTTCAAAGCTACACTTCCAGGAAGTAATGTCATGAAATGGTTTGGTCCTCCTCAACAGAGGCTCAGTGTTCTTGGATTCACACTCATACAAGTGCTTATTTGTGTGCTTTGGTTAACAATTTCTCCACCATTTCCCTTTAAAAATTTTAATTACTTTAAGGAAAAGATCATTTTAGAATGTCATGTAGGTTCATCAATAGGGTTCTGGGCTGTTCTGGGTTACATAGGATGTCTGGCTCTTTTGTGTTTTATTTTGGCTTTTCTGGCACGTAAATTGCCTGACAAGTTTAATGAAGCCAAATTCATTACATTCAGTATGCTCATATTCTGTGCTGTATGGATTGGTTTTATTCCAGCTTATGTAAGCTCGCCCGGGAAGTTTACTGTGGCCGTAGAGATATTTGCTATTTTAGCTTCAAGTTTTGGTTTACTATTCTGCATTTTTCTCCCAAAATGCTATGTAATTTTATTTAAACCAGACAAGAATTCCAAAAAGCACATCATGGGTAAATTAACGTCAAAC

>OlfCm2

ATGGAGCCACTAGTTGCACTGCTGCATATGGTGATAGCCATCATGACTTTTTCTAAAGCTAATGAAACTACCTGTACACTACAAGGAGAGCCAGTACTCCCTGAATTATGGAAAGATGGGGACATCATAGTTGGAGGTGTTTTCTCTTTCCATAGCAGCTGGGAAGTCAGGCAGCTGACATACACCTTTGTGCCACCTCCACTAAAATGCATCAGTCTTAATTTCAGAGACTTCCAGTATGCACAATCAATGCTTTTTGCCATAGAGGAAATTAATAACAGTTCCACTTTGCTACCCGGGGTCTCATTAGGCTACAAGATCTATGACACATGTGGCTCAGTGGCAGTTGGGGTTCGAGCCGCTATGGCCCTTGCAAATGGGCATGAGAAAATATCTGTTGAAGGGCCCTGCACAAAGCATGCTGAAGTTCAAGCCATTTTAGGGGACACAACTTCATCAGCTTGCATGGCAATAACAAAGAGTATCGGGCCATTCAAGCTTCCATTGATCAGTCACTATGCCACCTGTGAGTGTCTTAGTGACAAGGTGAAGTATCCTTCATTTCTTCGAACTATTGCAAGTGACCACTACCAGAGCAGAGCACTGGCAGAACTGGTTAGACACTTTGGCTGGACCTGGGTTGGAGCCTTAAGAACAGATGATGATTATGGCAACAGTGGGATGGCAACTTTTACTAAGGTGGCTGAACAGATGGGAATCTGTTTGGAATATTCTCTCCCATTTTTCAGAACATATACTGAAGATAAAGTAATGAGGATAATTGAACAGATTAAAAGCTCTACTTCACGAGTTATTGTAGGATTTCTTGCTCACTGGGACTTGGAGGTGCTGCTGCATAAATTTGTTGAATATAATATTACTGGATACCAGTGGGTAGGCACTGAAGCCTGGATCTCTGATTCAGTCATTGCCAGCATGGATACACATCACATTTTACAAGGAGCTGTTGGTCTGGCTATACCCAAAACAAAAGTGACAGGTCTACAAGAATTCATTTTAAATATAACACCACTAAAGTCTTCTGGTGGTGCAATTTTTTCTGAGTTCTGGGAAGCCCTGTTTCAGTGTAAATATTCCAACAAGGATACTTCAGTTTCCATAAATGCATGTACAGGCAAGGAAGAGTTGTCACAAGTAGAAAACCTATTCACAGATATGTCGCTCATGCCTATTTTCAGTAATGTGTACAAAGGAGTGTATGCTGTTGCTCATGCACTTCATGAACTTCTAGGCTGCAAAGACAAATGTGCCTTAAAGAAACAGCCTGATCCTGTAACTTTTCTGAAGCACATTAGAAAGGTACATTTCAAAACCAAAGATGGTGAGGAGGTTTACTTTGATGAGAATGGCGATCCAGTGGCAAAATATGATATCATTAACTGGCAGCAAAGTACAAAACAACATTACGAATTTGTGACTGTTGGGTTTTATGATGCTTCTTTTATGGGGATGGATCGTTTAGCTGTCAATATGTCATCCATTTTCTGGGCAATTAATTCAACAAAGGTTCCAGTGTCTGTGTGCAGTGAGAGCTGCCCCCATGGTACTAGAAAAGCTGTTAAGAAAGGAAAACCTATATGCTGTTATGACTGCATTTCGTGCACAGAGGGAGAGATCAGTAATACAACAGATTCAGTAACATGTCTTCGGTGTCATCAAGACTTTTGGTCAAATCTACAAAAGGATCAGTGTATTAGGAAGCAAACTGAATTTCTGTCTTATGAGGAGATCATGGGGCTTTTGCTCACAACTATTTCAGTGGCTGGTGCATTTTTAACAATGCTAATTGCAGTCATATTTTTCAAGTACAAAAATACACCAATAGTGAAAGCCAACAATTCAGAACTGAGCTTCTTGCTTCTCTTTTCACTGATGCTGTGTTTTCTCTGTTCCCTCACTTTCATTGGTCGCCCCACTGAGTGGTCTTGTATGTTGAGACACACAGCATTTGGAATCACTTTTGTCCTCTGTATTTCCTGTGTTCTGGGGAAAACAATAGTGGTGTTAATGGCCTTCAAAGCTACACTTCCAGGAAGTAATGTCATGAAATGGTTTGGTCCTCCTCAACAGAGACTCAGTGTTCTTGCCTTTACTCTTATTCAAGTCCTTATTTGTGTGCTTTGGTTAACAATATCACCCCCATTTCCTTTTAAAAATGTGAAATACTTTAAAGAAAAGATCATTCTAGAGTGTAATGTTGGATCAGTATTAGGTTTTTGGGCTGTGTTAGGTTATGTTGGGCTACTTGCTATTCTCTGTTTCTTTTTGGCTTTCCTGGCACGAAAACTCCCTGATAACTTTAATGAAGCCAAATTCATCACATTTAGTATGCTCATATTCTGTGCAGTTTGGGTTGCTTTCATACCAGCTTATGTTAGTTCGCCTGGAAAATTCACTGTGGCAGTGGAGATTTTTGCTATTTTAGCCTCCACTTATGGACTTTTGTTCTGCATATTTATTCCTAAATGCTATGTTATTTTATTAAAACCAGACAAGAATTCTAAAAAACATTTGATAGGTAAAGGACCCCCAAGAGTCTTA

>OlfCn1

ATGAAAAACTTTAACATTTTGTTATTTTGCTCTCTGCTCTATGTACAAACAAGCTCAATGTGCCAGCTGCTTAGAACATTTGAAATGCCTAACATTTTTAAAGTAGGGGACATTATGATTGGGGGAATTTTCCCCATTTTCAATAAACAAGAAAATATAATTGGCTCTTTTGAGAGAAACATGCAAAGGATTAAGTGCACGGGATTTGACCTACGTGCATTTCGTTGGATTCAGTCAATGATGTTTGCAGTGGATGAAATAAATAAAAATGAGGAGTTGCTTCCTCACATTTCTTTGGGCTATAAAATAATGGATTCTTGTGCTTCACCCACGAACGTTTTACGAGCAGTATTCTCACTGGTAAGTGAGCAGAAGGAGCAGGAATTTACATCTCAGTGTCACCTTCCACTTACAGCTCTTGTTGCTGAATCAGGATCTTCACAGTCTTTAGCTGTTGCCGGAATGCTTGGACCATTTAGAGTGCCCATGGTAAGTTATTTTTCAACATGTGCGTGTCTCAGTGATAGAAAAAAATATCCATCATTTTTCCGCACAATTCCAAGTGACTTCTACCAAGCAAAAGCTTTGGCCTCTCTGGTAAAACAGTTTGGATGGACTTGGATTGGAGCTTTGCAGTCTGACAATGATTATGGGAGAAATGGAATTTCAGCTTTCACAAAGGAAGTGGAAAAAATGGGAGTTTGCATTGCATTTGTCGGCACTATATTAAGAACATATCCCCAAAGTAAAATCACTGAAGTGGTAGAAATGATCAAAGAATCGACAGTAAAAGTCATCCTGGCATTCGTGCCAGAGGGAGATCTCTATCCTCTAATGAAAGAAGTTGTGAACCGGAATATCACAGGAATCCAGTGGATTGCAAGTGAAGCCTGGGTAACAGCAGCAAGACCATCTACCCCTGAAATGTTCAAGTCATTTGGAGGCACCGTAGGATTTGTGGTCAGAAAGATGGCTATGTTAAAACTAAGACCTTACCTGGAAAACATCAGTCCCTACTCTCCTACACAATCAGCTTTCGTCAGTGACTTCTGGGAAACAGTTGTTGGCTGTAAACCATGTCTGAACTGTGAGCCATCTGCAAATAGCACACTCAATGGCCAAATGTGCACTGGACAGGAAAAACTAACGTTTACAGACAAATTTTTCGATGTAACACAAGTAAGAGTGACATATAATGTGTATCAAGCTGTCTATGCAATTGCACATGCAATTCACAAAGTGCTTTATTGCCAAGGAGATAACAATCTTTCAAAAATGTGCTTAAATGTATCACAGATAACACCTAAACAGGTCAGCGATCAGTTGGAAAGAGTGAATTTTATAGATGAATATGGAGAAAATGTGTTCTTTGATGAGAATGGAGACCCGCCTGCATCATATGAGCTCATAAACTGGCAGCTAAATCAGGGAGAGGTGCAACATGTGACTGTGGGCTATTTCAGCAAATCCCCAGATGGAACATATAAACTTACAATTAAAGAGGATAACGTCCACTGGAGCACAGAAAATTTGATACCCAAAGCAGTGTGCTCAGACACCTGCCCGAAAGGCACAAGGAAAGCACAAATAAAAGGTCGGCCTGTTTGCTGCTTTGACTGCATCCCATGTGCTGATGGTTCAATATCAAATACAACCGGAGCAGCAGACTGCACTCTTTGTCCTAAAGAATACTGGTCAAATGAAAGACGGGACAAGTGTCTTGTGAAAACAATAGAGTTTCTGTCGTATACAGAAACAATGGGAATAATTCTCACTGCTCTATCGCTATTTGGAGCCAGTCTTACAGTTGCAACTATGATTGTCTTCATACACTATAGAGAAACACCAATAGTGAAAGCTAACAACTCAGAGCTGAGCTCACTATTACTCTCTTCGTTGTTCTTTTGTTTCCTTTGTCCTCTTACATTCATTGGTGAGCCGACACATTGGTCATGTATGCTACGTCATACAGCTTTTGGGCTTACTTTTGCTCTCTGCATTTCTTGTGTTTTGGGGAAGACCATAGTAGTTGTCACAGCTTTCAGAGCCACATTACCAGGAAATAAATTGTCTGGTAAGTTTGGGCCAGTACAACAAAGAGCCATCGTGTTCTTATGCACTGCGATTCAAATAGTGATCTGTGCTTTATGGCTTCTAATAAAGCCACCATTTCCTGACAAGGCCCTCAGGTATGACCATAAAAAGATCATTTTAGAGTGCAACACTGGCTCGGATGCTGGATTTTATGCAGCTTTAGGATATGTTGGCCTTCTGTCAACAATCTGTTTGATTTTAGCATTTTTAGGAAGAAAACTTCCTGATAATTTTAATGAGGCCAAATTCATCTCATTCAGTATGCTCATATTTTGTGCTGTTTGGGTAACATTTATACCTGCATACATTAGTTCACCTGGCAAGTACACTGTTGCGGTGGAGATTTTTGCCATTTTGTCATCTGCCTTTGGATTGTTGCTATGTATATTTGCACCAAAATGTTTCATTATTCTCATTAAACCAGAGAGAAATACTAGAAAGCATGTCATGGGGAAATCAAAGACTAGTCTC

>OlfCq1

ATGGACACGTGGATCACTTTATATTTATGTTTTTATCTGTTTTTTAAATCTATCTCTGCCTCTTCAATCAGTAAATCAGGCTCTTGTCAGCTCCAAGGACACTTCAAGTTAAATGGGATGTACCAAGATGGAGACCTTATGATTGGGGGCCTGTTTGCATTTCATCTTATCACAGTTTTCCCGGAACTAAACTTTAAAAGAGAACCGGAACAGACACATTGTGAGCGGTTCTATATGGCAAGTTTTCAGCAGGCACAAACTATGGTATATGCCATAAATGAAATCAATAACAATCCTAACCTGTTGCCTAACATCACCCTCGGTTACCATCTGTATGACAATTGTGTGAAGCTTGGAGTTGCATTCAGGTCTGCAACAGCTTTGGTTAGTGGGACAGAGGAATCCTTCAATGTTTTGAACTGCACTGGTCCACCACCCATCATTGGGATTGTGGGGGATCCTGGATCTACTCATTGTATAGCAATCTCCAGTGTGCTGGGATTATTTCGAATACCTATGGTTAGCTACTATGCCACATGCTCCTGTTTAAGTGACCGGAGCAAATACCCCTCTTTCTTCAGAACAATTCCCAGTGATGCTTTCCAGGTGCGGGCTATGGTTCTGATCTTGAAGCACTTTGGATGGACCTGGGTTGGGCTCCTCTACAGTGATGATGATTATGGCATCAATGCTGCTCAATCTTTCCAAAAGGAAGTGCAGTTGTTTGGAGGTTGTGTTTCTTTTTCTGAAATCCTGCCACTTGATAATAACCACATGGACATCCAGCGTATTGTACAAGTGATTCAGGCCTCTACAGCAATAGTGGTGGTAGTTTTTTCCACAGAAGCCTATCTGCTTTCCTTGATGGACGAGGTGGTTTTGCAGAATGTAACAGGCAGGCAGTGGATTGCAAGTGAAGCTTGGGCCACCTCTTCAGTTTTTCACACTAAGCGTCTTTTGCCTTTCCTGGGAGGCACACTGGGTATTGCCATAAGACGTGGAGAGATCCAGGGACTTCGTGATTTTCTGCTAAGCCTCCACCCAGACAGTAATTTGAGAAATAATATGGTGAAAATCTTCTGGGAAAACATGTTTGAGTGCAGTTTTGATACTGTGGGTAGAAAGGGTGAAACGATGTGTACAGGTCAAGAAGATATCAGGACAACAAACACACCATATTCTGACGTTTCAGAACTGAGGGCTTCTTATAATGTCTATAAGGCAGTTTATGCCCTGGCACATGCTCTTCATGACCTAATACAGTGTGAGGAGGGGAAAGGACCATTCAGATGTGATGGCATAACAAACTTGAAACCCTGGCAGCTGGTTCACTACCTACAGAAAGTGAACTTCACCACAGGCTTTGGGGATCATGTGTCATTTGATGAGAATGGAGATGCTCTGGCCATCTATGATGTGATGAACTGGCACCCCAGCTCTGATGGGTCAATTGTTGTCCGCACAGTAGGTGTTGTAGATGAAGGGGCATCAACAGGGAAGGTGCTTACATTGGAGGAAGATGAAATATACTGGAACACAGCAAAAAATAAACCTGCACGGTCTGTGTGTAGTGAGAGCTGCCCCACAGGAACCAGACGAACAAGGAGGAAGGGGCTTCCTGTCTGCTGTTTCGACTGCCTTCCATGTGCAGACGGAGCAATTTCTACAATACCAGATTCCATTGAATGTGTGGTGTGTCCAGATGAGTTCTGGTCTAGCCCAAAAAAGAATCAGTGTGTCCCCAAAGATGTAGAGTTTCTTACTTATGGGGATCCTCTGGGCATCTCTCTGACCACCGCTTCCCTGCTTGGCTCCTGCATCTGTTCTGCTGTGGTTGTCATTTTTGCACATCATCGTCACACTCCTGTAGTACGTGCTAACAATTCAGAGCTCAGCTTCCTTCTGCTTGTGTCGCTCAAACTGTGTTTCCTGTGCGTGCTGCTGTTCATTGGCCAGCCACAGTTGTGGACATGTCGGTTAAGACATGCTGTGTTTGGCATTAGCTTTGTCCTGTGCATCTCCAGCATTCTGGTCAAGACTATGGTGGTGATAGCAGTATTCAAGTCCTCTCGACCTGAGGGTAAAAGTGCTATGAAATGGTTTGGTTCACATCAACAAAGATGCACAGTTCTGGTCCTCACTGCACTCCAGGTTGTCATATGTGCAGTCTGGCTAACAAATGCATCCCCAAAACCTTACAAAAACAACCAGTATACAAGCTCCAAAATAGTATATGAATGTACTATTGGCTCAGTGGTTGGTTTTGCAATGCTACTTGGCTATATTGGCATTTTAGCAGCAGTAAGTTTTCTCTTAGCCTTCCTGGCACGAAATTTACCAGATCATTTTAATGAGGCAAAGTTCATCACTTTTAGCATGCTCATCTTCTGTGCTGTATGGATTGCATTTGTTCCAGCATATGTGAGCTCTCCAGGAAAATATGCAGTGGCTGTGGAGATATTTGCTATTTTAGCTTCTAGTTTTGGATTGCTGGCTGCCATATTTGCACCAAAATGCTACATTATTATTTTACACCCAGAAAGAAACACTAAAAAAGCCATCATGGGAAGAGCAACC

>OlfCq2

ATGCTGTGTGAGATGTGGATAACTTTAAACATCTGTCTTTATATGTATTTTAATCATATCTCTGGTGTTTTTACCTCTGAGTCTTGTCAGCTCCAGGGATGCTTCAAGTTGAATGGGATGTACCAGGATGGTGAATTTATTATTGGAGGCCTCTTTGAGGTCCAGAACCTCAAAGTTTTCCCAGAGCTTAGCTTCACAAATGAGCCTGAACTGCCCCAGTGTGAGGAATTCTATATGGCAAGTTTTCAGCAAGCACTGACTATGGTTTTTGCTATAAATGAGATTAATAGTAATAGCAAATTGTTGCCTAACATCACACTTGGTTACCAGATTTATGACAACTGTTTAAGGCTTGGAGTGGCATTCCGGGCTGCTATGTCCCTGGTTAGTGGGACAGAGGAATCCTCAAACCTCAGCTGCACTGGCCCTCCGCCGGTGATTGGCATTGTAGGGGATCCTGGTTCAACTCAATCTATAACAATTTCTAGTGTCCTGGGGCTATATCGAGTTCCTATGGTTAGCTACTATGCGACCTGCTCATGTTTGAGTGACAGGAAAAAGTACCCCTCTTTCTTCAGAACAATCCCCAGTGATGCCTTCCAGGTGCGGGCTATGGTTCAGATCTTAAGACATTTTGGATGGACCTGGGTTGGTCTCCTCTACAGTAATGATGACTATGGCATCTACGCTGCTCAGTCCTTCCAGCAAGAAATGCAGATTTTGGGAGGTTGTGTGGCTTTTTCTGAAATGTTGCCTTATGATAACAACGGTAGAGACATTCAACGAATAGCAGGAGTTATTCAAAAGTCTACAGCCAAAGTAGTTGTAGCATTCTCAACTGATCTGGCATCCTTGATGGATGAGTTGTTACTGCAAAATGTGACAGGCAAGCAGTGGATTGCAAGTGAGGCTTGGACTACAACACCTGTCCTGCAAACTCCACAATATTTACCGCTACTGGGGGGCACACTGGGAATTGCCATCCGGCGTGGACAGATTCAGGGACTTTATGAATTTCTAAAAAATCTTCGACCTGACAAAAATCCAAAAAACAGCACCATTAGAATCTTTTGGGAGACCATGTTTGGGTGCAAGTTTGAGGTTGGAGGTAAAAAAGTAGATGAACAGCAAGAAAGGGGGAGAAACAAATGTTCAGGCCAAGAGGATCTTAACAACTCAGAAACAGCATACACGGATGTGTCAGAGCTGAGAGCCTCTTATAATGTTTATAAAGCTGTTTATGCCTTGGCACATGCACTTCATGACCTAGTAAAGTGTGAGGAAGGAAAAGGGCCATTCACTGGGAACAGCTGTGCTGACATATCCAATCTAAAACCATGGCAGCTGGTTCACTACATACAGAATGTAAAATTCACCACAGGCTTTGGGGATCATGTGTCATTTGATGAGAATGGAGATGCTCTGGCCATCTATGATGTAATGAATTGGCATCCAAGGTCTGATGGGTCGATCATTGTCCATGTGGTCGGTGTTTATGAAGAGGCAATATTAGGGAAAGTCTTAACACTGGATGAGGATGCATTATTCTGGAACTTTGAAACAAAAAAGAATATTCAAAATAAAATGAATAATGCTCCCCCAGACTCTGTGTGTAGTGAGAGCTGCCCCCCAGGAACCAGGCGAGCCAGGAAGAAGGGTCTCCCTGTCTGCTGTTTTGACTGCCTGCCATGTGGGGATGGCGAGATTTCTAATACTACAGATTCTCCTGAATGTTTCATATGCCCAGAAGATTTTTGGTCAAGTCCAGAAAATGACCGTTGTGTTCCCAAAGAAATGGAATTTCTTTCCTATGATGATCCGCTTGGAATATCGCTGACCACTGCCTCTCTTTTAGGAACCTGCTTCTGTGTTCTTGTGTTGGTTATCTTTGCTTATCACCATAACACTCCAGTAGTACGAGCCAACAACTCAGAGCTCAGCTTCCTTCTACTGGTGTCACTCAAATTTTGCTTTCTGTGTGTGCTGCTGTTCATTGGCCGGCCACGGTTGTGGACGTGCCAGTTAAGACATGCTGTGTTTGGCATAAGCTTTGTCCTGTGCATCTCAAGCATTCTAGTTAAGACTATGGTAGTAATAGCTGTATTCAAGACCTCGCGGCCAGAGGGAAAAACTTCCATAAAGTGGTTTGGTTCAGCACAGCAAAGAGGGACAGTTATAGCCCTAACTTCTGCACAGGTAGCAATATGTACTGTCTGGTTGTCCTCTGCATCTCCAACACCTCATAAAAACAACCAGTACATTCATTCTAAAATAGTATATGAATGTGCCATTGGCTCAGTTGCTGGTTTTTCTTTGCTCCTTGGATACATTGGACTGTTGGCAGCAGTAAGCTTCTTGTTAGCCTTCATGGCAAGGAAACTTCCAGATAATTTCAATGAAGCAAAGTTTATCACATTTAGCATGCTGATCTTCTGTGCTGTATGGATTGCATTTGTTCCAGCATATGCGAGTTCACCAGGAAAATATGCAGTGGCTGTGGAGATATTTGCTATTTTAGCTTCCAGTTTTGGATTACTGGCTGCCATATTTGCCCCAAAGTGCTACATCATTATATTACACCCAGAAAGAAACACTAAAAAAGCCATCATGGGACGATCGACTCAAAATAAA

>OlfCq3

ATGTGTATCACTCTAAACATCTGCCTGCATCTGTCCTTTATCTTTATTTCTGTCAATTCTGACTCCTGTCAAATCCAGGGACATTTCAAGTTGAACAGAATGTATAAGGATGGAGATTATATCATTGGAGGCTTGTTTGAGGTTCAGCACCTCAAAGTATTTCCAGAACTGAGTTTCCGAATGGAGCCAGAACAGCCAAAGTGTGAGGAATTCTACATGTCAAGCTTCCAGCAGGCACAGACCATGGTTTTTGCTATAGATGAGATCAATAAGAATCCAAACCTGTTGCCTAACATCACACTTGGTTACCATCTTTATGACAACTGTTTAAAGCTTGTGGTGGCATTTCGGGCTGCTACAACTCTTATTAGTGGGACGGAGGAAACGTTTTCAAACTTCAACTGCACTGGCCCACCCCCAGTGATTGCTATCGTTGGGGATCCTGGGTCCACTCATTCCATCGCAGTTTCTAGTGTACTAGGCCTGTTTCATGTCCCATTGATTAGCTACTATGCCACATGCTCATGTTTGAGTAACAGGAAAAAGTACCCCTCTTTCTTCAGAACAATCCCCAGTGATGCATTCCAGGTTCGGGCTATGGTTCAGATCTTAAAATATTTTGGATGGACCTGGGTTGGTGTTCTCTATAGTGATGATGACTATGGCATTTATGCTGCTCAGTCCTTCCAGCAGGAAATGCAGCGGTTCAAAGGCTGTGTGGCTTTTTCTGAAATAGTGCCCTATGATAATCATAGGGACATTCAACGCATAGTGGCAGTTATTAAGGCCTCTACAGCCAGAGTAGTCGTGGCTTTCTCAACTGATCTGTTACCCCTGATGGAAGAGCTGTTACAACAAAATGTGACAGGCAGGCAGTGGATTGCAAGTGAGGCATGGTCCACCTCCCCTGTCCTTCATCTTCCACGTTTTGTACCTCTCGTTAGGGGCACGCTTGGCATTGCTATCCATCGGGGAAAGATCAAAGGACTTCATGAATTTCTGCTACACATACAACCTGACAATGATCCAACAAATAACATGGTGAGAATTTTTTGGGAGAACATGTTTGGGTGCAGTTTTGAGAAAGGCAACGGGTATGGAGAAAAAATGTGTACAGCACAAGAGGATCTAAACATCACAGTTAATGAATACAATGATGTATCAGAGCTGAGAGCCTCTTATAATGTCTATAAGGCAGTTTATGCACTGGCACATGCACTTCATGATCTTATGCAGTGTGAGGAGGGGAGAGGACCATTCAGTGGGAACAGCTGTGCTGACATAACAAAACTGAAACCTTGGCAGATGGTTCCCTACCTGCAAAAAGTGAACTTCACTACAGGCTTTGGGGATCATGTGTCATTTGATGAGAATGGAGATGCTCTGGCCATTTATGATGTGATGAACTGGCACCCCAGCTCTGATGGGTTAATTGTTGTCCGCACAGTAGGTGTCGTAGATGAAGGGGCATCAGCAGGGAGGGTCCTCACTCTGGATGAGGATGCAATATATTGGAACTTTGAGACAAAAAAACCACCACGGTCTGTATGCAGTGAGAGCTGCCCTCCAGGAACCAGACGTGCCATGAGGAAGGGCCTTCCTGTCTGCTGTTTTGACTGCTTGCCATGTGCGGACGGGGAGATTTCTAATATGTCAGATGCTACTGAATGCACATCATGTCCAAATGAATTCTGGTCTAGTCCAAAAAAGGATCAATGTGTCCCAAAAGAAGTAGAATTTCTATCCTATGAGGATCCCCTGGGCATCTCGTTGACCACTGCTTCACTGCTTGGCACCTGCTTTTGTGCTCTTGTGTTGGCCGTCTTTGCTCATCACCATAACACTCCTGTAGTACGTGCCAACAATTCAGAGCTCAGCTTTCTGCTGCTTTTGTCACTCAAACTGTGCTTTCTCTGTGTGCTGCTGTTTATTGGTAGGCCACAGTTATGGACATGTCAGTTAAGACATGCCATGTTTGGCATAAGTTTTGTCTTGTGTGTCTCTAGCATCCTTGTCAAGACCATGGTGGTAATAGCTGTGTTCAAGTCCTCTCGACCTGAGGGTAAAAGTGCTGTGAAATGGTTTGGAGCAGTTCAGCAAAGAGGTACCGTCTTGGTTCTCACTGCTGTCCAGATTGTTATATGTGTGGTCTGGCTCTCAACTGCCTCTCCAACACCCCATAAAAACATCCTGTATGTCAGATCTAAAATAGTATTTGAATGCTCTATTGGCTCAATGGCTGGCTTTGCCATTCTGTTGGGTTACATTGGCATCCTGGCAGCTGTAAGCTTCCTGTTAGCTTTTCTAGCAAGGAACCTCCCAGATAACTTTAATGAAGCCAAATTCATCACATTTAGTATGCTGATATTCAGTGCTGTGTGGATTGCATTTATTCCTGCATATGTGAGCTCTCCAGGTAAATATTCAGTCGCAGTGGAGATATTTGCCATTTTGGCTTCTAGTTTTGGACTACTGGTTGCCATATTTGTCCCAAAGTGCTACATCATAATCTTACATCCAGAGAGAAATACTAAAAAAGCCATCATGGGAAGAACAGCTGAAAAA

>OlfCq4

ATGTGGGCAGTACTACTTTTTTGTTTGGTTCTGTCTTGTTACTATATCTGTGTAACTTTGATGGCCAGTACAGGTATCTGTCAGCAACAGGGACACTTTACAATAAATGGGATGCACCAGGATGGAGACTTTGTCATTGGTGGTTTGTTTGATGTTCAGACATACCTAAAAGTATACCCTGAGATAAGCTTCAGAACGCAGCCAAAACTACCAAACTGTGAACTCTTCTATATGGAAAGCTTCCAGCAAGCACTGACAATGGTTTTTGCCATCAGTGAGATTAATCACAATCCCAACTTGCTGCCAAACATCACACTTGGTTACCAGATCTATGACACTTGTTTAAGGCTTAGAGTGGCATTTCAGGCAGCAACAGCTCTGATAAGTGGGACAGAGGAGACCATCTCTGACTTCAACTGTAAAGGCCCACCACCAGTTATTGGACTCATAGGTGATCCAGGATCTACACATTCTATTGCAATTTCCAGTGTTCTGGGGCTGTTTCGAATGCCTATGATAAGCTACTACGCCACTTGCTCTTGTTTAAGTGACAAGAAAAAGTACCCTTCTTTCTTCAGAACAATTCCAAGTGATACCTTCCAAGTGCGGGCTATAGTTCAGACCTTGAGACATTTTGGCTGGACCTGGGTTGGTCTGATCTACAGTAATAATGACTATGGTATCTACGCTGCTCAGTCCTTCCATCAAGAAATGCAGTTGTTTGGACACTGTGTTGCTTTTTCTGAAATCCTGCCCCAAGATAACAACCCCAGAGTTATTGATCACATTATGGGAGTAATTCAGGCCTCTACAGCTAGAGTAGTGGTTGTTTTTTCTGCTTCATCCTTATTGATTCCTTTGATGAACAAGGTAGTGTTGCACAACTTAACAAGCAGGCAGTGGATAGCAAGTGAAGCCTGGGTCACCGCAGCTGTGTTCCGCACACCATATTACCAGCCCTTTCTAAAGGGAACATTGGGCATTGCTATTAGGCGTGGAGAAATCCAGGGTCTTCATAGTTTTCTGTTACGCCTTCATCCCAACAGTGACCAAAGAAATAATATAGTGAGAATATTCTGGGAGACCATGTTTGGGTGCAGTTTTGAAACTGGGGATAAAGAGACATTTGGGCAACAAATGAAAAAGGTGTGTACAGGACTGGAGGATCTGAGCACTGCAAACACACCTTACACTGATGTTTCAGGATTGAGGGCAACTTATAATGTGTATAAAGCAGTTTATGCCCTGGCCCATGCACTTCATGACCTGATGCAGTGTAAAGAGAAGAGAGGACCATTCAGTGGGAACAGCTGTGCTGACATAACAGATCTAAAACCCTGGCAGCTTGTTCACTACCTAAAAAAAGTGAATTTCACCACAAGCTTTGGGGATTCTGTATCATTTGACAATAATGGAGATGCTCTAGCCATCTATGATGTGTTGAACTGGCAGCCGAGCTCTGAAGAATCAATTAAACTTCACAATATCGGTGTAGTAAATGAGGTGGCAACAGAAATGGTGCTCACACTGAATAATGATGAAATTTACTGGAACTTTGAGGCACAAAAACCCCCACAGTCTGTGTGCAGTGAGAGCTGTCTCCCAGGCACCAGAAGAGCCATGAGGAAGGGCCTTCCTGTCTGCTGTTTTGACTGCCTGATTTGTGGAGATGGTGAAATTTCTAACACAACAGATGCTATTAAGTGCACAGTTTGTCCAGATGAATTTTGGTCCAATCTAAATAAGGATCAATGTGTTCCTAAAGAAATTGATTTTCTATCATATGAGGATCCTCTGGGCATCTCGCTGACCACTACTTCCCTGCTGGGAACCTGTTTTTGTGCTCTTGTGATGATAATTTTTACTTTTCACCGTAACACTCCTATAGTACGTGCCAACAATTCAGAGCTCAGCTTCCTGCTGCTTTTGTCACTCAAACTGTGTTTCCTGTGTGTGCTGCTGTTCATTGGTCAGCCCCAGTTGTGGACGTGTCAATTAAGACATGCTGTGTTTGGCATAAGCTTTGTCCTGTGCATCTCCAGCATTTTGGTCAAGACTATGGTGGTAATAGCTGTGTTCAAATCCTCTCGGCCTGAGGGCTCAGGAGCAATGAAATGGTTTGGAACAGCACAACAAAGATGCACAGTTCTGGTCCTAACAGCCCTCCAAATTGTAATATGTGCAGTCTGGCTATCAACTTCTTCTCCAACACCCTATAAAAATAACCAGTCTATCCGCTCTAAAATAGTCTATGAATGTGCCATTGGCTCATTGGCTGGGTTTTCTTTGCTGCTGGGATATATAGGATTGTTGGCAGCAATAAGCTTCCTACTAGCCTTTCTGGCTAGAAATCTTCCAGATAATTTTAATGAGGCAAAGTTTATCACTTTTAGTATGCTGATCTTCTGTGCGGTATGGGTTGCATTTGTTCCAGCATATATGAGCTCATCAGGGAAATATGCAGTGGCTATGGAGATATTTGCTATCTTAGCTTCTAGTTTTGGAGTGCTGGTGGCCATATTTGCCCCAAAGTGTTACATAATCATTTTACATCCGGAGAGAAACACTAAAAAAGCCATCATGGGAAGAGAAAACAAAAATAAA

>OlfCq5

ATGTGGGCAGTACTACTTTTTTGTTTGGTTCTGTCTTGTTGCTATATCTGTGTAACTTTGATAGCCAGTACAGGTACCTGTCAGCAACAGGGACACTTTACAATAAATGGGATGCACCAGGATGGAGACTTTGTCATTGGTGGTTTGTTTGATGTTCAGACATACCTAAAAGTATACCCTGAGATAAGCTTCAGAACGCAGCCAAAACTACCAAACTGTGAACTCTTCTATATGACAAGCTTCCAGCAAGCACTGACAATGGTTTTTGCCATCAGTGAGATTAATCACAATCCCAACTTGCTGCCAAACATCACACTTGGTTACCAGATCTATGACACTTGTTTAAGGCTTAGAGTGGCATTTCAGGCAGCTACAGCTCTGATAAGTGGGACAGAGGAGACCATCTCTGACTTCAACTGTAAAGGCCCACCACCAGTTATTGGACTCATAGGTGATCCAGGATCTACACATTCTATTGCAATTTCCAGTGTTCTGGGGCTGTTTCGAATGCCTATGATAAGCTACTATGCCACTTGCTCTTGTTTAAGTGACAAGAAAAAGTACCCCTCCTTCTTCAGAACAATTCCAAGTGATACCTTCCAAGTGCGGGCTATAGTTCAGACCTTGAGGCATTTTGGCTGGACCTGGGTTGGTCTGATCTACAGTAATAATGACTATGGTATCTACGCTGCTCAGTCCTTCCATCAAGAAATGCAGTTGTTTGGACACTGTGTTGCTTTTTCTGAAATCCTGCCCCAAGATAACAACCCCAGAGTTATTGATCACATTATGGGAGTAATTCAGGCCTCTACAGCTAGAGTAGTGGTTGTTTTTTCTGCTTCATCCTTATTGATTCCTTTGATGAACAAGGTAGTGTTGCAGAACTTAACAAGCAGGCAGTGGATAGCAAGTGAAGCCTGGGTCACCGCAGCTGTGTTCCGCACACCATATTTCCAGCCCTTTCTGAAGGGAACGTTGGGCATTGCTATTAGGCGTGGAGAAATCCAGGGTCTTCATAGTTTTCTGTTACGTCTTCATCCCAACAGTGACCAAAGAAATAATATAGTGAGGATATTCTGGGAGACCATGTTCGGGTGCAGTTTTGAAACTGGGGATAAAGAGACATTTGGTCAACAAATGAAAAAGGTGTGTACAGGACTGGAGGATCTGAGCACTACAAACACACCTTACACTGATGTTTCAGGATTGAGGGCAACTTATAATGTGTATAAAGCAGTTTATGCCCTGGCCCATGCACTTCATGACCTGATGCAGTGTAAAGAGAAGAGAGGACCATTCAGTGGGAACAGCTGTGCTGACATAACAGATCTAAAACCCTGGCAGCTTGTTCACTACCTAAAAAAAGTGAATTTCACCACAAGCTTTGGGGATTCTGTATCATTTGACAATAATGGAGATGCTCTAGCCATCTATGATGTGTTGAACTGGCAGCCGAGCTCTGAAGGATCAATTAAACTTCACAATATTGGTGTAGTAAATGAGGTGGCAACAGGAATGGTGCTCACACTGAATAATGATGAAATTTACTGGAACTTTGAGGCACAAAAACCCCCACAGTCTGTGTGCAGTGAGAGCTGTCTCCCAGGCACCAGTAGAGCCATGAGGAAGGGCTTTCCTGTCTGCTGTTTTGACTGCTTGATATGTGGAGATGGTGAAATTTCTAACACAACAGATGCTATTAAATGCACAGTTTGTCCAGATGAATTTTGGTCCAATCTAAATAAGGATCGATGTGTTCCTAAAGAAATCGATTTTCTATCATATGAGGATCCTCTGGGCATCTCGCTGACCACTACTTCCCTGCTGGGAACCTGTTTTTGTGCTCTTGTGATGATAATCTTTACTTTTCACCGTAACACTCCTATAGTACGTGCCAACAATTCAGAGCTCAGCTTCCTGCTGCTTTTGTCACTCAAACTGTGTTTCCTGTGTGTGCTGCTGTTCATTGGTCAGCCCCAGTTGTGGACGTGTCAGTTAAGACATGCTGTGTTTGGCATAAGCTTTGTCCTGTGCATCTCCAGCATTTTGGTCAAGACTATGGTGGTAATAGCTGTGTTCAAATCCTCTCGGCCTGAGGGCTCAGGAGCAATGAAATGGTTTGGAACAGCACAACAAAGATGCACAGTTCTGGTCCTAACAGCCCTCCAAATTGTAATATGTGCAGTCTGGCTATCAACCTCTTCTCCAACACCCTATAAAAATAACCAGTCTATCCGCTCTAAAATAGTCTATGAATGTGCCATTGGCTCAGTGGCTGGGTTTTCTTTGCTGCTGGGATATATAGGATTGTTGGCAGCAATAAGCTTCCTACTAGCCTTTCTGGCTAGAAATCTTCCAGATAATTTTAATGAAGCAAAGTTTATCACTTTTAGTATGCTGATCTTCTGTGCTGTATGGGTTGCATTTGTTCCAGCATATATGAGCTCATCAGGGAAATATGCAGTGGCTATGGAGATATTTGCTATCTTAGCTTCTAGTTTTGGAGTGCTAGTGGCCATATTTGCCCCAAAGTGTTACATAATCATTTTACATCCGGAGAGAAACACTAAAAAAGCCATCATGGGAAGAGAAAACAAAAATAAA

>OlfCq6

ATGTGGATAACTTTGCTTATTAATATTTACTTAATTTTAAAATGTATCTCTGCAGCTGTAGTCCTCAGAGCAGGTGCCTGTCAGCTCCAGGGACGTTTCAGGCTAAACGGAATGTACCAGGATGGAGATGTTATACTTGGTGGCATGTTTGAGGCTCATTTTTTCACTTTGTACCCTGAGCTGACTTTCAGAACAGAGCCAGCTCCACCATACTGTGAAATATTTAGTATGGAAAGTTTCCAGAATGTGCAAACCATGGCTTTTGCAATAAATGAGATTAATATGAATCCCAATCTTCTGCCTAATATCATTCTTGGTTACCATATTCATGACAACTGTGTGACGTTTGGAATGGCATTGCGAGCTGCCATGTCCCTGGTTAGTGGGATAGAGGAGTCCTTCATAAACCTAAACTGCACTGGTCCTCCTCCGATAATTGGAATTGTGGGGCATCCAAGTTCAACTCCTTCCATTGCAATTTCCAGTGTTTTGGGACTATTTCGAGTACCTATAGTTAGTCACTATGCTACGTGTTCCTGTTTGAGTGACAGAAAAAAGTACCCCTCTTTCTTCAGAACAATCCCCAGTGATGCCTTCCAGGTGCGGGCTATGATTCAACTCATTAGTCATTTTGGATGGACCTGGGTTGGTCTCCTCTACAGTAATGATGACTATGGTACCTATGCTGCTCAGTCCTTTCATCAAGAAATGCAGTTATTTGGAATTTGTATTGCTTTTTCTGAACCACTGCGGTATGATAGCAACCCCAGAGATATTCAACGTATAATGGCAGTGATTCAAGCCTCAACATCTAGAGTGGTGGTTGTTTTTTCTCCATCAACTTTAGTGATACCTTTAATAAATGAAGTGGTGTTGCAGAATATGACAGGCAGGCAGTGGATTGCAAGTGAATCTTGGGCCACTTCACCTGTCTTTTACACTCCACATTTCCTGCCCTTCCTTGGGGGCACACTTGGCATAGCCATCAGACGTGGAGAGATTGAGGGGCTCCGTGAATTTCTTTTACAGCTTCGTCCACAAAATGATCCAAGAAATAATATGCCTAAGATTTTCTGGGAGAACATGTTTGGATGTAGTTTTGAAACTGGGGCACAAGCAAAAAATGTGTGTACAGGACAGGAAGATCTGAGCACCACAAACACATCTTACACTGATGTTTCAGAATTGAGAGCAGCAAATAATGTCTATAAGGCAGTTTATGCCCTGGCACATGCACTTCATGACCTAATGAAGTGTGAGGAGGGGAAAGGACCATTCAGTGGAAACAGCTGTGCTGACATAACTAATCTAAAACCGTGGCAGCTTGTTCACTATCTACAGAAAGTGAACTTCTCCACACGCTTTGGGGATCATGTGTCATTTAATAAGAATGGAGATGCTCTGGCCATCTATGATGTGATGAACTGGCAGCCAGGCTCAGACAGATCAATAAGAATCCACACAGTAGGTGTAGTGAGGGAAGAGCCAGAAAAAGGATTGATGCTGACACTGGATGAGGATGCAATATACTGGAACTTTGAGACAAAAAAACCACCACAGTCTGTTTGCAGTGAAAGCTGCCCGCGAGGAAGCAGACAAGCCACAAGAAAGGGCCATCCTGTCTGCTGTTTTGACTGCCTGCCATGTAGAGATGGGGAAATTTCTAACACAACAGATCCTACTAAGTGCACAGTCTGTCCAGATGATTTCTGGTCTAATCTATATAAGGATCAGTGCATTCCCAAAGAAGTAGAGTTTCTGTCCTATTATGATCCTCTGGGCATATCTTTGACCACTGCTTCACTGCTTGGCACCTGTTTCTGTGCTTTTGTGATGGTCATCTTTGTGCATCACCATAACACTCCCATAGTACGAGCCAACAATTCAGAGCTCAGCTTCCTGCTGCTTTTCTCTCTCAAACTGTGTTTCCTGTGTGTGCTTCTCTTCATTGGTCGACCGCAGTTGTGGACATGTCAGTTAAGACATGCTGTGTTTGGCATAAGCTTTGTCCTGTGCATCTCCAGCATTCTGGTCAAGACTATGGTGGTAATAGCTGTGTTTAAGTCCTCTAGGCCTGAGGGCAAAAAAGCAATGAAATGGTTTGGAGCAGCTCAACAAAGATGTACAATTCTGGTCCTAACAGCCATCCAGGTTGTTATATGTGCAGTCTGGCTATCAACTGCCTCTCCAACTCCCCATAAAAACAACCTGTACATCCGCTCTATAATAGTATATGAATGTACTATAGGCTCAGTGACTGGATTTTCAATGCTTTTGGGATACATTGGACTGTTGGCAGCAGTAAGCTTCCTTATAGCCTTCCTGGCAAGAAATCTTCCAGATAATTTTAATGAAGCAAAGTTTATCACTTTTAGCATGTTGATCTTCTGTGCTGTATGGATTACATTTGTTCCAGCATATGTGAGCTCTCCAGGAAAATACTCGGTGGCTGTAGAAATATTTGCTATTTTAGCTTCTAGTTTTGGACTATTAGTGGCAATATTTGCCCCAAAGTGTTACATTATCCTTTTACATCCAGAAAGAAACAATAAAAAAACCATCATGGGAAGAGAGACACAT

>OlfCq8

ATGTGGATAACTTTGCTTATTAATATTTACTTAATTTTAAAATGTATCTCTGCAGCTGTAGTCCTCAGAGCAGGTGCCTGTCAGCTCCAGGGACGTTTCAGGCTAAACGGAATGTACCAGGATGGAGATGTTATACTTGGTGGCATGTTTGAGGCTCATTTTTTCACTTTGTACCCTGAGCTGACTTTCAGAACAGAGCCAGCTCCACCATACTGTGAAATATTTAGTATGGAAAGTTTCCAGAATGTGCAAACCATGGCTTTTGCAATAAATGAGATTAATATGAATCCCAATCTTCTGCCTAATATCATTCTTGGTTACCATATTCATGACAACTGTGTGACGTTTGGAATGGCATTGCGAGCTGCCATGTCCCTGGTTAGTGGGATAGAGGAGTCCTTCATAAACCTAAACTGCACTGGTCCTCCTCCGATAATTGGAATTGTGGGGCATCCAAGTTCAACTCCTTCCATTGCAATTTCCAGTGTTTTGGGACTATTTCGAGTACCTATAGTTAGTCACTATGCTACGTGTTCCTGTTTGAGTGACAGAAAAAAGTACCCCTCTTTCTTCAGAACAATCCCCAGTGATGCCTTCCAGGTGCGGGCTATGATTCAACTCATTAGTCATTTTGGATGGACCTGGGTTGGTCTCCTCTACAGTAATGATGACTATGGTACCTATGCTGCTCAGTCCTTTCATCAAGAAATGCAGTTATTTGGAATTTGTATTGCTTTTTCTGAACCACTGCGGTATGATAGCAACCCCAGAGATATTCAACGTATAATGGCAGTGATTCAAGCCTCAACATCTAGAGTGGTGGTTGTTTTTTCTCCATCAACTTTAGTGATACCTTTAATAAATGAAGTGGTGTTGCAGAATATGACAGGCAGGCAGTGGATTGCAAGTGAATCTTGGGCCACTTCACCTGTCTTTTACACTCCACATTTCCTGCCCTTCCTTGGGGGCACACTTGGCATAGCCATCAGACGTGGAGAGATTGAGGGGCTCCGTGAATTTCTTTTACAGCTTCGTCCACAAAATGATCCAAGAAATAATATGCCTAAGATTTTCTGGGAGAACATGTTTGGATGTAGTTTTGAAACTGGGGCACAAGCAAAAAATGTGTGTACAGGACAGGAAGATCTGAGCACCACAAACACATCTTACACTGATGTTTCAGAATTGAGAGCAGCAAATAATGTCTATAAGGCAGTTTATGCCCTGGCACATGCACTTCATGACCTAATGAAGTGTGAGGAGGGGAAAGGACCATTCAGTGGAAACAGCTGTGCTGACATAACTAATCTAAAACCGTGGCAGCTTGTTCACTATCTACAGAAAGTGAACTTCTCCACACGCTTTGGGGATCATGTGTCATTTAATAAGAATGGAGATGCTCTGGCCATCTATGATGTGATGAACTGGCAGCCAGGCTCAGACAGATCAATAAGAATCCACACAGTAGGTGTAGTGAGGGAAGAGCCAGAAAAAGGATTGATGCTGACACTGGATGAGGATGCAATATACTGGAACTTTGAGACAAAAAAACCACCACAGTCTGTTTGCAGTGAAAGCTGCCCGCGAGGAAGCAGACAAGCCACAAGAAAGGGCCATCCTGTCTGCTGTTTTGACTGCCTGCCATGTAGAGATGGGGAAATTTCTAACACAACAGATCCTACTAAGTGCACAGTCTGTCCAGATGATTTCTGGTCTAATCTATATAAGGATCAGTGCATTCCCAAAGAAGTAGAGTTTCTGTCCTATTATGATCCTCTGGGCATATCTTTGACCACTGCTTCACTGCTTGGCACCTGTTTCTGTGCTTTTGTGATGGTCATCTTTGTGCATCACCATAACACTCCCATAGTACGAGCCAACAATTCAGAGCTCAGCTTCCTGCTGCTTTTCTCTCTCAAACTGTGTTTCCTGTGTGTGCTTCTCTTCATTGGTCGACCGCAGTTGTGGACATGTCAGTTAAGACATGCTGTGTTTGGCATAAGCTTTGTCCTGTGCATCTCCAGCATTCTGGTCAAGACTATGGTGGTAATAGCTGTGTTTAAGTCCTCTAGGCCTGAGGGCAAAAAAGCAATGAAATGGTTTGGAGCAGCTCAACAAAGATGTACAATTCTGGTCCTAACAGCCATCCAGGTTGTTATATGTGCAGTCTGGCTATCAACTGCCTCTCCAACTCCCCATAAAAACAACCTGTACATCCGCTCTATAATAGTATATGAATGTACTATAGGCTCAGTGACTGGATTTTCAATGCTTTTGGGATACATTGGACTGTTGGCAGCAGTAAGCTTCCTTATAGCCTTCCTGGCAAGAAATCTTCCAGATAATTTTAATGAAGCAAAGTTTATCACTTTTAGCATGTTGATCTTCTGTGCTGTATGGATTACATTTGTTCCAGCATATGTGAGCTCTCCAGGAAAATACTCGGTGGCTGTAGAAATATTTGCTATTTTAGCTTCTAGTTTTGGACTATTAGTGGCAATATTTGCCCCAAAGTGTTACATTATCCTTTTACATCCAGAAAGAAACAATAAAAAAACCATCATGGGAAGAGAGACACAT

>OlfCq9

ATGTGGGCAATTCTACTTTTTTGTTTGTTCCTGTCTTGTAATTTCATCTGTGTAACTTTGATGGTTAGTTCAGGTACCTGCCAGAAACAGGGACATTTTACATTAAATGGGATGCACCAGGATGGAGATTTTGTCATTGGTGGTCTTTTTGAGATTCAGAGCTATGTGAAAGTATACCCTGAGATAAGCTTCAGAACACAGCCAAAACTACCAAACTGTGACCTCTTCTATATGACAAGCTTCCAGCAAGCACTAACAATGGTTTTTGCCATCAGTGAGATTAATAACAATCCTAACCTGCTACCTAACATTACACTTGGTTACCAGATCTATGACAACTGTTTAAGGCTTGGAGTGGCATTTCGGGCAGCTACAGCTCTGATAAGTGGGACAGAGGAGACAATCTCTGACCTCAACTGTAAAGGCCCACCACCAGTTATTGGAGTCATAGGTGATCCAGGATCTACACATTCTATTGCAATTTCCAGTGTTCTGGGGCTTTTTCGGCTGCCTATGATTAGCTACTACGCCACTTGCTCTTGTTTAAGTGACAAGAAAAAGTACCCTTCCTTCTTCAGGACAATCCCAAGTGATACCTTCCAAGTGCGGGCTATGGTTCAGACCATGAGGCATTTTGGCTGGACCTGGGTTGGTCTGATCTACAGTAATAATGACTATGGTATCTACGCAGCTCAGTCCTTTCATAAAGAAATGCAGTTATTTGGACACTGTGTTGCTTTTTCTGAAATCCTGCCCCAAGATAACAACCCCAGAGTTATTGATCACATTATAGGAGTAATTCAGGCCTCTACAGCTAGAGTAGTGGTTGTTTTTTCTGCTTCATCCCTATTGATTCCTTTGATGAACAAGGTAGTGTTGCAGAATTTGACAGGCAGGCAGTGGATAGCGAGTGAAGCCTGGGTCACCTCAACTGTATTTCGCACTCCATATTACCAGCCCTTTTTGAAGGGAACGTTGGGCATTGCTATTAGGCGTGGAGAAATCCAGGGCCTTCATAGTTTTCTTTTACGCCTTCATCCCAACAGTGACCAAAGAAATAATATAGTGAGGATATTCTGGGAGACCATGTTTGGGTGCAGTTTTGAAACTGAAGATAAAGAGACATTTGGGCAACAAATAAAAAAGGTGTGTACAGGACAGGAGGATCTGAGCATCACAAACACACCTTACACTGATGTTTCAGGTTTGAGGGCACCTTATAATGTGTATAAAGCAGTTTATGCCCTGGCCCATGCACTTCATGACCTAATGCAGTGTAAAGAGAAGAGAGGACCACTCAGCGAAAAAAACTGTGCTGACATAACAGATCTAAAACCCTGGCAGCTGCTTTCCTACCTAAAAAAAGTGAATTTTACCACAGGCTTTGGGGATCCTGTGTCATTTGACAATAATGGAGATGCTCTAGCCATCTATGATGTTTTGAACTGGCAGCCGAGCTCTGAAGGATCAATTAAACTTCACAGTATTGGTGTAGTAAATGATGAGGTGGCAACAGGAATGGTGCTCAGACTGAATAAGGATGAAATTTACTGGAACTTTGAGGCACAAAAAGCCCCACGGTCTGTGTGCAGTGAGAGCTGTCCCCCAGGCACCAGAAAAGCCATGAGGAAGGGCTTTCCTGTCTGCTGTTTTGACTGCCTGACATGTGGAGATGGTGAAATTTCTAACACAACAGATGCTATTAAGTGCACAGTTTGTCCAGATGAATTTTGGTCCAATTTTAATAAGGATCAGTGTGTTCCTAAAGAAATAGAGTTTCTATCATATGAAGATCCTCTAGGCATCTCTCTGACCACTGCTTCCCTGCTAGGAACCTGCTTCTGTGCTCTTGTGATGATAATCTTTGCTTTTCATCATAACACTCCTATAGTACGTGCCAACAATTCAGAGCTCAGCTTCCTGCTGCTTTTGTCACTTAAACTGTGTTTTCTGTGTGTGCTGCTGTTCATTGGTCAGCCACAGTTGTGGACGTGTCAATTAAGACATGCTGTGTTTGGCATAAGTTTTGTCCTGTGCATCTCCAGCATTCTGGTGAAGACTATGGTGGTAATAGCTGTGTTCAACTCCTCTCGGCCTGAGGGCAAAGGAGCAATGCAATGGTTTGGAGCAGCTCAACAAAGATGTACAGTTCTAGTCCTCACAGTCCTCCAAGTAGTAATATGTGCTGTCTGGCTATCAACTTCTTCTCCAACACCACATAAAAATAACCAGTATGTCCGCTCCAAAATAGTCTATGAATGTGCCATTGGCTCAGTGGCTGGGTTTTCTTTGCTGCTAGGATATATAGGATTGTTGGCGGCAATAAGCTTTCTACTAGCCTTCCTAGCAAGAAATCTTCCAGATAATTTTAATGAGGCAAAGTTTATCACTTTTAGCATGTTAATCTTCTGTGCTGTATGGATTGCATTTGTTCCAGCATATGTGAGCTCTCCAGGAAAATATGCAGTGGCTGTGGAGATATTTGCTATCTTAGCTTCTAGTTTTGGAGTGCTGGTGGCCATATTTGCCCCAAAGTGTTACATAATTATTTTACATCCAGAAAGAAACACTAAAAAAGCCATCATGGGAAGGGAAACCAAAAAATAT

>OlfCq10

ATGTGGGCGATTCTACTTTTTTGTTTGTTCCTGTCTTGTAACTACATCTGCGTAACTTTGATGGTTAGTTCAGGTACCTGTCAGCAACAAGGACACTTTACATTAAATGGGATGCACCAGGATGGAGACTTTGTCATTGGTGGTCTTTTTGAGATTCAGAGGTACCTGAAAGTATACCCTGAGATAAGTTTCAGAACGCAGCCAAATCTACCAAACTGTGAACTCTTTTACATGACAAGCTTCCAGCAGGCATTAACAATGGTTTTTGCCATCAGTGAGATTAATAGCAATCCCAACCTGCTGCCTAACATCACACTTGGTTACCAGATCTATGACAACTGTTTAAGGCTTGGAGTGGCATTTCGGGCAGCTACAGCTCTGATAAGTGGGACAGAAGAGACCATCTCTGACCTCAACTGTAAAGGCCCACCACCAGTTATTGGACTCATAGGTGATCCAGGATCTACACATTCTATTGCAATTTCCAGTGTTCTGGGGCTTTTTCGGCTGCCTATGATTAGCTACTACGCCACTTGCTCTTGTTTAAGTGACAAGAAAAAGTACCCCTCCTTCTTCAGAACAATTCCAAGTGATACCTTCCAGGTGCGAGCTATGGTTCAGACCTTGAGGCATTTTGGCTGGACCTGGGTTGGTCTGATTTACAGTAATAATGACTATGGTATCTACGCAGCTCAGTCCTTTCATCAAGAAATGCAGTTGTTTGGACATTGTGTTGCTTTTTCTGAAATCCTGCCCCAAGATAACAACCCCAGAGTTATTGATCACATTATGGGAGTAATTCAGGCCTCTACAGCTAGAGTAGTGGTTGTTTTTTCAGCTTCATCTGTATTGATACCTTTAATGAATGAGGTGGTGTTACAGAATTTGACAGGCAGGCAGTGGATAGCGAGTGAAGCCTGGGTCACCTCAGCTGTATTTCGCACACCATATTTCCAGCCCTTTCTAAAAGGAACGTTGGGCATTGCTATTAGGCGTGGAGAAATCCAGGGCCTTCATAGTTTTCTGTTACGTCTTCATCCCAAGAGTGACCAAAGAAATAATATAGTGAGGATATTCTGGGAGACCATGTTCGGGTGCAGTTTTGAAACTGGGGATGAAGTGACATTTGTTCAACAAATGAAAAAGGTGTGTACAGGACAGGAGGACCTGAGCATCACAAACACACCTTACACTGATGTTTCAGGATTGAGAGCACCTTATAATGTGTATAAAGCAGTTTATGCCCTGGCCCATGCACTTCATGACCTGATGCAGTGTAAAGAGAAGAGAGGACCACTCAGTGAGAACAGCTGTGCTGACATAACTGATCTAAAACCCTGGCAGCTGGTTCCCTACCTAAAAAAAGTGAATTTTACCACAGGCTTTGGGGATCCTGTGTCATTTGACAATAATGGAGATGCTCTTGCCATTTATGATGTTCTGAACTGGCAGCCGAGCTCTGAAGAATCAATTAAACTTCACACTATTGGTGTAGTAAATGATGAGGTGGCAACAGGAATGGCCCCACGGTCTGTGTGCAGTGAGAGCTGTCCCCCAGGCACCAGAAAAGCCATGAGGAAGGGCTTTCCTGTCTGCTGTTTTGACTGCCTGACATGTGGAGATGGTGAAATTTCTAACACAACAGATGCTATTAAGTGCACAGTTTGTCCAGATGAATTTTGGTCCAATTTTAATAAAGATCAGTGTGTTCCTAAAGAAATAGAGTTTCTATCATATGAAGATCCTCTAGGCATCTCTCTGACCACTGCATCCTTGCTAGGAACCTGCTTCTGTGCTCTTGTGATGATAATCTTTACTTTTCACCGTAACACTCCTATAGTACGTGCCAACAATTCAGAGCTCAGCTTCCTGCTGCTTTTGTCACTCAAACTGTGTTTCCTGTGTGTGCTGCTGTTCATTGGTCAGCCACAGTTGTGGACGTGTCAATTAAGACATGCTGTGTTTGGCATAAGTTTTGTCTTGTGCATCTCCAGCATTCTGGTCAAGACTATGGTGGTGATAGCTGTGTTCAACTCCTCTCGGCCGGAGGGCACAGGAGCAATGAAATGGTTTGGAGCAGCTCAACAAAGATGTACAGTTCTAGTCCTCACAGCCCTCCAAGTAGTAATATGTGCTGTCTGGCTATCAACTTCTCCTCCAACACCACATAAAAATAGCCAGTATGTCCGCTCTAAAATAGTCTATGAATGTGCCATTGGCTCAGTGGCTGGGTTTTCTTTGCTGCTGGGATATATAGGATTGTTGGCAGCAATAAGCTTTCTACTAGCCTTCCTGGCAAGAAATCTTCCAGATAATTTTAATGAGGCAAAGTTTATCACTTTTAGCATGTTAATCTTCTGTGCTGTATGGATTGCATTTGTTCCAGCATATGTGAGCTCTCCAGGAAAATATGCAGTGGCTGTGGAGATATTTGCTATCGTAGCTTCTAGTTTTGGAGTGCTGGTGGCCATATTTGCCCCAAAGTGTTACATAATCATTTTACATCCAGAAAGAAACACTAAAAAAGCCATCATGGGAAGAGAAACCAAAAATAAT

>OlfCq11

ATGACAAACACAATATCTTTATTTTTAGAGTGGTACCGCCTTTCAAGAAGAAAAAAAAAGGCTTTATATATATGTGATCTCAATGACGAAGGACATAAACAGATAAGAGGGATGTGGGTGATTCTGCACATTTATTTTTTTATATCTTGTAATTATATGTCTGTGGCTTTGATGGCCAGTTCAGGAACCTGTCAACTACAAGGACACTTTACATTGAACGGGATGTACCAGAATGGAGACTTTTTAATTGGTGGTTTATTTGAGATTCAATACCTCAAAGCATTCCCGGGGCTAAGCTTCAGAACAGAGCCAAAACTACCCCACTGTGAAAACTTCTATATGACAAGCTTCCAGCAGGCAATAACAATGGTTTTTGCCATCAATGAGATTAATAACAATCCCAACCTGCTGCCTAACATCACACTTGGTTACCAGATGTATGACAACTGTTTAAGGCTTGGAGTGGCATTTCGGGCAGCTACAGCTCTTATAAGTGGGACAGAGGAGACCCTCTCTGACCTCAACTGCAAAGGCCCACCACCAGTTATTGGAGTCATAGGTGATCCAGGATCTACACATTCTATTGCAATTTCTAGTGTTTTGGGGCTTTTTCGAATGCCTATGATTAGCTATTATGCCACCTGTTCCTGTTTGAGTAACAGGAAAAAGTACCCCTCTTTCTTCAGAACAATACCCAGTGATGCTTTCCAGGTGCGGGCTATGATTCAAATTTTGAGGCATTTTGAATGGACCTGGGTTGGTCTCCTCTACAGTGATGATGATTATGGCATAAATGCTGCTCTGTCTTTCCACCATGATGTGCAGCAATTTGGAGGTTGCGTGGCTTTCTCTGAGATTCTCCCCAATGATAACAACCAGATGGCAGTACAACACATAGTCAGAGTCATTCAGAACTCTACAGCAAAAGTAGTGGTTGTGTTTTCCACTTCAAGCTATTTGTTGCCTGTGATTGATGAGATGCTGTTGAAAAATATTACAGGCAGGCAATGGATTGCTAGTGAGGCTTGGTCCACCTCACCTGTGCTTCTTAATCCCCGACTTAGGTATGTTTTAGGGGGTACACTGGGAATTGCCATCCGACGGGGAGAGATTGAAGGACTTGACAACTTTCTGTTACGCCTTCGTCCCGACAATTTTTCACAAAACAGTATGATGAGGATATTCTGGGAGAACATGTTTGAATGCAATTTCGACACTATTGGAGGACTGAGAACCAAATTGTGCTCAGGGCAAGAGGATTTGAGAAGCAAATTTACACCATACACTGATGTTTCTGAGCTGAGAGCTTCTTATAATGTCTATAAGGCAGTTTATGCTCTAGCACACGCACTACATGACCTAATGCATTGTGAGGAGGGGAGAGGACCATTCAGTGAGAACAGATGTGTTGACATATCAAATCTAAAACCTTGGCAGCTGGTTCACTACCTTCAAAGAGTGAAATTTACCACAGGCTTTGGGGATCATGTGTCCTTTGACAAAAATGGAGATGCTCTGGCCATCTATGATGTGCTGAACTGGCAGCTGAGCTCTGAGGAGTCAGTAACTGTCAGAAGAATTGGTGTTGTAGATGAAGGGGTGACAACAGGGAAGGTGTTCACACTAGATGAAAATGCAATATACTGGAACTTTGAGACAAATAAACCTCCACGGTCTGTGTGCAGTGAAAGCTGCCCCCCTGGAACTAGACAAGCAACAAGGAAAGGGCTTCCTGTCTGCTGTTTTGACTGCTTGCCATGTGGAGATGGAGAGATTTCCAATACAACAGATACCACTGAGTGCATAGCATGTCCAAATGATTTCTGGTCCAACCCAGAAAAAGATCAATGCATCCCAAAAGAAGTAGAGTTTCTCTCGTATGAGGATCCTTTGGGCATTTCTCTGACCACTGCGGCCATGCTTGGCACTTTCATCTGTGCTCTTGTAATGACCATTTTTGCTCATTACCGCAACACTCCTGTAGTACGTTCAAATAATTCAGAGCTCAGCTTCCTGCTTCTGTTGTCCCTTAAACTCTCTTTCTTGTGTGTGCTGCTGTTCATTGGTCAGCCACAGTTGTGGACGTGTCAATTAAGACATGCTGTGTTTGGCATAAGCTTTGTTCTGTGTGTCTCCAGTATCCTGGTTAAGACTATGGTGGTAATAGCTGTCTTTAAATCATCACGACCCGAGGGTAAAAATGCCATGAAATGGTTTGGAGCAGCTCAACAAAGAGGTACAGTCCTGATCCTAACAGCTCTTCAAGTTTTGATATGTGCAGTCTGGCTGTCAACTGCTTCTCCAACACCTCATAAAAACAGCCGGTATATCAGATCTATTATAGTATATGAATGTGCCATTGGCTCAGTGGCTGGATTTTCTCTGCTGCTGGGATACATTGGACTTTTAGCAGCAGTAAGCTTCCTGCTGGCCTTCCTAGCCAGAAACCTTCCAGATAGTTTTAATGAAGCAAAGTTCATCACTTTTAGCATGCTGATCTTTTGTGCTGTGTGGATTGCATTTGTTCCAGCATATGTGAGCTCTCCAGGGAAGTATGCTGTGGCTGTGGAGATTTTTGCCATACTAGCTTCTAGTTTTGGCTTACTGCTAGCAATATTTGCCCCGAAGTGCTACATCATTATATTACACCCAGAAAGAAACACTAAGAAAGCCATTATGGGAAAAGGTACAAAAAATAAA

>OlfCq12

ATGTTAACATGGCACCGCCTGTTTATTAAGGCAACGCCCTCCAAAGTATTTATTATAAATAATATCCATAGGGGTGAGCTAAAAAGAAATGTGGAAATGTGGATCATTGCAAAAATCTGCCTGTACTTGTCCTTCAGTTGTATCTCTGTAGCTTCTATCTTCAGATCAGGTCCCTGTCAGCTCCAGGGGCAATTCAGGCTGAACGGGATGTTCCAGGAAGGAGATCTCATCCTTGGAGGGCTATTTGAGGTTCACTTCCTCACAGTGTTCCCAGAGCAGAGCTTCAGAACAGAGCCAGAACCACCATACTGTGAGCAATTTGATATGGCAAGTTTCCAGCAGGCACAGACTATGGTCTTTGCAATAGACGAAATCAATAGGAATCCAAACCTGCTTCCGAACATCACTCTTGGTTATTATCTTTATGACAACTGTGTCAAGTTGAGTGTGGCATTCAGGGCTGCTATGGCTCTTGTTAGTGGCACAGAGGAATCATTCTCCAGCCTAAACTGCACTGGCCCTCCACCAGTGATTGCCATAGTGGGGGACCCTGGATCTACCCACTCAATTGCAATCTCTAGTATATTAGGGCTGTTTAGAGTGCCTATGGTTAGCTACTTCGCAACCTGCTCCTGTTTAAGTGACAGGAAAACATACCCATCTTTCTTCAGAACAATACCCAGTGATGCTTTCCAGGTGCGGGCTATGGTTCAGATCTTAAAGCGTTTTGGATGGACCTGGGTTGGTCTGCTCTACAGTGATGATGACTATGGAATCTACGCTGCTCAGTCATTCCAGAAGGAAATGCAGCTCTTTGGAGCATGTGTTGGCTTTTCTGAAATGCTGCCTCGTGATAATAACCATGAAGATATCCAGAGTATTGTGGAAGTGATTCAGACTTCAACAGCTAGAGTGGTGGTGGTGTTCTCCACAGAAGCCTATTTATTACCACTGATGGATGAGGTGGCATTGAAAAATGTGACAGGCAGGCAGTGGATTGCGAGTGAAGCTTGGGCCACCTCTCCTGTGTTTCATACTCAGCATCTCCTGCCCTTCCTTGGGGGCACACTGGGCATTGCAATAAGGCGTGGGGAGATCCAGGGCCTTAAAGAATTTCTGTATTACCTCCATCCTGACAGCAATACAAGGAACAATATTGTAAGAATCTTTTGGGAGAACATTTTTGGGTGCAGTTTTGAGATTGGAGGCAGAGAGAGAAAGGTTTGTACAGGGCAAGAAGATCTGAAAAGCACAAACACAGCATACACTGATGTATCAGGACTGAGGGCCTCTTATAATGCATATAAAGCTGTTTATGCTCTAGCCCATGCACTTCATGACCTGATGCAGTGTGAAGATGGGAGAGGACCATTTAGTGGAAACAAATGTGCAGACCAAATCAACCTACAGCCCTGGCAGGTAGTCCATTATCTTCAAAAAGTAAACTTTACCACAGGATTTGGAGATCATGTGTCATTTGATGAGAATGGAGATGCTCTGGCCATCTATGATGTGATGAACTGGCAGCCGAGTTCAGATGGTGCAATAAGTGTTAGCACAGTTGGTGTGGTGAATGAAGGGGCTTCAATGAAGATGGTGCTCACACTGAAGGAGAATTCAATATTCTGGAACTTTAAAAACAGAAAACCCCCACAGTCTGTGTGTAGTGAGAGCTGCCCCCCAGGCACCAGACAAGTCAGGAGGAAGGGCCTTCCTGTCTGCTGTTTTGATTGCCTGCCATGCGCAGATGGAGAGATTTCTAACACAACTGATGCTATTGAGTGCAAAATATGTCCTGATGAGTTATGGCCCAATCCAAATAAAGATCAGTGTGTCCCAAAAGAAGAAGATTTTTTGTCCTTTGAGGATCCTCTGGGCATCTCTCTGACCACTGCTTCCCTGCTTGGCACCTGCTTCTGTGCTCTTGTTATGGTCATCTTCTCTCATCATCGTAACACTCCTGTAGTTCGTGCCAACAATTCAGAGCTCAGCTTCCTGCTACTTTTGTCACTCAAACTGTGTTTTCTGTGTGTGTTGCTGTTCATTGGACGACCAAAGTTATGGACATGTCAGTTAAGGCATGCTGCATTTGGCATAAGTTTTGTTTTATGTGTCTCCAGTATCCTGGTCAAGACTATGGTGGTAATAGCCGTGTTTAAATCATCTCGGCCTGAGAGTAAAAGTGCCATGAAATGGTTTGGGGTAGCTCAACAAAGAGGGACAGTTATGGCCCTAACCACCCTCCAGATTGTGATATGTACAGTTTGGTTAAGCATGGCATCCCCAAAACCCTATAAAAACAGCCTGTATATCTCCTCCAAAGTTGTCTATGAATGTGATATTGGCTCAGTAGTTGGTTTTTCTCTATTGCTGGGATACATTGGTCTTCTGGCAGCAGTAAGCTTTCTATTTGCCTTCCTGGCAAGAAACCTTCCAGATAATTTTAATGAAGCCAAATTTATTACATTCAGTATGTTGATTTTCTGTGCTGTGTGGATTACATTTGTTCCAGCTTATGTGAGTTCACCAGGAAAATATGCAGTGGCTGTGGAGATATTTGCAATTTTAGCTTCCAGTTTTGGATTACTGGTGGCTATATTTGCCCCAAAGTGCTACATGATCCTTTTTCATCCAGAGAGAAACACTAAAAAATCAATCATGGGAAGAGCCACACAAATTAAA

>OlfCq13

ATGTGGAGCCCTTTAATCATTTGTCTATATCTGTCCTATAACTTTAAGTCTGCAGCTTCAATCCTTATGGGTTCTTGCCAGCTCCAGGGACACTTTGGTTTAAATGGAATGTACCAGGCTGGAGATGTTATTCTTGGAGGGCTGTTTGAAGTTCACTTACTTGCAGTGTTCCCAGAGCTAAGCTTTAGATCAGCACCCGAACCACCTTACTGTGAACAATTCGATATGGCAAGCTTCCAGCAGGCACAGACTATGGTTTTTGCCATAGATGAGATCAACAAGAACCCAAAACTGCTTCCTAATATTACTCTTGGTTATCATCTTTATGACAACTGTGTGATGCTAGGAATGGCATTCCGGGCTGCCATATCCCTAGTTAGTGGAACAGAGGAATTGTTCTCTAACCTTAACTGTACTGGCTCACCTCCGGTGATTGGCATTGTGGGGGATTCAAATTCCACTCCCTCTATTGCAATTTCCAGTGTTTTAGGACTGTTTCGAGTGCCTATAGTTAGCTATTTTGCAACCTGTTCCTGTTTGAGTAATAGAAAACAATACCCATCCTTCTTTAGAACAATCCCCAGTGATGCCTTCCAAGTGAGGGCTATGGTTAATATTTTAAAACATTTTGGATGGACCTGGGTTGGTCTTATCTACAGTGATGATGATTACGGCAACTATGCCGCTCAGTCCTTTCTTCAGGACATTCAGATATATGGAGGGTGTGTTGCTTTTACTGAAATTCTGCCTCTAAATAACAACCGCAAATATATTGAACATGTAGTTGGAGTAATTCAGGCCTCTACAGCAAGGGTGGTAGTGGTGTTTTCGACTTCGACTTATGTGTTGCCTTTAATGGACGAGGTGGTATTGCAGAATGTAACAAACAGACAATGGATTGCAAGTGAAGCTTGGGCTACTTCACCTATGTTTCACACTCAGCGCCTTTTGCCTTTCCTGGGGGGCACACTGGGTATCGCCATCAGGCGTGGAGAGATCCAGGGACTTCATGAGTTTCTGCTACATCTTCGTCCTAAAAACGATCAACATAATAACATGGTAAGAATTTTCTGGGAGAAAATGTTTGGGTGCAGATTTAACCCTGGAGGCAAAGGAGACAAACAGTGCTCAGGGCAAGAGGATCTGAGCAGCATAGATACAGCTTACACTGATGTCAAAGAGCTGAGGGCATCATATAATGTGTATAAAACGGTTTATGCCCTGGCACATGCCCTTCATGACCTGACGGAGTGTGATGAGGAGAGAGGACCATTCAGTGAAAACAGATGTGCTGACATAACCAATTTACAACCCTGGCAACTGGTCCACTACCTACAGAAAGTCAACTTTACCACAGGCTTTGGGGATCATGTGTCATTTGATAAGAATGGAGATGTACTGGCCATCTATGATGTGATGAACTGGCACCCAAGCTCTGATGGATCGATAAGTGTCCGCACAGTTGGAGTGGTAAATGAAGGAGCAGCATCAGGAAAGGTGCTCACACTGGATGAAGATGCAATATACTGGAACTTTAAGATAAAGAAAGCTCCACAGTCTGTGTGCAGTGAGAGCTGTCCCCCAGGAACCAGACAAGCAACAAAAAAAGGGCTTCCTGTGTGCTGTTTTGACTGTCTGCCATGTGGAGATGGGGAGATTTCTAATGTAACAAATGCTATTGAGTGCACAGTATGTCCAGACGAGTTCTGGTCTAATAAAGATAAGGATCAGTGTGTACCCAAAGAAGTAGAGTTTCTGTCTTATGAGGATCCCTTGGGCATCTCTCTGACTACTGCTTGCCTGCTTGGCACCTGCTTCTGTGCTCTTGTAATGATCATCTTCTGTCAACATCGTAACACTCCCATAGTACGAGCCAACAATTCAGAGCTTAGCTTCCTGCTTCTATTGTCACTCAAACTGTGTTTCCTGTGTGTGCTGCTGTTCATTGGCCGGCCACAGTTGTGGACATGTCAGTTAAGACATGCTGTGTTTGGCATAAGCTTTGTTTTGTGTGTGTCCAGTATCCTGGTTAAGACTATGGTGGTAATAGCCGTGTTCAAGTCCTCTCGACCAGAGGGAAAAGGGTCTATGAAATGGTTTGGGACAACCCAGCAAAGATGTACTGTCCTCATCCTAACTGCACTCCAGGTAGTAATATGCATAGTCTGGCTATCAAATTCTTCTCCAGCACCCCATAAAAACAGCCAGCATATCAGTTCCAAAATTGTTTATGAATGTGCCATTGGCTCATTGGCTGGTTTTTCTCTGCTATTGGGATACATTGGTCTTCTGGCAGCAGTAAGCTTTCTGTTAGCCTTCTTAGCAAGAAACCTTCCAGATAATTTTAATGAAGCAAAGTTCATCACTTTTAGCATGTTGATTTTCTGTGCTGTTTGGATTGCATTTGTTCCAGCATATGTGACCTCTCCAGGGAAATATGCAGTGGCTGTGGAAATTTTTGCTATTCTTGCCTCAAGTTTTGGATTATTAGGAGCAATATTTGCCCCAAAATGCTATATTATCCTTTTACATCCAGAGAAAAATACAAAAAAAGCCATCATGGGTAGACAAATAACTAAA

>OlfCq14

ATGTGGATCATTCTAAACATATTCTTGTGTCTGTCTAAATGGATCTCTGCAGATTCAGTCCTCAGATTAGACACATGTCAGCTTCAGGGACACTTCATGTTGAATGGGGTGTACAAACATGGAGATGTTATACTAGGAGGGTTGTTTGATGTTCACTTACTTACAGTTTTCCCACAGCTGAGCTTCAGAGTGCAGCCAAAGCCACCATACTGTGAAAAGCAAGTGTGTATGGAAAACTTCAAATCGTCACAGACAATGGCTTTTACTATAGATGAGATCAATAATAATCCAAATCTGCTACCCAACATCACTCTTGGATATCATCTTTATGACAACTGTGTGAGACTAGCAATGGCATTTCGGGCTGCCATGTCTCTGGCTAGTGGGACAGAGGAATCCTTCTCCAACTTAAACTGCACTGGCCCTCCTCCGGTGATTGGGATTGTAGGAGATCCAAGTTCAACTCCTTCTATTGCAATTTCCAGTATCCTGGGGCTGTTTCATGTACCTATAGTTAGTCACTACGCCACCTGCTCTTGTTTGAGTGACAAGAAGAAATACCCCTCTTTTTTCAGAACAATCCCTAGTGATGCCTTTCAGGTGCAGGCTATGGTTCAGGTTTTGAGGCATTTTGGATGGACCTGGGTTGGCCTTCTTTACAGTGATGATGACTATGGCACCTATGCAGCACAGTCCTTCCAAAAGGAAGTTCAGCTTTTTGGAGGTTGTATCGCTTTTTCTACAATACTGCCCCAGAACAACCCTAGAGAGATTCAGAATATAATGGGAGTCATTCGGGCTTCTACAGCTAGAGTAATAGTTGCAATTTCTACATCATCTTATCTGCTGCCTTTGATGGATGAAGTGGTATTGCAAAACCTGACAGGTAGACAGTGGATTGCAAGTGAAGCTTGGGCCACCACACCAGCATTTCGCACTCCACGCTTTCTGACAATTTTGAGGGGAACAATTGGCATTGCTATCAGACGTGGTGAGATCCAGGGACTTCTTGAATATCTTTTACGTCTCCGTCCTAGCAGTGATCCAAGAAATAATTTGGTGAGAATCTTTTGGGAGAACATGTTTGGGTGCAGTTTTGCATCGCAAACAGTAGGTGAGCAAGTAAAAAAGTTGTGCACAGGACAGGAGGATCTGAGCATCACAAACACAGCATACACTGATATTTCAGGGTTGAGAGGCCCTTATAATGTTTATAAAGCAGTTTATGCCCTGGCACATGCACTTCATGGACTGATGCAATGTGAAGAGGGGAGAGGACCATTCAGTGGGAACAGCTGTGCTGACATAACAAATCTGAAACCCTGGCAGCTAATCCACTATCTACAAAAAGTGAACTTCATCACAGGCTTTGGGGATCGTGTGTCATTTGATAAGAATGGAGATGCTCTGCCCATCTATGATGTGATGAATTGGCAACCAAGCTCTGATGGTTCAATACGAGTCCAAACAGTTGGTGTAGTAAATAAGGGAGTGACATCAGGTATGGTGCTCAATTTGGTTGAAGATGCAATATACTGGAACTTTGAAACAAAAAAGCCCCCGCAGTCTATGTGCAGTGAGAGCTGCCCCCTAGGCACCAGAAAAGCCAGAAGGAAGGGTCTTCCTGTATGTTGTTATGACTGCCTGCCATGTGGAGATGGAGAAATTTCTAATAGAACAGATGCAACTGAGTGCCTGGTGTGTCCATATGAGTTTTGGTCCAATAAAGAAAAGGATTATTGTGTGCCCAAACAAGTTGAGTTTTTATCCTATGAGGATCCCCTGGGCATCTCTCTGACCACTGCTTCCTTGCTAGGCATCTGCTTTTGTGCTCTTGTGATGGTCATCTTCTCTCATCATCATAACACTCCCATAGTACGTGCCAACAATTCAGAGCTTAGCTTCCTGCTGCTGGTGTCACTCAAATTGTGTTTTCTGTGTGTGCTGCTGTTCATTGGCCAGCCACAGTTATGGACATGTCAGTTAAGATATGCTGTCTTTGGCATAAGTTTTGTCCTGAGTGTCTGCAGCATTTTGGTTAAGACTATGGTGGTAATAGCTGTGTTCAAGTCATCTCGTCCAGAAGGCAAGGATGCAATGAAATGGTTTGGACTACTTCAACAAAGATGTACTATTCTAGTCCTAACAACCATTCAAGTTGTTATATGCACAGTATGGATATCAAATGCATCGCCAACACCCCATAAAAACCACCAGTACATCCGTTCTAAAATAGTATTTGAATGTGCCATTGGCTCAGTGGCTGGTTTTTCTTTGCTGCTGGGATACATTGGACTATTGGCAGCAATAAGCTTTCTGTTAGCCTTTCTAGCCAGAAATCTTCCGGATAATTTTAATGAAGCAAAGTTTATCACTTTTAGCATGTTGATCTTTTGTGCTGTTTGGATTGCATTTGTTCCAGCATATGTGAGCTCTCCAGGGAAATATGCAGTGGCTGTAGAAATTTTTGCTATATTAGCTTCGAGTTTTGGTTTACTGATGGCCATATTTACCCCAAAGTGCTACATTATTCTTTTACATCCAGAGAGAAACACCAAAAAAGCCATCATGGGAAGA

>OlfCq15p

GGGGCACACATTGCCATCAGAAGAGGAGAGATCCTGGGATTACAAGAGTTTCTGCTATATAGTCATGCTAGCATCAATCCAAAAAATAATATGCTGAAGATCTTCTGAGAGAACATGTTCAGGTGCAGTTTTGAAACTAGGGGTAAAGACACAATTGGAGAGCAGGAGAAAAGTATCTGTACAGGACATGAGAATCTGAGTACCACAAAAACACCATACACGGATGTTTTAGGATTAAGAGCATCTTACAACGTCTATAAGACTGTTTATGCACTGGCGCATGCACTCCATGACCTAATGGAGTTTGAAGAGGGTAGAGGACCATCT

>OlfCq16

ATGTGGACCACTCTGTATATTTTTCTGTTGTTTCGCTGTTTCTCTTCAGAATCAGCCCTTCGATCAGGTTCCTGTCAGCTCCAGGGACGATTTAAGTTGGAAGGAATGTACCAGGATGGAGATTTTATACTTGGAGGTCTGTTTCATGTTCACTTTTTCACAGTTTTCCCAGAGCTGAGTTTTCAAACAGAGCCAGAACCACCATACTGTGAAAAATTTAATATAGAAATCTTCCAGCAGGCACAGACAATGGCTTTTGCAGTAGATGAGATCAACAGGAATCCAAATCTGCTGCCAAACATCACTCTTGGTTACCACCTTTATGACAATTGTGTCAGACTAGGAATAGCATTCCGGGCAGCCATATCCCTGGCTAGTGGGACAGAAGAGTCCTTCTCCAATTTAAACTGCACTGGCCCTCCTCCAGTGATTGGAATCGTGGGAGATCCAAGTTCAACTCCCTCCATTGCAATTTCCAATATTCTTGGGCTGTTCCGGGTACCTATTGTTAGTCACTATGCCACTTGCTCTTGTTTGAGTGACAGGAAAAAGTATCCATCTTTCTTCAGAACAATTCCCAGTGATGCCTTTCAGGTGCGAGCTATGATTCAGATTTTGAGATATTTTGGATGGACTTGGGTTGGTCTTATTTACAGTGATGATGACTACGGCATCTACGCTGCTCAGTCCTTCCAGCAGGAAATGTTGCTGTTTGGATATTGTGTTGCTTTTTCTGCAATTCTTCCCCATGACAACAACCACAGAGATATTCAACGTATAACAGCCATCATTCAGGCCTCTACAGCTAGAGTGGTGGTTGTTTTTTCCACTTCATCCTTTTTAATACCTTTGATGGAGGAGGTGGTGGTACAGAACATGACAGGTAGGCAGTGGATTGCAAGTGAAGCTTGGACCACCTCTCCAGTATACCACACTCCACGTTTTCTGCCAATCCTAGGGGGCACACTGGGCATAGCTATCAGGCGAGGAGAAATCGAGGGCCTTCATGACTTTCTGTTACGTCTCATTCCCAGTAATGATAAAAAAAATAGTATAATAAGAATTTTTTGGGAAAATATTTTTGGGTGTAGTTTTGAAAAGTGGGGTACAGAGACATTTGGAGAGCAAGTAAAAAATATTTGTACAGGACAGGAGGATCTGAGCACCACAGACACACCATATACTGATGTGTCAGGGCTAAGAGCAGCTTATAATGTCTATAAAGCAGTTTATGCCCTGGCACATGGACTTCATGATCTCATGCAGTGTGAGGAGGGTAGAGGACCATTCAATGGGAACAGTTGTGCTGAAACAACAAATCTAAAACCCTGGCAGCTGGTTCACTACCTCCAAAATGTAAACTTTACCACAGGCTTTGGTGATCAAGTGTCATTTGATAAAAATGGAGATGCTTTGCCCATCTACGATGTGCTGAACTGGCATCCAAGCACTGATGGATCAATAAAACTACATACAGTTGGGTTGGTAAACAAAGGAGCAGCAATGGAGATGGTGCTTACATTGGATGATGATGCAATATACTGGAATTTTGAGACAAGAAAACCTCCACAGTCTGTGTGCAGTGAGAGCTGCCCTCCAGGAACCAGGCAAGCAAGGAGGAAGGGTCTTCCGGTATGTTGTTTTGACTGCTTGCCATGTGGTGAAGGAGAGATTTCTAATGCAACAGGTAAGAAGATTATTTTTGACAAAATTAAACAGAATGTTTTAATACTGCCTCTCTTTACAGCATTTGTGTGTTTCTTTTTCAAAGGTGCTATTGAGTGCACAGTATGTCCAGATGAGTTCTGGTCCAATCCAGAGAAGGATCAGTGTGTACCAAAAGAAGTAGAGTTTCTGTCCTATGAGGATCCTCTGGGCATCTCTTTGACCACTGCTTCTCTGCTTGGCACCTGCTTCTGTGCTCTTGTGATGATAATCTTTGCTTTGCATCGTAACACTCCTATTGTACGTGCCAACAATTCAGAGCTCAGCTTCCTGCTTCTGTTGTCACTAAAAATGTGCTTCCTTTGTGTGCTACTGTTTATTGGTAGACCACAGTTATGGACATGTCAGTTAAGACATGCTGTGTTTGGCATAAGCTTTGTCCTGTGCATCTCCAGCATTCTGGTCAAGACTATGGTGGTAATAGCTGTGTTTAAGGCATCTCGACCAGAAGGCAAAGGAGCAATGAAATGGTTTGGAGCAACTCAACAAAGATGCACAGTTCTGGTCCTTACGGCCCTCCAGGTTGTTATATGTGTAGTCTGGCTATTAACTGCATCTCCAACACCCCATAAAAACAACCAATATATTCGCTCAAAAATAGTATATGAATGTGCCATTGGCTCAGTGGCTGGTTTTTCTCTGCTGCTGGGATACATTGGACTGTTGGCAGCAATAAGCTTCCTGTTAGCCTTCTTGGCAAGAAATCTTCCAGATAATTTTAATGAAGCAAAGTTTATCACTTTCAGCATGTTGATATTCTGTGCTGTTTGGATTGCATTTGTTCCAGCCTATGTGAGCTCTCCAGGAAAATATGCAGTTGCTGTAGAAATTTTTGCTATCCTGGCTTCCAGTTTTGGATTATTAGTGGCCATATTTGCACCAAAGTGCTACATCATTCTTTTACATCCAGAGAGAAACACTAGAAATGCCATAATGGGAAGAGAAATGCAAAATAAA

>OlfCq17

ATGTGTATCACTCTGTATCTTTCTCTATGCTTGTGCTTTAAACATATCTATGCAGATTCAATCCATAGACTACGTTCCTGTCAGCTCCAGGGACGTTTTAAACTGAATGGAATGTACCAGGATGGAGATTTTATACTTGGAGGCCTGTTTCATGTTCACTTTTTCACAGTGTTCCCAGAGCTGAGCTTCAGAATGGAACCACAACAACCATATTGTGAAAAATTTAATATGGAAGGTTTACAGCATGCACAAACCATGGCATTTGCAATAAATGAGATCAATAAGAATCCAACCCTGCTGCCAAACATTACTCTTGGATACCATCTTTATGACAACTGTGTGATGCTAAGAATGGCGTTCCGGGCTGCCATGTCACTGGCCAGTGGAACAGAGGACTCATATTCTAACCTCAATTGTACTGGCCCTCCTCCGGTGATTGGGATTGTGGGAGATGCAAGTTCAACTCCTTCCATTGCAATTTCCAGTGTTCTGGGGCTGTTTCGAGTACCTATAGTTAGTCACTATGCCACCTGCTCCTGTTTGAGTGACCGGAAAAAGTACCCTTCTTTCTTCAGAACGATTCCTAGTGATGCCTTTCAGGTGCGGGCTATGGTTCAGATTTTAAAGCATTTTAAATGGACTTGGGTTGGTCTCCTCTACAGTGGTGATGATTATGGTGTCTATGCTGCACAAATGTTCCACAAGGAAATGCAGTTGTCTGGACACTGTGTTGCTTTTTCAGAAATCCTGCCCAATGATCACAATCCCAAAGATATTCAGCGTATAATCAGGGTGATCCAGGGCTCTACAGCAAGAGTGGTTGTTGTTTTTGCCCTTTCATCCTTTCTGATACCTTTAATGGATGAGGTGGTGCTGCAGAACATGACAGGTTGTCAGTGGATTGCAAGTGAAGCTTGGGCCACCTCTCTTGAATACCACACTCCTCGTTTCTTGCCCTTCTTGGGAGGAACACTTGGCATTGCTATTAGGCGTGGAGAGATTGAAGGGCTTCATGAATTCCTACTACGTATTCGTCCCAGTAATGATACATCACAAAATATAGTGAGGATCTTCTGGGAAAATCTTTTTGGATGCAGGTTTGAAACTGGAGGTCTAAAAACAGGTCGAGAGCAAGAAAAAATGGAGTGTACAGGACAAGAGGATCTAAGTACAACAAACACACCTTACACTGATGTTTCAGGGTTAAGGGCTTCTTATAATGTCTATAAAGCAGTTTATGCTCTGGCACATGGACTTCATGACCTGATGCAGTGTGAGGAGGGAAGAGGACCATTCATTGGGAACAGCTGTGCTGACATAACTAGCCTTAAACCCTGGCAGTTGGTTCACTATCTACAAAATGTGAACTTCACCACAGGCTTTGGGGATCATGTGACATTTGATAAGAATGGAGATGCTCTAGCCATCTATGATGTGTTGAATTGGCAGCCAAGCTCTGATGGGTCAATAACAGTACACAAGATTGGTGTAGTATATGAAGGGGCAACAACAGGGATGATGCTCACACTGGATGCAGATGCAATATATTGGAACTTTGAAACCAAAAAACCCCCACTGTCTGTATGTAGTGAGAGCTGCCCCCCAGGCACCAGACAAGCAACAAGGAAGGGACTTCCTCTCTGCTGTTTTGACTGCCTGCCTTGTGGAGATGGAGAGTTTTCTAATACAAGAGATGCTGTGGAGTGCATGATGTGTCCAGATGAGTTTTGGTCCAGTCCGGATAAGGATCAATGTGTCCCAAAAGAAGTAGAGTTTCTGTCCTATGAGGATCCTCTGGGCATCTCTCTGACCACTGCTTCTCTGCTTGGCACCTGCTTCTGTGCTCTTGTGATGATCATATTTGCTCTTCATCATAACACACCCATAGTACGAGCCAACAATTCAGAGCTCAGCTTCCTGCTGCTTTTGTCACTCAAACTGTGTTTTTTATGTGTGCTGCTGTTCATCGGCCGACCACAGTTGTGGACGTGTCAGTTAAGACATGCTGTGTTTGGCATAAGTTTTGTTCTGTGTGTCTCCAGCATTCTGGTCAAGACTATGGTGGTAATAGCTGTGTTCAAGTCCTCTCGGCCAGAAGGCAAAGGAGCAATGAAATGGTTTGGAGCATCTCAACAAAGATGCACAGTTCTGGTCCTTACGGCCCTCCAGGTTGTTATATGTGCAGTCTGGTTATCAACAGCCTCTCCAACACCCCATAAAAACAATCATTACTTCCGCTCTATAATAGTATATGAATGTGCCATTGGTTCAGTGGCTGGTTTTTCTCTGCTGTTGGGATACATTGGACTGTTGGCAGCAATAAGTTTCCTGTTAGCCTTCTTGGCAAGAAATCTCCCAGATAATTTCAATGAAGCAAAGTTCATCACTTTTAGCATGTTAATCTTCTGTGCTGTGTGGATTGCATTTGTTCCAGCCTATGTGAGTTCGCCAGGAAAATATGCAGTGGCTGTTGAGATATTTGCCATATTAGCTTCTAGTTTTGCATTATTATTGGCCATATTTGCCCCAAAGTGCTACATCATCCTGTTACATCCAGAGAGAAACACAAAAAATGCAATAATGGGAAGAGAAACACAAAACAAA

>OlfCq18

ATGTGGTTTACTCTGTATATTTACATTTGCCTGTCTTTGAATTGTATCTCTGCAACTTTAATCCTCAAACCAAGTATGTGTTTGCTACAAAGGAGTTTTAAGTTGAATGGAATGTACCAGGATGGAGATTTTATACTCGGAGGCCTATTTGAGGTTCACTTCTTTACAGTGTTTCCAGATCTGACTTTCACAACAAATCCAAAATCACCATACTGTGAAATATTTAATATGGAAGGCTTCCAGCATGCACAGACCATGGCTTTTGCAATAGAAGAGATAAATAAGAATCCAAAACTGCTGCCAAACATCACTCTTGGATATCATCTTTATGACAATTGTGTGATGCTAGGAATGGCATTCCGGGCTGCCATTTCACTTACTAGTGGGACAGAGGAGTCCTTCTTGAACCTAAATTGCAGTGGTCCATCACCAGTGATTGGGATTGTTGGAGATCCAAGTTCAACTCCTTCCATTGCAATTTCCAGTGTTCTGGGGCTGTTTCGAGTGCCTATAGTTAGTCACTACGCCACCTGCTCCTGTTTGAGTAACAGGAAAAAGTACCCCTCTTTCTTTAGAACAATCCCCAGTGATGCATTTCAGGTACGGGCTATAGTCCAAGTCTTAAAACATTTTAAATGGACCTGGGTTGGTCTCCTCTACAGTGATGATGACTATGGCGTCTATGCTGCTCAGTCTTTTCAGCAAGAAATGCGGCAATTTGGACTTTGTGCTGCATTTTCTGAATTCTTGCCTCATGATAATAATCCCAGAACCATACAGCATATAATGGGGGTTATTCAGGGCTCTACAGCAAAAGCGGTGGTTGTTTTTGCCCCATCATCCTTTCTCATACCTTTGATGAATGAGGTGGTTTTGCAGAATATGACAGGCAGGCAGTGGATTGCAAGTGAAGCTTGGGCCACCTCTCTTGAATCCCATATTCCAAGTTTCCAGCCCTTTTTGAGGGGCACAATAGGAATTGCTATCAGGCGTGGAGAGATTCAAAGACTTCATGACTTTCTACTACGTATTCGCCCTAGTAATGATCCAAAAAATTATATGTTGAGAATCTTCTGGGAGAATATGTTTGGGTGCAGTTTTGGTAAAGGAGATACTGAAGGAGAGCAAGTCATAAAGGTATGTACAGGACAGGAGGATCTGAGCACCACAAATACACCATACACTGACGTCTCAGGACTAAGAGCAGCTTATAATGTATATAAGGCAGTTTATGCACTGGCACATGCCCTTCATGACTTGATGGAGTGTGAGGAGGGGAAAGGACCATTCAGTAAAAACAGCTGTGCTGACAAAACAAACCTAAAACCCTGGCAGGTGGTTCACTACCTACAGAATGTAAACTTCACTACGGGCTTTGGGGATTATGTGTCATTTGATAAGAATGGAGATGCTTTGGCTATTTATGATGTACTAAACTGGCAGCCAAGCTCCGATGAATCAATAAGGATCTACACGGTTGGTGTTGTAAAAGAAGGAACAGAAACTGGAATGGTACTCACACTGGATGAAGATGCAATATTCTGGAATTTTGAGACGAGAAAACCCCCACGGTCTGTGTGCAGTGAAAGCTGCCCTCCAGGCACCAGACGAGCCACTCGCAAGGGTCTTCCTGTCTGCTGTTTTGACTGCCTGCCATGTGGAGATGGAGAGATTTCTAACACAACAAATGCTGTTGAGTGCTTTCTATGTCCAGATGAGTTTTGGTCCAATCAATATAATAATCACTGTGTACCAAAAGAAGTAGAGTTTCTCTCCTATGAAGATCCTCTGGGGATCTCCCTGACCACTGCTTCTCTGCTTGGCACCTGCTTCTGTGCTCTTGTGATATTTGTTTTTGCTCTTCATCGTAATACTCCTATAGTACGTGCAAATAATTCAGAGCTCAGCTTCCTACTGCTGCTGTCACTCAAACTGTGTTTCCTTTGTGTCCTGCTGTTTATTGGTCGACCACAGCTGTGGACATGTCAGTTAAGACATGTTGTGTTTGGTATAAGCTTTGTCCTGTGCATGTCCAGTATTCTGGTCAAGACTATGGTGGTAATAGCTGTGTTCAAGTCCTCTCGGCCAGAAGGCAAAGGAGCAATAAAATGGTTTGGAGCAGTTCAACAGAGAAGCACAGTTGTTGTCCTCACAGTCCTCCAGGTTCTTATATGTGCAGTCTGGTTATCAACTGCCTCTCCAACACCCTATAAAAACAACCAATATATCCGTTCTAAAATAGTATATGAATGTGCTATAGGTTCAGTTGTTGGGTTTTCAATGTTACTCAGCTATATTGGCTTTTTGGCAGCAGCCAGCTTTTTGTTAGCCTTCCTGGCTAGAAATCTTCCAGATAATTTTAATGAAGCAAAGTTCATCACATTTAGCATGTTGATTTTTAGTGCCGTGTGGATTGCATTTGTTCCAGCGTATGTGAGTTCACCAGGTAAATACACAGTGGCTGTAGAAATTTTCGCCATTCTAGCTTCGACTTTTGGTTTACTGATTGCCATATTTGCCCCAAAGTGCTACATCATTCTTTTACATCCAGAGAGAAACACCAAAAATGTCATAATGGGAAGAGAAACACAAAATAAA

>OlfCq19

ATGTGGGTCATTCTGAACATCTCCATCTATCTGATTTTTAATTTTATTATGTCAGCTTCAATACTCGGATCAAACACCTGTCAGCTCCAGGGACACTTCAGATTGAATGAAATGTACCATGATGGAGATGTTATACTTGGCGGCCTGTTTGAGGTTGACTTCCTCACAGTGTTCCCAGATCTGGACTTCAAAACAGAGCCAGAACCACCATACTGTGTGCAATTTGACATGGAAAGCTTCCAGCAGGCTCAAACCATGGCTTTTGCAATTGATGAAATCAATAAAAATCCAAATCTTCTGCCAAACATCACTCTTGGTTACCATCTATTTGACAACTGTGTGATGCTAGGAATGGCATTTCGGGCTGCCATATCCATGGCTAGTGGAACAGAAGAGTTCTTCAATAACATCAACTGCACTGGCCCACCCCCAGTGATTGGAATCGTAGGGGATTCAAGTTCAACTGTTTCCATTGCAATTTCAAGTATTCTAGGGTTGTTTCGAGTACCTATAGTTAGTCACTATGCCACCTGCTCCTGTTTGAGTGACAGAAAAAAGTACCCCTCTTTCTTCAGAACGATTCCCAGTGATGCCTTCCAGGTCCGTGCTATGGTTCAGATCTTGAAATATTTTAGATGGACTTGGGTTGGTCTCATTTACAGTGATGATGACTATGGAGTTTATGCGGCTCAGTCCTTCCAGCAAGAAATGCAGCTGTTTGGAGGTTGTGTTGCTTTTTCTGAAGTTTTACCCCATGATAACAACCTCGAAGATATTAAGCACATAACCAAAATGATTCAGGCCTCTACAGCTAGAGTGATAGTTGTTTTTTCCACTCCTTCCTTTTTGATACCTTTGATTGATGAGTTGGTGTTGCAGAACATGACAGACAGGCAGTGGATTGCAAGTGAAGCTTGGGCTACCTCTTTTGTACATCACAGTCCACGTTTACTGCCCTTTCTGAAGGGCACACTTGGCATTGCTATTAGGCGTGGGGAGATTCAGGGACTTTATGAATTTCTGCTACGTCTCCAACCTACAAATGATCCAAAAAACAATATGATCAGAATATTCTGGGAAATCATGTTTGGGTGCAATTTTGAAACTGGGGGTAAAAAGAATAGTGGAGATCAAGTCATAAATATATGTACAGGACAGGAGGATCTGAGAACCACAAAAACACCATACACAGAGGTTTCAGAGCTACGAGCGTCTTACAATGTCTATAAGGCAGTTTATGCCCTGGCACATGCGCTTAATGACTTGATGCAGTGTGAGGACGGAAGAGGACCATTCAGTGTAAACAGCTGTGCTGACATAACAAATTTAAAACCCTGGCAGCTGGTTCATTACCTACAGAATGTCAACTTTACCACAGACTTTGGGGATCATGTGTCATTTGACAAGAATGGAGATGCTTTGGCCATCTATGATGTCCTGAACTGGCAGCCGAGCTCTGACGGCTCAATAAGGGCCCAAACAGTTGGTGTAGTAAATGAAGAGGTTGCAACAGGGATGGTGCTTACACTGGATGAGGATGCATTATATTGGAACTTTGAGTCAAAAAAACCCCCAAGTTCTGTGTGCAGTGAGAGCTGTGCACCAGGCACTAGGCTTGCCACGATTAAGGGCTTGCCGGTTTGCTGTTTTGACTGCCTCCCATGTGGAGATGGTGAGATTTCAAATATAACAGGTGCAGTTGAATGCACAATGTGTCCAGATGAGTTTTGGTCTAATTTAGATAAGGACCAATGCATTCCCAAAGAAGTAGAGTTTCTGTCCTATGAGGATCCTCTGGGCATCTCTCTGACCACTGCTTCCCTCCTTGGCACCTGCTTCTGTGTTCTTGTGATGTTTGTATTTGCTGTTCATCGTAATACGCCTATAGTAAGGGCCAACAATTCAGAGCTCAGTTTCCTGCTGCTTTTGTCACTAAAAATGTGTTTCCTGTGTGCGCTGCTGTTTATTGGCCAGCCCCAGTTATGGACGTGTCAGTTAAGACATGTTGTGTTTGGCATAAGCTTTGTCCTATGCATTTCCAGCATTCTGGTCAAGACTATGGTGGTAATAGCTGTATTCAAGTCATCTCGACCAGAGAGCAAAGGTGCTATGAAATGGTTTGGAGCAGTTCAACAAAGATGCACAGTTCTGGTATTGACAGCAATACAGGTTGTGATATGTGCAGTCTGGCTATCAACTGCCTCACCCGCACCCCATAAAAACAACCAATATATCCGCTCTAAAATAGTTTACGAATGTGCCATTGGCTCAGTGGCTGGTTTTTCAATGTTATTAGCATACATTGGACTTTTGGCAGCAGTAAGCTTTCTTTTAGCCTTCCTAGCAAGAAATCTTCCAGATAATTTTAATGAAGCTAAGTTTATTACTTTTAGCATGTTGATCTTCTGTGCTGTATGGATTGCATTTGTTCCAGCATATGTGAGCTCTCCAGGAAAATACGCAGTGGCTGTGGAAATATTTGCCATTTTAGCTTCCAGTTTTGGATTACTGCTGGCCATATTTGCCCCTAAGTGCTACATCATCCTTTTACATCCAGAGAGAAACACTAAAAAGGCAATTATGGGAAAAGAAGCACAAAATACA

>OlfCq20

ATGTGGATTACTTTGTGTATTTATCTATACCTGTCCTTGAACTATATTTGTGCACATTCAATCCTCAGGACATCAGATTCATGCCGGCTCCAGAGACGTTTTCACCTGAATGGCATGTACAAGGATGGAGATGTGATACTTGGAGGCTTGTTTCAGGTTCATTTCTTTACAGTGTTTCCAGAACAGAGCTTCAGAACTGAACCAGAGCCACCATTCTGTGAAAAATTTGATATGGAAAGCTTCCAACAAGCACAGACCATGGCTTTTGCAATAGAGGAGATCAACAAGAATCCAAACTTGCTGCCAAATATCACTCTTGGTTTCCATCTTTATGACAACTGTGTGAGATTAGGAATGGCATTCCGGGCTGCCATATCTCTGGCTAGTGGGACAGAGGAGTCCTTCCAAAACTTAAACTGCACTGGGCCTCCACCAGTGATTGGGATTGTTGGGGATCCAAGTTCAACTCCTTCTATAGCGATTTCCAGTCTTCTGGGGCTGTTTCGAGTGCCTATTGTTAGCTACTACGCCACTTGCTCCTGTTTGAGTGACAGAAAAAAGTACCCGTCCTTCTTCAGAACAATTCCTAGTGATGCCTTCCAAGTCCGGGCTATGGTTCAGATATTGAGACATTTTGGATGGACCTGGGTCGGTCTTATTTACAGTGATGATGACTATGGTGTCTATGCTGCCCAATCTTTCCATCTGGAAATGCAGATGTTTGGACATTGTGTTGCTTTCTCTGAAATTCTACCACATAATAATAACCAAAGAGATATTCAGCGCATAATGGGGGTGATTCAGGCATCCACAGCTAGAGTGGTGGTTGTTTTTTCAACTTCATCCTTTCTGTTGCCTTTGATAGATGAGGTGGCATCTCAGAACATGACAGGCAGGCAGTGGATTGCAAGTGAAGCTTGGGCTACTTCACCTGTATACCATTCTCCTCGTTTACTGCCCTTTCTGGGGGGCACATTGGGTATTGCTGTTAGACATGGAGAGATTCCAGGGCTTCATGATTTTCTGTTACATCTCGGCCCCGGCAATGAGTCAAGAAATAATATGTTGAGAATCTTCTGGGAGAACATGTTTGGATGCAGTTTTGAACCTGGAGTTAAAGACACAAACAGTGAGATAAAATTGTGCACAGGCCAGGAGGATCTAAGCACCACAAACACACCATACACTGATGTTTCAGGACTGAGGGCAGCTTATAATGTCTATAAGGCAGTTTATGCCCTGGCACATGGACTGCATGACCTGATGCAGTGTGAAAAGGGGAGAGGACCATTCAGGGGGGAAAGCTGTGCTGACATAACAAATCTAAAACCCTGGCAGCTAGTTCACTACTTACAGAAAGTGAACTTCACCACAGGCTTTGGGGATCATGTGTCATTTGATGAGAATGGAGATGCTCTGGCTATTTACGATGTGCTGAACTGGCAGCCAAGCTCTGATGGATCAATCCAAATCTTCACTGTTGGTGTTGTAAAGGAAAGGACAGAAACAGGAATGGTGCTCACAATAGATGAGGATGCAATATACTGGAACTTTGAGACAAAAAAATCCCCACAGTCTGTGTGCAGTGAGAGCTGCTCTCCAGGCACCAGACGGGCCACACGCAAGGGGCTTCCAGTCTGCTGTTTTGACTGTCTGCCATGCAGAGATGGAGAGATTTCCAATACAACAGATGCTATTGAGTGTATGCTGTGTCCAGATGAGTTCTGGTCCAGTCCAGATAAGGATCAATGTGTCCCAAAGGAAGTAGAGTTTCTGTCCTATGAGGATCCCCTGGGCATCTCTCTGACCACTGCTTCCCTGCTTGGCTCCTGCTTCTGTGTTCTTGTGATGATCATCTTTGGTCTTCATCGTAACACACCCATAGTACGAGCCAACAATTCTGAGCTCAGCTTCATGCTGCTTTTGTCCCTAAAAATGTGTTTCCTGTGTGTTCTGCTGTTTATTGGTCGGCCCCAGTTATGGACATGTCAATTAAGACATGCTGTGTTTGGCATAAGCTTTGTCCTGTGCATCTCCAGCATTCTGGTCAAAACTATGGTGGTAATAGCTGTGTTCAAGTCCTCTCGACCTGAGGGCAAAGGAGCAATGAAATGGTTTGGAGCAGCTCAACAAAGATGTACAGTTCTGGTCCTCACAGTCCTCCAGGTTGTCATATGTTCAGTTTGGCTATCAACTGCCTCTCCAACACCTTATAAAAACAACCAGTACATTCGTTCTAAAATAGTATATGAATGTGCCATTGGCTCAGTTGCTGGTTTCTCTCTATTGTTGGGATACATTGGACTGTTGGCAGCAGTAAGTTTTCTTTTAGCCTTCTTGGCAAGAAACCTTCCAGATAACTTTAATGAAGCAAAGTTCATCACTTTTAGCATGTTGATCTTCTGTGCTGTTTGGATTGCATTTGTTCCAGCATATGTGAGCTCTCCGGGAAAATATGCAGTGGCCGTTGAGATATTTGCTATTTTAGCTTCTAGTTTTGGATTATTGGTGGCCATATTTGCCCCAAAGTGCTACATTATCCTTTTGCATCCAGAGAGAAACACAAAAAATGCTATAATGGGAAGAGAAACACAAAAGAGA

>OlfCq21

ATGTGGATCAGTCTAAATATCTTTCTTTATCTGTCTTTAAATTGTATCTTTGCAGCATCAGTTGTCAATCCAGGTACCTGTCAGCTCCAGGGACACTTCAAGTTAAATGGAATGTACCAGGATGGAGATTTTATACTTGGAGGCCTGTTTGAGGTTCATTTTTTTACATTGTTCCCTAAGCTTAGCTTCAAATCAGAGCCAGAACCACCATATTGTGAAAAATTTGATATGGAAAGCTTTCAGCAGGCACAAACTATGGTTTTTGCTATAGATGAGATCAATAAAAATCCAAATCTGCTGCCAAACATCACTCTTGGCTACCATCTTTATGACAACTGTGTGATGCTAGGAATGGCATTTCGAGCTGCCATATCTCTGGCTAGTGGGACAGAGGAGTACTTCTCCAACCTAAACTGCACTGGTCCTCCTCCAGTAATTGGGGTTGTCGGTGATCCTGGATCAACTCCATCTATTGCAATTTCCAGTGTTCTGGGGCTGTTTCGAGTACCTATAGTTAGTCACTACGCCACCTGCTCCTGTTTGAGTGACAGGAAAAAATATCCCTCTTTTTTCAGAACAATCCCTAGTGATGCCTTTCAGGTCCGAGCTATGGTTCGAATCTTGAGCCATTTTGGATGGACCTGGGTTGGTCTCATCTACAGTGATGACGATTATGGTATCTATGCATCTGTGTCTTTTCAGCAGGAAATGCAGCAGTTTGGAAGCTGCATTTCTTTTTCTGAAATCCTTCCCCATGATAATAACCCCAAAGATATACAGCGTATAATGGAAGTGATTAAGGCCTCTACAGCTAGTGTGGTGGTTGTTTTTTCAACATCATCCTATCTGATGCCTTTGATTGATGAGGTGGTGTTGCAAAACTTGACAGGCAGGCAATGGATTGCAAGTGAAGCTTGGGCCACTTCACCAGTATTTCACACTCCACGTTACTTGCCTTTCCTGGGGGGCACACTAGGCATCGCCATCAGGCGTGGAGAGATCCTGGGATTACAAGAGTTTCTGCTACATATTCATGCTAGCAACAATCCAAAAGATAATATGCTGAAGATCTTCTGGGAGAACATGTTCAGGTGCAGTTTTGAAACAAGAAGTAAAGACACAATTGGAGAGCAGGAGAAAAGTATCTGTACAGGACATGAGGATCTGAGTACCACAAAAACTCCATACACTGATGTTTCAGGATTAAGAGCATCTTACAATGTCTATAAGGCTGTTTATGCTCTGGCACATGCCCTTCATGACTTAATGGAGTGTGAGGAGGGTAGAGGACCATTTGATGGAAACAGCTGTGGCAACATAACAAATCTGAAACCCTGGCAGCTGGTCCACTACTTACAAAAAGTGAACTTTAAAACTGGTTTTGGAGATCATGTGTCATTTGATGAGAATGGAGATGCTCTGGCAATCTATGATGTGCTAAACTGGCACCCAAGCTCGGATGGATCAATAAGATTACACACGGTTGGTGTAGTCAATGAAGGGGCAGGAACAGGGAAAGTGCTCTCACTGGATGAGAGTGCATTATACTGGAACTTTCAGACAAAAAATCCCCCACGGTCTGTGTGTAGTGCGAGCTGCCCACCAGGCACCAGACAAGCGATGAGAAAGGGTCTTCCTATCTGCTGCTTTGACTGCTTGCCATGTGGCGATGGAGAGATTTCTAATGCAACAGATTCCACTGAATGCGTGACATGTCCAGATGAATTCTGGTCCAATTTTGATAAAAATCAATGCGTCCCTAAGGAAGTAGAGTTTCTGTCCTATGAAGATCCTCTGGGCATCTCTTTGACCACTGCTTCCCTGCTTGGCACATGCTTCTGTGCTCTTGTGATGATCATCTTTGCTCTACATCTTAACACGCCCATAGTACGAGCCAACAATTCGGAGCTCAGCTTCCTGCTGCTTTTGTCACTCAAACTGTGTTTCCTGTGTGTTCTTCTATTCATTGGCCAGCCACAGTTGTGGACTTGTCAGTTAAGACATGTTGTGTTTGGCATAAGCTTTGTCCTGTGCATCTCCAGCATTCTGGTCAAGACCATGGTGGTAATAGCTGTGTTCAAGTCATCTCGGCCTGAGGGTAAAGGAACAATGAAATGGTTTGGAGCAGCTCAACAAAGATGCACAGTTTTGGTCCTCACGGCTCTCCAGGTTGTGATATGTGCAGTCTGGTTATCAAATGCATCTCCAACACCCATCAAAAACAACCAGTATATCCGTTCTAAAATAGTCTATGAATGTGCTATTGGTTCAGTGGCTGGTTTTTCTCTGCTGCTGGGATACATTGGACTATTGGCGGCAATAAGCTTCCTGTTAGCTTTCCTGGCAAGAAATCTTCCAGATAATTTTAATGAAGCAAAGTTCATTACTTTTAGCATGTTGATCTTCTGTGCTGTATGGATTGCATTTGTTCCAGCATATGTTAGTTCACCAGGTAAATATGCAGTGGCTGTTGAGATATTTGCTATTTTAGCTTCTAGTTTTGGCTTACTGGTGGCCATATTTGCTCCAAAATGCTACATAATCCTTTTACATCCAGAGAGAAACACTAAAAAAGCCATAATAAGAAGAGAAACAAAAAAGAGA

>OlfCr1

ATGTCTATACATGTCCTGTCATGGCTCACACTTTCTGTGCCGGTGCTTCTGTTGTGGCCAGTATGTGGATCTGGAACTGAGGTGACTCCGCTGGAAACATGTGTGTACTTGATCACGCCACAGTACTCAGATGAACTTGGTACCTATCAAGACGGTGACGTGATTATTGGTGGTCTCATCAATTTACACAACCTGGCTGCAACTCCAGACCTGAGCTTCACCAGAAAACCTGGATTAGCACAATGCTTAGAGTTTCAGGAACGTACTTACCGCTGGTTTCAGGCAATTGTTTTTACAGTTGAAGAAATTAACCAACATCCCTCACTGTTGCCGGGGGTCAAACTGGGTTATCACATAATGGATAGTTGTTCCCGTTACCCACACAGCCTGACAGCAGCCATGTCAATGATCAGTGGAGGAAACAAAACTTGCGGTCCCACTAAACCAGCCAAGCTTCTGATCGGAGATTCTTCATCTACTCAAAGCATTCTTCTATCCACAACCTTGGTTCCCCTGAAGATTCCAATGATCAGTTACCTGGCTGGCTGCCCATGTCTCAGTGACAGACAAAAGTACCCAAATTTTTTTCGCACTATCCCCAGTGATTTTTATCAGGCACGCACCATGGTACAGATTGCCAAACGCTTTGGGTGGACCTGGATTGGAGCCGTGATTGCAGATAGTGACAATGGGCACGGGACTTTGCAGGCGCTTGAAGAAGAAATTAAAGGCACTGGGATCTGCTTGGCCTTTTATCATACACTCTACAGGGAGCGGTTACAAAAGGACGTGGCCTTTGCTGCCAGAACAGTCCAAGCCTCCTCTGCTCGAGTGATCCTGGTTTTGGCTTGGTATACAGATGTGGAGGCATTTCTGTTGGAATTGATGCGTAGGAATGTGACAGATAGGCTGTTCCTGGCCAGTGAGATCTGGAGCACCAGTTACAATATCATGGCTAATCCCCAGCTCTATACCATCTCTAAGGGAACTTTAGGTGTAGCTTTAAGAAGTGCACCCATACCTGGCTTCAGTGCACATCTTCAGCAGTTACATCCTTCCCGTTATCCTGAGGATAAATTCTTAAGGACCCTTTGGGAAAATACATTTGGTTGTAGTCCTACAATAAGCTCTTCCAAGATTATCCAGAATTCCTTGCCTCCATGCAGTGGCAGAGAGAGTCTAGAGGGTCACATTGAATTTATTGATACATCTCATCTGACAGTTTCATATAATATTTATTTGGCTGTTTATGCTGCTGCTCACGCACTTCATTCATTGCTGGAGTGTAACTCACAAAATCACACCGATTCAAAAATAAAACCAAAGTGCTCCTCTCCAGACAACATCACACCAGCGCAGCTTTTACAGCATGTTAAAGACGTTCAATACACCACTCAGTTAGGAGAGGAGTTCTATTTCCTGGAAGGTGGCATTCCACCAGTCTATGACTTGGTGAACTGGCAGATAGCTCCTGATGGTTCACTCCAATATGCTTTTATTGGACATGTGGATGGGAACCAGCTCAGTATTAATGACTCAGCTATCACATGGCCTGGAGATTCTGGCAAGGTTCCAATATCTGTGTGCACGTCTGAGTGTCCTCCAGGCACACGTAAAGCCATTAAGAAGGGTCTTCCAGTGTGCTGTTTTGACTGCCTGCCCTGCACAGAGGGGGAAATCAGCAACAGTACAGGCTCAATAAAATGTTATCGCTGTCCTAAAGAGTTCTGGTCAAATAGTTACAAAAATGAATGTGTGGCCCGTGAAACTGAGTTTCTCTCTGTTAAAGAAACGATGGGCATCACGCTGATGAGTGTCGCTGTTTCCGGTGCTGTCATGACAACTACAGTAGCTGTGATTTTCCTCTACCACCGGAACACTCCAATAGTAAAAGCCAACAACTCAGAGCTGAGCTTTCTGCTCCTGCTGTCTCTCAAACTATGCTTCCTCTGCGCACTGGTGTTTGTGGGCCAGCCTTCACTCTGGTCATGTAGGATTCAGCAGGCTGCATTTGGGATTAGTTTTGTCCTGTGTATCTCCTGTATTCTGGTCAAGACATTTGTTGTTCTAATTGCATTCCATTCTACTAGACCTGAATCTTCAGCTCTTATCAAATGGTTTGGACTGGGCAAGCAGAGAGGAATTGTGCTGGTGTTTACCTGCGTCCAGGTGGTTATCTGTGCCATTTGGCTCTGTGTCAGTCCACCTTTACCTTACCAAAACTTTGGAATGCACAGATCAAAGGTCATCCTTGAGTGCACAATTGGGTCTGTTGTGGGATTCACTTGTGTACTGGGTTACATTGGCTTTTTGGCGACTGTCTGCTTCCTACTGGCCTTCTTTGCCCGCAAACTTCCAGATAATTTTAATGAGGCAAAATATATTACTTTCAGCATGCTGATCTTCTGTGCTGTCTGGGTTGCATTTGTGCCAGCATATGTTAGCTCGCCAGGAAAGTACAGTGTAGTGGTGGAAGTTTTTGCCATCCTGGCCTCTAGTTTTGGACTTTTGTTTTGTATTTTTGTCCCAAAATGTTACATTATTTTACTTAAGCCAGAAAACAACACTAAAAAGTTTCTTATGGGAAAAGAA

>OlfCs1

ATGATGGGTTTTATTATAGGGTTATGGTTGGTGGGATTTATTGTTGGCATGGTCAGGGTTTGTAATGCAAACCCAACCTGTAGTCTAAAGAAAAGTTTTGTCTCGGAAAGCTTGTATAAAGACGGCGATGTGATTATTGGTGGTCTGTTTCCAGTTCATGTTCAATCCCCAGTGCCTGATCCTGCTTTTACTCAACAACAGCATGGTTCCAACTGCCAGGGTATTGACCTTCGATCATATCGCTGGCTCAGAGCTATGATCTTCACTGTGGATGAGATTAATAGGGACCCTGTCCTGCTACCAAATGTTACTCTGGGTTACCTAGTAGCTGATACTTGTTTAGCTGAGAGTACAACACTGAGTGCTGCTTTAGCTCTGGTAACAGGGCAAGAAACAACAGTGTTTAGCACCGAATGCAATGGAGCCCCAATGGTTCCTGTCATTATTGGTGATGCTCGTTCCTCTGCCTCAGTGGTGGTGGCAGACACACTGGGAGTGTTTGATATTCCTATGGTGAGTTATTTTGCATCATGTGCTTGTCTCAGTGACAAGCACAGATTTCACACTTTTCTACGCACTGTCCCTAGTGATGCCTTCCAGGCCAAGGCCATGGCCCGTCTGCTTCATCTACTAGACTGGAACTGGGTGGGGGTGGTTGCAGGGGATGATGAATATGGCAAAAGTGGCTCACACCTGCTTTTAAAAGAGCTCGAAGGTTCAGGGGTCTGTGTGGACTATGTTGAAGTCATTCCTAAATCCCATTCTCAGAGCCGGATCAAGCAGATTATGGAGAGGATCCAGAGTTCCACAGCTAGTGTGGTGGTGACATTTGCCATCGGGCCAGACCTTGAAGTTATTTTCAAAGAAATGGTGGTGCAGAATGTGACCAATAGGCAGTGGATAGCAACAGAGGCATGGAGCACTACTGTTCAGTACTCTGACCCAGAAAGCATTCCTCTCCTAGCTGGTACCATTGGCTTTGCTTTGCGCAGAGCTGAAATTAAGGGGCTTGGTGCTTTTCTCACCCAACTGAATCCTGTGAAGCAATCAAATGAACCATTTGTAAAAGATGTATGGGAAGAAATTTTTAGGTGCTCTTTAGCACATGACAGGCAGCCTTCATTTAAAAGACCAAAGTGCACAGGATCAGAGAGTGTGGAAAAACATGGCCGCATCTACACAGACGTTTCACAGCTGAGAGTCACTTACAATGTGTATAAGGCTGTGTATGCCATTGCCTATGCTCTTCACAACATGATTGCCTGCCTGCCGGGTAGGGGCCCTTTTGAAAATGGACAGTGTCCAGATGCAACTCAAGTAAAGCCAAGGCAGCTTCTCCACTACCTAAATGCAGTAAACTTCACAACACCAGTGGGAGAGCTGGTCTATTTTGAAAATAATGGTGAGCCGTCTGCATCTTATGACATAATGAATTGGTATGTCAATAAAAGTGGAGAAGTAAATTTTGTCCAGGTGGGACAGTATGATGCAGCCAAAGGACCAGGCCAGGAGCTGAACATAAACATTGAAAAAGTGATTTGGGGAGGGGGCTGGGGTAACCAGGTCCCTGTGTCTGTATGCAGTGTGAGCTGTCTTCCAGGCACTAGAAAGGTTGTACAAAAAAGAAAACCTATCTGCTGTTTTGACTGTATCCCTTGTGCAGCAGGTGAAATCAGCAATATGTCAGACTCTACGGAGTGTATGAGATGTCCTGAGAAGTTCTGGTCAAACACCGAAAAAACTAAGTGCATTCCAAAGGTTGTAGAATTTCTGTCCTTACAAGATACCATGGGAATTGTGCTGACAGTCTTATCTGTCACTGGGGCTACACTAACTACAACTGTTTTGGCAACTTTTTTCCATCATCGTGACACGCCACTTGTGCGAGCTTACAACTCAGAGCTGAGCTTCCTGTTGTTGATGTCACTCACACTTTGTTTCTTGTGTGCTCTAGTTTTTATTGGCCGGCCTGCAGCATGGAACTGCATGTTACGTCACACTTTATTTGGAGTGAGTTTTGTTACCTGTATTGGTTGTATCCTCAGTAAGACTGTGGTGGTGCTGGTAGCTTTTCGAGCTACTCGGCCTGGATCTCATTTGATGCAGTACTTTGGGCCCATCCAGCAGAGGGCAGCAATCTTCATTTGCACGCTGGTTCAGGTTGTCATATGTTTGCTGTGGCTACTGCTGGATCCCCCTCAGCCCACAGAGAGTGCAGGGGAGTTAGGTGCTCGATTGATCCTGCAGTGCACTGTGGGATCTGTTGTGGGCTTTGTATTTGTGCTGGGGTACATTGGTATGTTAGCAGTTGTCTGCTTCTTGTTGGCTTTCTTTGCTAGAAAGCTTCCAGACAATTTCAACGAAGCTAAATTCATTACTTTTAGCATGTTTATTTTCTGTGCTGTGTGGGCTGCATTTGTGCCAGCATATATCAGCTCCCCAGGAAAGTACACAGTGGCTGTAGAGATTTTTGCTATTTTGGCTTCCAGTTATGGCCTGTTAATTTGTATATTTACTCCAAAATGTTATATCATTCTGCTTAAACCTGAGAAAAACACAAAAAAGACGATGATGGCTAGA

>OlfCs2

ATGCAGTTGTGGATTGTGGGATTAATTGGTGAAATAATCAGGCTTTGTAGTACAGAGCAGTCTTGTGGTCTGCAAGGCAGAGTTATTTCAGAGAGTCTATATAAAGAAGGAGATGTGATAATTGGTGGTCTGTTTCCAGTTTATAATGAGGCACCAGTACCTAACCATGCTTTTACTCAAATACAGAATAGATCTCGCTGTCAGGGTGTTGACCTTCGTTCATACCGCTGGCTTAAAACCATGCTTTTCACAGTGGAGGAGATTAATCGAGACCCCTTTCTGCTTCCGAACATTATTTTGGGGTACCTGGTAGCTGACACATGCTTAGCTGAGAGCACAACATTGAGTGCTGCTTTAGCAATAATGACAGGGCAGGAGGAGACAGTGTCAGACACAGAGTGCATTGTGGCACCTAGGGTACCTGTGATTGTCGGTGATGCTCGTTCCTCAGCCTCAATAGGGGTGGCTGACACACTGGGAGTATTTGATATCCCCATGGTGAGTTATTTTGCATCCTGTGCTTGTCTCGGTGACAATCACAGATTTCATACTTTCCTACGCACTGTTCCCAGTGATGCCTTCCAGGCCAAGGCCATGGCCCGTCTGCTTCACCTGCTAGACTGGACATGGGTGGGGGTGGTCGCAGGGGATGATGAATATGGTAAAAGTGGTGTGCAGCTGCTTTTGAAAGAACTGGAACATGTAGGGGTTTGTGTGGACTACCTTGAATTCATTCCAAAATCCCACTCACAGAGAAGAATCAGACGAACTGTAGAGACAATACAGAGTTCCACAGCTCATGTTGTGGTGACATTCGCCATTGCCCCTGATATAGAAGTTTTATTCAAGGAAGTGGTGGTGCAGAATGTGACCAATAGGCAGTGGATAGCCACAGAGGCCTGGAGCACCTCTGTTCAGTTCCTTGACCCAGCAAGCATTCCTCTCTTAGCTGGTACACTTGGATTTGCCCTGCACAGAGCTGATATTGAGGGTCTTGGTGCTTATCTTTCACAGCTGAACCCTGCAAAACAGTCAAATGAACCATTTGTAAAAGATGTATGGGAAGAAATTTTTGGGTGCTCGTTAGCACAGGATTGGCAGCCTTCTTTCAAAAGGCCAAAGTGCACAGGGTCAGAGAATGTGGAAATTCATGGCGGCATCTACACAGATGTTTCACAGTTAAGAGTCACTTACAATGTGTATAAGGCTGTGTATGCCATTGCTTATGCTATTCACAACATGATTGCTTGCCAGCCTGGAAGAGGCCCATTTGAAAATGGACAATGTCCTGATGTGAACCAAATAAAGCCTAGACAGCTTCTCCACTACCTAAATGCAGTAAACTTTACAACACCAGTGGGTGAGCTGGTTTATTTTGAAGATAATGGTGAGCCATCTGCCTCTTATGACATTATGAACTGGCATGTGGATGAAAGTGGAGCAGTGAATTTTGTCCAGGTGGGACAGTTCGATGCAGCCAACGGACCAGGCCAGGAGCTGAACATAAACATTAAGAAAGTGGTTTGGGGAGGGGGCTGGTCTGACCAAGTTCCTGTGTCTGTGTGCAGTGTAAGCTGCCTTCCAGGCACAAGAAAGTCTGTACAAAAAGGAAAAGCCATCTGCTGTTTTGACTGCCTCCCTTGTGCAGCAGGGGAAATCAGTAACTTGACAGACTCTACAAAGTGTATAAGATGTCCAGAGAAGTTCTGGTCAAATGTTGAAAGAACAAAGTGTGTCCCAAAGATTGTGGAATTTCTGTCCTTACAAGATACCATGGGGATTGTACTGACAGTCTTATCTGTCACTGGGGCTACACTGACTACAACTGTTCTGGCAACGTTTTTCCATCATCGTGACACACCTCTTGTGCGAGCCAACAACTCAGAGCTGAGCTTCATGTTGTTGGTGTCGCTCACTCTCTGTTTCCTTTGTGCTCTAGTTTTTATTGGTCGGCCTGCAACATGGAATTGCATGTTACGTCACACTTTGTTTGGAGTGAGTTTTGTTATCTGCATTGCCTGCATCCTCAGTAAGACTGTGGTGGTGCTGGTAGCTTTTCAAGCCACTCTGCCTGGATCTAACTTGATGCAGTACTTTGGACCAATCCAGCAAAGGGCAGGCATACTCGTTTGCACAATGGTTCAGGTTGTCATATGTTTGCTGTGGCTGCTGCTGGCTCCCCCTCTGCCCACAGAGAGAGCAGGAGGAGAGTTTGGTGCTCGGGTGATCCTGCAGTGCACAGTGGGATCCGTTGTAGGGTTTGCATTAGTGTTGGGATACATTGGTTTGTTGGCAGTGGTCTGCTTTCTACTTGCTTTTTTTGCTAGAAAGCTTCCAGACAGTTTTAATGAAGCTAAATTTATTACATTCAGCATGCTGATTTTCTGTACTGTGTGGATTGTGTTTGTGCCAGCATATGTCAGTTCACCAGGAAAGTACACAGTGGCTGTGGAAATTTTTGCCATTTTAGCTTCCAGCTATGGTCTGCTCCTCTGTATATTCACCCCAAAATGTTACATTATTCTGCTTAAACCTGAGAAAAATACAAAAAAGAACATGATGGCCAAA

>OlfCu1

ATGCCAGTGATTGAACTGGTGTCACTGGGCCTTGCTCTTCTGTTGAACTTCTCAGTAGTTTTATGCACCTCATTGGACAAAAGTTGCTGGACATTGGGTGATTTTAATTCCCCAGTACTGGAACAGGATGGTGATATTGTAATTGGAGGGCTGTTTCCCATGCACAATATAGCTCCAGAAACTGACTATAATTTCTCTGATTTGCCACACTATCAAGATTGTAGCAGGTTTGACTTTCGTGCATTTCGCTGGGTTCAAACAATGGTCTTTGCCATTGAAGAAATCAACAGCAACTCAAGTTTGTTACCTGGAGTGGCACTAGGTTATAGGATCTTGGATAGCTGTGATCATGTGCACACCAGTCTGCGCAGTGCACTTTTTCTTCTGAACGGCACATTTGGTCAAACACAGACTGATGCTGTTGGCACTGCAAATTGCCTGTCCACAGCCCCAGTGCCAGCTGTCATAGGCCTGGCATCATCTTCACCTACACGAGCTGTGGCACAAACTTTGGGTCCGTTTGGAATTCCATTGATAAGTTACTTTGCCACATGCACTTGCCTCACAGATAAAAAGGAGTATCCTTCTTTCCTTCGCACTGTCCCTAGTGACAGGTTTCAGGTGCAGGGACTGGTCCAGTTGGTTTCCCACTTTGGTTGGCGCTGGGTGGGTACAGTGGGCACGGATGATGACTACAGTCACTATGGTATCCAGGCATTTACCGAGCAGCTTGAAAGACTTGGTAGCTGTATTGCCTTTCATCAAACTATACCCAAATCACCTTCTCAGGCACAGATACGTTCTATTCTTAATAGTCTTGAAAGTTCTACAGCACAAGTAATTATAGCATTTGCCACTGAAGGAGAACTGCTGGAGCTCCTGGTTGAAGTTGCTCGCCGGAATCTGACTAGGTTGCAATGGGTGGCCAGTGAGGCCTGGGTGACAGCCAAACTTCTCACAATCCCCGAGCTTCATCCTGTCCTGATAGGAACAGTGGGCTTTTCTTTCAGGGGCACCGCTATTCCTGGCTTGGCTGAGTTTCTCTTCAGGGTAAGGCCTTCATCGAGACCTGAATCTGCTTTCACTAACATGTTTTGGGAGGAACTGTTTGGATGCAGACTAGGTTACGAGGACAGCAATGATTCTGTGTTGCCACTCTGTACTGGCTCAGAGAATCTGCTGGAGACGGAAAGTAGCTACACTGATGTATCAAGGGTGAGGATTTCATACAATGTCTATAAGGCTGTCTATGCTATAGCTCATGCTCTACACCAGCTATTACAATGTGGCTCTAAGGAACAAGGCTCAGATCACAGCTGTAATACTGAATCAAAGTTCTCTCCATTGCAGCTTCTACGCTATCTGAAGAAGGTACACTTTACAAACCAGTTCAATGAAAAAGTCTATTTTGACACCAATGGAGAGCCTGTGCCACTCTATGACATCATAAACTGGCAGAAAAATGCTAGAGGTACCATCAATTTTCAGTTGGTTGGCACCTATGACGGCTCCGCCCCACATGGGCAGCAGCTTAAAATAGAAGAGGGCTTAATTCGGTGGACAGGCGGACAAACCAAGGTTCCAGTTTCCTTATGCAGTCCTCCTTGCCCACCTGGAACCCGCCAGGCCACTCGACCAGGACAGCCTGTCTGCTGCTTTGACTGTTTGCCTTGTGCTGAAGGAGAAATCAGCAATATATCAGGTGCTACTGAGTGTCTCAAGTGTCCAGAGTACTACTGGCCAAACAAAGAAAGAGTTTCCTGTGTTGCTGGCATTGAGGAGTTTCTGTCATACCATGACGTGATGGGGATCATTCTCATTTCTTTGTCTCTATTTGGTGTTGCTGTGGCAACAGTTGTTATGGTGATTTTTTTTATTTTCCGAAGCACACCCATAGTTAAGGCCAACAACTCTGAGATGAGTTTTCTACTGCTGCTGTCTCTCAAACTTTGCTTCCTTTGCTCACTGGTGTTCGTGGGTCGACCCTCGCCATTGACATGCCGCGCCCGACAAGCAGCATTTGGAATCAGCTTTGTTCTATGTATCTCATGCATACTGGTCAAGACCATAGTGGTGCTTTTGGCCTTTCGTTCTACAGTTCCTGGATCCATTTCTCTAAAGGTGTTTGGCCCCCCACAGCAAAGGGTTTTCATTTTCTGCTGCACAACAGGACAAGTTATTCTGTGCGCTTGTTGGTTGGCACTAGCACCTCCTTACCCCTATAAAAACACATCCTATCAGGATGGCAGGATTATACTAGAATGCAAAGACATTTTGCCACTTGGGTTTTACCTGGTTCTGGGCTATATAGGATTGCTCTCTTGTATGTGCTTTGTCCTCGCTTTCCTTGGAAGGAAACTTCCAGATACATTTAATGAAGCTAAGCTTATCACATTTAGTATGCTCATATTTTGTGCAGTGTGGATCTCTTTTATACCAGCATACAACAGTTCTCCAGGGAAATACACAGTTGCTGTTGAGATATTTGCCATTCTAGCTTCTACTTTTGGACTTTTATTTAGCATTTTTATACCTAAATGTTATGTAATTCTGTTAAGGCCTGATTTAAACACCAAAAAGGGAATGACTGGAAAATTGTCGAAA

>OlfCv1

ATGATTCATATTTTTCTTTGTATTTTTCATCTTTCCAGTGCAGCTATGACTTCAAGATGTATTCCAAGAGGGAACTTTGATCTGCCAGTTTTCATGACTAGTGGAGACTTCACAATAGGAGGAATTTTTCCTTTACATTACAGAGTAGAGCTTCCACCAACAGACTATATGAAGAAACCTTTAACAGCAATGTGTCGAGGGTTTGATCCAATGGCTTTTCACTGGGCTCTCACAATGGGACTGGCCGTAGAAGAGATAAACAATCGAAAAGACCTCTTACCAGAACACACTCTTGCCTATAGAATCTTTGACTCTTGTGCAACTCCTGTAATGGCTCAGAAAGCAGTCCTGGCAGCGCTGAATGGACAGGACGTTGTTCAAAGTTTCATGTGCTCTGGAGCTAGCCCCTTATTAGGATTAATTGGGGAGTCTGGATCCTCACAATCAATTGTTGTCTCCAGAACTTTGGAGCCTTTCAGAATACCAATGATAAGCTATTTCTCCACCTGCTCTTGTCTAAGTGATCGAAAGCAGTTCCCGACATTTTTCAGAGTGGTTCCAAGTGATGACTACCAGGTCAAAGCGATTGCACAGCTCCTAAAGAGATTTGACTGGACATGGATTGGTGTTGTGACTGAAGACCATGATTATGGCAGGTTTGCTTTACAAGGCTTAAAGCGAGAAATTGAAAATACAAAAATTTGTTTGGCTTATCATGAAATGATTCCAAAAGACTATACCCAGGAACGAGTCTTGAAGATACTTAAAGTTATGAAGGAATCAACAGCCAAGGTGGTTGTTGTTTTTTCAGGGGAAGGGGAATTTTACCCTTTTTTGACAGAGTTTGTGGCTCAGAATATTACAGGAATTCAGTGGATTGCAAGTGAGGCTTGGGTTACAGCATCAATGTTAGCAGAGACATATTCATTTTTAGATGGCACAATTGGCTTTGCAATCCGTCAAGGACACGTCCCAGGTCTTCAGGATTACATCAGGACCGTCACTCCAGAAAGGTACCCTTCTATTCCTCAGGTTCAGGAACTGTGGGAGGCTTTGTATGGTTGTTCTCCATCCACATCCACCTTGAGCAGTCATTTACCCTCATGCACAGGAAAAGAAATTCTCAGAAAAGAATACTCTGCCTACATGAACACATCCAGCCCTCGTGTGACCTACAATGTTTACAAAGCGGTGTATGCCTTTGCTCATTCTCTTCATAATCTTATTGAATGTAGAAATGGACATGGGCCATTTGAGAATTTATCATGTGCGAACCTCAACAATGTGTTTCCATGGCAGCTTCAACATTATTTACAGGAAATATCCTTCTCTATTTCTGGAGAGGAAGTTAATTTTGATATCAAGGGAGATGCAATACCATCTTATGATTTAATAAACTGGCAAAGAAGTGCAAGTGGAGATATGCAGTTTATTAAAGTGGGCCTCTATGATGGTGCTCAGCATTCTGGCAAAGAACTAGTGATTGAGAAGCAGGCTATTGCATGGTCTAACCAACAGACTAAGGTACCTGTTTCTGTATGCAGTAACGGCTGCACTCCAGGATACAGGAAGGCTGTCCGACATGGGCAGTCTCTGTGTTGTTTTGATTGTGTTCCATGTGACATTGGCAAGATCAGTAATCAGACAGATTCAGTAGATTGTCTGTCTTGCCCTGAAGACTATTGGTCCAATGTAAATAGAACAGAGTGCATCCAGAAAGTGATTGAATTTCTCTCCCATGATGCGATGGGAATGACCTTGACTGTGATAGCTGTAGCAGGAGCCTTTCTGACCATCACTGTGCTGGGGATATTTCTGTACAACAAAGGAACCCCTATAGTCCGTGTGAATAACTCTGAACTCAGTTTCTTCATCTTGGTATCACTGACTTTTTGTTTTCTGTGTGCTTTGATATTTATCGGGGAACCCACATCCTGGTCCTGTATGCTTCGGCACACTGCATTCAGCATCACCTTCTCACTTTGCATCTCTTGCATCTTGGGAAAGACTTTAGTGGTGCTGGCTGCTTTCACAGCGACCCGACCTGGAAACAATATAATGAAATGGTTGGGCCCTACACAGCAGAGAATCATCATTTTCTGCTGCACTCTCGTTCAGGTGCTCATATGTACAGTCTGGCTTGTAGTATCCCCACCATTTCCCTATAGAAACACTAAATATCAACAGTCCAAGATCATTCTGGATTGCAGTGTGGGCTCTGATCTGGCTTTTTGGTGTGTGCTGGGATATATCGGTCTTTTAGCTTGTGTCTGCTTTTTTCTGGCTTTTCTGGCCCGGAAATTACCAGGTAATTTTAATGAGGCCAAATACATCACCTTCAGCATGATTATATTTTGCACTGTATGGCTAGCTTTTGTGCCTGCATATGTCAGTTCGCCTGGTAAGTTTACAACTGCTGTGGAGATTTTTGCTATACTAGCTTCTAGTTTCGGCCTGCTTTTCTGCTTGTTTGCTCCAAAATGTTACATTATTTTAGTGAAGCCAGAGAAAAACACCAAGCAACATTTAATTGGAAAAGTCACAAAA

>OlfCv2

ATGATTTACATTTTCATGTCGATATTTCATCTTTCCAGTGCGTCTATGGCTTTAATCTGCTCCCCACATGACTTTGATCTGCCAGTTTTCATGACTAGTGGAGACTTCACAATAGGAGGAATTTTTCCTTTACATTACAGAGTAAAGCTTCCACCAACAGACTATATGAAGAAACCTTTAACAGCACAGTGTCGTGGGTTTGATCCAAGAGCTTTCCGTTGGGCTCTCTCTATGAAGCTTGCTGTGGAGGAGATAAACAATAGAAAAGATCTCCTCAACAATTTTACACTTGCTTACAAAATATTTGACTCTTGCTCTACTCCTGTAACAGCTCAGAAAGCAGTTCTGGCTGTAATGAATGGGCAGGAAGTTGTTCAAAGTTCTATGTGCTCTGGGGCTGGCCCTTTAATAGGATTAATAGGAGAATCTGGATCTTCACAGTCTATTGTTCTCTCCAGAACAGTGCAAGCCTTTCAAATACCTATGATAAGCTATTTCTCCACCTGCTCTTGTCTAAGTGATCGAAAGCAGTTCCCGACATTTTTCAGAGTGGTTCCTAGTGATGACTACCAGGTCAAAGCGATTGCACAGCTTTTAAAGAGGTTTGACTGGACATGGATTGGTGTTGTGACTGAAGACCATGATTATGGCAGATTTGCTTTACAAGGCTTAAAGCGAGAAATTGAAAATACAAATATTTGTTTGGCTTATCATGAAATGATTCCAAAAGACTATACCCAGGAACGAGTCTTGAAGATTCTTAAAGTAATGAAGGAATCAACAGCCAAGGTAGTTGTTGTTTTTTCAGTAGAAGGAGAGTTTTATCCTGTTTTAAGAGAGTTTGTGGCTCAAAATATCACTGGAATTCAGTGGATTGCAAGTGAGGCTTGGGTTACAGCATCAATGTTAGCAGAGACATATTCATTTTTAGATGGCACAATTGGCTTTGCAATCCGTCAAGGACACGTCCCAGGTCTTCAGGATTACATCAGGACCGTCACTCCAGAAAAGTACCCTTCTATTCCTCAGGTTCAGGAACTGTGGGAGGCTTTGTATGGTTGTTCTCCATCCACATCCACCTTGAGCAGTCATTTACCCTCATGCACAGGAAAAGAAACTCTCAGAAAAGAATACTCTGCCTACATGAACACATCCAGCCCTCGTGTGACCTACAATGTTTACAAAGCGGTGTATGCCTTTGCTCATTCTCTTCATAATCTTATTGAATGTAGAAATGGACATGGGCCATTTGAGAATTTCTCATGTGCGAACCTCAACAATGTGTTTCCATGGCAGCTGAAGCATTACCTTGAGGATGTTTCCTTCTCAATATCTGGACAAAATGTTAACTTTGATAATAAAGGAGACTCAGTTCCATATTATGATTTAATAAACTGGCAAAGAAGTGCAAGTGGAGATATGCAGTTTGTTAAAGTGGGCCTCTATGATGGTGCTCAGCATTCTGGCAAAGAACTGGTGATTGAGGAGCAGGCCATTACATGGTCTAACCAACAGACTAAGGTGCCCGTGTCTGTGTGCAGTTATAGGTGTGCACCAGGATTTAGGACAGCTGCCTGTCGTGGGCAACCTTTGTGCTGCTTTGACTGTGTTCCATGTGAAAGTGGCAAGATCAGTAATCAGACAGATTCAGTAGATTGCCTGCCATGTCCTGAGGACTATTGGTCAAATGCAAATGGTACATCATGTGTACCAAAAGTGATTGAATTTCTCTCCCATGATGCGATGGGAATGACCTTAACTGTCATAGCTGTAATAGGAGCCTTTTTGACCATCACTGTGCTGGTGATTTTTCTGTACAACAAAGGAACCCCTATAGTCCGTGTGAATAACTCTGAACTCAGTTTCTTCATCTTGTTATCACTGACTCTTTGTTTTCTGTGTGCTTTGATATTTATCGGGGAACCCACATCCTGGTCTTGTATGCTTCGGCACACTGCATTCAGCATCACCTTCTCGCTTTGCATCTCTTGCATCTTGGGAAAGACTTTAGTGGTGCTGGCTGCTTTCACAGCGACCCGACCTGGAAACAATATAATGAAATGGTTGGGCCCTACACAGCAGAGAATCATCATTTTCTGCTGCACTCTCGTTCAGGTGCTCATATGTACAGTCTGGCTTGTAGTATCCCCACCATTTCCCTATAGAAACACTAAATATCAACAGTCCAAGATCATTCTGGATTGCAGTGTGGGCTCTGATCTGGCTTTTTGGTGTGTGCTGGGATATATCGGTCTTTTAGCTTGTGTCTGCTTTTTTCTGGCTTTTCTGGCCCGGAAATTACCAGGTAATTTTAATGAGGCCAAATACATCACCTTTAGCATGATTATATTTTGCGCAGTATGGCTAGCTTTTGTACCTGCATATGTCAGCCAGCCTGGTAAGTTTACAACTGCTGTGGAGATTTTTGCTATACTAGCTTCTAGTTTTGGCCTGCTTCTTTGTTTATTTGCCCCCAAATGTTACATTATTTTAATGAAGCCAGAGAAAAACACCAAGCAGCATCTCATGGGAAAAGTTATAACA

>OlfCv3

ATGCATTCATTAGAGACAATGACTATCCTGCTTCTGTGGCTACTGGTTTACCATCTCAGTGTACTCAATCCCGCACAAGCTTCCAAATGCCTACTGCAAAATGAATTTGAGCCAGGCCTCATGGCTAATGGAGATTTTGTAATAGGTGGTATTTTTCCTTTACACTATAATCAGGAAATGCCAGACCTCAACTTTACTTACAAACCTGGGCCGGTCAAATGTAATGGGTTTGATACGAGAGCTTTTAGATGGGCCATTACAATGAAACTTGCAGTGGAAGAAATTAACAAGCGAGTTGATCTCCTTCCTAACTACACTTTGGGTTACAAAATCTTTGACTCTTGTGCTTACCCTCTGACAGGACAGAGATCAGCAGTGGCTGTTCTAAATGGGCCAAATGAACTAGAAAGCCCCTTGTGTGCAGATGCTGCTCCACTGCTTGCTGTGATAGGAGAATCTGGGTCAGCTCAGTCTATTGTAGTTTCTAGAATTTTGCAACCTTTTGGGATACCAATGATCAGTTATTTTTCATCATGTGCCTGTCTGAGTGATAGGAGAGAATTCCCCACATTCTTCAGAGTCATACCTAGTGATGCTTATCAAGTGAAGGCCATTGCCAAACTCCTGCGGCATTTTAACTGGACATGGATCGGAGTGATACGAGGAGATCACGAGTATGGCCGCTTTGCCCTTCAGGGCTTACTGAAGGAGCTGGAGGGTACAGGCATATGTGTTGCTTATCAGAAGATGATCCCTTTGTTATATGATCGTCAGAAGGCGCTGGAAATAATACATGTCATGAGCCACTCCACTGCCCGTGTAGTAGTGGTGTTTTCAGCTGAAGGTGAGCTCACACCTTTCTTGAGAGATTACATGGAACAGAATGTGACAGGCATACAGTGGATCGCAAGTGAGGCTTGGGTTACATCCTCTGTGTTTGCAGGAAGTGAATTTGACCCTTTCCTTGGAGGGACTATTGGATTTGGCATTCGACAGGGTCAAATTCCTAGATTAAAAGAATATCTCACAACTGTGAACCCAGAAAGGTACCCCACAAATCCTCTGGTTTATGAGCTTTGGGGGGCTTTATATGGCTGTTCCCCCTCATGGTCCAACCTGAGTAGTCACTTGCCATCCTGTACAGGGAAGGAGACTGTTAGGCTGCAGTATTCTGCCTATTTAAACACATCCAGTCCTCGCATTTCCTATAATGTCTACAAAGCAGCATATGCCATTGCATATTCCCTGCACAATCTTATTTACTGTACCCCTGGAAAAGGACCATTTAGTAACTCCACATGTGCAAACGCACCTCATATCTACCCGTGGCAGTTACAACAGTATCTCCAAGAGGTGTCATTCACTATTTCAGGAGAGAAAGTGAACTTTGACATGAAAGGTGATTCTATTCCATCCTATGACCTCATAAACTGGCAGAGGGGCTCAGCAGGAAATATTGAGTTTATTAATGTGGGCATGTTCGATGGTGCACTTGAATCTGGCCAAGAGCTGGTCATCAAGGAGGAGGCTATCATGTGGCCCGGCCATCAGACTGAGGCAAGCTGTTCTCACAGTGTGCTGGTGTCTGTGTGCAGCAACAGCTGTGCTCCGGGATTCAGGAAGGCTGTTCGTCGTGGCCAGCCTCTGTGCTGCTTTGACTGTGTACCATGTGACAGTGGCAAAATCAGTAATGAGACAGATTCACTAGATTGTATTGCCTGCTCTGAAGATTACTGGTCAAATGCTGATGGAACAGTGTGCATTCCTAAAGTGGTTGAATTCCTGTCCCATGATGCAATGGGATTAACCCTGACAGTAATAGCTGTAGCAGGAGCCTGCCTCACATTAGCTGTGTTTTCCGTGTTTCTTTATTACAAAAACACTCCTGTAGTACGCATAAATAACTCCGAACTCAGTTTCTTCATCTTGTTATCACTGACTCTTTGTTTTCTGTGTGCTTTGATATTCATCGGGGAACCCACATCTTGGTCCTGTATGCTTCGACACACTGCATTCAGCATCACCTTCTCGCTTTGCATCTCTTGCATCTTGGGAAAGACTTTAGTGGTGCTGGCTGCTTTCACAGCGACCCGACCTGGAAACAATTTAATGAAATGGTTGGGCCCTACACAGCAGAGAATCATCATTTTCTGCTGCACTCTCATTCAGGTGCTCATATGTACAGTCTGGCTTGTAGCATCCCCACCATTTCCCTATAGAAACACTAAATATCAACAGTCCAAGATCATTCTGGATTGCAGTGTGGGCTCTGATCTGGCATTTTGGTGTGTGCTGGGATATATCGGTCTTTTAGCTTGTGTCTGCTTTTTTCTGGCTTTTCTGGCACGGAAATTACCAGGTAATTTTAATGAGGCTAAATACATCACCTTCAGCATGATTATATTTTGCGCAGTATGGCTGGCTTTTGTGCCTGCATATGTCAGTTCACCTGGTAAATTTACAACTGCTGTGGAGATTTTTGCCATTTTAGCCTCTAGTTTTGGCCTGCTTCTTTGTTTATTTACCCCAAAGGTTTATATCATTTTAGTGAAGCCAGAGAAGAACACAAAACAACATCTCATGGGAAAAGACAAG

>OlfCw1

ATGAAGACGCCAGTCCTCCGAGCTATACATTTGGTATTTTTTTTATTCTACCCTATTCAGTCCAACTCAGAGGCAAATTGTAAACTCTGGAAGGAATTAGACTTGACTGTTGTGCACAAGGAGGGTGATGTAATTCTCGCAGGCATGTTTCCTATTCATTCCAAAGGCATTGACCAGGAGTTAAATTTTAGGAATCAGCCAGACCAGAGGAAATGTTGGGGGTTTAACATGCGCGTTTTCCGCTGGTCTCAGGCAATGATTTTCTTCATTGAAGAGATCAACCGGAACCCCACTCTTCTCCCAAACATCACACTGGGCTATAGATTGTATGACACATGTGGGCTGATAGCGCTGTCTCTTAGGACTGCATTGTCTGTAGTTTCTCAACCCATGAAGAGGAGTAGCTCAGAGATGTGCTCTTCCCCGAGCATCCCCATCATTATTGGGGACTCTGGATCCACTCTGTCAATGGCCATTTCCAGACTCCTCAACCTTTTCCATATCCCGCTGGTGAGTTATTTTGCTTCATGTGCTTGCCTGAGTAATAAGCATCAGTTCCCTTATTTTTTTCGCACAATTCCCAGTGATGTAAATCAGGCAAATGCTCTTGCTCGTTTGGTGAAGCACTTTGGATGGACCTGGGTGGGCACAGTCGGGGCCGATGATGCTTATGGCCGCACCGGCATAGATCTTTTCACCGCTGCAGTGACACAGTTGGGTGTATGTGTGTCTTATAGAATAATCATACCCAAACTGCCAACTCAACAACAGTTGCAAGACATAGTCAGGACAATTCGTGATTCTTCAGCTCATGTGTTGGTGGCTTTTGCTATTGAAGAGGATATCAAGCCAGTAGTTGATGAAATAGTCTTGCAGAATGTGACCGGGAAGCAGTGGGTTGCCAGTGAAGCGTGGGTGACATCCACTCTTATCTCCACTAAAGAAAACTTTCCCTCACTTAGTGGCACCATTGGGTTTGCTATTCGCCGAGCTGAGATACCTGGCCTCAAACATTTCCTTGACAGCATACAGCCCCTAGCTGACCCATACAATGTCTTTGCAAGAGAGTTCTGGGAAACCCAGTTTCAGTGCACATTAAACACAAGTCTTCCCACTTCATCCACAATGGATCCAGTAAATTATAGCCACAGCTGTACTGGCATGGAGAGAATGCAGGACACTCAGAGCATCTTTAATGATGTGTCTCAGCTCAGAGTGACATATAACATGTACAAAGCTGTCTACACTGTGGCACATGCACTACATAATCTGCTTCTGTGTCAAAGAGAGCATAGATCTGCCTTGACACAGCAGTGTCCTGATATCCACAATCTGCAGCCCTGGCAGGTAATTGAGGTCCTGAGAAAAGTGAATTACACAAACATGTTTGGAGATCTCATCTACTTTGATGAAAATGGAGACCCTGTTGGATCATATGACATAGTGAACTGGCAAAAAGGGGGAGATGATGTGCCAGTACAGTATATTACAGTGGGTCGCTTTGACTCCTCTTTACCCAAAGGACAACAATTAGTGCTGAACCAGAACAAAATCGTCTGGCATGGGGGAACAAACAAGGTGCCAGTGTCTGTATGCTCTGCTAGCTGTCCACCAGGGTACAGAAAGGTTAGACTTGAGGGTCAGCCTGTGTGCTGCTATGACTGTATGCTTTGTGCAGAGGGGAGTATCAGCAACACAACTGATCAAGCTGAATGTTTGCTTTGCCCTGAAGACTTCTGGTCCAACAAGCATCGCAACTATTGTGTTCCAAAGGAGATTGAGTTCCTTTCATACTTGGAGGCTTTCGGTATGGTCCTGGCAGCTATTGCCATATTGGGTGCAGTTGCTGCAATAAGTGTTGGTGTGGTCTTCTTCCGTCATCGTGACACACCCCTTGTTCGTGCTAATAACTCAGAATTGAGCTTTTTGTTGTTGATTTCTCTCACTCTCTGTTTCATATGTGCACTAACCTTTCTTGGTCAGCCATCACACTGGGCATGCCCTCTTAGACGTATTTCTTTTGGCTTAACTTTTGCCCTCTGCCTATCTTGTCTCCTCAGTAAGACCCTAGTGGTTCTTATTGCTTTTAAATCCACACTGCCAGGTAACAACACAGCTCGTTGGTTTCGCCCCCCTCAACAAAGATTGGGTGTGTTTATTTGCTCACTCCTACAGGGTGGTGTCTGCATAGCTTGGCTGACCACAGCATCCCCGTATCCAGTGAAGAACACATGGCTATATCGCGACCGGATTATTTTAGAATGTCATCTGGGTTCAGTTGTTTATTTCTGCTGTGTGTTGGGTTACATTGGGTGCTTAGCTGCCTTTTGCTTCATTTTAGCCTTTTTAGCTAGAAAACTGCCTGATAATTTTAATGAAGCCAAATTCATCACATTCAGTATGCTTATTTTCTGTGCTGTTTGGATTACATTTATCCCAGCTTATGTCAGTTCCCCAGGGAAATTTACCGTTGCTGTGGAGATATTTGCAATTTTAGCGTCTAGTTATGGTGTTCTTTTGTGTATTTTTGCACCTAAGTGTTATATCATTATTTTCATGCCGGAAAAGAATTCTAAAAAGTATCTCATGACCCAAAAA

>OlfCx1

ATGTGTACCACAGGTTGTAGCCATCAGCATTGCTCAGTATCAACGATGGCACAGATTTATGCAACTCTCCTTACATTGTGTCTTCTACTGTATATTAGGGTGCAGTGCCAATCTAACAACAAATATAAGTGCATCTATCAAGGGGATGATGATACTTACAGCTTTTACCAGGGTGGAGATTTAGTTTTGGGAGGGATTTTTCCTTTGCATTCCAGCACAATACCACAGTTTTTTTCTTTTACTACCAAACCCAAGTTAATTCAATATAAATTCTTCACCCCTCGAGCCCTAAGGTGGATGCAAACAATGATCTTTGCTGTGAGAGAAATAAACCAACGACAAGATCTTTTACCAAACCTGTCTCTGGGCTACCACATCAGGGACAGCGGTGATGATATACCAGTGTCTGTGAAAAGGTCTCTTCTCCTGGTCAATGGACAGCCAGAGAAGGGCAGTGGACAGAGCTGTGAAGATACACGAAAGCAGCCAAGTCCTGTAATCGTGGGAGAGGCATCATCAGGCGTGTCCATGGCTGTTCTGAGAACTTTAGGCACCTTCCAAATACCCTTAGTGAGCTACTTTGCATCATGCTCTTGTCTCAGTAACAAAAGGGAGTTTCCAGCATTCATGCGCACAATGCCAAGTGACCTATTTCAGATTAAAGCTTTAGTAAAACTTGTTTATTATTTTAAGTGGACATGGCTTGGGGTTATCGGTGTGGATACAGACTATGCTCGATTTGCCATCCAGCTGTTCTTGAAAGAGTCAGAAAAATATAATATTTGTCCTGCCTATGTTCACATTTATCCTGTTGCGCTTACTCAAAATACAGTTGAGGAACTTGTTAAGATTTTAAAATCATCTTCTGCTAGAGTCATACTAAATTTCTCTGTGGACTCTTATCTTTATGGTGTTCTAAAAGAATGCAGACGGCAAAATGTCACAAATCTGCAGTGGATCGCCAGTGAGGCTTGGGCCACATCAAAAGTCCTCTGGGATGACTTTGGTGATTTGTTAAAGGGAACACTGGGCTTTGCCATACGGAGAGCTGATATTCCGCATCTTGGCAGTTATCTCAGATCAGTAAGCAGTTCTGTTGCTCAGACCTCCCCTTTTTTTACTGAGTTTTGGGAAGAGACTTTCCATTGCAGACTAAATGGATCCTTAAACACTCATGTACATGAAGAAGCATCTTACAACTGGCCAGCCTGCAATGGTAATGAAACTCTGGATGATGTGTATACTCTTTATTCGGATGTGTCACAACTCAGAGTCTCCTACAATGTTTACAAAGCAGTGTATCTCATAGCACATGCACTGCATGACATGAGCACATGTGATCCTGGAAAGGGCCCATTTAAGAATGGTACATGTGGGAGTCTGTATCAAATTTTGCCATGGCAGCTTTTGTACTATATGAAACGAACCAACTTCACAACACTCGGAGAAGAGGTTCGATTTGATAAGAATGGGGATCCTATTGCATCCTATGATCTGATGAACTGGCAACGAGAATCTGATGGCTCCCTTCAGCTTGTCAGGGTGGGAATCTATGATGCCTCCTTCAAAGATGACAAGGACCTAGTGATAGATGAGTCAGTAATTATGTGGCACAGAGGGGACAAGGCGCCAGAATCTTTGTGCAGTAAAAGCTGTCTGCCAGGCTCCAGGAAGGCCAGGCAAAAAGGAAAGCCAGTCTGCTGCTTTGACTGTATATCATGTGCTGAAGGAGAGATCAGTAATCAGACAGACTCAATTGATTGCCTGACATGTTCCAAGGACACGTGGCCTAACCAATCCCAAGACCAGTGCATCCCCAAAACTTTAGAGTTTTTGTCCTTTCAGGAGCCCTTGGGAATAATTCTTTGGGTATTTTCAGCATTGGGGGCCTGTGCAGCATTAGCAGTGCTCTGTGTGTTTGTAATGTATAGGACGACTCCAGTCATACGAGGAAACAATATAGAGTTAAGTTTCCTTCTTCTGTTGTTCCTCTGTGCTTGTTTTTTGATTGGCCTAACATTTTTAGGGAAGCCCACTGACTGGTTGTGCCAAATTCGCTATCCAGCTTTTGGAATCAGCTTTACGCTCTGTATTTCCTGCATCTTGGCAAAAACTGTAGTCGTTTTAATGGCTTTCAGGGCCACCATCCCTGGCAATAATGTCATGAAGTGGTTTGGTCCTGTTAAACAAAGATCAAGTGTTATCCTGTGCACATGTGTGCAAGCACTTATTTGCATCATATGGTTAACAACTAAGCCCCCTCTTGCTTCATACAACAGCAAGTTCCTGAGCGCAACTATAATTGTTGAATGTTCTGTGGGATCTGAAGTTGGATTTTGGTGTGTTCTCGGATATATTGGCTTCCTATCTTCCTTGTGCTTCTTCTTGGCATTTTTGGCCAGAAAACTGCCAGACAACTTTAATGAGGCTAAATTCATCACATTCAGCATGCTCATATTTTTTGCTGTGTGGATAACCTTCATTCCAGTCTACGTGAGCTCAAGTGGAAAGTATATGGTTGCTGTCCATGTATTTGCTATTCTAGCTTCTGCCTTTGGTCTTTTAATGTGTATATTTGCTCCTAAATGTTATGTTGTATTACTGAAACCAGAGAGAAATGACAAAAAGAGTATGATGAAGAAA

>OlfCx2

ATGGCACTGTTATGTGCAACACTCCTTATGGTTTGGTTTCTGATGTACCTTAGTGTACAGAGTCAGTATAATAGCAAGGTTAGGTGTGTCTTTCAGGGAGATGATGATACAAACACCTTTTACCAGGGGGGAGATGTGGTTTTGGGAGGGCTCTTTCCTTTACATTTCAGTCCTATTTCATCGCTTTCTTCTTATAAGACCAAACCTACTCCAACCACATATAAATTTTTTACCCCTCGAGCCTTGAGGTGGATGCAGACAATGATCTTTGCTGTAAGAGAAATAAACCAACGACAAGATCTTTTACCAAACCTGTCTCTGGGCTACCACATCAGGGACAGCGGTGATGATATACCAGTGTCTGTGAAAAGGTCTCTTCTCCTGGTCAATGGACAACCAGATAAGGACAGTGGACAGAGCTGTGAAGATACACGAAAGCAGCCTAGCCCTGTAGTTGTGGGAGAGGCATCATCAGGAATATCTATGGCGGTTCTGAGAAGCCTAGGCTGCTTCAAAATACCTTTAGTGAGTTATTTTGCATCCTGCTCTTGTCTCAGTAACAAGAAAGAGTTTCCAGCATTCATGCGGACCATGCCAAGTGATCTATTTCAAATCAAAGCTTTAGCAAAACTTGTGCATTATTTTCAGTGGACATGGGTCGGGGTGATTGGTGTGGATACAGACTATGCCCGATTTGCAATCCAGCTCTTTTTGAAAGAGTCACAACAGTTGAATATTTGTCTTGCTTATGTCCAACTTTATCCTGTTGCACTAAATCAAGATTCAGCAGAAGAACTTGTGAGGATGTTGAAATCTTCTTCTGCCACAGTCATAATAAGCTTCTCTGTAGACTCTTATCTTTATGGTATTCTAAAAGAATGCAGACGGCAAAATGTCACACATCTGCAGTGGATTGCCAGTGAGGCTTGGGCCACATCGAAATCCCTCTGGGAGGAATTTGGTGATCTGCTAAAGGGTACATTGGGTTTTGCCATACATAGAGCTGATATCCCACATCTTGGCAGTTATCTTAAAGAAATAAGACCTCAGACCTCCCATTTCCTCTCTGAGTTTTGGGAAGAGACTTTTCGTTGTCGACTGAATGGTTCCTTAAACACTCATGCACATGGAGAACAGGCACAAAACTGGCCAGCCTGTAATGGTAGTGAAAGTCTGGATGATGTGTACACCACTTATTCAGATGTGTCACAACTCAGAGTCTCCTACAATGTTTACAAAGCTGTGTATCTCATAGCACATGCACTGCATGACATGAGCACATGTGTTCCAGGAAAGGGCCCATTCCAGAATGGGACATGTGGGAGTCTGTTTCAAATTCAGCCATGGCAGCTCCTGTACTACATGAAACAAACTAATTTCACAACCTTAGGGGAAGAGATCAGATTTGATGAGAATGGTGATCCTATTGCATCTTATGACCTTATGAACTGGCAAAGAGGATCCGATAGCTCCCTTCAACTTGTCAGGGTGGGATTCTATGATGCCTCCCTTGAAGATGACAAGGACCTAGTCATAGATGAGTCACTAATTATGTGGCACAGAGCAGAGAAGGCACCAGAATCATTGTGCAGTAAAAGCTGTCTGCCAGGGTCCAGGAAGGCCAGGCAAAAAGGAAAGCCAGTCTGCTGCTTTGACTGTATATCATGTGCTGAAGGAGAGATCAGTAATCATACAGACTCAATTGAATGCCTGACATGTTCCGAGGACACGTGGCCTAACCAATCCCAAGACCAGTGCATCCCCAAAACTTTAGAGTTTTTGTCCTTTCAGGAGCCCTTGGGAATAATTCTTTGGGTATTTTCAGCATTCGGGGCCTGTGCAGCATTAGCAGTGCTCTGTGTGTTTGTAATGTATAGGAAGACTCCAGTCGTACGTGGAAACAATATAGAGTTAAGTTTCCTTCTTCTGTTGTTCCTCTGTGCTTGTTTTTTGATTGGCCTAACATTTTTAGGGAAGCCAACTGATTGGCTATGCCAAATTCGCTATCCAGCTTTTGGAATCAGCTTTACACTCTGTATTTCATGCATCTTGGCAAAAACTGTGGTCGTTTTAATGGCTTTCAGGGCCACCATCCCTGGCAATAATGTCATGAAGTGGTTTGGTCCTGTTAAACAAAGATCAAGTGTTATCCTGTGCACATGTGTGCAAGCACTTATTTGCATCATATGGTTAACAACTAAGCCCCCTCTTGCTTCATACAACAGCAAGTTCATGAGTGAAACTATAATTGTTGAATGTTCTGTGGGATCTGAAATTGGATTTTGGTGTGTGCTGGGATACGTTGGCCTTCTTGCTTGCTTGTGTTTTTTAATGGCATTTTTGGCTAGAAAGTTACCAGACAACTTTAATGAGGCTAAATTTATCACATTCAGCATGCTCATATTTTTTGCTGTGTGGATAACCTTCATTCCAGTCTACCTGAGCTCAAGTGGAAAGTATATGGTTGCTGTCCATGTCTTTGCTATTCTAGCTTCTGCCTTTGGTCTCTTGCTATGTATATTTTTTCCTAAATGTTATATTGTATTACTGAAGCCAGAGAAAAATGATAAAAAACATATAATGAAAAAA

>OlfCx3

ATGGCAGGAGTTTATAAAGTGTTGATTTCATTTTGGCCACTGCTGTGTATCGGTGTGCAGAGCCAATATAAAAGCAAGGAAAACTGTGTCTTTCAGGGAGATGAAGACACATACAGCTTTTACCAGCGGGGAGATGTGGTTTTGGGAGGAATCTTTCCTTTGCATTCTAGCCCTGTATCATCGCTGTTTTCTTTCAGAACCAAACCTAAGCCAACAAGTTACAAATTTTTTACCCCTCGAGCCTTGAGGTGGATGCAGACAATGATCTTTGCTGTGAGAGAAATAAACCAGCGGCAAGATCTTTTACCCAATCTGTCTCTGGGCTACCACATCAGGGACAGCGGTGATGATATACCAGTGTCTGTGAAGAGGTCTCTTCTCCTGGTCAATGGACAGCCAGAAAAGGGCAGTGGACAGAGCTGTGAAGACATACAAATGCAGCCGAGTCCTGTGATAGTAGGTGATGCTGCATCAGGAGTCTCCATGACTGTTCTGAGAACTTTAGGCTCCTTCAAAATACCATTGGTGAGCTATTTTGCATCCTGCTCTTGTCTCAGTAACAAAAGGGAGTTTCCAGCATTCATGCGGACCATGCCAAGTGACCTATTTCAGATTAAGGCTTTGGTAAAACTTATTAATTATTTTCAATGGACATGGGTTGGACTGATAGGTGTGAATTCTGACTATGCTCACTTTGCTATCCAGCTCTTCCTTAAAGAATCAGAACAATTTAATATTTGTCCTGCTTATGTTCACTTTTATCCTGTTGTACTGATGGAAGATGCATTGGAGGAACTTGTGAACATTTTAAAAGCCTCTTCTGCCACAGTCATAATAAATTTTTCTGGAGAATCAGAACTGCATAGTATTCTAAAAGAATGCAGACGTCAAAATGTCACACATCTGCAGTGGATCGCCAGTGAGGCTTGGGCCACATCAAAATCCCTCTGGGAGGAATTTGGTGATCTGCTAAAGGGTACATTGGGTTTTGCCATACATAGAGCTGATATTCCACATCTTGGCAGTTATCTTAAAGAAATAAGACCTCAGACCTCCCATTTCCTCTCTGAGTTTTGGGAAGAGACTTTTCGTTGTCGACTGAATGGTTCCTTAAACACTCATGCACATGGAGAACAGGCACAAAACTGGCCAGCCTGTAATGGTAGTGAAAATCTGGATAATGTGTACACCACTTATTCAGACGTGTCACAACTCAGAGTCTCCTACAATGTTTACAAAGCTGTGTATCTCATAGCACATGCACTGCATGACATGAGCACATGTGTTCCAGGAAAGGGCCCGTTCCAGAATGGGACATGTGGGAGTCTGTATCAAATTCAGCCATGGCAGCTCCTGTACTACATGAAACAAGCAAATTTTACAATCTTAGGAGAAGAGGTCAGATTTGATGAGAATGGTGATCCTATTGCATCTTATGACCTTATGAACTGGCAAAGAGGATCCGATAGCTCCCTTCAACTTGTCAGGGTGGGATTCTATGATGCTTCCCTTGAAGATGACAAGGACCTAGTGGTAGAGGAGTCAGTAATTATGTGGCACAGAGCAGAGAAGGCACCAAAATCAATGTGTAGTCAAAGTTGTCTGCCGGGCACCAGAAAGGCCAGGCAAAAAGGAAAGCCAGTCTGCTGCTTTGACTGTATATCATGTGCTGAAGGAGAGATCAGTAATCAGACAGACTCAATTGATTGCTTGACATGCTCAAAAGAAACGTGGCCTAACCAAGCCCAAGACCGGTGCATCCCCAAAACTTTAGAGTTTTTGTCCTTTCAGGAGCCCTTGGGAATAATTCTTTGGGTATTTTCAGCATTCGGGGCCTGTGCAGCATTAGCAGTGCTCTGTGTGTTTGTAATGTATAGGAAGACTCCAGTTGTACGTGGAAACAATATAGAGTTAAGTTTCCTTCTCCTCTTGTTCCTCTGTGCTTGTTTTTTGATTGGCCTAACATTTTTAGGGAAGCCAACTGATTGGCTATGCCAAATTCGCTATCCAGCTTTTGGAATCAGCTTTACACTCTGTATTTCATGCATCTTGGCAAAAACTGTGGTCGTTTTAATGGCTTTCAGGGCCACCATCCCTGGCAATAATGTCATGAAGTGGTTTGGTCCTGTTAAACAAAGATCAAGTGTTATCCTGTGCACATGTGTGCAAGCACTTATTTGCATAATATGGTTAACAACTAAGCCCCCTCTTGCTTCATATAACAGCAAGTTCATGAGTGAAACTATAATTGTTGAATGTTCTGTGGGATCTGAAATTGGATTTTGGTGTGTGCTGGGATACGTTGGCCTTCTTGCTTGCTTGTGTTTTTTAATGGCATTTTTGGCTAGAAAGTTACCAGACAACTTTAATGAGGCTAAATTCATCACATTCAGCATGCTCATATTTTTTGCTGTGTGGGTAACCTTCATTCCAGTCTACGTGAGCTCAAGTGGAAAGTATATGGTTGCCGTCCATGTCTTTGCTATTCTAGCTTCTGCCTTTGGTCTTTTAATGTGTATATTTGCTCCTAAATGTTATGTTGTATTACTGAAACCAGAGAGAAATGACAAAAAACACATGATGAAAAAA

>Dr_CaSR

ATGAGGTTTCATCTGAAGTTTTACCTGCATTACCTGGTTCTACTGGGTTCCAGCTGTGTAATTTCGACCTATGGCCCAAACCAGAGGGCCCAGAAGACGGGCGATATCCTGCTTGGAGGACTTTTCCCAATGCATTTTGGGGTGGCCTCCAAAGACCAGGATCTTGCAGCACGGCCAGAGTCAACAGAGTGTGTCAGATATAACTTCCGTGGATTCCGCTGGCTGCAGTCTATGATCTTCGCTATAGAGGAGATCAATAACAGCTCCACTCTTCTTCCAAACATTACGCTTGGCTACCGTATATTTGACACGTGCAACACTGTCTCGAAAGCGTTGGAGGCGTCACTGAGTTTTGTCGCGCAGAATAAGATTGATTCACTGAATCTGGACGAGTTCTGTAACTGCACAGGGAACATCCCATCAACCATTGCAGTGGTCGGGGCTTCTGGATCTGCAGTGTCTACTGCCGTGGCGGACCTGCTGGGTCTTTTCTACATCCCTCAGATTAGCTATGCTTCATCCAGCCGCCTTTTGAGCAACAAAAACCAGTACAAATCCTTCATGAGGACAATCCCTACAGACGAGTACCAGGCGATTGCCATGGCTGCCATCATCGAGCACTTCCAGTGGAACTGGGTGATTGCTATCGCCTCTGACGACGAATACGGCCGGCCAGGCATTGAAAAGTTTGAGAATGAAATGTTTCACCGGGACATCTGCATTGACCTCAATGTTTTAATTTCACAGTACGTTGACGAAGCTGAGATTCGTCGCCTGGCAGATCGCATCCAGAACTCATCCGCCAAGGTAATTGTTGTGTTTGCTAGCGGACCAGATATCGAGCCGTTAGTTAAAGAGATGGTGAGACGAAACATCACAGACCGCGTCTGGTTGGCGAGCGAAGCATGGGCCAGTTCCAGTCTAGTCGCCAAACCAGAGTATCTTGACGTCATGGGAGGAACCATTGGCTTTGCTCTGAGGGCTGGACATATACCCGGCTTTAAAGACTTCTTACAGCAGGTCCATCCAAAGAAGTCAAGCCATAATGAATTTGTGCGAGAATTTTGGGAGGAGACGTTCAACTGTTATCTTGAAGACAGCCCAAGGAATGCTGATAGTGAGAATGGCAGTACGAGTTTCAGGCCGCTGTGTACTGGGGAAGAGGACATTGCCAGTGTTGAGACGCCATATTTGGACTACACGCACCTCAGGATTTCCTATAACGTGTATGTGGCTGTTTATGCCATAGCACAGGCACTTCAGGACATACTTACCTGCACTCCAGGGAAAGGATTGTTTTCTAATGGCTCTTGTGCAGATATTAGGAAAGTTGAAGCTTGGCAGGTTCTGAAACAACTAAGACACCTCAACTTCATAGATAGCATGGGGGAGAGAGTGCGCTTTGACAACGGCAGTGAGCTTTCAGCCAATTATACCATCATAAACTGGCATCGATCACCCGAGGATGGGTCTGTCGTATTCAAGGAGGTTGGCTATTACAGTATACATAACAAGAACGTGGCCAAGCTTTCCATTGACAAGAGCAAAATCCTGTGGAACGGACGTCTAACTGAGGTACCATTTTCCAACTGTAGTGTGGAATGTGAACCTGGAACAAGGAAAGGAATTATTGATGGCGAACCCACATGTTGCTTTGAGTGTACAGAATGCTCGGATGGGGAGTACAGTGATCATAAAGATGCCAGTTTTTGCGTTAAATGCCCAAACAACTCCTGGTCCAACGGCAATCACACTTCTTGCTTTCTGAAGCAAATCGAGTTTCTGTCCTGGACCGAACCGTTCGGGATTGCGCTGGCCTTATTTGCAGTCCTCGGGGTTCTCCTAACAGCTTTTGTGTTGGGTGTTTTTGTGCAATTCCGTGATACTCCAATCGTGAAGGCATCAAACCGAGAGCTGTCGTTTCTTTTGCTTTTCTCGCTTATATGCTGTTTCTCCAGCTCTCTCATATTCATAGGCGAACCACAGGATTGGACGTGCCGGGTACGCCAACCAGCATTCGGCATCAGCTTTGTGTTATGCATCTCATGCATTCTAGTCAAAACCAATCGTGTCCTGCTGGTGTTCGAAGCTAAAATCCCTACGAGTCTCCACCGTAAGTGGTGGGGATTAAACCTGCAGTTCTTGCTAGTCTTCTTGTTCACGTTTGTGCAGGTGATGATCTGCGTGGTTTGGTTGTACAACGCTCCGCCTGGAAGTTACAAGAACTACGACATCGATGAAATCATCTTCATCACCTGCAACGAGGGCTCCATGATGGCTCTGGGGTTTCTGATTGGCTACACGTGCTTGTTGGCCGCCATCTGTTTTTTCTTCGCGTTCAAGTCTCGGAAGCTGCCGGAGAATTTCACGGAGGCCAAATTCATAACGTTCAGCATGCTGATTTTTTTCATCGTCTGGATCTCCTTTATTCCGGCGTACTTCAGCACTTACGGCAAATTCGTCTCGGCTGTTGAGGTCATAGCAATCCTGGCGTCCAGCTTCAGTTTGCTTGCCTGTATCTTTTTCAACAAGGTGTATATCATTCTTCTGAAACCGTCGAGGAATACGATCGAGGAGGTGCGCTGTAGCACTGCGGCTCACGCCTTTAAAGCTGCAGCAAAAGCGACTTTACGACACAGTTCAGGCTTTAGAAAGAGATCCAGCAGTGTTGGCGGCTCCTCCGCATCTTCGCCCTCCTCATCCATCAGCATGAAGACCAACGGGAATGAAATGGAGTCACCGTCTACGCGGAGACACGGCTCAAAACCGAGGGTGAGCTTTGGAAGTGGGACGGTCAGTCTCTCCTTGAGCTTCGAGGAGGCTAGAAACTCGATGAAA

**References**

1. Guindon S, Dufayard JF, Lefort V, Anisimova M, Hordijk W, Gascuel O: **New algorithms and methods to estimate maximum-likelihood phylogenies: assessing the performance of PhyML 3.0**. *Syst Biol* 2010, **59**(3):307-321.

2. Alioto TS, Ngai J: **The repertoire of olfactory C family G protein-coupled receptors in zebrafish: candidate chemosensory receptors for amino acids**. *BMC Genomics* 2006, **7**:309.

3. Hashiguchi Y, Nishida M: **Evolution and origin of vomeronasal-type odorant receptor gene repertoire in fishes**. *BMC Evol Biol* 2006, **6**:76.
