# Supplementary figures and images for: Overlapping but distinct topology for zebrafish V2R-like olfactory receptors reminiscent of odorant receptor spatial expression zones
Source: BMC Genomics. 2018 May 23;19:383. doi: 10.1186/s12864-018-4740-8 (PMC5966872; doi:10.1186/s12864-018-4740-8)

A

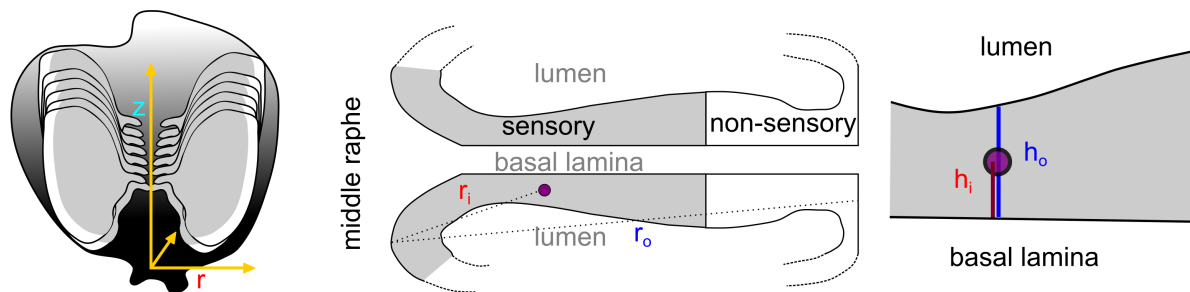

B

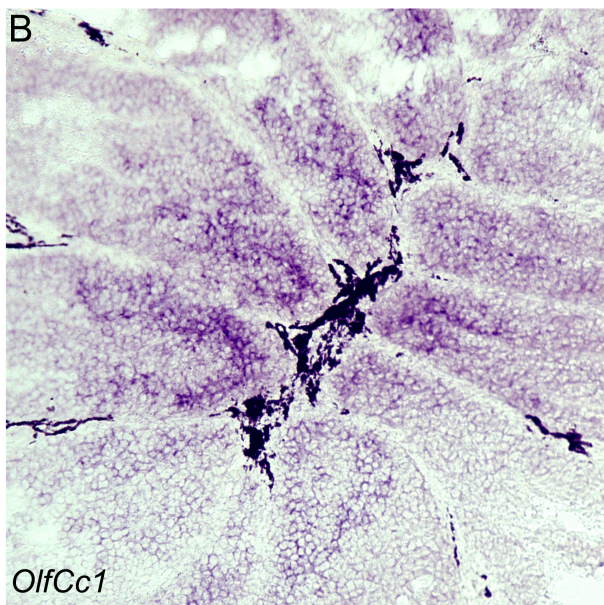

C

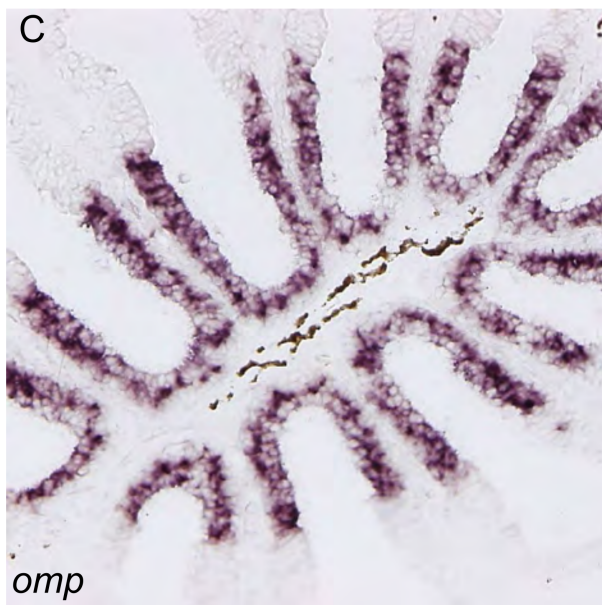

D

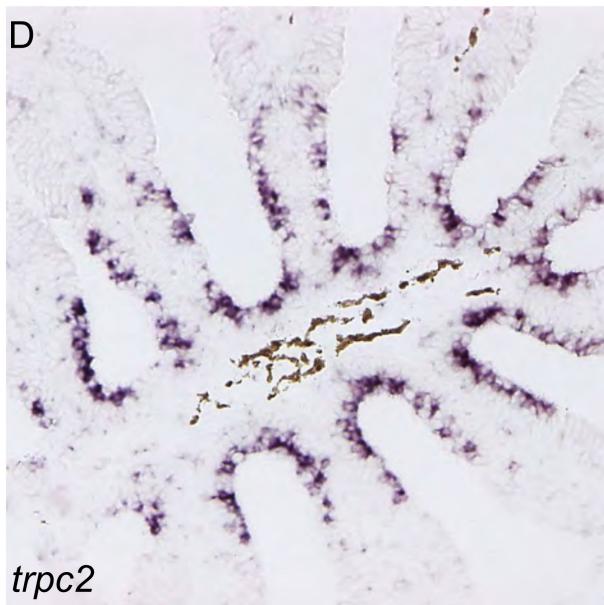

E

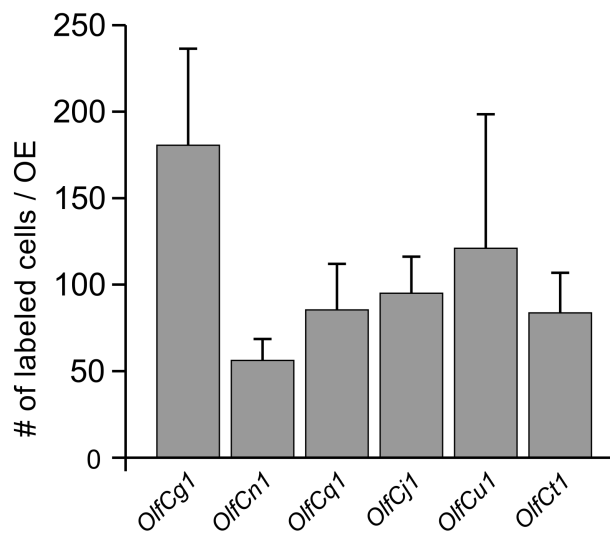

Supplement: Supplementary file 3 — Quantitative in situ hybridisation of OlfC-expressing cells. (A-C) Comparison of OlfCc1 expression with that of olfactory neuron marker genes OMP and TRPC2. Labeled cells were exclusively detected in the sensory region of the adult olfactory epithelium. OlfCc1 distribution is similarly apical as TRPC2 and more apical than OMP, the marker for ciliated neurons. (D) Expression frequency for six different OlfC genes. The bar graphs represent the number of cells observed for a particular OlfC gene in the complete olfactory organ comprising 40–60 sections (mean +/− SEM, n = 3–5 olfactory organs). (PDF 12927 kb) [file 12864_2018_4740_MOESM3_ESM.pdf]
