# Supplementary material for: Overlapping but distinct topology for zebrafish V2R-like olfactory receptors reminiscent of odorant receptor spatial expression zones
Source: BMC Genomics. 2018 May 23;19:383. doi: 10.1186/s12864-018-4740-8 (PMC5966872; doi:10.1186/s12864-018-4740-8)
Supplement: Supplementary file 5 — Interspecies comparison of spatial expression patterns. Interspecies comparison of spatial distributions of several OR and V2R genes for adult zebrafish, larval Xenopus laevis, adult mouse and adult rat. The respective species is represented graphically, olfactory organ and the olfactory receptor gene family examined are noted below. The respective genes are indicated by color code, which is unique within each panel; gene names are as given in the respective publications. Spatial distributions are represented as ECDF (y axis). For the radial distribution, the x-axis represents the normalized radial distance with a scale ranging from 0 (central) to 1 (peripheral); for the height distribution the x-axis represents the normalized laminar height with a scale ranging from 0 (basal) to 1 (apical). For Xenopus V2R [25, 41] and zebrafish OR [10] the raw data of the respective publications were used to generate the graphs. Original gene names used in [10] were fZOR6 (or112–1), fZOR9 (or107–1), fZOR8 (or103–1), and fZOR5 (or102–1). Note the structural similarities between different species, olfactory organs, and olfactory receptor families. (PDF 557 kb) [file 12864_2018_4740_MOESM5_ESM.pdf]

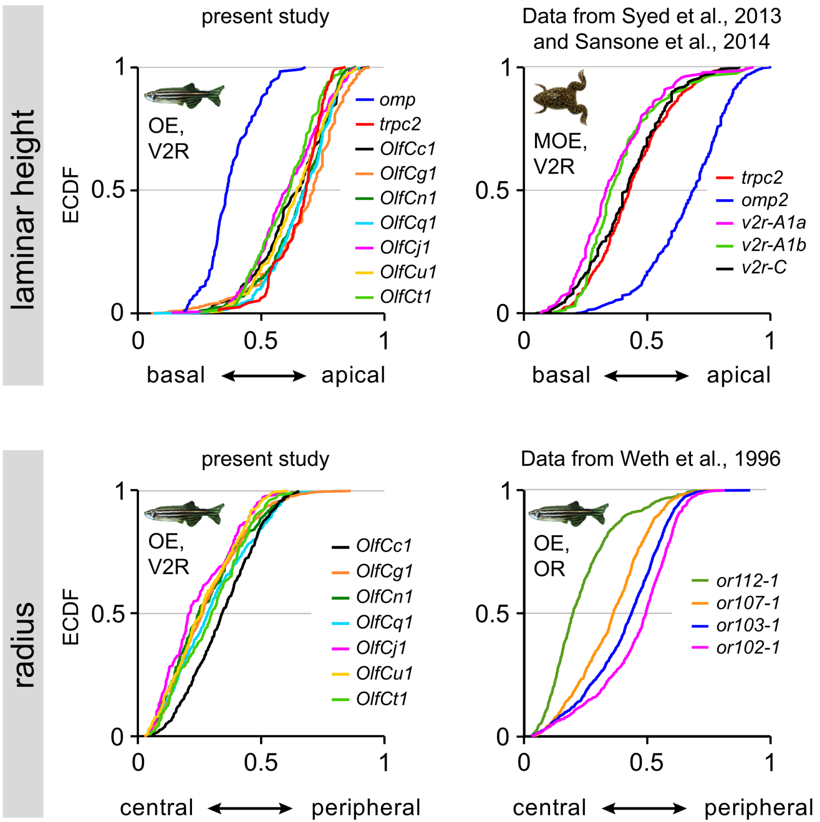
laminar height
present study
OE,
V2R
ECDF
omp
trpc2
OlfCc1
OlfCg1
OlfCn1
OlfCq1
OlfCj1
OlfCu1
OlfCt1
basal
apical
Data from Syed et al., 2013
and Sansone et al., 2014
MOE,
V2R
trpc2
omp2
v2r-A1a
v2r-A1b
v2r-C
radius
present study
OE,
V2R
OlfCc1
OlfCg1
OlfCn1
OlfCq1
OlfCj1
OlfCu1
OlfCt1
central
peripheral
Data from Weth et al., 1996
OE,
OR
or112-1
or107-1
or103-1
or102-1
0
0.5
1
